# NeoCircle: pre- and post-operative circulating tumor DNA dynamics predicts survival in neoadjuvant-treated early breast cancer

**DOI:** 10.1038/s44321-026-00447-z

**Published:** 2026-05-26

**Authors:** Anthony M George, Yilun Chen, Sergii Gladchuk, Miguel Alcaide, Hina Dalal, Pei Meng, Christian Brueffer, Hani Saghir, Siker Kimbung, Kristina Aaltonen, Lucia Oton, Christopher Rushton, Sofia Birkeälv, Mats Jönsson, Sophia Zackrisson, Ida Skarping, Daniel Förnvik, Lina Zander, Gabriella Honeth, Samuel Woodhouse, Karen Howarth, Åke Borg, Anna Ehinger, Martin Malmberg, Lisa Rydén, Niklas Loman, Lao H Saal

**Affiliations:** 1 Division of Oncology, Department of Clinical Sciences, Lund University, Lund, Sweden; 2 Lund University Cancer Center, Lund, Sweden; 3 Skåne University Hospital Comprehensive Cancer Center, Lund, Sweden; 4 Center for Interdisciplinary Research on Cancer and Equity in Women, Lund University, Lund, Sweden; 5 SAGA Diagnostics, Morrisville, NC USA; 6 Diagnostic Radiology, Department of Translational Medicine, Lund University, Malmö, Sweden; 7 Department of Imaging and Physiology, Skåne University Hospital, Malmö, Sweden; 8 Medical Radiation Physics, Department of Translational Medicine, Lund University, Malmö, Sweden; 9 Department of Hematology, Oncology, and Radiation Physics, Skåne University Hospital, Lund/Malmö, Sweden; 10 Department of Pathology, Skåne University Hospital, Lund, Sweden; 11 Department of Surgery, Skåne University Hospital, Lund, Sweden

**Keywords:** Biomarkers, Cancer

## Abstract

Persistent circulating tumor DNA (ctDNA) during neoadjuvant treatment (NAT) of early breast cancer (EBC) indicates high-risk disease. Similarly, detection of ctDNA post-resection indicates molecular residual disease (MRD) and impending relapse. For ctDNA to be integrated into EBC management, accessible and scalable diagnostics are required. Here we apply an ultrasensitive, personalized tumor-informed approach to ctDNA evaluation predicated on analyses of structural variants (SVs) using a novel digital PCR (dPCR) multiplex SV technology. 136 patients eligible for NAT (29.4% TNBC, 44.9% HR+ /HER2− and 24.3% HER2+), enrolled between December 2014 and March 2019, were analyzed from the prospective SCAN-B study (NCT02306096, substudy NeoCircle). ctDNA detection at baseline was 89.7%; end-NAT ctDNA-positivity (21.4%) and NAT ctDNA-non-response (13.1%) were significant predictors of disease recurrence and death, and both superior to pathologic complete response. Detection of ctDNA post-operatively or during adjuvant monitoring was significantly associated with distant recurrence (median lead-time 13.8 months, range 0–47.7 months). These findings validate SVs as an MRD analyte and provide evidence for clinical use of this approach in EBC.

## Synopsis

For 136 patients with early breast cancer, 1497 plasma samples were collected during neoadjuvant therapy and post-operative adjuvant therapy follow-up (median 6.5 years) and analyzed for ctDNA using an ultrasensitive tumor-informed structural variant-based personalized multiplex dPCR approach.

- High baseline ctDNA detection was seen in the study (89.7%), with tumor fraction levels down to as low as 0.000136% (1.36 parts per million).
- Lack of total ctDNA clearance after neoadjuvant therapy was associated with worse breast cancer-free interval (BCFi; HR 3.3, 95% CI 1.4–7.9; *p* = 0.004) and overall survival (OS; HR 5.6, 95% CI 1.9–16.7; *p* = 0.0006), and lack of dynamic response to neoadjuvant therapy was associated to worse BCFi (HR 9.4, 95% CI 3.8 to 23.7; *p* = 0.004) and OS (HR 11.9, 95% CI 3.9 to 35.9; *p* < 0.00001).
- Post-operative sensitivity for distant recurrence was 86.7%, and 92.9% when considering only pathologically confirmed relapses.
- ctDNA detection during follow-up was strongly associated with poor BCFi (HR 37.9, 95% CI 12.7 to 113.6; *p* < 0.00001) and OS (HR 12.7, 95% CI 4.1–39.2; *p* < 0.00001).
- Molecular residual disease detection preceded clinical relapse by up to 4 years (median lead time of 13.8 months).

## Abstract (web summary)

For 136 patients with early breast cancer, 1497 plasma samples were collected during neoadjuvant therapy and post-operative adjuvant therapy follow-up (median 6.5 years) and analyzed for ctDNA using an ultrasensitive tumor-informed structural variant-based personalized multiplex dPCR approach.

## Graphical abstract

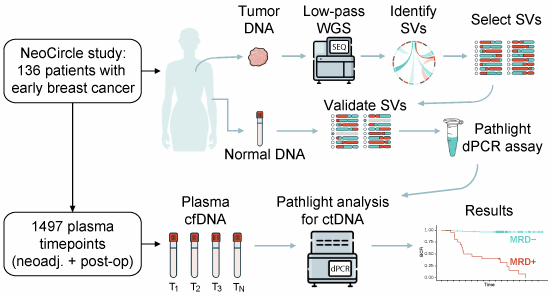

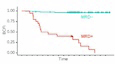

The paper explained **Problem**

Despite advancements in treatment of patients with early breast cancer, current standards for monitoring patients with high accuracy during neoadjuvant treatment, post-surgery, and surveillance are lacking, leading to poorly personalized care. Circulating tumor DNA (ctDNA) is a biomarker that is strongly prognostic, but comprehensive clinical utility data in early breast cancer has yet to be demonstrated. To integrate such testing into routine clinical management requires assays that are sufficiently sensitive and available at scale.

**Results**

In this study, we monitored patients with early breast cancer throughout neoadjuvant and adjuvant treatment, with a median follow-up of 6.5 years, using an ultrasensitive tumor-informed structural variant (SV)-focused ctDNA analysis method. Baseline ctDNA detection was very high, and ctDNA-negativity at the end of neoadjuvant treatment and dynamic ctDNA response during neoadjuvant treatment were highly prognostic. Similarly, patients who had a positive ctDNA detection result post-operatively or during adjuvant treatment, signifying molecular residual disease (MRD), were significantly more likely to experience recurrence and death. The median lead-time between a positive result from the MRD assay and clinical detection of recurrence was 13.8 months, and in some patients was nearly 4 years.

**Impact**

Ultrasensitive analysis of ctDNA and MRD using an SV-based approach is feasible at scale, is highly prognostic, and can detect occult metastasis well in advance of clinical relapse which may enable treatment adaptation to potentially improve outcomes for patients with breast cancer.

## Introduction

Neoadjuvant treatment (NAT) in early breast cancer (EBC) increases the rate of breast preservation, and the response to treatment can provide prognostic information: achieving a pathologic complete response (pCR) is associated with improved overall and event-free survival (Spring et al, 2020). While pCR is an established prognostic marker, its effect size is modest and the non‑pCR majority is prognostically heterogeneous. Cell-free circulating tumor DNA (ctDNA) analysis is emerging as a powerful tool that not only provides prognostic insight but also enables real-time disease monitoring in the neoadjuvant, adjuvant, and metastatic settings (Stroun et al, 1989; Jung et al, 2010; Leary et al, 2010; McBride et al, 2010; Dawson et al, 2013; Bettegowda et al, 2014; Newman et al, 2014; Wan et al, 2017; Cutts et al, 2024). Persistent ctDNA detection during NAT of EBC indicates high-risk disease, and persistent or re-emerging ctDNA positivity after surgical resection, signaling molecular residual disease (MRD), indicates presence of occult metastatic disease and heralds impending disease relapse (Garcia-Murillas et al, 2015; Olsson et al, 2015; Magbanua et al, 2021). However, integrating ctDNA monitoring into routine clinical management of EBC will require assays that are not only highly sensitive and specific, but also accessible and scalable.

Current ctDNA analysis technologies are broadly classified as being either tumor-informed or tumor-naïve (also known as tumor-agnostic). Tumor-informed approaches begin with sequencing of the patient’s tumor tissue to identify tumor-specific alterations, which are then tracked in liquid biopsy cell-free DNA (cfDNA). In contrast, tumor-naïve assays rely on a fixed panel of recurrent genomic or epigenomic alterations that are not personalized to the patient’s tumor. While tumor-naïve approaches are valuable when tumor tissue is limited or unavailable, tumor-informed approaches have demonstrated superior sensitivity and specificity for longitudinal ctDNA monitoring (Martinez-Castedo et al, 2025; Camblor et al, 2026).

**Figure 1:**
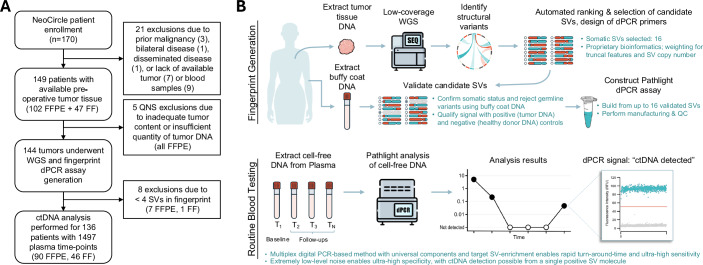
CONSORT diagram and schema of structural variant (SV)-based ctDNA analysis method. (**A**) Diagram illustrates NeoCircle patient enrollment, exclusions, and patients and samples for tumor-informed whole-genome sequencing (WGS), multiplex digital PCR (dPCR) fingerprint assay generation, and ctDNA analyses. FF = fresh frozen tumor specimen from diagnostic biopsy; FFPE = formalin-fixed paraffin-embedded tumor from diagnostic biopsy; QNS = quality/quantity not sufficient. (**B**) Schema outlining the tumor-informed SV-based multiplex dPCR method for detection and quantification of ctDNA in liquid biopsies.

Across both tumor-informed and tumor-naïve approaches, most ctDNA assays focus on the detection of single-nucleotide variants (SNVs) (Chen et al, 2021). Here we describe a distinct tumor-informed ctDNA assay that utilizes low-coverage whole-genome sequencing (WGS) of the patient’s tumor tissue to identify tumor-specific structural variants (SVs) (Olsson et al, 2015; Elliott et al, 2025), such as chromosomal translocations, rearrangements, inversions, and large deletions, which arise from cancer-associated genomic instability (Mertens et al, 2015; Cosenza et al, 2022). Based on the enumeration of patient- and tumor-specific SVs, a multiplex dPCR SV fingerprint assay is constructed for analysis of plasma-derived cfDNA to measure ctDNA (Fig. 1).

SVs have numerous characteristics that make them highly suitable as tumor biomarkers. For example, it is understood that many SVs form early in tumorigenesis during the ‘telomere crisis’, the period when rapidly dividing pre-malignant cells experience telomere shortening that leads to chromosome end fusions, double-strand breakage, and erroneous repair (Chin et al, 2004). As a consequence of the early nature of the telomere crisis, the resulting SVs are often truncal (Tang et al, 2015). Additionally, such SV formation is largely stochastic and does not necessarily impact oncogenes or tumor suppressors, nor confer cell-fitness advantages; thus, these SVs are less susceptible to negative selection under anti-cancer therapeutic pressure and allowing them to persist during tumor progression (Tang et al, 2015). Moreover, SVs frequently co-occur with copy number gains, increasing their abundance within each tumor cell and enhancing the signal available for detection in liquid biopsies. Unlike single-nucleotide variants (SNVs), which alter only a single base and are more prone to detection challenges due to artifactual noise that can mimic the appearance of true SNVs (e.g., heat-, chemical-, or sonication-mediated DNA damage during sample handling and preparation, and analytical and equipment noise seen in all sequencing methods), SVs comprise exquisitely unique breakpoint sequences that are significantly less likely to arise from such noise, thus conferring SVs’ superior specificity. Furthermore, tumor-specific SVs are not associated with clonal hematopoiesis of indeterminate potential (CHIP), a well-known age-related process in which primarily SNVs accumulate in immune cells, including in typical cancer genes like *TP53*, and thus can mimic tumor-derived SNVs in cell-free DNA (cfDNA). Together, these features of early emergence, persistence, abundance, and cancer- and technical-specificity make SVs ideal “fingerprint” biomarkers for sensitive and reliable cancer monitoring in cfDNA liquid biopsies.

Our SV-based ctDNA approach is also distinguished by its use of digital PCR (dPCR) for ctDNA detection, whereas most ctDNA assays rely on next-generation sequencing (NGS) (Chen et al, 2021). Compared with NGS, dPCR offers faster turnaround times, simpler and more cost-effective workflows, tolerance of a broad range of cfDNA input concentrations, and substantially reduced computational and analytical requirements. Historically, adoption of dPCR for tumor-informed ctDNA detection for MRD at clinical scale has been limited by technical constraints, including incomplete utilization of available cfDNA due to small per-reaction input volumes and instrument dead volumes, restricted numbers of fluorescent channels that cap the number of trackable targets, and challenges related to assay design turnaround time and scalability. Pathlight^TM^ overcomes these limitations through a proprietary, SV-optimized analytical method that combines near-complete utilization of available cfDNA, targeted enrichment of tumor-specific SVs, unique dPCR chemistry, and exceptionally low background noise. Together, these features enable reliable single-molecule ctDNA detection, multiplex tracking of up to 16 tumor-specific targets, and rapid, automated, and scalable development of personalized assays suitable for routine clinical use.

NeoCircle is a prospective clinical study for ctDNA monitoring of patients with EBC eligible for NAT that enrolled patients between December 2014 and March 2019 as a subproject within the large ongoing population-based SCAN-B initiative (ClinicalTrials.gov identifier NCT02306096) (Saal et al, 2015). NeoCircle was initiated to further evaluate the clinical validity of SV-based ctDNA detection in EBC (Olsson et al, 2015), evaluate the usefulness of this approach for assessment of response to NAT and its prognostic value, and validate the approach to detect MRD during post-operative follow-up.

Here we report on the ctDNA dynamics during regular monitoring throughout both neoadjuvant and adjuvant treatment and on clinical outcomes after a median follow-up from diagnosis of 6.5 years for 136 patients and 1497 plasma timepoints within the NeoCircle study. In this study, we demonstrate the feasibility of multiplex dPCR for SV detection in blood plasma and monitoring ctDNA at scale, and through both planned and exploratory analyses, report on ctDNA detection before, during, and after NAT as well as post-surgical ctDNA monitoring during adjuvant follow-up and the relationship of ctDNA measurements to clinical outcomes in EBC.

## Results

### Prospective enrollment of NeoCircle patients

The NeoCircle study is a real-world cohort of prospectively enrolled patients planned for pre-operative chemotherapy with a curative intent for EBC (see Methods). Between December 2014 and March 2019, 170 patients were enrolled.

After patient exclusions due to disseminated disease, prior malignancy, bilateral disease, or unavailable tissue or blood samples, 149 patients had requisite pre-operative tumor tissue samples available for tumor-informed analysis, of which 136 cases proceeded to ctDNA analysis (13 cases, 8.7%, could not proceed to plasma analysis due to technical reasons; Fig. 1A). The clinicopathological characteristics for the 136-patient cohort for ctDNA analysis are presented in Table 1. In summary, the analyzed patients reflected the expected population distribution with 44.9% being hormone receptor positive and HER2 negative (HR+ /HER2−), 29.4% triple-negative (TNBC), and 24.3% HER2 positive (HER2+), the median age at diagnosis being 52 years (range 24–78 years), 47.1% lymph node negative, and the majority of cases clinical stage II (80.9%). All patients were treated according to Swedish national guidelines, and at time of database lock, the patients were followed for a median of 6.5 years (78.0 months) from diagnosis (range 17.5–115.9 months) and 6.0 years (72.3 months) post-surgery (range 12.4–110.3 months), with a median of 11 plasma timepoints per patient (range 6–14 timepoints/patient) (see Appendix Table S1).

**Table 1:** Characteristics of 136-patient cohort.

*Patient age (years)*		*Clinical subtype*	
Median (range)	52 (24–78)	HR+/HER2−	61 (44.9%)
*Clinical stage*		HER2+	33 (24.3%)
IA	6 (4.4%)	TNBC	40 (29.4%)
IIA	62 (45.6%)	n/a	2 (1.5%)
IIB	48 (35.3%)	*Neoadjuvant regimen*	
IIIA	13 (9.6%)	Any Chemotherapy	136 (100%)
IIIC	1 (0.7%)	Any Endocrine	1 (0.7%)
n/a	6 (4.4%)	Any Anti-HER2	33 (24.3%)
*Tumor size (mm)*		Any Supportive	33 (24.3%)
Median (range)	11 (0–70)	*Adjuvant regimen*	
*Lymph node status*		Any RT	118 (86.8%)
Negative	64 (47.1%)	Any Endocrine	85 (62.5%)
1 to 3	52 (38.2%)	Any Chemo	9 (6.6%)
≥4	17 (12.5%)	Any Anti-HER2	35 (25.7%)
n/a	3 (2.2%)	Any Supportive	58 (42.6%)
*ER status*		*Follow-up time (months after diagnosis)*	
Positive	82 (60.3%)	Median (range)	78.0 (17.5–115.9)
Negative	52 (38.2%)	*Follow-up time (months after surgery)*	
n/a	2 (1.5%)	Median (range)	72.3 (12.4–110.3)
*PgR status*		*Relapse event (BC-specific)*	
Positive	70 (51.5%)	Distant relapse	15 (11.0%)
Negative	64 (47.1%)	Local-only relapse	4 (2.9%)
n/a	2 (1.5%)	CNS-only relapse	2 (1.5%)
*HER2 clinical status*		None	115 (84.6%)
Positive	33 (24.3%)	*Death event*	
Negative	101 (74.3.0%)	Yes	13 (9.6%)
n/a	2 (1.5%)	No	123 (90.4%)
*Nottingham histologica*	*l grade*		
Grade 1	9 (6.6%)		
Grade 2	48 (35.3%)		
Grade 3	17 (12.5%)		
n/a	62 (45.6%)		

Note: patient age, clinical stage, ER, PgR, and HER2 are from the workup at diagnosis and diagnostic biopsy, whereas tumor size, lymph node status, and Nottingham histological grade based on the surgical specimen. For ER, PgR, and HER2, if missing for the diagnostic biopsy, the result from the surgical specimen is used. Two patients with clinically HER2-negative tumors recieved adjuvant anti-HER2 therapy: one patient had a HER2-negative tumor at diagnostic biopsy, but the residual tumor was HER2-positive at surgery and she received adjuvant anti-HER2 treatment; one patient had a focally HER2-positive (< 10%) tumor, thus clinically HER2-negative but she received adjuvant anti-HER2 treatment.

*BC* breast cancer, Chemo chemotherapy, *CNS* central nervous system, *ER* estrogen receptor, *HER2* human epidermal growth factor receptor 2, *n/a* not available, *PgR* progesterone receptor, *RT* radiotherapy.

### Tumor-informed SV breakpoint assay design

A tumor-informed ctDNA approach that uses low-coverage WGS to identify tumor-specific SVs was employed (see Methods) (Fig. 1B). In brief, from the WGS data, candidate SVs were identified and ranked according to proprietary bioinformatic algorithms. For fresh-frozen (FF) tumor tissue as starting material for WGS, up to 8 SVs were selected for each fingerprint (research test, SAGA Diagnostics), and for formalin-fixed paraffin-embedded (FFPE) tumor tissue as starting material for WGS, up to 16 SVs were selected for each patient-specific fingerprint (Pathlight^TM^, SAGA Dx Inc), and the selected SVs were used to construct a multiplex dPCR assay with target-SV enrichment personalized for each patient (Elliott et al, 2025).

**Figure 2:**
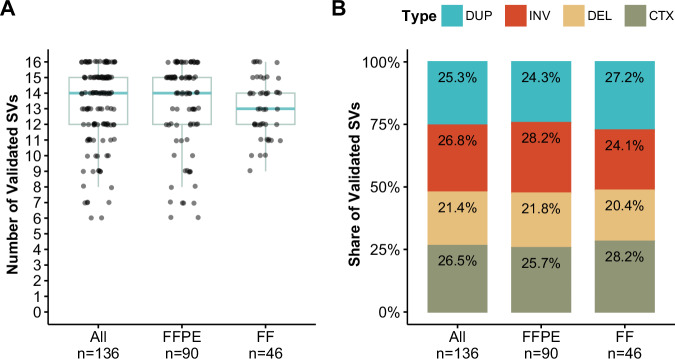
SV validation rates and distribution of SV types. (**A**) Boxplot of the number of SVs validated per patient, as well as subdivided by tumor-informed FFPE (formalin-fixed paraffin-embedded) or FF (fresh frozen) workflow. (**B**) Stacked bar plot illustrating similar distribution of SV types. DUP = duplication; INV = inversion; DEL = deletion; CTX = inter-chromosomal translocation. All values are plotted in the boxplot, with the median (center line), interquartile 75th to 25th percentile range (box), and 1.5 times the interquartile range (whiskers) indicated.

We have previously demonstrated the Pathlight method to have an estimated limit of detection (LOD) at 95% certainty of 0.00052% tumor fraction (5 parts per million, PPM) with SVs detected as low as 1 part in 10 million (0.00001% or 0.1 PPM) in analytical validation studies, as well as 100% analytical specificity across 5268 SV measurements of 217 control cfDNA samples and 217 control normal buffy coat DNA samples (Elliott et al, 2025). Summarizing the WGS analysis of 136 breast tumors and generation of multiplex dPCR assays, a median of 14 SVs were validated per case (Fig. 2A), including inversions, deletions, duplications and translocations, and with the number and distribution of SVs being similar for both FF and FFPE workflows (Fig. 2B).

### ctDNA dynamics during neoadjuvant therapy

**Figure 3:**
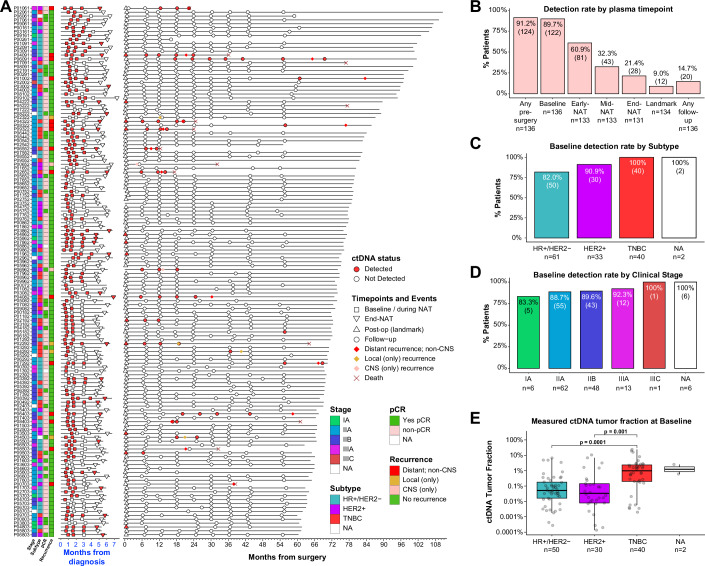
ctDNA detection across all patients. (**A**) Swimmer plot of all 136 patients analyzed for ctDNA, ordered by date of enrollment. Clinical Stage, breast cancer Subtype, pathologic complete response (pCR) status, and Recurrence are annotated according to the color key. The neoadjuvant treatment (NAT) period is plotted in months from diagnosis, and the post-surgical adjuvant treatment period is plotted in months from surgery. CNS = central nervous system; NA = not available. (**B**) Bar graph of ctDNA detection rate by plasma timepoint illustrating high detection rates. (**C**) Bar graph of baseline ctDNA detection rate by breast cancer subtype at diagnosis. (**D**) Bar graph of baseline ctDNA detection rate by clinical stage at diagnosis. (**E**) Boxplot of baseline ctDNA tumor fraction by breast cancer subtype at diagnosis. *P*-values calculated using the Mann–Whitney U test. All values are plotted in the boxplot, with the median (center line), interquartile 75th to 25th percentile range (box), and 1.5 times the interquartile range (whiskers) indicated.

For the 136 patients where minimum quality control criteria were met for the final ctDNA analysis, 1497 plasma samples were analyzed using personalized multiplexed dPCR SV fingerprint assays following a pre-specified analysis plan (Figs. 1A and 3A). All analyses presented were specified in the analysis plan, unless otherwise noted herein as exploratory.

**Figure EV1:**
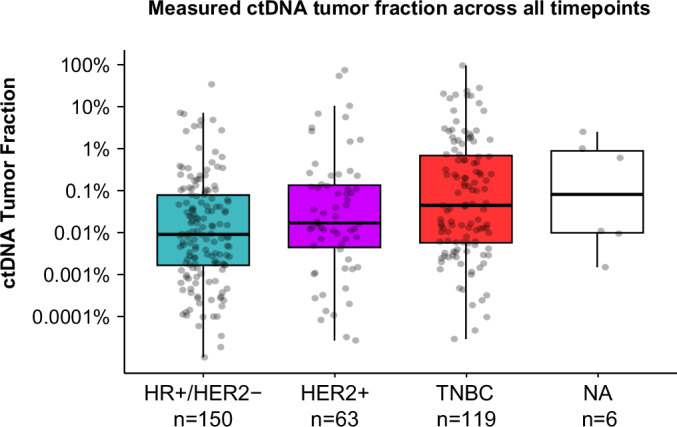
Measured ctDNA tumor fraction across all timepoints. Boxplots of every ctDNA tumor fraction measured across all 1497 plasma samples for all patients, according to the tumor clinical subtype.

At the baseline plasma timepoint, taken at the time of diagnostic biopsy prior to any therapy, ctDNA was detected in 122/136 patients (89.7%; Fig. 3B). Across all patients, baseline ctDNA detection varied by stage and subtype, with the highest baseline detection rate of 100% in patients with TNBC disease, 90.9% in HER2+, 82.0% in HR+/HER2−, and in 2 of 2 patients with undetermined subtype (Fig. 3C), with the detection rates generally increasing with clinical stage (from 83.3% in stage IA disease to 100% in stage IIIC; Fig. 3D). Among the 122 cases with ctDNA detected at baseline, the median tumor fraction (TF) was 0.11% (range 0.000136% to 23.8% TF), with significantly higher measured levels in TNBC compared to HR+/HER2− or HER2+ (*p* ≤ 0.001; Fig. 3E). Across all 1497 plasma sample timepoints, ctDNA was detected in 338 timepoints and the ctDNA TF levels tended to be higher in TNBC, moderate in HER2+, and lower in HR+/HER2− (Fig. EV1).

**Figure EV2:**
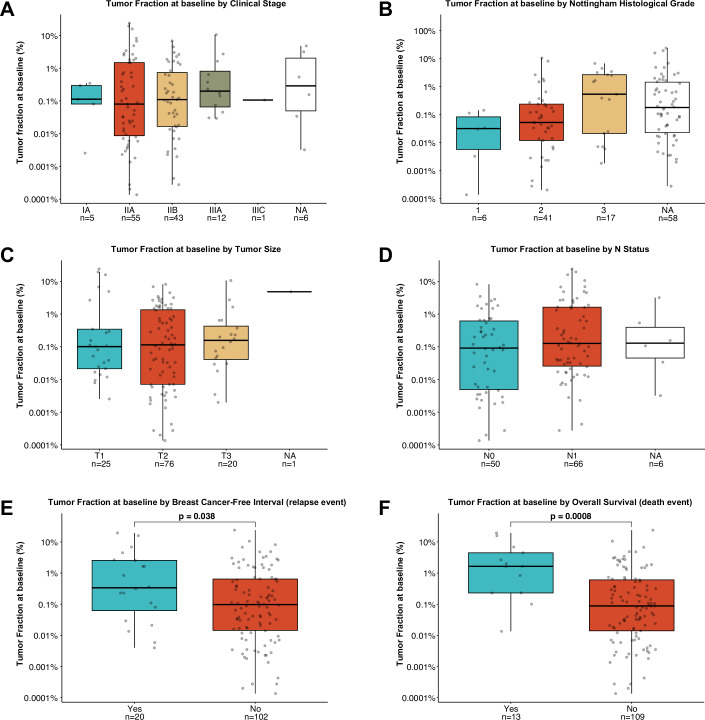
Measured ctDNA tumor fraction at baseline timepoint by clinicopathological categories. (**A**) Tumor fraction boxplots at baseline by clinical stage. (**B**) Tumor fraction boxplots at baseline by Nottingham Histological Grade. (**C**) Tumor fraction boxplots at baseline by clinical Tumor size. (**D**) Tumor fraction boxplots at baseline by clinical Node stage. (**E**) Tumor fraction boxplots at baseline by breast cancer-free interval. (**F**) Tumor fraction boxplots at baseline by overall survival. *P*-values calculated using the Mann–Whitney U test.

As an exploratory analysis, baseline ctDNA TF levels were examined in the context of additional clinicopathological variables: ctDNA TF at baseline did not vary by clinical stage, T size, or N status at diagnosis; however, the baseline ctDNA TF levels were significantly higher in patients aged ≤50 at diagnosis (*p* = 0.021), as well as among those who had an eventual recurrence (*p* = 0.038) or death (*p* = 0.0008) event (Fig. EV2A–F).

**Figure 4:**
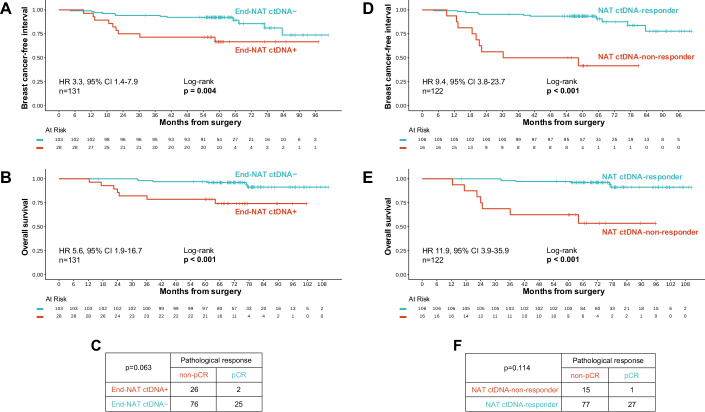
Detection of ctDNA at end-NAT and pathological response. Kaplan-Meier plot illustrating that end-NAT ctDNA status is a significant predictor of breast cancer-free interval (**A**) and overall survival (**B**). (**C**) Contingency table showing lack of significant association between end-NAT ctDNA status and pathologic complete response (pCR) status. Kaplan-Meier plot illustrating that NAT ctDNA-responder status is a significant predictor of breast cancer-free interval (**D**) and overall survival (**E**). (**F**) Contingency table showing lack of significant association between NAT ctDNA-responder status and pCR status. Survival analysis *p*-values calculated using the log-rank test, and contingency table *p*-values calculated using the Fisher’s exact test.

**Figure EV3:**
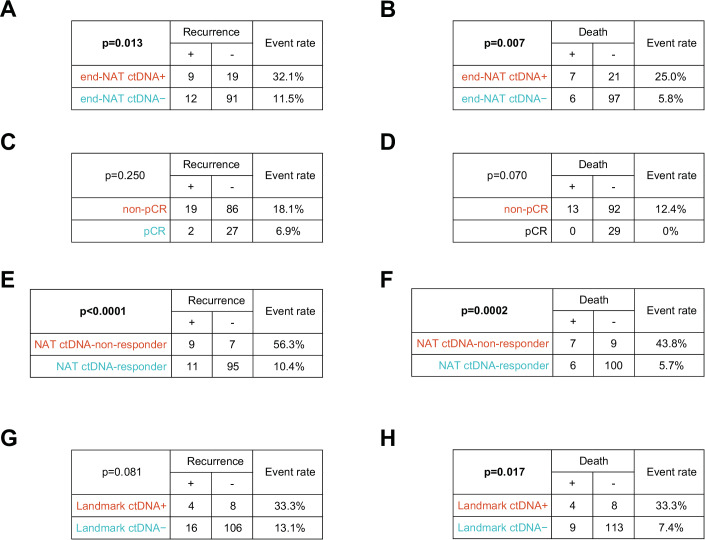
Contingency tables for pCR and ctDNA variables versus survival outcomes. End-NAT ctDNA status versus recurrence (**A**) and death (**B**). pCR status versus recurrence (**C**) and death (**D**). NAT ctDNA responder status versus recurrence (**E**) and death (**F**). Landmark ctDNA status versus recurrence (**G**) and death (**H**). *P*-values calculated using the Fisher’s exact test.

**Figure EV4:**
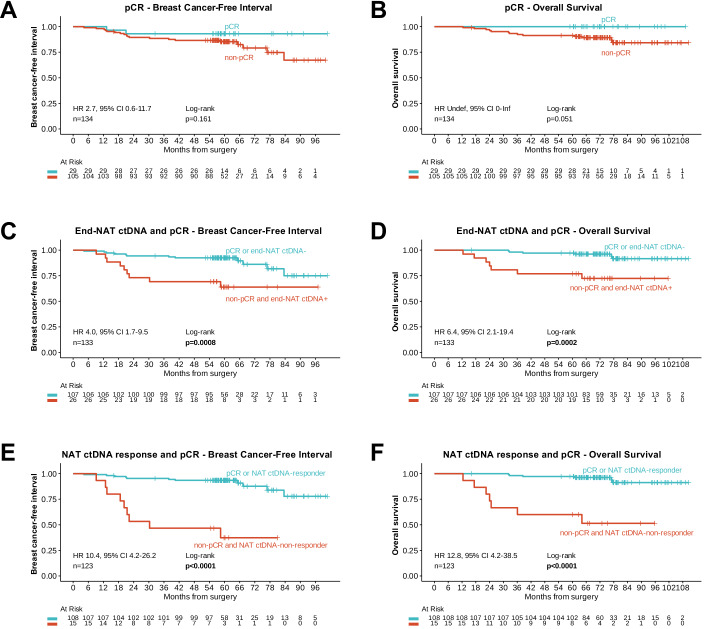
Kaplan–Meier survival estimates for all patients. For breast cancer-free interval (**A**, **C**, **E**) and overall survival (**B**, **D**, **F**), survival curves are plotted for: (**A**, **B**) pCR, (**C**, **D**) End-NAT ctDNA and pCR combined, and (**E**, **F**) NAT ctDNA response and pCR combined. *P*-values calculated using the log-rank test.

For nearly all patients, ctDNA levels generally decreased during NAT, however, for 21.4% of patients (28/131) remained detectable at the end-of-NAT timepoint (defined as the final timepoint after the last administration of NAT and prior to surgery; Fig. 3B; Appendix Fig. S1). Lack of ctDNA clearance at the end-of-NAT timepoint (end-NAT ctDNA+) was a significant predictor of poor breast cancer-free interval (BCFi; hazard ratio [HR] 3.3, 95% confidence interval [CI] 1.4 to 7.9; log-rank *p* = 0.004; Fig. 4A) and overall survival (OS; HR 5.6, 95% CI 1.9 to 16.7; *p* = 0.0006; Fig. 4B), with 32.1% versus 11.5% experiencing recurrence and 25.0% versus 5.8% having a death event (Fig. EV3A,B). Lack of pathologic complete response (non-pCR) was not a significant predictor of recurrence (*p* = 0.250) or death (*p* = 0.070) in this cohort (Figs. EV3C,D and EV4A,B). Relating ctDNA to pCR, end-NAT ctDNA status trended but was not significantly associated to pCR (*p* = 0.063; Fig. 4C).

As an exploratory analysis, we investigated whether the dynamic reduction of ctDNA during NAT carried prognostic information. A variable ‘NAT ctDNA-response’ was defined as either complete clearance of ctDNA at end-NAT (i.e., being end-NAT ctDNA−) or partial ctDNA clearance, defined as a decrease in ctDNA TF by at least 50% from baseline to the end-NAT timepoint and with a decreasing TF trend from the prior timepoint to the end-NAT timepoint. Indeed, this NAT ctDNA-response variable was a significant predictor of good outcome with improved HRs for no events when compared to single timepoint end-NAT ctDNA status (HR 9.4, 95% CI 3.8 to 23.7 for BCFi and HR 11.9, 95% CI 3.9 to 35.9 for OS; *p* = 0.004 and *p* < 0.00001, respectively; Fig. 4D,E), with 10.4% experiencing recurrence (versus 56.3% for the NAT ctDNA-non-response group; *p* < 0.0001) and 5.7% having a death event (versus 43.8%; *p* < 0.00001) (Fig. EV3E,F). NAT ctDNA-response status trended but was not significantly associated to pCR (Fig. 4F). We also tested whether the combination of pre-surgical ctDNA and pCR carried additional prognostic information. Indeed, cases which were both end-NAT ctDNA+ and non-pCR had higher hazard ratios for BCFi (HR 4.0, 95% CI 1.7 to 9.5; *p* = 0.0009) and OS (HR 6.4, 95% CI 2.1 to 19.4; *p* = 0.0002) (Fig. EV4C,D). Moreover, cases which were both NAT ctDNA-non-responders and were non-pCR had marginally higher hazard ratios for BCFi (HR 10.4, 95% CI 4.2 to 26.2; *p* < 0.0001) and OS (HR 12.8, 95% CI 4.2 to 38.5; *p* < 0.0001) (Fig. EV4E,F) as compared to NAT ctDNA-response alone (Fig. 4D,E).

Within the BC clinical subtypes, the end-NAT ctDNA variable had inferior prognostic utility compared to NAT ctDNA-response (Appendix Figs. S2–S4). Interestingly, the NAT ctDNA-response variable was most prognostic for BCFi and OS within TNBC (HR 45.2, 95% CI 5.3 to 386.3; *p* < 0.00001; and HR 33.8, 95% CI 3.9 to 296.1; *p* < 0.00001, respectively), but showed similar trends and was also significantly associated to BCFi in the HR+ /HER2− subgroup (HR 7.3, 95% CI 1.0 to 51.8; *p* = 0.020), and to OS in the HER2+ subgroup (HR 10.8, 95% CI 1.0 to 122.7; *p* = 0.017) (Appendix Figs. S2–S4).

### Post-operative detection and ctDNA monitoring for molecular residual disease

Blood collection timepoints used for ctDNA monitoring from diagnosis through adjuvant treatment are represented in Fig. 3A and complete ctDNA longitudinal TF measurement results for each patient is provided in Appendix Fig. S1.

**Figure 5:**
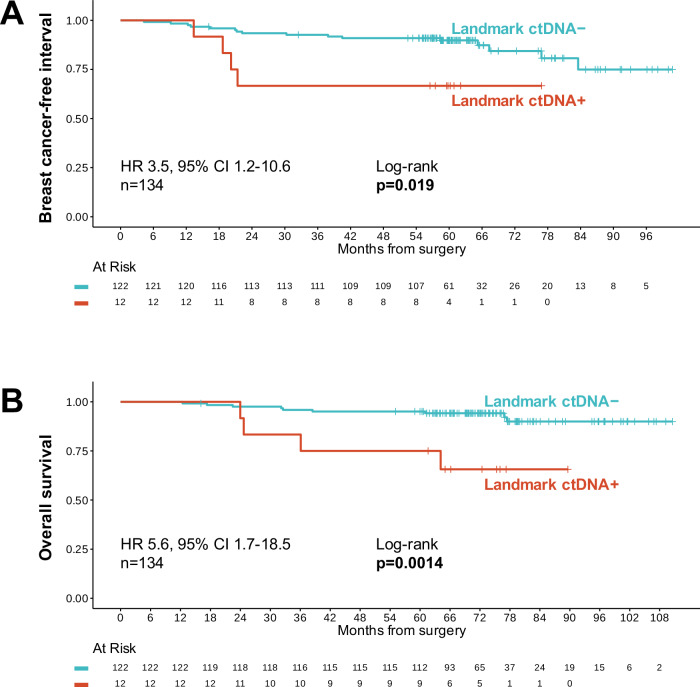
Detection of ctDNA at landmark timepoint. Kaplan-Meier plots illustrating that the immediate post-surgery landmark timepoint ctDNA status is a significant predictor of breast cancer-free interval (**A**) and overall survival (**B**). *P*-values calculated using the log-rank test.

**Figure 6:**
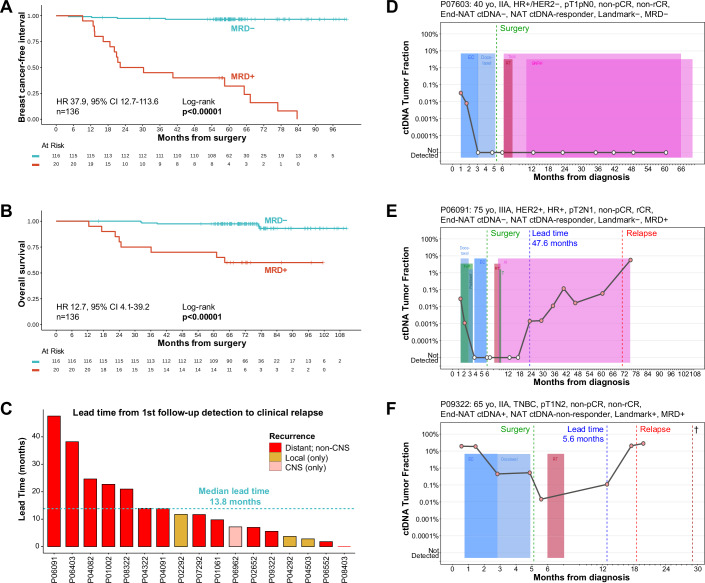
Detection of ctDNA at follow-up timepoints. Kaplan-Meier plots illustrating that the molecular residual disease (MRD) status in follow-up timepoints is a significant predictor of breast cancer-free interval (**A**) and overall survival (**B**). *P*-values calculated using the log-rank test. (**C**) Bar graph of all patients with a recurrence, indicating the recurrence type and illustrating the superior 13.8-month median lead-time gained by ctDNA analysis as compared to the clinical detection of recurrence. CNS = central nervous system. (**D**) Representative patient longitudinal plot illustrating the pattern of ctDNA clearance during neoadjuvant therapy (NAT) and no evidence of ctDNA during adjuvant follow-up. Patient ID is provided followed by age (in 5-year bins; yo = years old), clinical stage, subtype, pathological stage, pathological response (pCR) and radiological response (rCR), and on the next line, the ctDNA statuses are summarized. Timing of therapies are provided by colored backgrounds. AI = aromatase inhibitor; EC = epirubicin + cyclophosphamide; T = trastuzumab; TAM = tamoxifen; RT = radiotherapy. (**E**) Representative patient longitudinal plot illustrating the pattern of response to NAT and subsequent early detection of MRD+ with lead-time to clinical relapse. (**F**) Representative patient longitudinal plot illustrating the pattern of lack of response to NAT and detection of MRD+ with lead-time to clinical relapse and death (†).

The first post-operative blood plasma timepoint within 120 days of surgery, with median time from surgery of 14 days (range 5–103 days; *n* = 134), is defined as the ‘landmark’ timepoint and all subsequent post-surgery plasma timepoints are defined as ‘follow-up’ timepoints for MRD determination. Positive detection of ctDNA at landmark (landmark+) was significantly associated to BCFi (HR 3.5, 95% CI 1.2 to 10.6, *p* = 0.019) and OS (HR 5.6, 95% CI 1.7 to 18.5; *p* = 0.0014) (Figs. 5A,B and EV3G,H). Moreover, post-operative follow-up MRD positivity was associated with poor BCFi (HR 37.9, 95% CI 12.7 to 113.6; *p* < 0.00001) and OS (HR 12.7, 95% CI 4.1 to 39.2; *p* < 0.00001) (Fig. 6A,B). Generally, similar prognostic performance and trends were observed within the breast cancer subtypes for most ctDNA biomarker variables (Appendix Figs. S2–S4).

For the 15 patients who experienced distant clinical recurrence as of clinical database lock, ctDNA was detected at one or more of the 69 post-operative follow-up timepoints in 13/15 patients (sensitivity of 86.7%; 13/14 = 92.9% if only including the metastases that were pathologically-confirmed), with lead-times up to 47.6 months (median 13.8 months, range 0 to 47.6 months) (see Methods; Fig. 6C and Appendix Fig. S1). Notably, for the patients who presented with local-only or central nervous system (CNS)-only recurrences, 3 of 4 local-only relapses and 1 of 2 CNS-only relapses were detected (Fig. 6C). In the 115 patients without presentation of clinical distant recurrence as of last date of follow-up, ctDNA was undetectable in the follow-up plasma samples for 112 patients (97.4%).

### ctDNA and clinical course

Dynamic changes in ctDNA levels during the clinical course were observed across the serial plasma samples (Appendix Fig. S1). Three representative examples of ctDNA patterns and their relationship to the clinical course are highlighted (Fig. 6D–F). The majority of patients exhibited rapid and complete clearance of ctDNA during NAT, as exemplified by patient P07603 diagnosed with a clinical stage IIA, HR+/HER2−breast cancer at 40 years of age, and treated with neoadjuvant epirubicin plus cyclophosphamide followed by neoadjuvant docetaxel (Fig. 6D). At pathological examination of the surgical specimen, the tumor was ypT1ypN0, non-pCR, and by radiographic response measures, was non-radiological complete response (non-rCR). The patient was treated post-operatively with radiotherapy and adjuvant endocrine therapy for 5 years. By ctDNA, in contrast to pathological and radiological response evaluations, this patient was end-NAT ctDNA−, a clear end-NAT ctDNA-responder, was landmark−, and MRD− at all follow-up timepoints and remains recurrence free.

Patient P06091 is a classic case of a patient whose disease recurrence is detected by ctDNA monitoring (Fig. 6E). This 75-year-old patient was diagnosed with stage IIIA, HER2+, HR+ disease, treated with combination neoadjuvant chemotherapies as well as trastuzumab and pertuzumab, and was pathological ypT2ypN1, non-pCR, but showed rCR, and following surgical resection received adjuvant radiotherapy, a short course of trastuzumab (discontinued due to cardiotoxicity), and an aromatase inhibitor (AI) for 5 years. By ctDNA, she was end-NAT ctDNA−, a NAT ctDNA-responder, landmark−, but MRD+ at 18 months post-surgery (24 months after diagnosis). MRD positivity provided an almost 4-year lead-time to clinical relapse and showed a consistent rising pattern of ctDNA levels despite continuous AI treatment during this occult metastatic period.

The third example represents the group of patients who did not respond to NAT and had a particularly poor prognosis. Patient P09322, at the age of 65 was diagnosed with stage IIA, TNBC, and treated with sequential chemotherapy pre-operatively (Fig. 6F). The disease was non-pCR, non-rCR, and ypT1ypN2 at surgery, and by ctDNA was revealed to be end-NAT ctDNA+ with an increase from mid-NAT to end-NAT and thus classified a NAT ctDNA-non-responder, and was landmark+ and MRD+ at approximately 7 months post-surgery with a lead-time of 5.6 months to eventual clinical recurrence which occurred at 13 months post-surgery; approximately 10 months subsequent to disease relapse, the patient died from her disease. This patient was treated with approximately 2 years of zoledronic acid, begun pre-operatively and stopped at the time of clinical relapse.

We note that for 8/115 patients without clinical presentation of recurrence to date, ctDNA was detected at the landmark timepoint which subsequently cleared in all follow-up timepoints (6/8; patients P04222, P04862, P07862, P01962, P03503, and P04803; all ER+, with P03503 also HER2+) or within one to two follow-up timepoints (2/8; P02182 and P09503; both TNBC). To note, all of these patients were treated with adjuvant zoledronic acid, with the exception of P03503 who received adjuvant trastuzumab, and P02182 who did not receive any adjuvant medical treatment.

### ctDNA quantity as a prognostic factor

As an exploratory analysis, we investigated further whether the quantity of ctDNA—as measured by each patient-specific multiplex dPCR SV assay—carried quantitative prognostic information. Baseline ctDNA quantity was significantly associated to OS (per each doubling of ctDNA TF: HR 1.4, 95% CI 1.1 to 1.6; *p* = 0.0012) but did not reach significance for BCFi (HR 1.1, 95% CI 1.0 to 1.2; *p* = 0.079). The amount of ctDNA at end-NAT was also a quantitative prognostic factor, with HR of 1.2 for every doubling of ctDNA TF for BCFi (95% CI 1.1 to 1.2, *p* < 0.0001) and HR of 1.2 per doubling for OS (95% CI 1.1 to 1.3, *p* < 0.0001). Similarly, the ctDNA TF quantity at landmark was also a quantitative prognostic factor for BCFi (HR 1.2, 95% CI 1.1 to 1.3; *p* = 0.0009) and OS (HR 1.2, 95% CI 1.1 to 1.3; *p* = 0.0001).

## Discussion

In this prospective clinical validation study of patients with EBC treated with neoadjuvant chemotherapy and monitored for a median of 6.0 years of post-surgical follow-up, we demonstrate that ctDNA monitoring using an innovative tumor-informed multiplex dPCR method for SV detection provides clinically relevant prognostic information on relapse and survival. During the neoadjuvant period, we found that ctDNA persistence after NAT was associated with worse BCFi and OS, with ctDNA-response (complete or partial clearance) during NAT being an even stronger prognostic factor. Furthermore, ctDNA positivity at the landmark timepoint after surgery is a significant predictor of worse BCFi and OS. Moreover, post-operative MRD detection precedes clinical relapse by up to 4 years, with a median lead-time of 13.8 months. The overall sensitivity for detecting distant recurrences was 86.7%, increasing to 92.9% when considering only pathologically-confirmed recurrences. When local-only and CNS-only relapses were included, sensitivity was 81.0%.

SVs are well-recognized features of cancer genomes, yet their potential as truncal tumor-specific biomarkers has been largely underappreciated, except for a subset of well-known and targetable driver translocations and fusion genes in a variety of cancer types such as leukemias and lung adenocarcinoma. Extensive genomic analyses have shown that SVs are highly prevalent across diverse cancer types, supporting the broad applicability of an SV-directed ctDNA assay across malignancies (Stephens et al, 2009; Banerji et al, 2012; Nik-Zainal et al, 2012; Alkner et al, 2015; Olsson et al, 2015; Tang et al, 2015; Li et al, 2020; Elliott et al, 2025; Glueck et al, 2025). To leverage the potential of SVs as tumor-specific biomarkers, our automated and scalable method generates a personalized SV-based tumor fingerprint, which is then used to generate a validated multiplex dPCR assay. Once established, the personalized fingerprint SV-directed dPCR assay enables rapid cfDNA analysis within hours to days of blood collection, and longitudinal monitoring over years to decades using the same personalized assay.

Previous studies have demonstrated that the vast majority of SVs present in the primary tumor persist in metastatic disease, even when separated by many years (Alkner et al, 2015; Tang et al, 2015). Given that Pathlight’s personalized multiplex assay tracks up to 16 SVs identified in the primary tumor, it is highly probable that many and at least one tracked SV is retained in an eventual metastasis.

In line with other studies (Magbanua et al, 2021; Magbanua et al, 2023; Elliott et al, 2025; Garcia-Murillas et al, 2025), our results suggest that ctDNA monitoring can predict impending clinical metastasis by months to years in advance and may serve as a quantitative predictive factor in both the neoadjuvant and adjuvant settings for patients with EBC. Interestingly, 8 patients in our cohort were landmark+ but then cleared ctDNA and have not recurred clinically as of last follow-up. Several biological explanations are possible. One is that the landmark positivity reflects transient perioperative ctDNA release that persists after surgery in the absence of true microscopic residual disease. This is unlikely given that landmark blood collection occurred a median of 14 days post-surgery (minimum 5 days) and cfDNA has a reported half-life of minutes to hours (Lo et al, 1998). A second possibility is cure of microscopic residual disease in the adjuvant setting, consistent with findings from other cancer types such as colorectal (Kotani et al, 2023). This may be mediated by therapies received and/or by immune system activation; to note, 7/8 of these patients received adjuvant treatment. Lastly, residual cancer cells may have entered a dormant state and thus shed little to no cfDNA, with potential for later reactivation and relapse, a well-known phenomenon in breast cancer that can manifest more than 10–15+ years after primary surgery (Giannakeas and Narod, 2019). These possibilities may be answerable in the future as additional clinical follow-up time accrues.

The future of breast cancer treatment lies in personalized therapies and precision care. To realize this vision, accurate, non-invasive methods for monitoring breast cancer progression and response to treatment are essential. Clinical trials that tailor secondary adjuvant therapy guided by ctDNA monitoring could help determine whether this approach increases long-term breast cancer survival. Our findings indicate that an SV-based approach may possess the necessary performance characteristics to assess whether detection of occult metastasis through ctDNA monitoring can occur at the earliest possible moment, allowing for interventions that could lead to more durable treatments and improved patient outcomes. Importantly, as a limitation, ctDNA analyses should be interpreted in the context of available treatment options, which have been rapidly changing in EBC during and after the enrolment period of NeoCircle. As part of an “active surveillance” strategy, and in light of the multiple new treatment options, molecular monitoring by liquid biopsies could also reduce overtreatment in low-risk breast cancer patients who may be cured with locoregional therapies alone, or aid in selection of treatment and escalation or de-escalation of treatment intensity or duration to maximize survival outcomes while reducing unwanted side-effects. In conclusion, non-invasive ctDNA monitoring shows significant promise for providing biologically and clinically relevant information, with Pathlight exemplifying its potential for integration into routine clinical management for patients with early breast cancer.

## Methods

**Table:** Reagents and tools table

Reagent/Resource	Reference or Source	Identifier or Catalog Number
**Oligonucleotides and other sequence-based reagents**		
Hydrolysis probes and patient-specific primers for dPCR	Integrated DNA Technologies	n/a – patient specific
**Chemicals, Enzymes and other reagents**		
Pathlight dPCR assays	This study	n/a – patient specific
Tumor-derived positive control	This study	n/a – patient specific
Matched normal-derived negative control	This study	n/a – patient specific
Healthy donor-derived negative control	Biochain	n/a – donor specific
**Software**		
Pathlight^TM^ patient-specific SV fingerprint design	SAGA Dx Inc.	n/a – patient specific
R v4.4.1, *tidyverse* v2.0.0, *ggplot2* v3.5.1, *ggpubr* v0.6.0, *survminer* v0.4.9, *survival* v3.7.0	The R Project for Statistical Computing	https://www.r-project.org/
**Other**		
Cell-Free DNA BCT CE	Streck	218997
Mag-Bind FFPE DNA/RNA kit	Omega Biotek	M6955
AllPrep DNA RNA Mini kit	Qiagen	80204
Mag-Bind Blood & Tissue DNA HDQ kit	Omega Biotek	M6399
QIAamp DNA Blood Mini kit	Qiagen	51106
QIAamp Circulating Nucleic Acid kit	Qiagen	55114
QIAamp MinElute ccfDNA kit	Qiagen	55284
Qubit 1X dsDNA High Sensitivity (HS) Assay kit	Thermo Fisher Scientific	Q33265
Qubit Flex Fluorometer instrument	Thermo Fisher Scientific	Q33327
E220 or ML230 Focused-ultrasonicator instrument	Covaris	500239, 500656
QIAseq UDI Y-Adapter sequences	Qiagen	180312, 180314, 180316, 180318
Pippin HT instrument	Sage Science	HTP0001
NextSeq 550, NovaSeq X, NovaSeq 6000	Illumina	https://www.illumina.com/systems/sequencing-platforms.html
QIAcuity Nanoplate 8.5k 96-well	Qiagen	250021
QIAcuity Eight Digital PCR Platform System	Qiagen	911052

### Ethics statement

The study was approved by the Regional Ethical Review Board in Lund (registration numbers 2009/658, 2009/659, 2010/383, 2012/58, 2012/379, 2013/12, 2013/459, 2014/521, 2014/681, 2015/277, 2016/541, 2016/742, 2016/944, 2018/267), Swedish Ethical Review Authority (registration numbers 2019-01252, 2019-00700, 2024-02040-02), and the county’s governmental biobank authority and the Swedish Data Inspection group (diary number 364-2010). Trained health professionals provided written and oral information, and all patients signed written informed consent in accordance with the WMA Declaration of Helsinki and the U.S. Department of Health and Human Services Belmont Report.

### Patients

Patients were prospectively enrolled in NeoCircle, a substudy within the SCAN-B clinical study (ClinicalTrials.gov identifier NCT02306096) (Saal et al, 2015). Eligible patients for NeoCircle were diagnosed at Skåne University Hospital in Lund or Malmö with early or locally recurrent breast cancer and were considered suitable for pre-operative chemotherapy (+/−HER2-directed therapy) with curative intent. Tumor biopsies were collected through ultrasound-guided core needle biopsy from the primary tumor.

All clinical and pathological information were retrieved from the patient records. Estrogen receptor (ER) and progesterone receptor (PgR) status were measured by immunohistochemistry (IHC) and evaluated according to Swedish guidelines using a 10% cut-off, and HER2 was evaluated using standard HercepTest and in situ hybridization criteria. If ER, PgR, or HER2 status was not available for the diagnostic biopsy, then the result from the surgical biopsy was used. Clinical subtypes were defined as follows: HR+/HER2− = positive for ER or PgR and HER2 non-amplified; HER2+ = HER2 amplified; TNBC = negative for ER, PgR, and HER2.

For ctDNA analysis, blood samples were collected in Streck Cell-Free DNA blood collection tubes (Streck BCT) (Streck, Cat. Nos. 218996, 218997, 230244) at baseline during diagnostic mammography, after the first and third three-weekly chemotherapy cycles, before definitive surgery, two weeks post-operatively, and at 6-, 12-, 18-, 24-, 30-, 36-, 42-, and 54-months after surgery. A subset of patients also provided blood samples immediately before and after mammographic breast compression at baseline, as well as during and immediately after definitive surgery (Fornvik et al, 2019). Sampling generally occurred within +/−1 month of schedule, however, some timepoints may have had greater deviations or have been missed, e.g., due to the COVID-19 pandemic.

All patients received treatment according to Swedish national guidelines. For NAT, most patients underwent sequential chemotherapy including an anthracycline-based regimen (epirubicin and cyclophosphamide q3w x 3) and a taxane-based therapy, either docetaxel (q3w x 3) or paclitaxel (q1w x 9-12) (Table 1, Appendix Table S1). HER2-directed antibodies were administered as appropriate. Some patients received modified regimens, including alternative sequences or single-agent chemotherapy, such as in cases of poor tolerability, hypersensitivity, or practical considerations. Post-operative treatment included radiotherapy, endocrine therapy, HER2-directed therapy, and zoledronic acid, according to guidelines, and, in selected cases, capecitabine for patients with poor response at conventional response evaluation.

All patients received routine clinical follow-up according to Swedish national guidelines. Patients who did not receive adjuvant chemotherapy had follow-up clinical visits and mammography at years 1, 2, and 3 after primary surgery, followed by mammographic surveillance within the national screening program. Mammographic surveillance for breast cancer patients is extended through 80 years of age, and performed annually for patients treated with breast-conserving surgery, or at 18–24 month intervals for patients that underwent unilateral mastectomy. For hereditary high-risk breast cancer, MRI may be utilized in high-risk surveillance programs (annually, often alternating with mammography). MRI may be considered if mammographic sensitivity is limited and clinical suspicion exists, such as in younger women with extremely dense breasts. MRI may also be performed when there is equivocal mammography/ultrasound, there is clinical suspicion of recurrence but negative conventional imaging, or there are post-surgical or radiation changes that obscure evaluation. Patients receiving adjuvant chemotherapy were clinically evaluated after completing chemotherapy, followed by annual clinical visits up to year 5, and subsequent mammographic surveillance as above. If any of the follow-up clinical visits or assessments indicated symptoms or signs of metastatic disease, or clinical suspicion but obscured or equivocal conventional imaging, appropriate advanced imaging and confirmatory work-up was performed. All administered cancer therapies are shown in Appendix Fig. S1.

A pathologic complete response (pCR) was defined as the complete absence of residual invasive cancer cells in the resected breast specimen and all sampled lymph nodes, as assessed by the clinically assigned pathologist. A radiological complete response (rCR) was defined as the absence of any visible residual tumor on post-NAT mammography, as assessed by the clinically assigned radiologist, irrespective of whether the lesion had been measurable or only visually identifiable (but not measurable) before treatment. For patients whose tumors were not detectable on mammography at baseline, rCR was instead evaluated using ultrasound, applying the same criterion—no radiological evidence of residual tumor following NAT. Local recurrence is defined as cancer relapse to the ipsilateral breast, surgical scar, or chest wall; distant recurrences are defined as cancer detected in any organ or tissue distant from the original tumor site; only 1 patient had a solitary recurrence to the ipsilateral axilla (regional relapse) and is counted within the distant recurrence group.

All analyses were conducted in a blinded fashion, and only blood samples that passed minimum quality control criteria were analyzed. Study results remained blinded to the clinical team throughout the study.

### Tumor tissue whole-genome sequencing

Tumor tissue samples, either formalin-fixed paraffin-embedded (FFPE) or fresh frozen (FF), were processed for DNA extraction and whole-genome sequencing (WGS) library generation. Separate workflows were employed for FFPE and FF tissue to ensure optimal DNA quality and yield for downstream analysis (Fig. 1A).

### FFPE workflow

For 90 patients (after exclusions; Fig. 1A), FFPE tumor tissue from diagnostic biopsies was used to generate tumor-informed SV fingerprints. Pathology review targeted ≥20% tumor cellularity (percentage of total cells present in the specimen). Up to ten 4-micron sections were obtained from FFPE biopsies, along with a H&E slide, for genomic DNA extraction in accordance with institutional policies to conserve diagnostic tissue.

FFPE tumor DNA extraction was performed using the Mag-Bind FFPE DNA/RNA 96 Kit (Omega Bio-Tek, Cat. No. M6955) on a Hamilton Microlab STAR liquid handler. Sequencing libraries were prepared from 50 ng of the FFPE DNA as previously described (Elliott et al, 2025). QIAseq UDI Y-Adapter sequences (Qiagen, Cat. Nos. 180312, 180314, 180316, 180318) were incorporated during library preparation. Libraries were equimolarly pooled, and WGS was performed on an Illumina NovaSeq X platform targeting ~15x depth (SAGA Diagnostics, Morrisville, North Carolina, USA).

### FF workflow

For 46 patients (after exclusions; Fig. 1A), DNA was extracted from FF tumor tissue using the AllPrep DNA RNA Mini Kit (Qiagen, Cat. No. 80204), following the manufacturer’s instructions, and used to generate tumor-informed SV fingerprints. A total of 500 ng of genomic DNA from FF tumor tissue was mechanically fragmented using a Covaris E220 or ML230 instrument.

Sequencing libraries were prepared with QIAseq UDI Y-Adapters (Qiagen, Cat. Nos. 180312, 180314, 180316, 180318). Libraries underwent size selection on a PippinHT instrument (Sage Science). After size selection, equimolar pooling of the libraries was performed, followed by WGS on an Illumina NextSeq 550 or NovaSeq 6000 (Novogene, UK) sequencing platform at ~15x depth.

### Serial blood sample collection and cfDNA extraction

Serial blood samples (one Streck BCT tube per timepoint) were collected and processed by double centrifugation, and plasma and buffy coat fractions were aliquoted and stored at –80 °C until cfDNA extraction (Chen et al, 2018).

Cell-free DNA extraction was performed using the QIAamp Circulating Nucleic Acid Kit (Qiagen Cat. No. 55114) or the QIAamp MinElute ccfDNA Kit (Qiagen Cat. No. 55284), according to the manufacturer’s protocols. The cfDNA mass concentrations were quantified using the Qubit 1x dsDNA High Sensitivity Assay Kit (Thermo Fisher Scientific, Cat. No. Q33265). Extracted cfDNA was stored at –80 °C until further analysis: ctDNA analysis was performed using the Pathlight dPCR workflow (Elliott et al, 2025) for FFPE cohort samples and the research dPCR analysis method for FF cohort samples (both SAGA Diagnostics), as described below.

### Patient-specific germline DNA preparation

For each patient, DNA was extracted from baseline buffy coat samples to serve as the source of germline genetic material. For the FFPE cohort, extraction was performed using the Mag-Bind Blood & Tissue DNA HDQ Kit (Omega Bio-Tek, Cat. No. M6399) on a Hamilton Microlab STAR liquid handler. For the FF cohort, the QIAamp DNA Blood Mini Kit (Qiagen, Cat. No. 51106) was used.

### Personalized SV fingerprint dPCR assay development and ctDNA monitoring

The FFPE cohort samples were analyzed with Pathlight (SAGA Dx Inc), a tumor-informed ctDNA assay designed to detect tumor-specific SVs using multiplex dPCR on plasma-derived cfDNA. The assay consists of two principal stages: fingerprint generation with orthogonal validation, and ctDNA analysis. In the fingerprint generation stage, SVs are identified from WGS of tumor tissue to create a unique, patient-specific fingerprint (Elliott et al, 2025) (Fig. 1B). In the ctDNA analysis stage, the validated tumor-specific SVs are targeted in plasma cfDNA using multiplex dPCR. For each patient, up to 16 SVs are interrogated in the FFPE cohort using the Pathlight assay, while up to 8 SVs are analyzed in the FF cohort using the SAGA FF method. dPCR signal detection is based on relative fluorescence unit (RFU) measurements, and results are interpreted using a proprietary thresholding algorithm to determine ctDNA status. Further details were described in Elliott et al (2025). Generally, whether the FF or FFPE workflow was utilized, similar patterns were seen for the ctDNA analyses using the resultant multiplex dPCR assays (Fig. 2; Appendix Fig. S1).

### Statistical analyses

All statistical analyses were conducted using R v4.4.1, with data management carried out using the *tidyverse* package (v2.0.0). Fisher’s exact test for count data was applied to assess statistical significance in contingency tables, and the Mann–Whitney U test was applied to assess the difference between two observed continuous values. All reported *p*-values and confidence intervals are two-sided. A *p*-value of ≤0.05 was considered statistically significant. Exact *p*-values not presented in the text or figures are provided in Appendix Table S2. Data visualization was performed using the *ggplot2* (v3.5.1) and *ggpubr* (v0.6.0) R packages.

Survival analyses were conducted using the Kaplan-Meier method, implemented in the *survminer* R package (v0.4.9), while Cox regression analysis was performed with the *survival* R package (v3.7.0). Breast cancer-free interval (BCFi) and overall survival (OS), as defined by DATECAN guidelines, were used as endpoints. As pre-specified in the analysis plan, patients with local-only or CNS-only recurrences were excluded from lead-time and sensitivity calculations.

ctDNA TF levels were log2-transformed (Olsson et al, 2015). The transformation applied the formula log₂(tf + 1e^−8^), where tf represents the ctDNA tumor fraction, and the constant 1e^−8^ was added to prevent undefined values.

## Supplementary information

Appendix

Peer Review File

Figure Source data

Expanded View Figures

## Data Availability

Due to patient privacy, the whole-genome sequencing data, which may contain personally identifiable genetic variation and disease-associated alleles, are not publicly available. All other data supporting the study’s findings are provided in the article and its supplementary information files. No other data requires deposition in a public database. The source data of this paper are collected in the following database record: biostudies:S-SCDT-10_1038-S44321-026-00447-z.
