# Supplementary material for: NeoCircle: pre- and post-operative circulating tumor DNA dynamics predicts survival in neoadjuvant-treated early breast cancer
Source: EMBO Mol Med. 2026 May 26;18(7):2617–34. doi: 10.1038/s44321-026-00447-z (PMC13365804; doi:10.1038/s44321-026-00447-z)
Supplement: Supplementary file 1 — Appendix [file 44321_2026_447_MOESM1_ESM.pdf]

APPENDIX

NeoCircle: pre- and post-operative circulating tumor DNA dynamics predicts survival in neoadjuvant-treated early breast cancer

Anthony M. George, Yilun Chen, Sergii Gladchuk, Miguel Alcaide, Hina Dalal, Pei Meng, Christian Brueffer, Hani Saghir, Siker Kimbung, Kristina Aaltonen, Lucia Oton, Christopher Rushton, Sofia Birkeälv, Mats Jönsson, Sophia Zackrisson, Ida Skarping, Daniel Förnvik, Lina Zander, Gabriella Honeth, Samuel Woodhouse, Karen Howarth, Åke Borg, Anna Ehinger, Martin Malmberg, Lisa Rydén, Niklas Loman, and Lao H. Saal

Contents

Appendix Figures and Tables 2

- Appendix Figure S1: Longitudinal ctDNA monitoring plots for all patients 2
- Appendix Figure S2: Kaplan-Meier survival estimates for the ER+/HER2− subgroup 139
- Appendix Figure S3: Kaplan-Meier survival estimates for the HER2+ subgroup 140
- Appendix Figure S4: Kaplan-Meier survival estimates for the TNBC subgroup 141
- Appendix Table S1: Per-patient clinicopathological characteristics 142
- Appendix Table S2: Exact P-values 143

Appendix Figures and Tables

Appendix Figure S1: Longitudinal ctDNA monitoring plots for all patients.

One patient is plotted per page. The header provides the Patient ID followed by age (in 5-year bins), clinical stage, subtype, pathological stage, pathological response and radiological response, and on the next line the ctDNA statuses are summarized. Therapies given are indicated by colored bars. Date of surgery is indicated by vertical green dotted line, date of relapse by vertical red dotted line, date of death by vertical brown dotted line, and lead-time from 1st follow-up MRD detection to clinical detection of relapse by vertical blue dotted line. The following abbreviations are used for the treatments: AI = Aromatase Inhibitor; AI&GnRH = Aromatase Inhibitor + Gonadotropin-Releasing Hormone agonist; CEX = Cyclophosphamide + Epirubicin + Capecitabine; Dtx = Docetaxel; EC = Epirubicin + Cyclophosphamide; FEC = Fluorouracil + Epirubicin + Cyclophosphamide; GnRH = Gonadotropin-Releasing Hormone agonist; GnRH&Other = Gonadotropin-Releasing Hormone agonist + Other; Ptx = Paclitaxel; Ptz = Pertuzumab; PCb = Paclitaxel+Carboplatin; TAM = Tamoxifen; TAM&GnRH = Tamoxifen + Gonadotropin-Releasing Hormone agonist; T = Trastuzumab; TP = Trastuzumab + Pertuzumab; V = Vinorelbine; X = Capecitabine; ZA = Zoledronic acid.

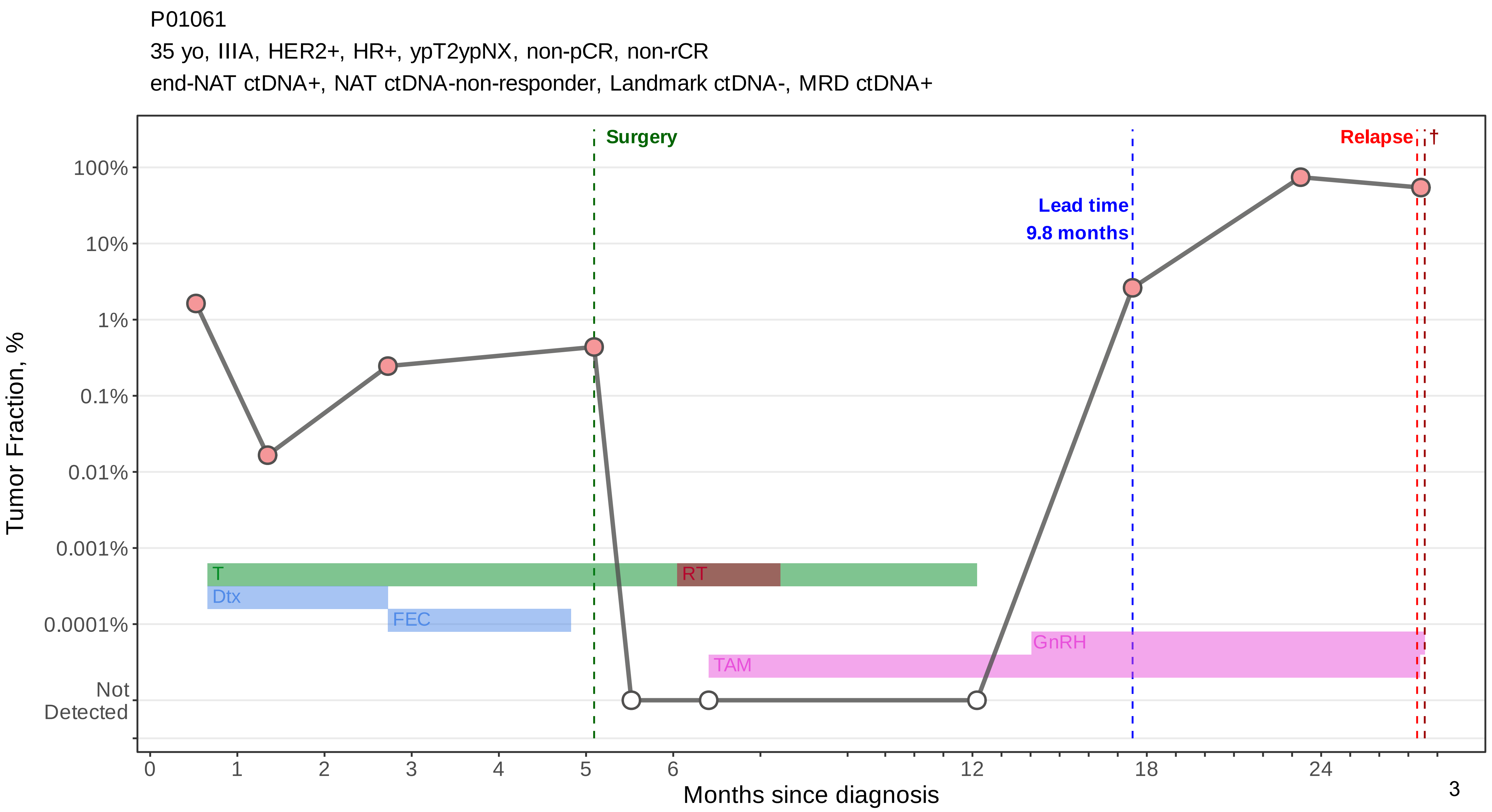
P01061
35 yo, IIIA, HER2+, HR+, ypT2ypNX, non-pCR, non-rCR
end-NAT ctDNA+, NAT ctDNA-non-responder, Landmark ctDNA-, MRD ctDNA+
Surgery
Relapse
†
Lead time
9.8 months
100%
10%
1%
0.1%
0.01%
0.001%
0.0001%
Not Detected
T
RT
Dtx
FEC
GnRH
TAM
0
1
2
3
4
5
6
12
18
24
Tumor Fraction, %
Months since diagnosis

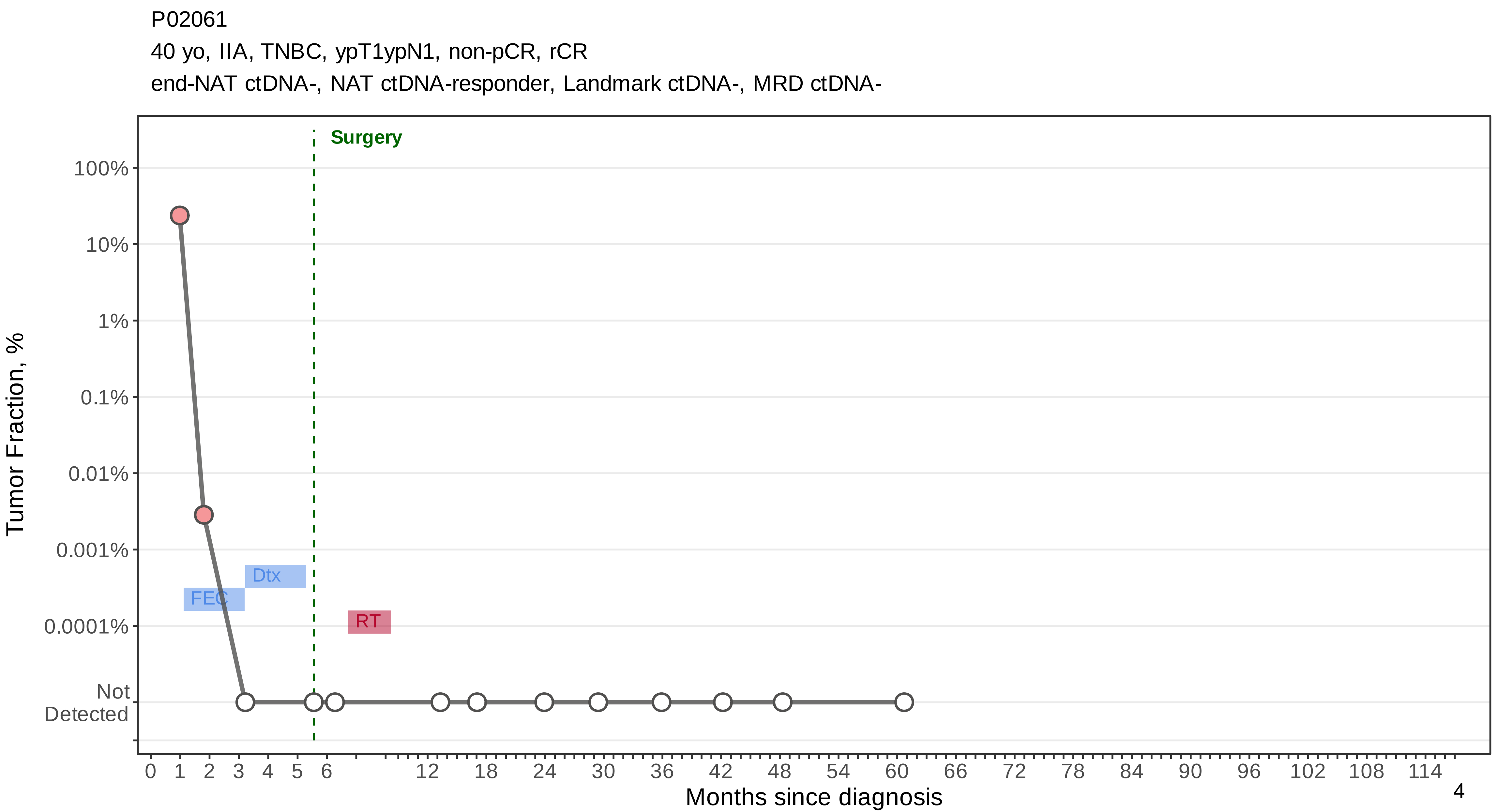

P02061
40 yo, IIA, TNBC, ypT1ypN1, non-pCR, rCR
end-NAT ctDNA-, NAT ctDNA-responder, Landmark ctDNA-, MRD ctDNA-
Surgery
100%
10%
1%
0.1%
0.01%
0.001%
0.0001%
Not Detected
Dtx
FEC
RT
Tumor Fraction, %
0 1 2 3 4 5 6 12 18 24 30 36 42 48 54 60 66 72 78 84 90 96 102 108 114
Months since diagnosis

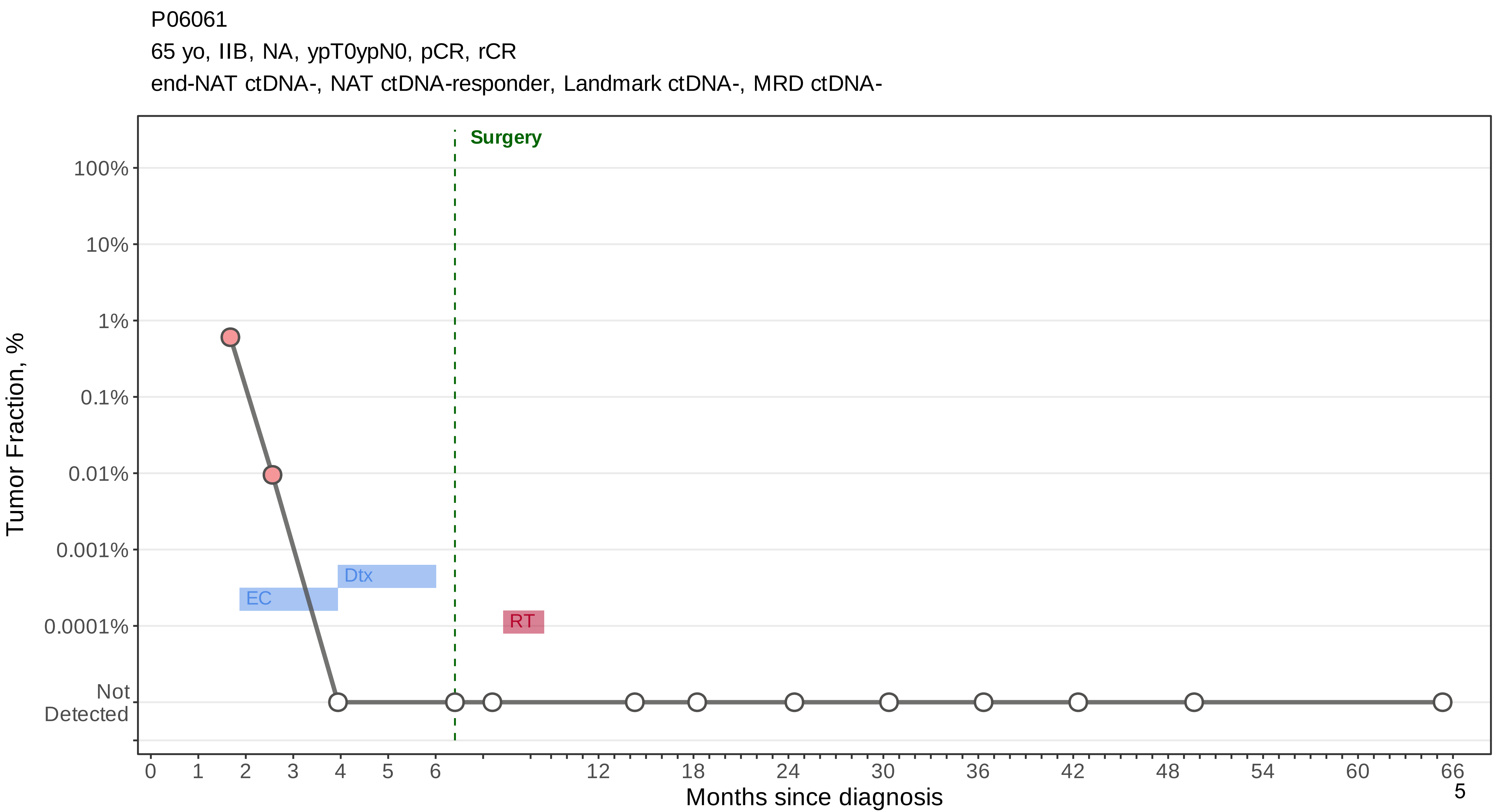

P06061
65 yo, IIB, NA, ypT0ypN0, pCR, rCR
end-NAT ctDNA-, NAT ctDNA-responder, Landmark ctDNA-, MRD ctDNA-
Surgery
100%
10%
1%
0.1%
0.01%
0.001%
0.0001%
Not Detected
Tumor Fraction, %
Dtx
EC
RT
0
1
2
3
4
5
6
12
18
24
30
36
42
48
54
60
66
Months since diagnosis

P07061
45 yo, IIIA, TNBC, ypT0ypN1, pCR, rCR
end-NAT ctDNA-, NAT ctDNA-responder, Landmark ctDNA-, MRD ctDNA-

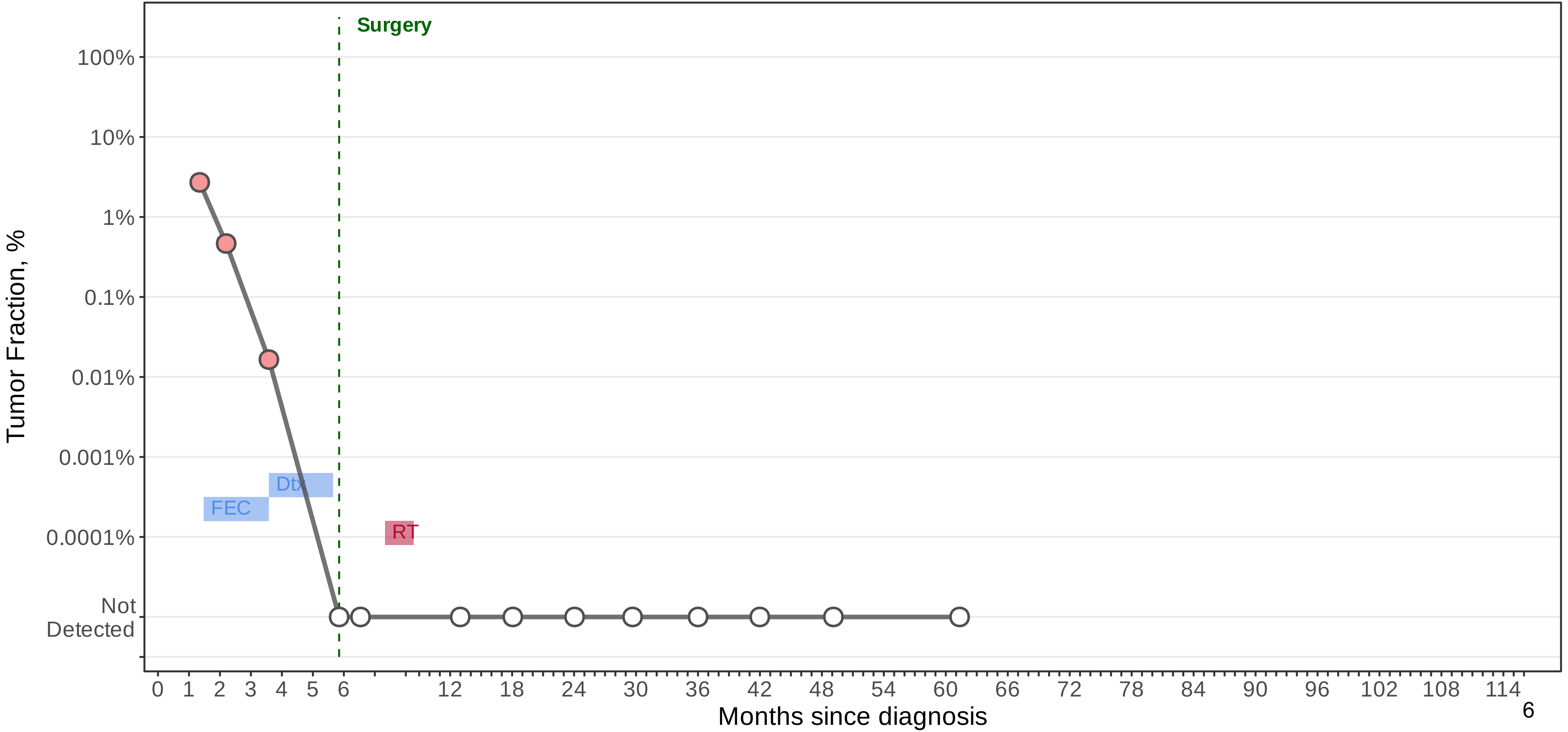

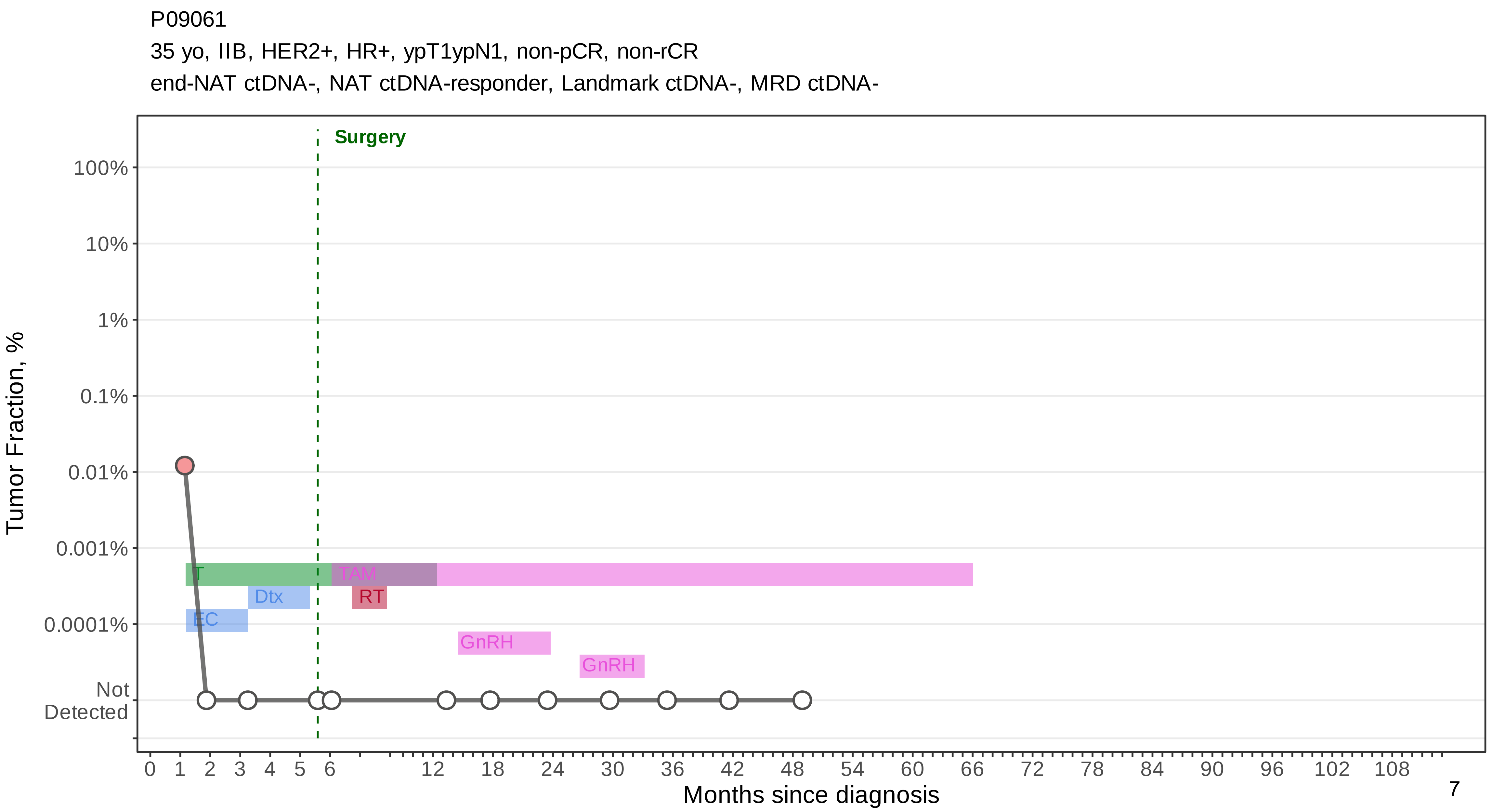

P09061
35 yo, IIB, HER2+, HR+, ypT1ypN1, non-pCR, non-rCR
end-NAT ctDNA-, NAT ctDNA-responder, Landmark ctDNA-, MRD ctDNA-
Surgery
100%
10%
1%
0.1%
0.01%
0.001%
0.0001%
Not Detected
Tumor Fraction, %
T
TAM
Dtx
RT
EC
GnRH
GnRH
0 1 2 3 4 5 6 12 18 24 30 36 42 48 54 60 66 72 78 84 90 96 102 108
Months since diagnosis

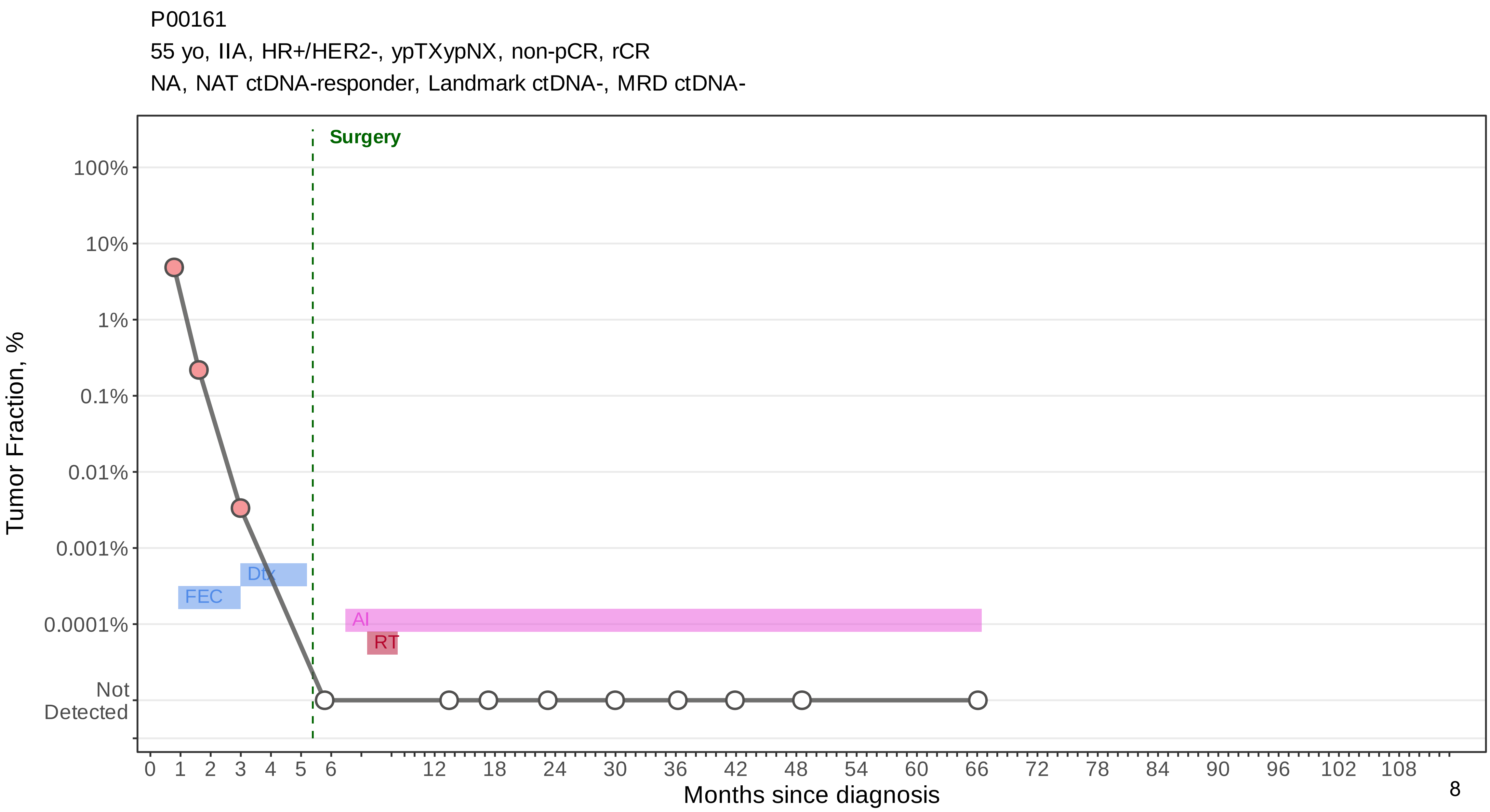

P00161
55 yo, IIA, HR+/HER2-, ypTXypNX, non-pCR, rCR
NA, NAT ctDNA-responder, Landmark ctDNA-, MRD ctDNA-
Surgery
Tumor Fraction, %
100%
10%
1%
0.1%
0.01%
0.001%
0.0001%
Not Detected
Dtx
FEC
AI
RT
0 1 2 3 4 5 6 12 18 24 30 36 42 48 54 60 66 72 78 84 90 96 102 108
Months since diagnosis

P03161
35 yo, IIA, HER2+, HR+, ypT1ypN0, non-pCR, rCR
end-NAT ctDNA-, NAT ctDNA-responder, Landmark ctDNA-, MRD ctDNA-

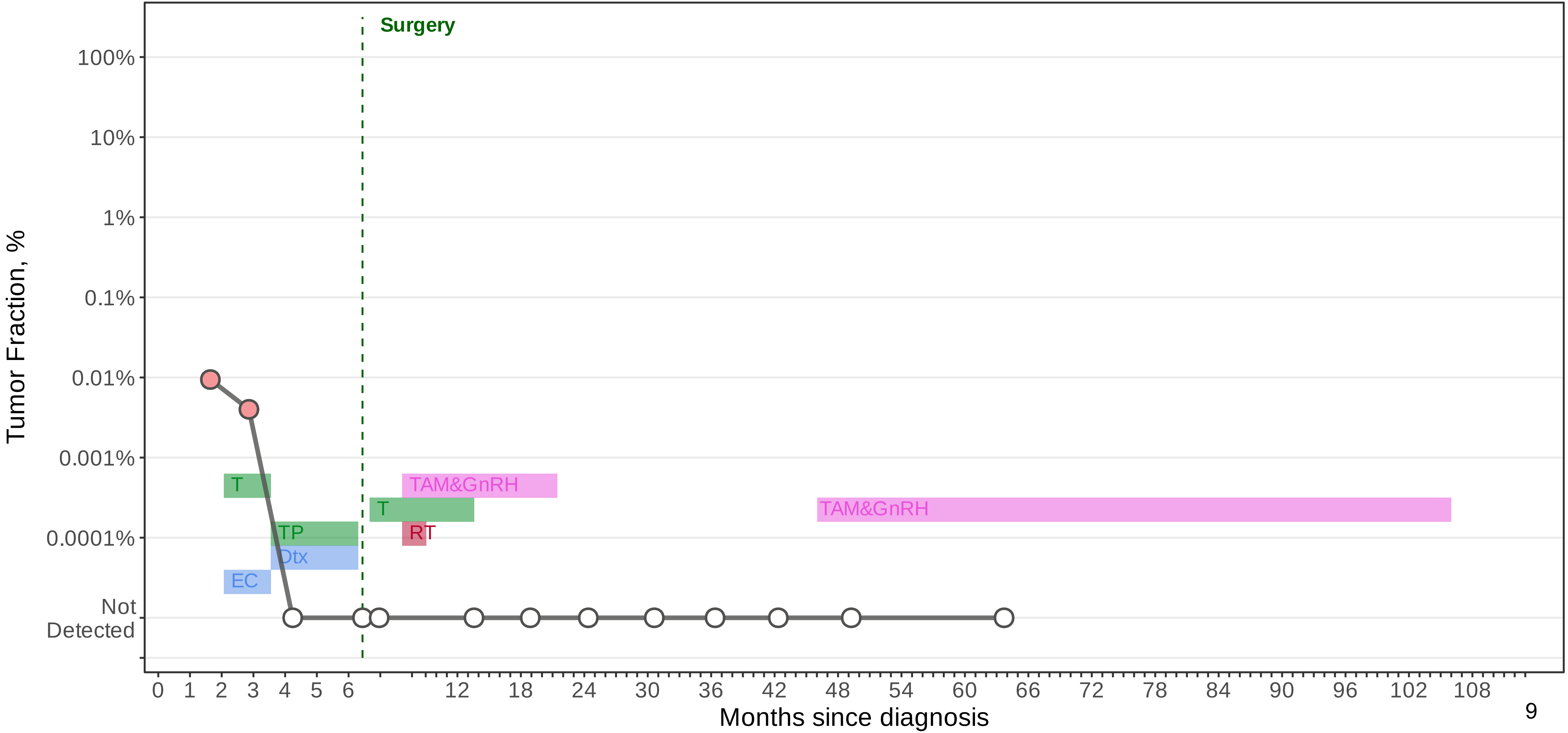

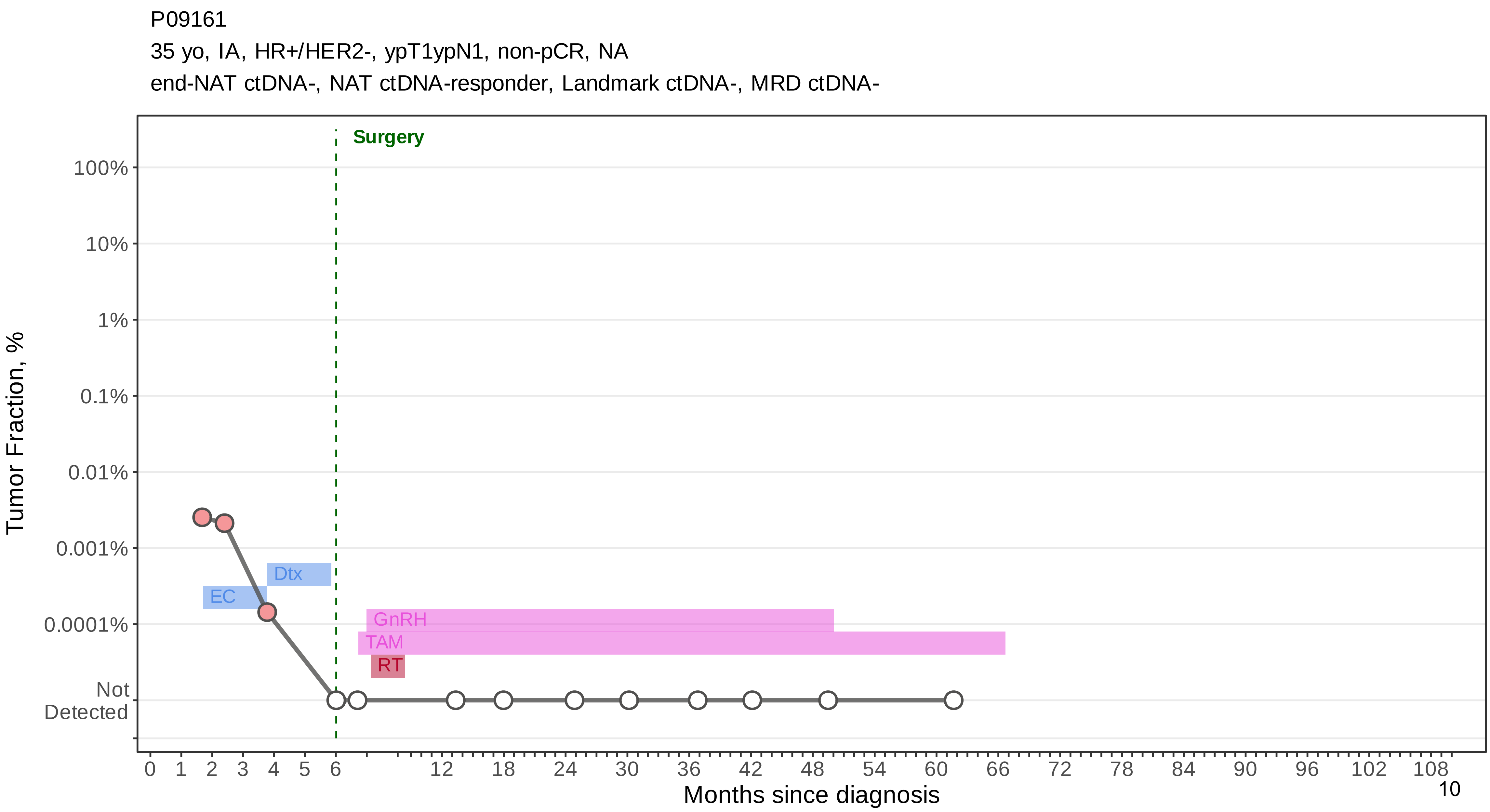
P09161
35 yo, IA, HR+/HER2-, ypT1ypN1, non-pCR, NA
end-NAT ctDNA-, NAT ctDNA-responder, Landmark ctDNA-, MRD ctDNA-
Surgery
Tumor Fraction, %
100%
10%
1%
0.1%
0.01%
0.001%
0.0001%
Not Detected
Dtx
EC
GnRH
TAM
RT
0 1 2 3 4 5 6 12 18 24 30 36 42 48 54 60 66 72 78 84 90 96 102 108
Months since diagnosis

P00261
40 yo, IIA, HR+/HER2-, ypT1ypN0, non-pCR, NA
end-NAT ctDNA-, NAT ctDNA-responder, Landmark ctDNA-, MRD ctDNA-

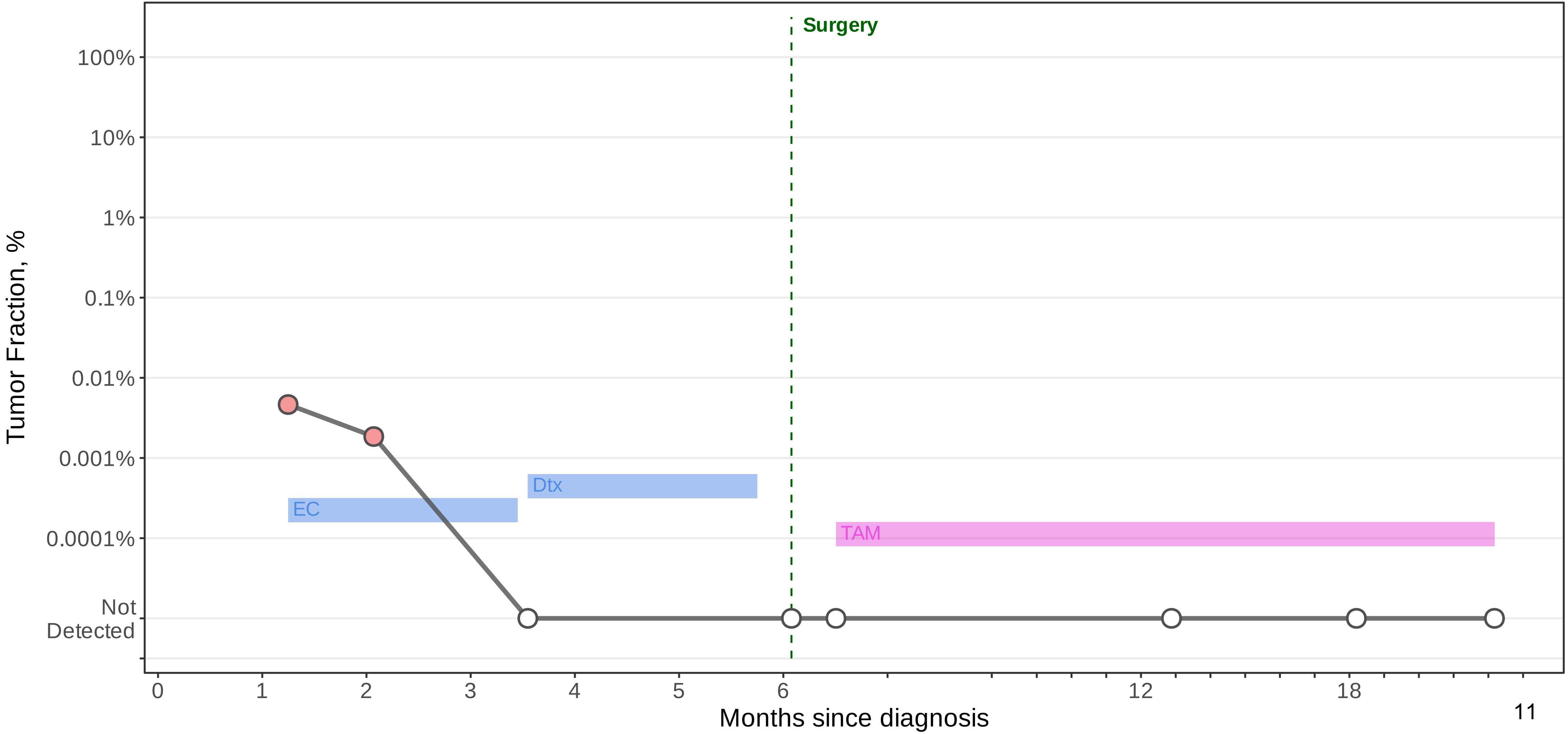

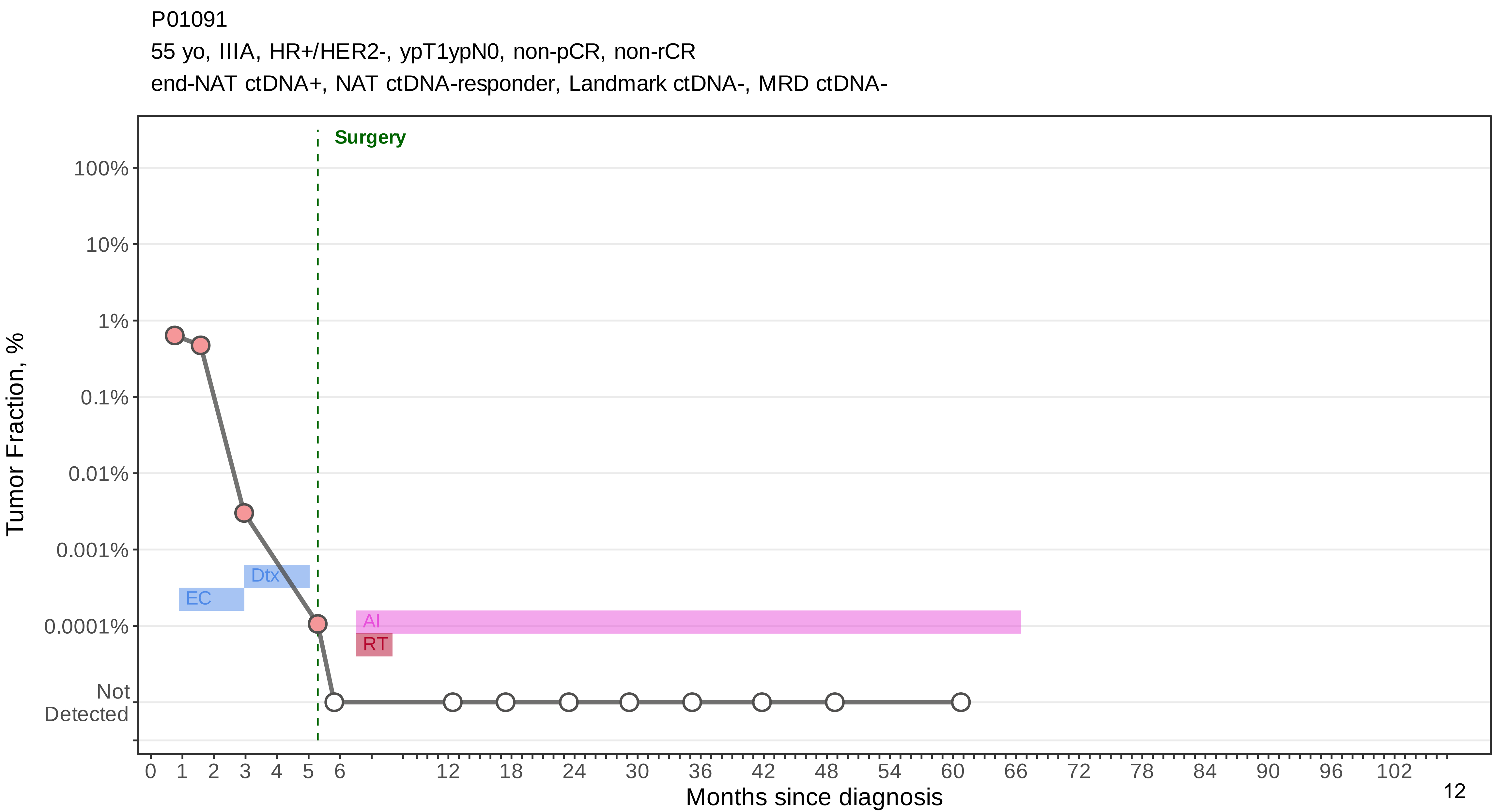

P01091
55 yo, IIIA, HR+/HER2-, ypT1ypN0, non-pCR, non-rCR
end-NAT ctDNA+, NAT ctDNA-responder, Landmark ctDNA-, MRD ctDNA-
Surgery
100%
10%
1%
0.1%
0.01%
0.001%
0.0001%
Not Detected
Tumor Fraction, %
EC
Dtx
AI
RT
0 1 2 3 4 5 6 12 18 24 30 36 42 48 54 60 66 72 78 84 90 96 102
Months since diagnosis

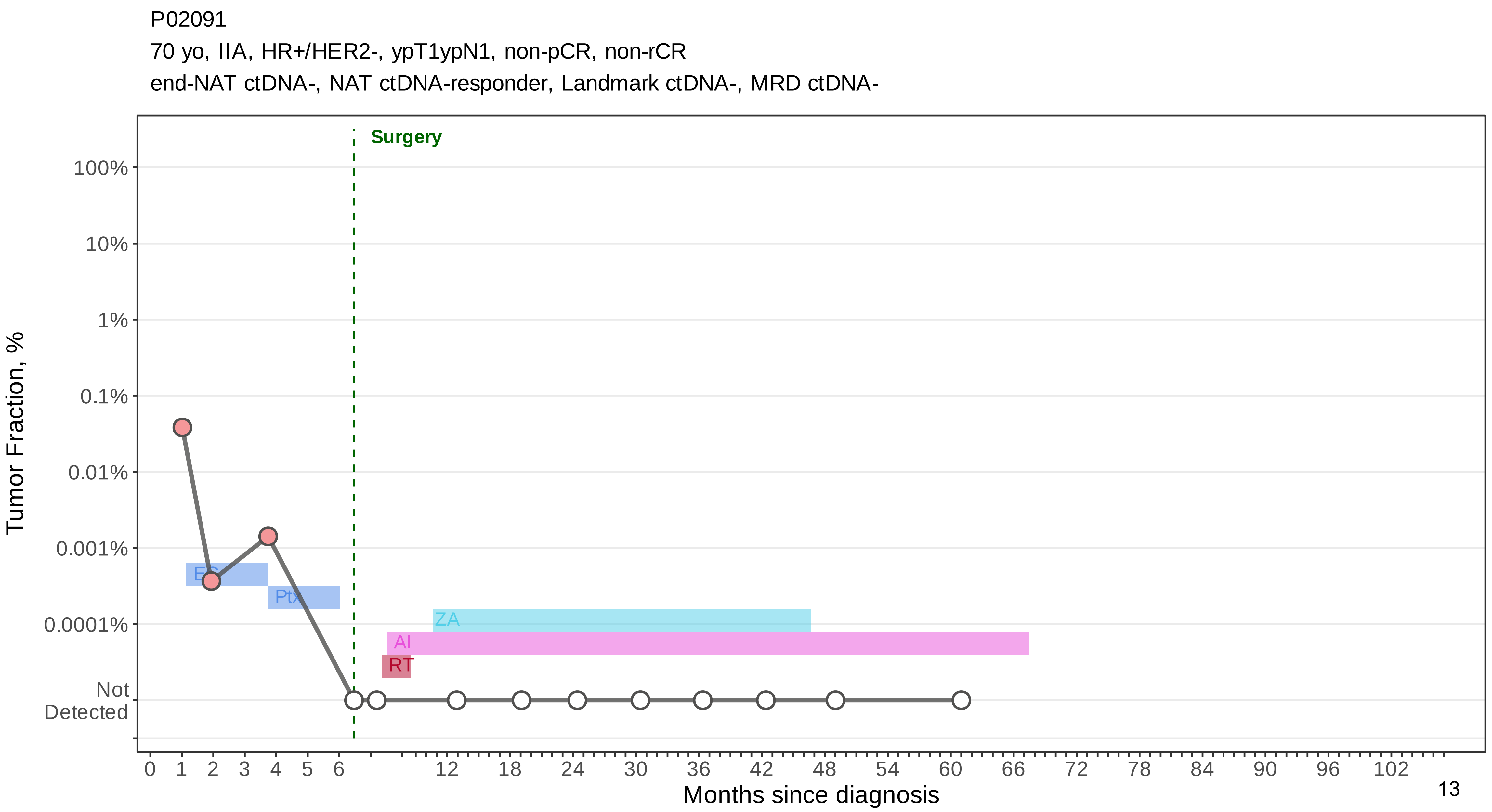
P02091
70 yo, IIA, HR+/HER2-, ypT1ypN1, non-pCR, non-rCR
end-NAT ctDNA-, NAT ctDNA-responder, Landmark ctDNA-, MRD ctDNA-
Surgery
100%
10%
1%
0.1%
0.01%
0.001%
0.0001%
Not Detected
Tumor Fraction, %
Ptx
ZA
AI
RT
0 1 2 3 4 5 6 12 18 24 30 36 42 48 54 60 66 72 78 84 90 96 102
Months since diagnosis

P03091
70 yo, IIB, HR+/HER2-, ypT1ypN1, non-pCR, non-rCR
end-NAT ctDNA-, NAT ctDNA-responder, Landmark ctDNA-, MRD ctDNA-

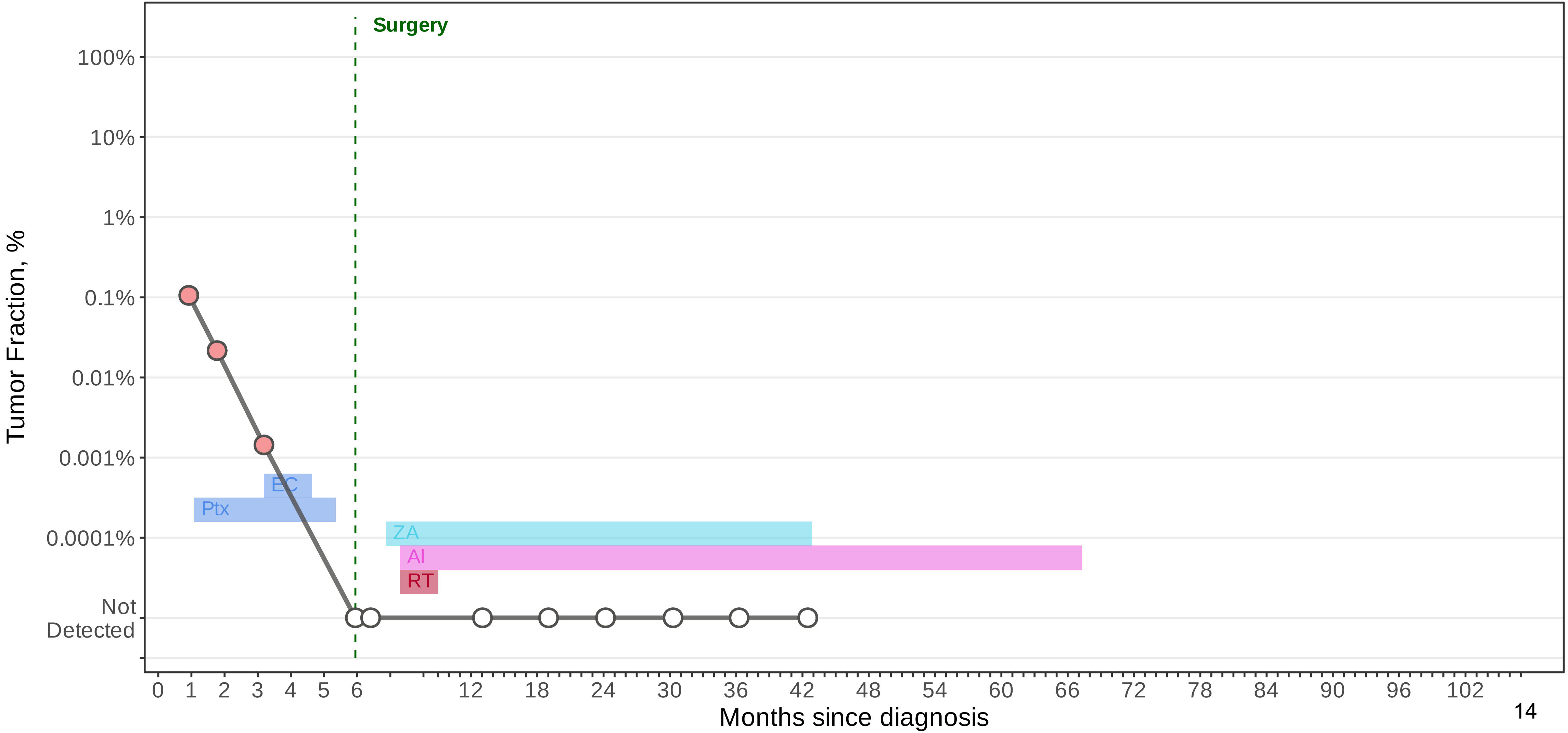

P04091
70 yo, IIB, HR+/HER2-, ypT2ypN1, non-pCR, rCR
end-NAT ctDNA+, NAT ctDNA-non-responder, Landmark ctDNA+, MRD ctDNA+

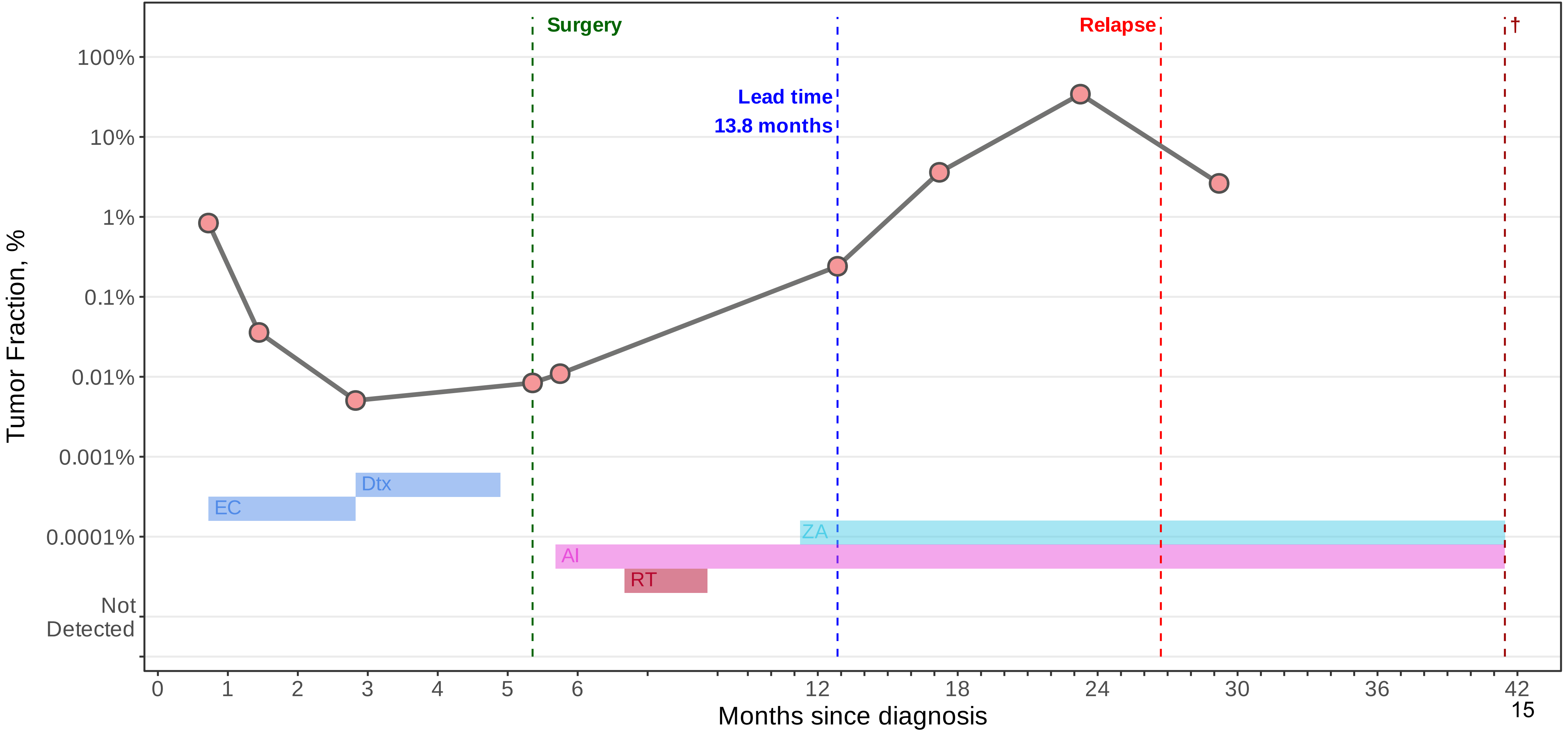

P06091
75 yo, IIIA, HER2+, HR+, ypT2ypN1, non-pCR, rCR
end-NAT ctDNA-, NAT ctDNA-responder, Landmark ctDNA-, MRD ctDNA+

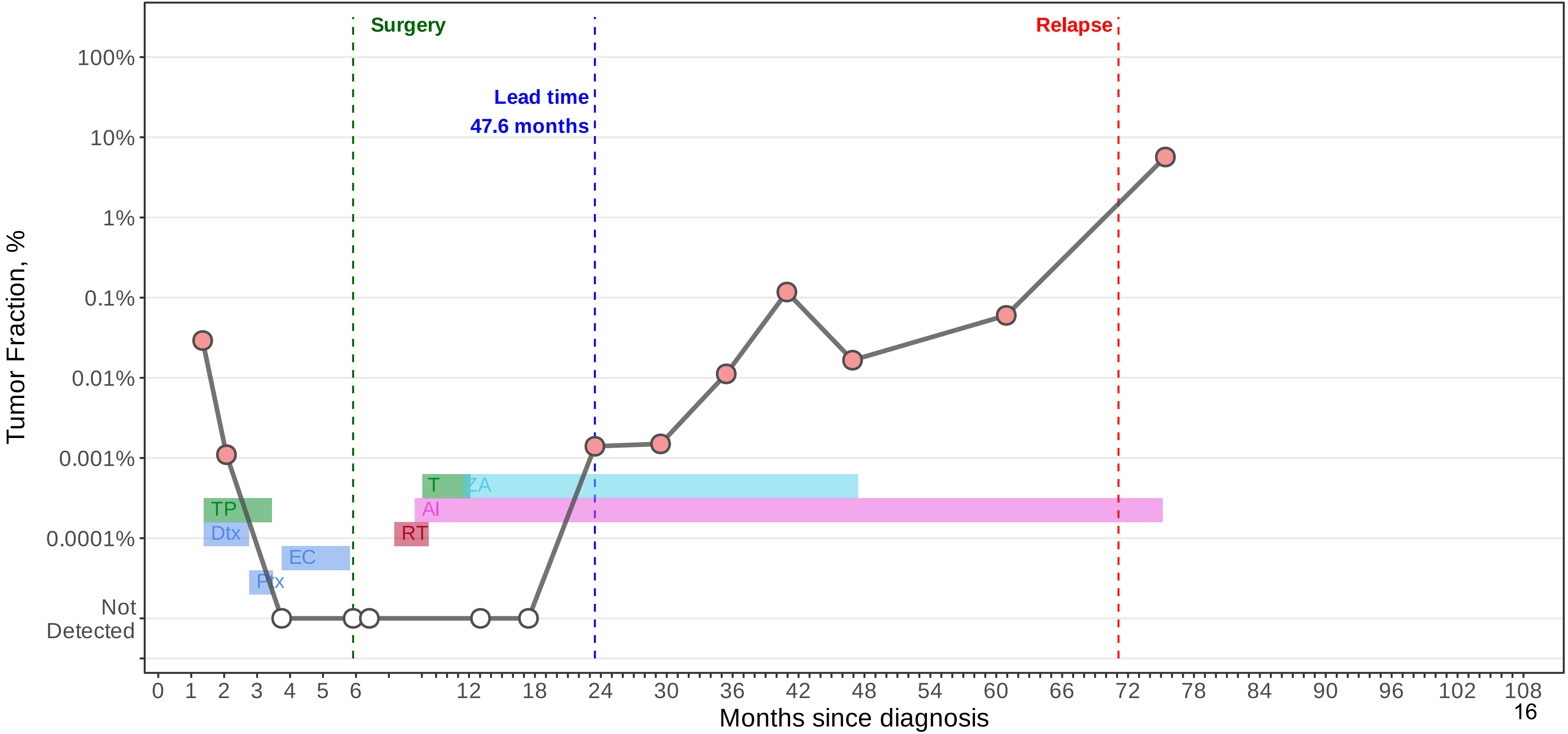

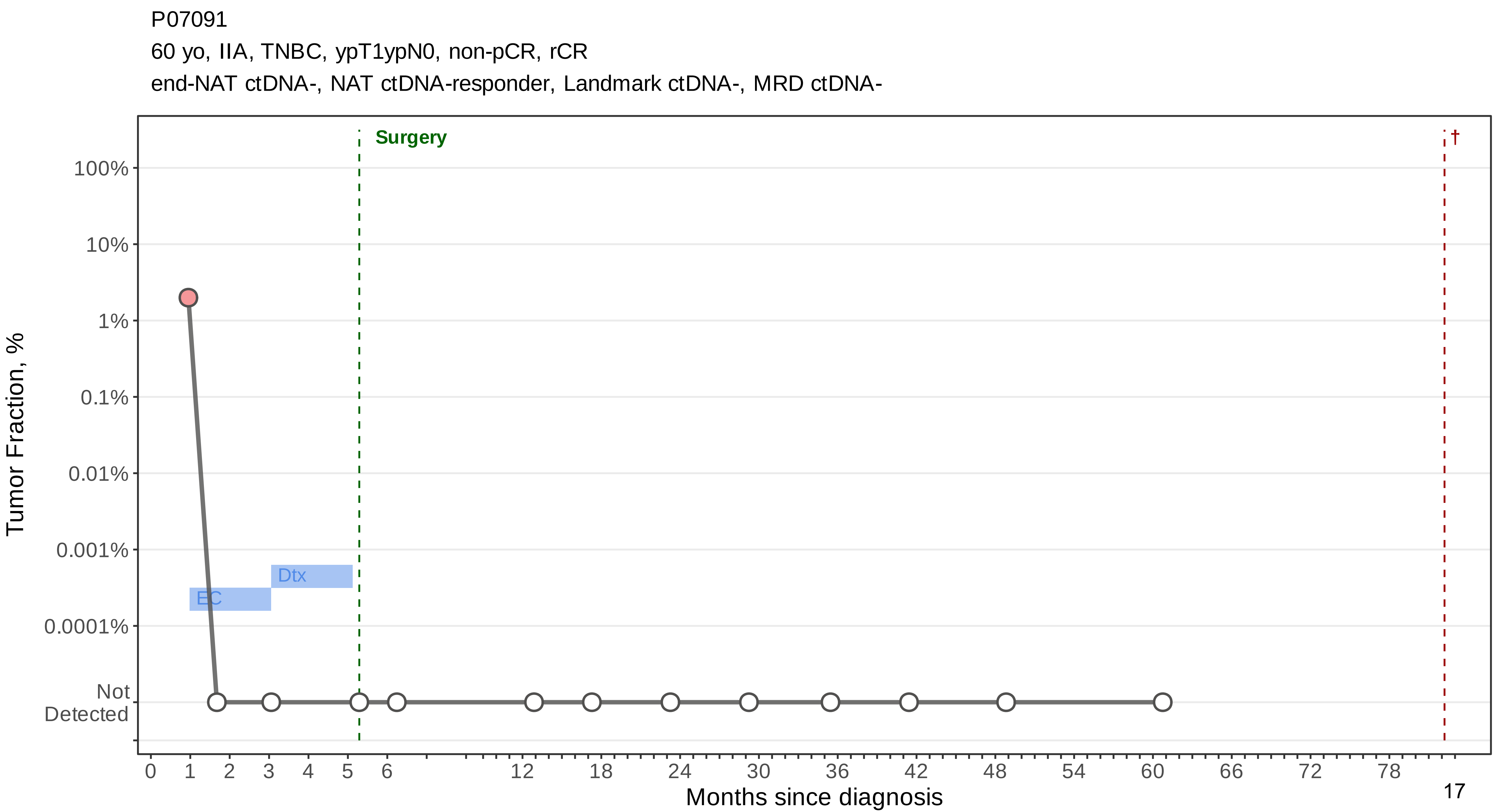

P07091
60 yo, IIA, TNBC, ypT1ypN0, non-pCR, rCR
end-NAT ctDNA-, NAT ctDNA-responder, Landmark ctDNA-, MRD ctDNA-
Surgery
†
100%
10%
1%
0.1%
0.01%
0.001%
0.0001%
Not Detected
Dtx
EC
Tumor Fraction, %
0 1 2 3 4 5 6 12 18 24 30 36 42 48 54 60 66 72 78
Months since diagnosis

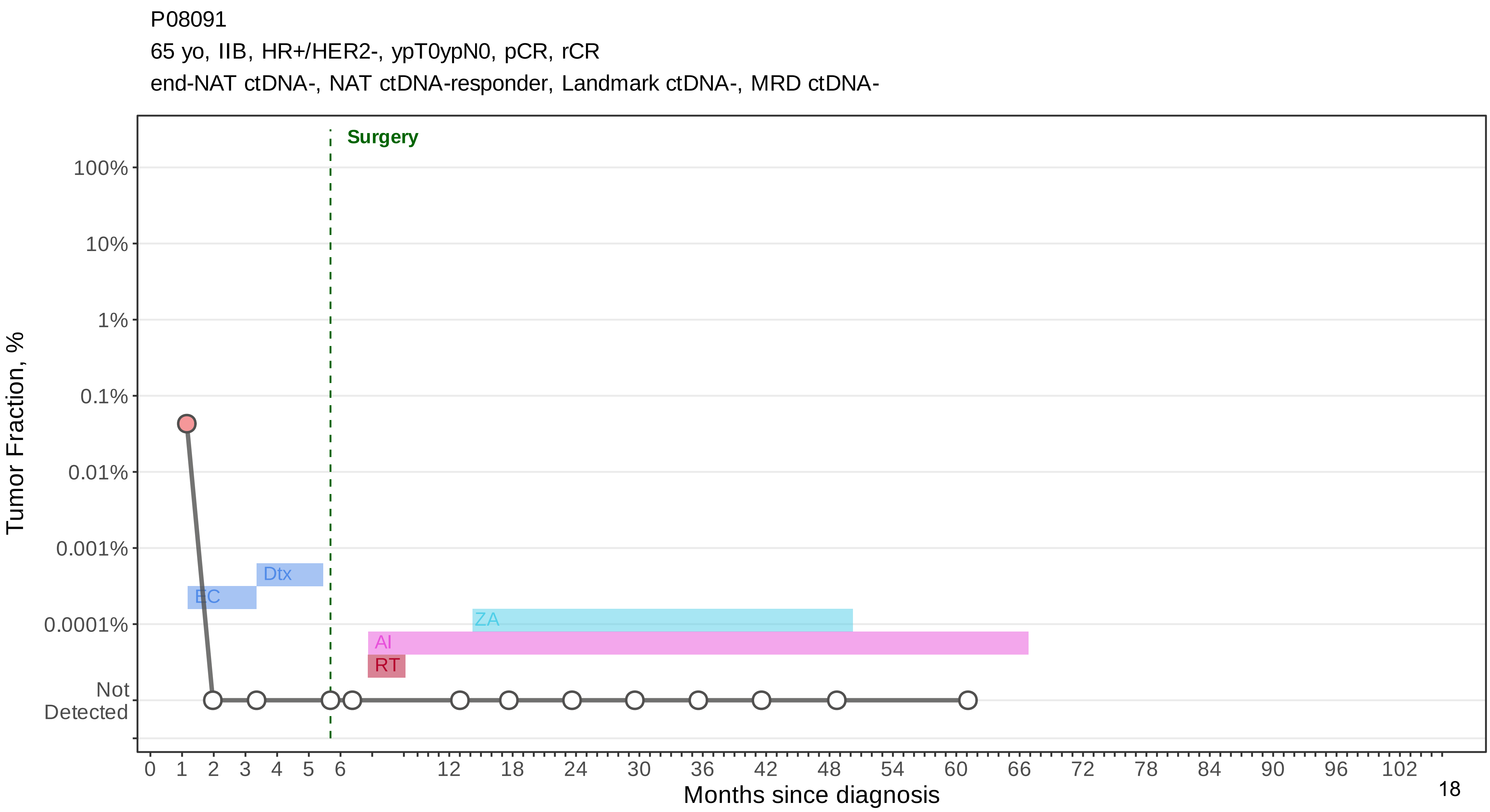
P08091
65 yo, IIB, HR+/HER2-, ypT0ypN0, pCR, rCR
end-NAT ctDNA-, NAT ctDNA-responder, Landmark ctDNA-, MRD ctDNA-
Surgery
Tumor Fraction, %
100%
10%
1%
0.1%
0.01%
0.001%
0.0001%
Not Detected
Dtx
EC
ZA
AI
RT
0 1 2 3 4 5 6 12 18 24 30 36 42 48 54 60 66 72 78 84 90 96 102
Months since diagnosis

P02191
50 yo, IIA, TNBC, ypT0ypN0, pCR, non-rCR
end-NAT ctDNA-, NAT ctDNA-responder, Landmark ctDNA-, MRD ctDNA-

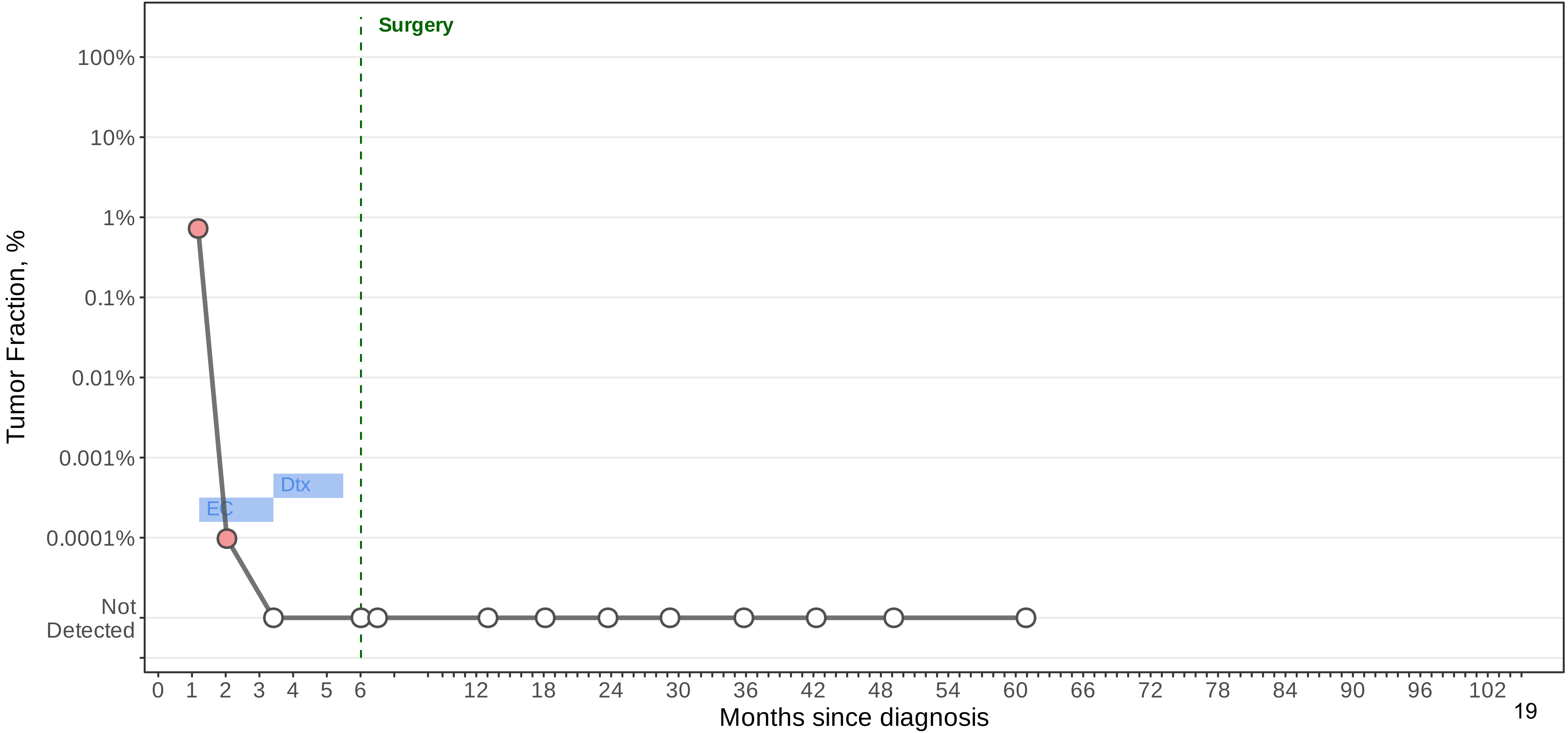

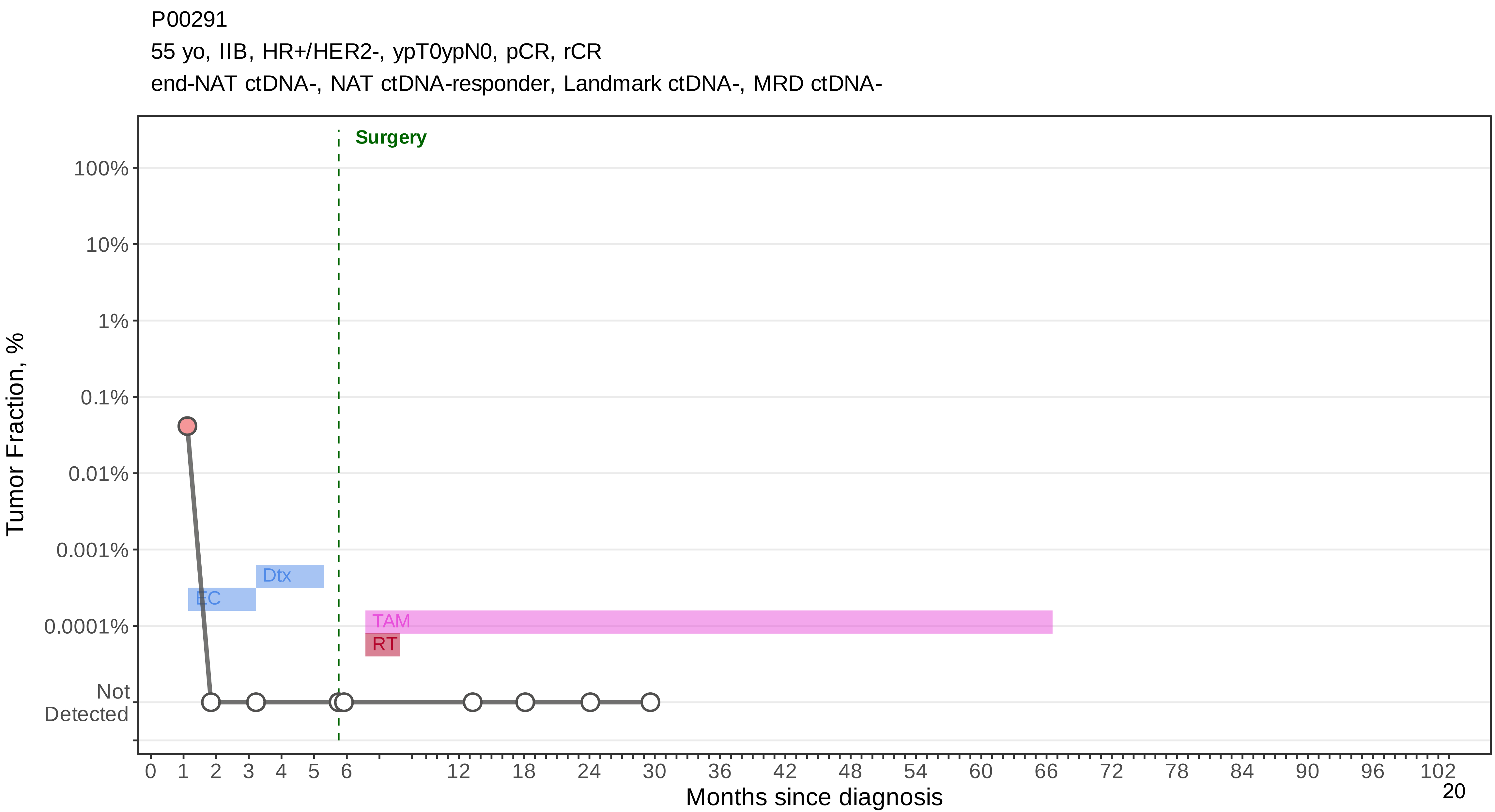
P00291
55 yo, IIB, HR+/HER2-, ypT0ypN0, pCR, rCR
end-NAT ctDNA-, NAT ctDNA-responder, Landmark ctDNA-, MRD ctDNA-
Surgery
100%
10%
1%
0.1%
0.01%
0.001%
0.0001%
Not Detected
Tumor Fraction, %
EC
Dtx
TAM
RT
0 1 2 3 4 5 6 12 18 24 30 36 42 48 54 60 66 72 78 84 90 96 102
Months since diagnosis

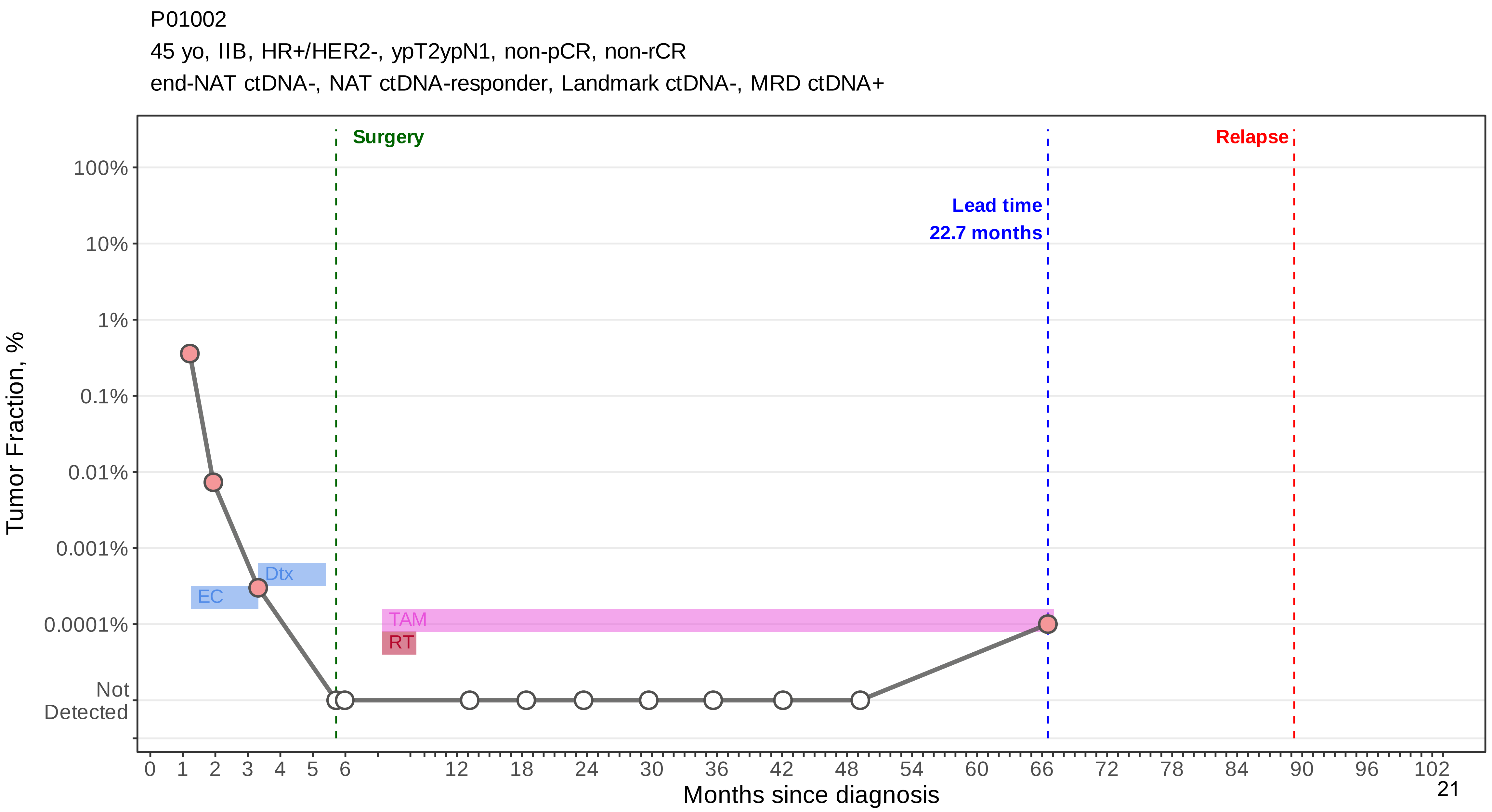

P01002
45 yo, IIB, HR+/HER2-, ypT2ypN1, non-pCR, non-rCR
end-NAT ctDNA-, NAT ctDNA-responder, Landmark ctDNA-, MRD ctDNA+
Surgery
Relapse
Lead time
22.7 months
100%
10%
1%
0.1%
0.01%
0.001%
0.0001%
Not Detected
Tumor Fraction, %
Dtx
EC
TAM
RT
0 1 2 3 4 5 6 12 18 24 30 36 42 48 54 60 66 72 78 84 90 96 102
Months since diagnosis

P02002

50 yo, IIIC, HR+/HER2-, ypT1ypN2, non-pCR, non-rCR

end-NAT ctDNA-, NAT ctDNA-responder, Landmark ctDNA-, MRD ctDNA-

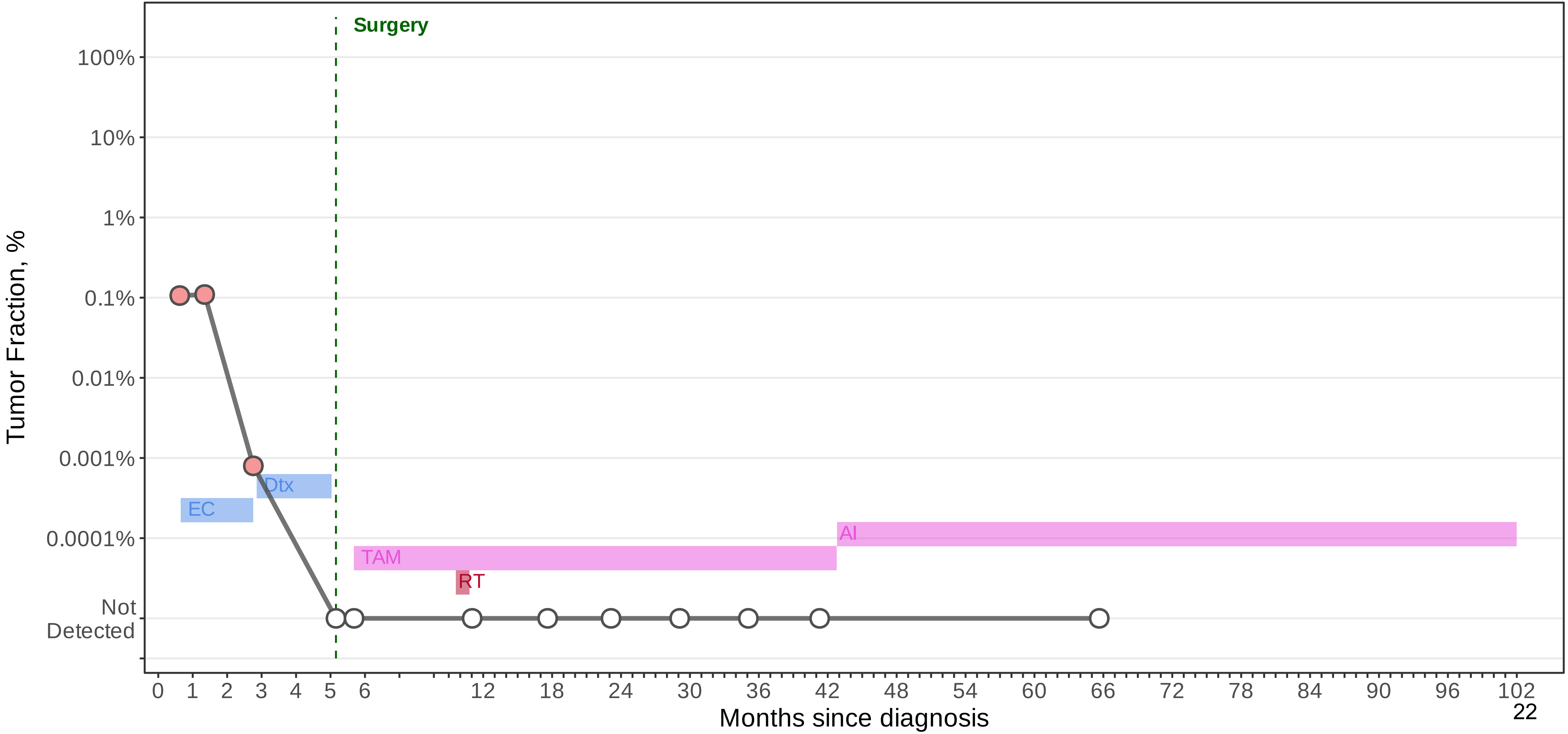

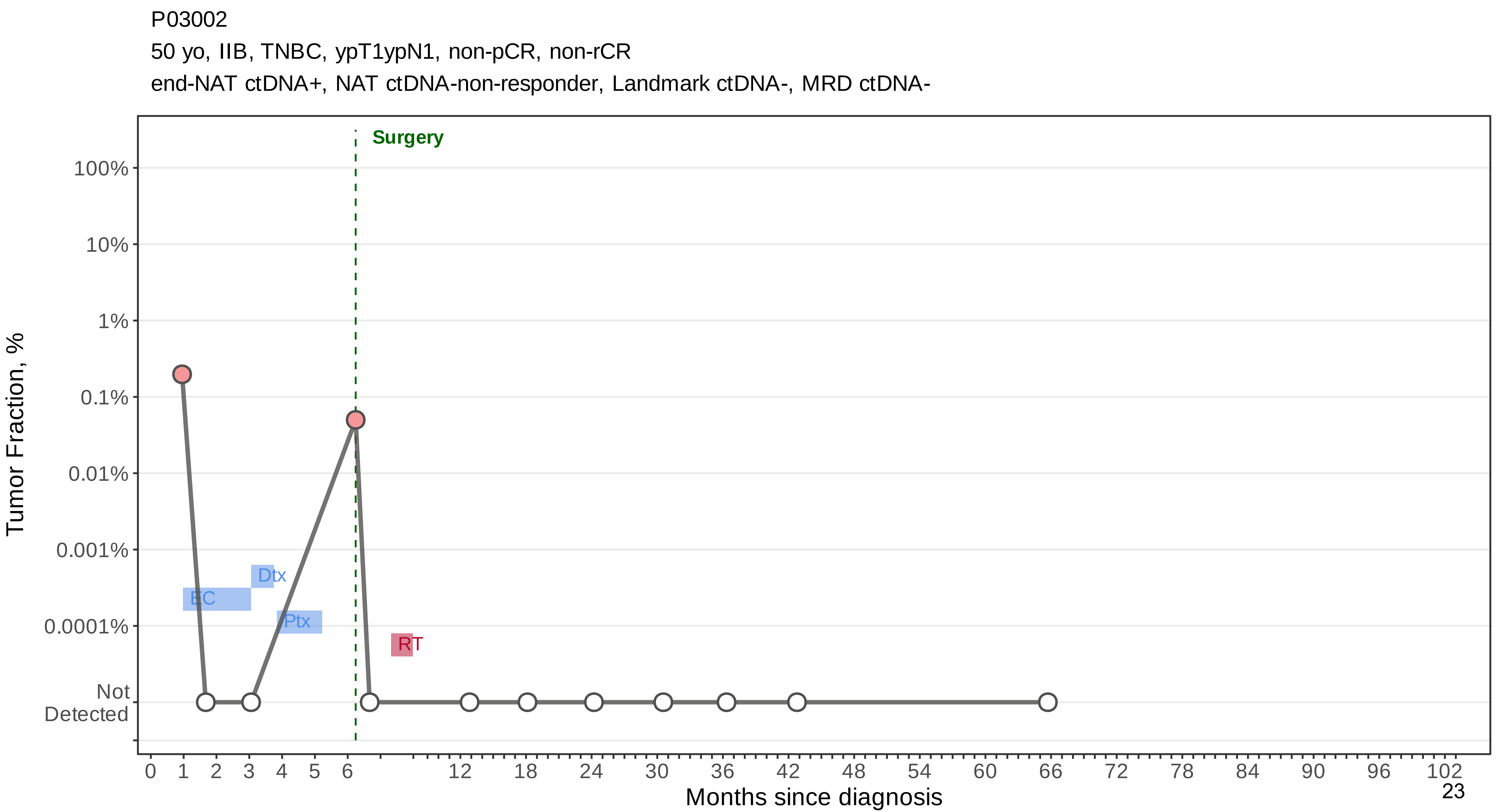
P03002
50 yo, IIB, TNBC, ypT1ypN1, non-pCR, non-rCR
end-NAT ctDNA+, NAT ctDNA-non-responder, Landmark ctDNA-, MRD ctDNA-
Surgery
100%
10%
1%
0.1%
0.01%
0.001%
0.0001%
Not Detected
Tumor Fraction, %
EC
Dtx
Ptx
RT
0 1 2 3 4 5 6 12 18 24 30 36 42 48 54 60 66 72 78 84 90 96 102
Months since diagnosis

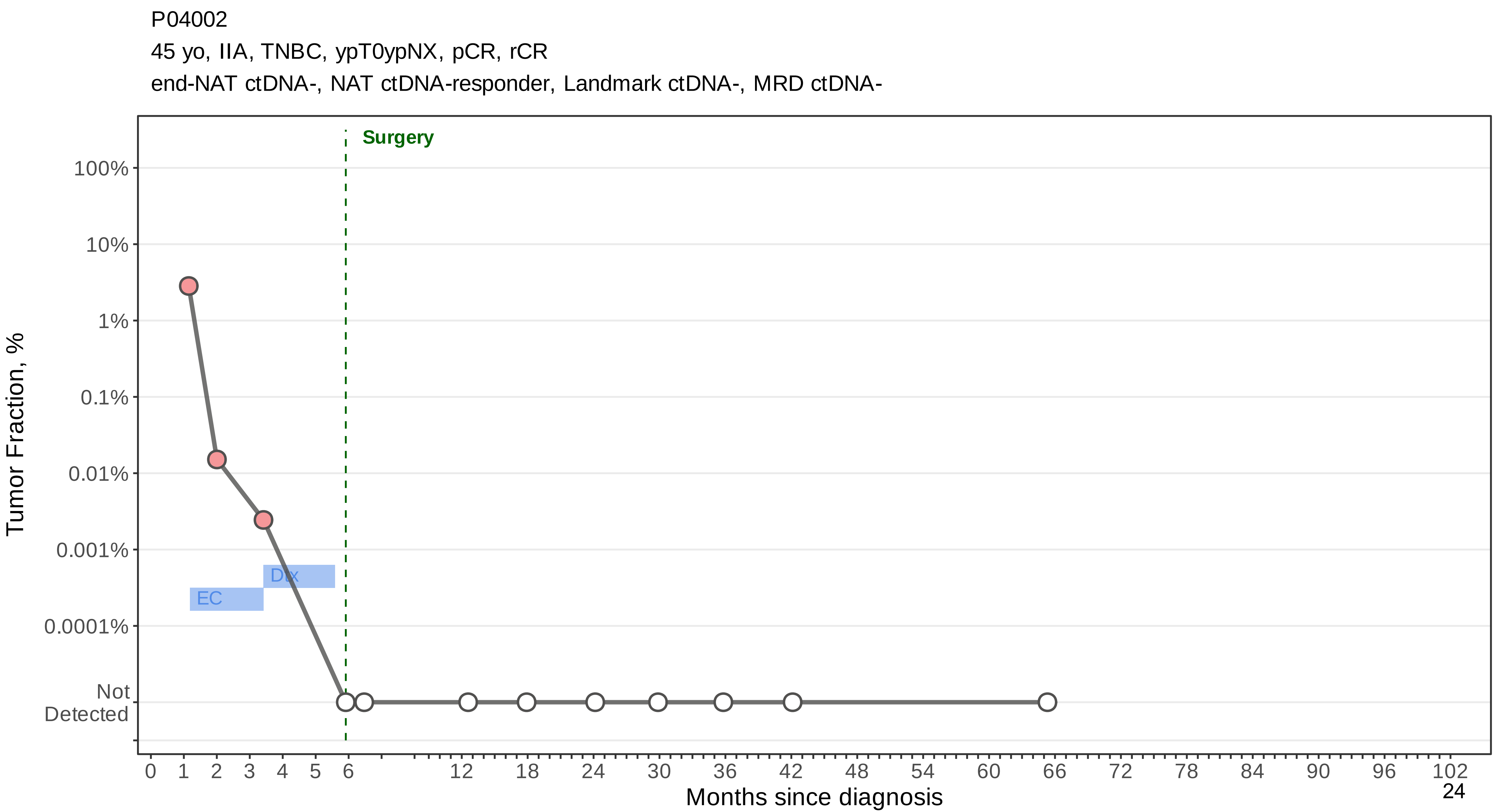
P04002
45 yo, IIA, TNBC, ypT0ypNX, pCR, rCR
end-NAT ctDNA-, NAT ctDNA-responder, Landmark ctDNA-, MRD ctDNA-
Surgery
Tumor Fraction, %
100%
10%
1%
0.1%
0.01%
0.001%
0.0001%
Not Detected
EC
Dtx
0
1
2
3
4
5
6
12
18
24
30
36
42
48
54
60
66
72
78
84
90
96
102
Months since diagnosis

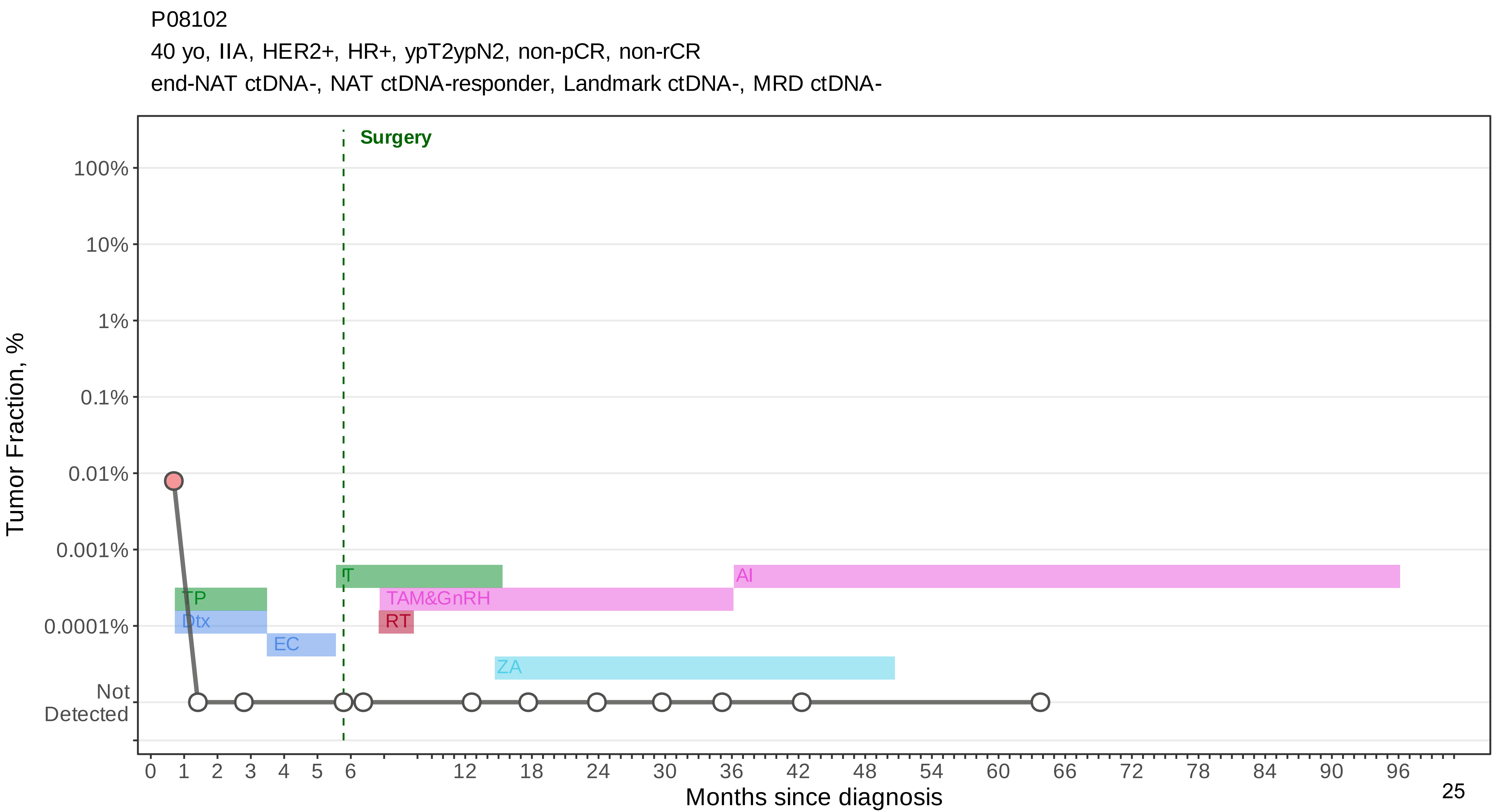
P08102
40 yo, IIA, HER2+, HR+, ypT2ypN2, non-pCR, non-rCR
end-NAT ctDNA-, NAT ctDNA-responder, Landmark ctDNA-, MRD ctDNA-
Surgery
100%
10%
1%
0.1%
0.01%
0.001%
0.0001%
Not Detected
Tumor Fraction, %
TP
Dtx
EC
T
TAM&GnRH
RT
ZA
AI
0 1 2 3 4 5 6 12 18 24 30 36 42 48 54 60 66 72 78 84 90 96
Months since diagnosis

P09102
65 yo, IIB, HR+/HER2-, ypT1ypN2, non-pCR, non-rCR
end-NAT ctDNA-, NAT ctDNA-responder, Landmark ctDNA-, MRD ctDNA-

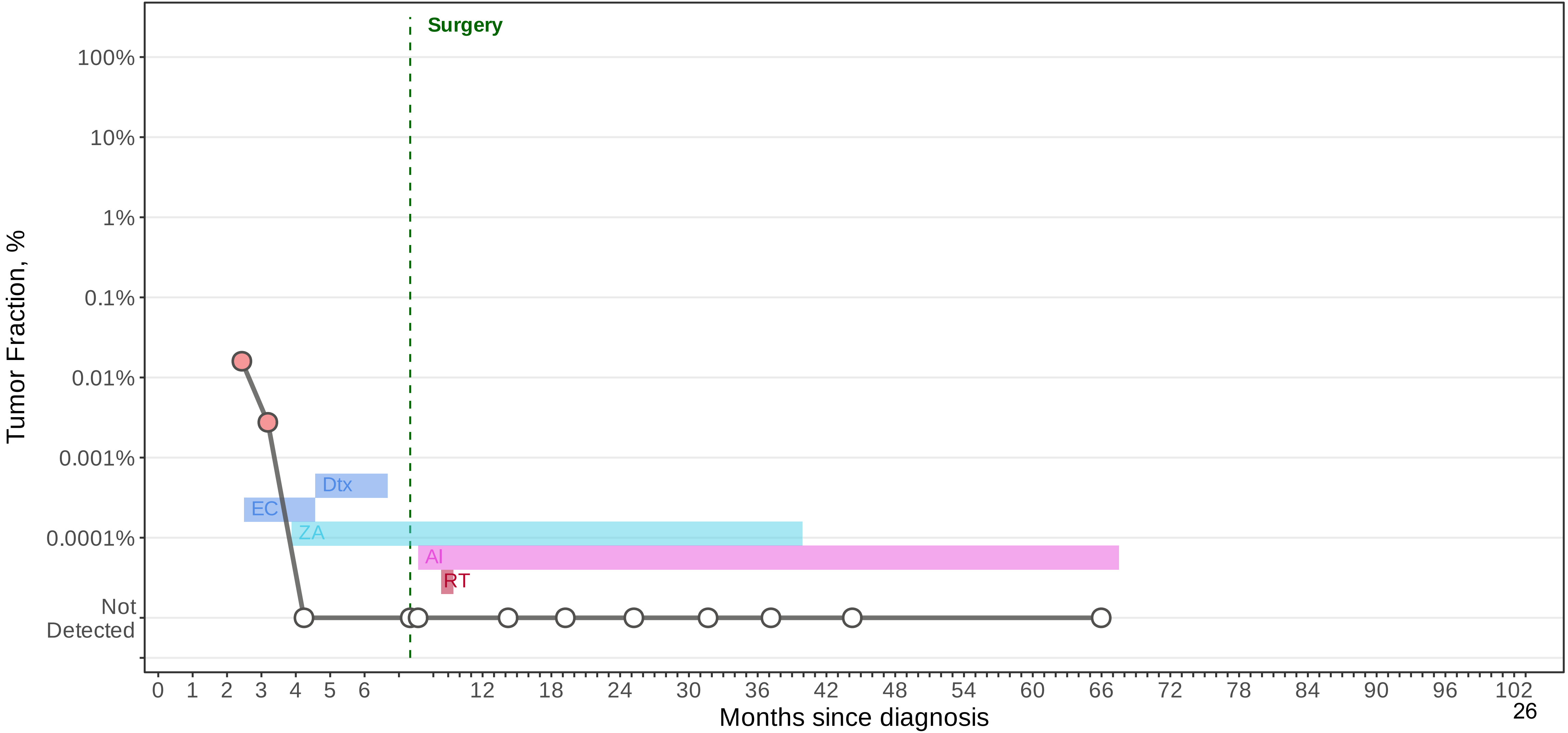

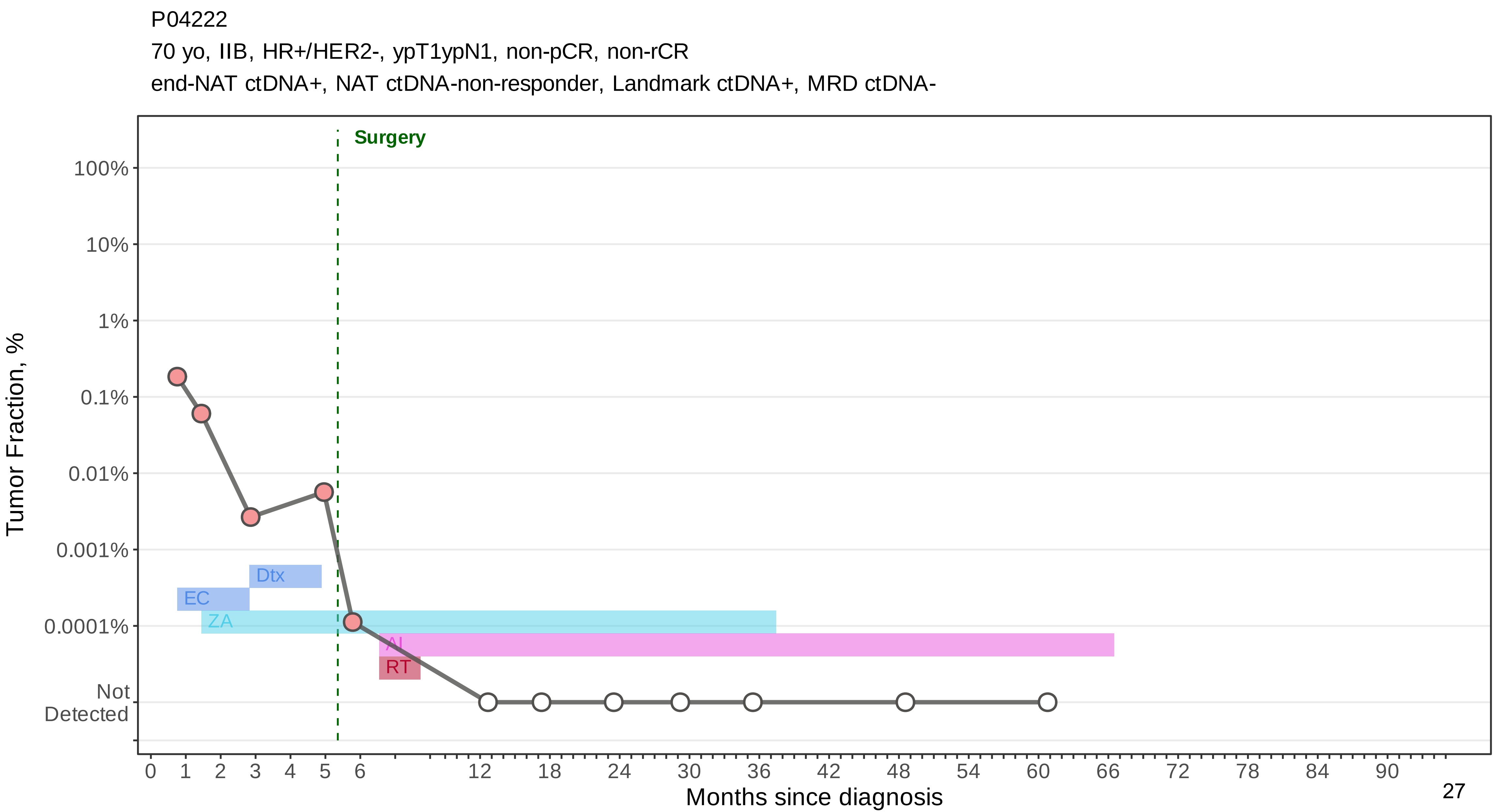

P04222
70 yo, IIB, HR+/HER2-, ypT1ypN1, non-pCR, non-rCR
end-NAT ctDNA+, NAT ctDNA-non-responder, Landmark ctDNA+, MRD ctDNA-
Surgery
Tumor Fraction, %
100%
10%
1%
0.1%
0.01%
0.001%
0.0001%
Not Detected
Dtx
EC
ZA
AI
RT
0 1 2 3 4 5 6 12 18 24 30 36 42 48 54 60 66 72 78 84 90
Months since diagnosis

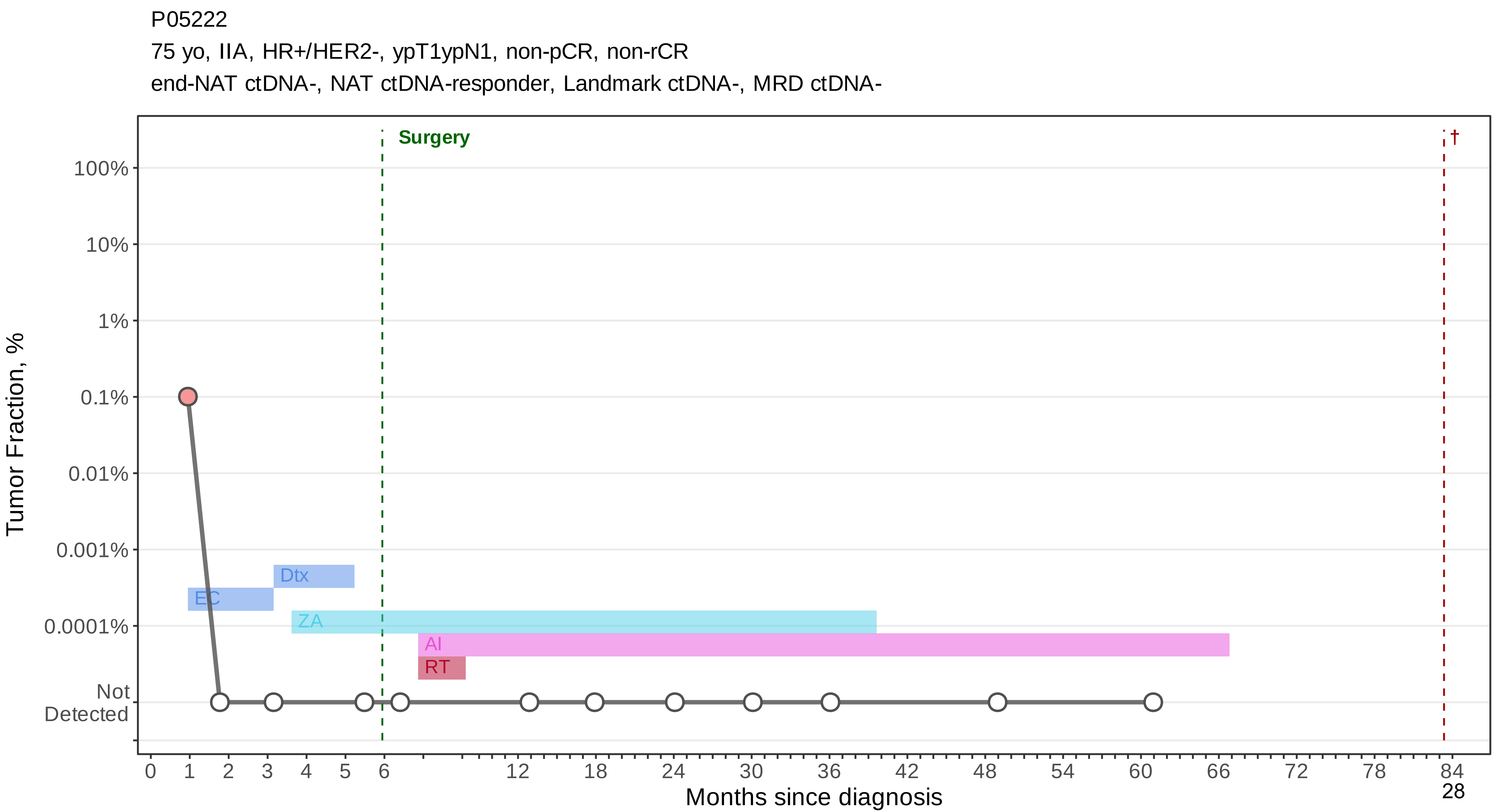
P05222
75 yo, IIA, HR+/HER2-, ypT1ypN1, non-pCR, non-rCR
end-NAT ctDNA-, NAT ctDNA-responder, Landmark ctDNA-, MRD ctDNA-
Surgery
†
100%
10%
1%
0.1%
0.01%
0.001%
0.0001%
Not Detected
Tumor Fraction, %
Dtx
EC
ZA
AI
RT
0
1
2
3
4
5
6
12
18
24
30
36
42
48
54
60
66
72
78
84
Months since diagnosis

P07222
65 yo, IIB, HR+/HER2-, ypT1ypN2, non-pCR, NA
end-NAT ctDNA-, NA, Landmark ctDNA-, MRD ctDNA-

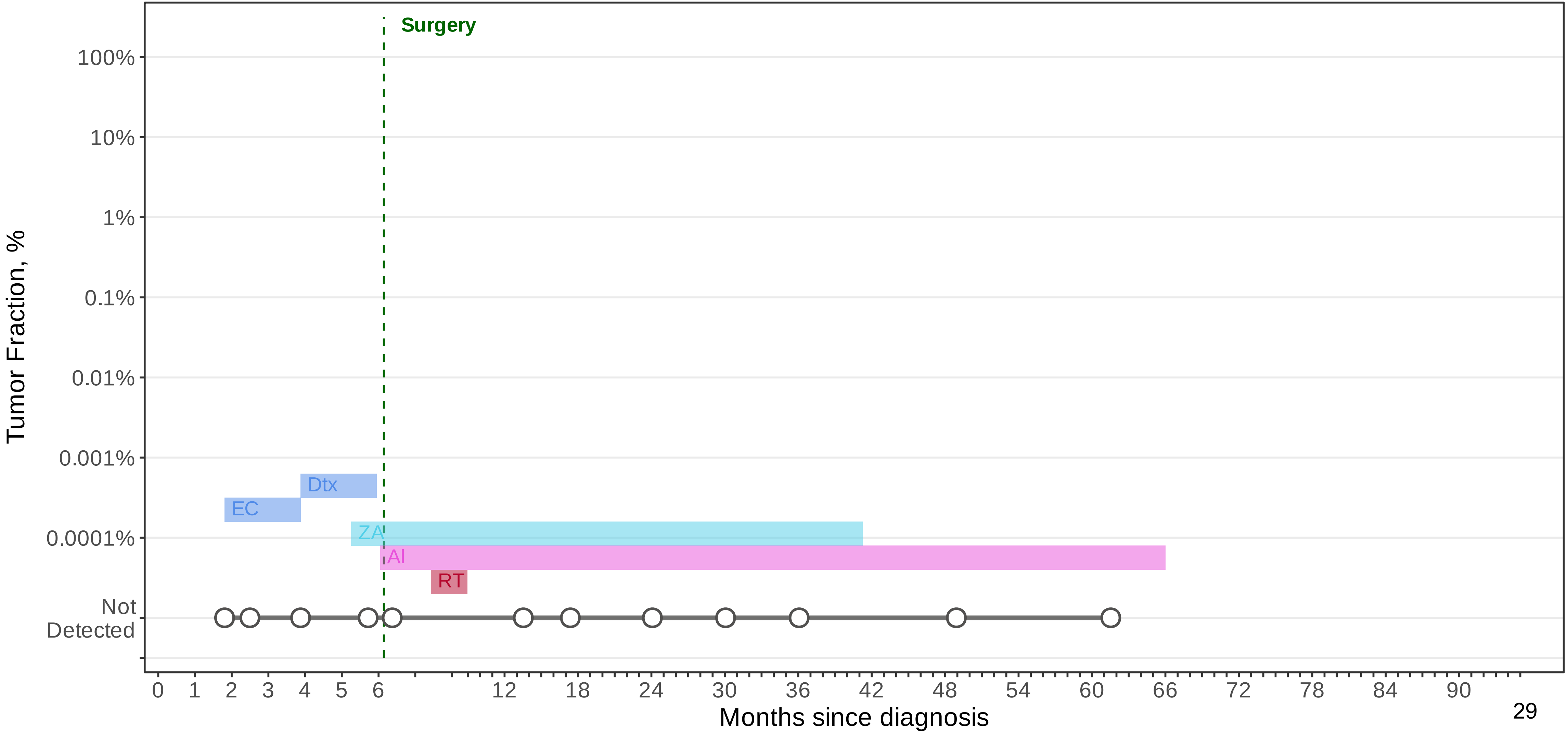

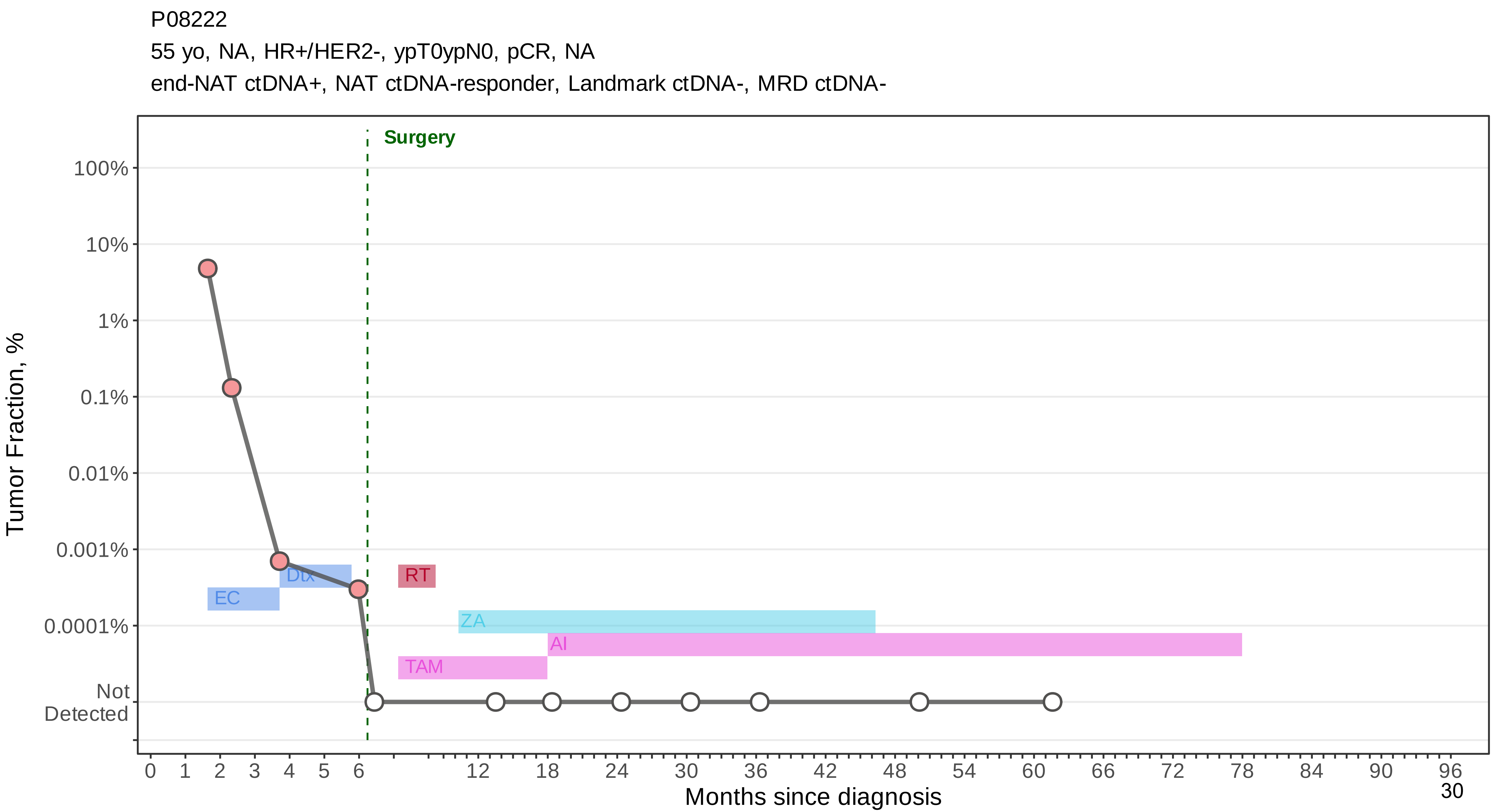
P08222
55 yo, NA, HR+/HER2-, ypT0ypN0, pCR, NA
end-NAT ctDNA+, NAT ctDNA-responder, Landmark ctDNA-, MRD ctDNA-
Surgery
Tumor Fraction, %
100%
10%
1%
0.1%
0.01%
0.001%
0.0001%
Not Detected
EC
Dtx
RT
ZA
AI
TAM
0 1 2 3 4 5 6 12 18 24 30 36 42 48 54 60 66 72 78 84 90 96
Months since diagnosis

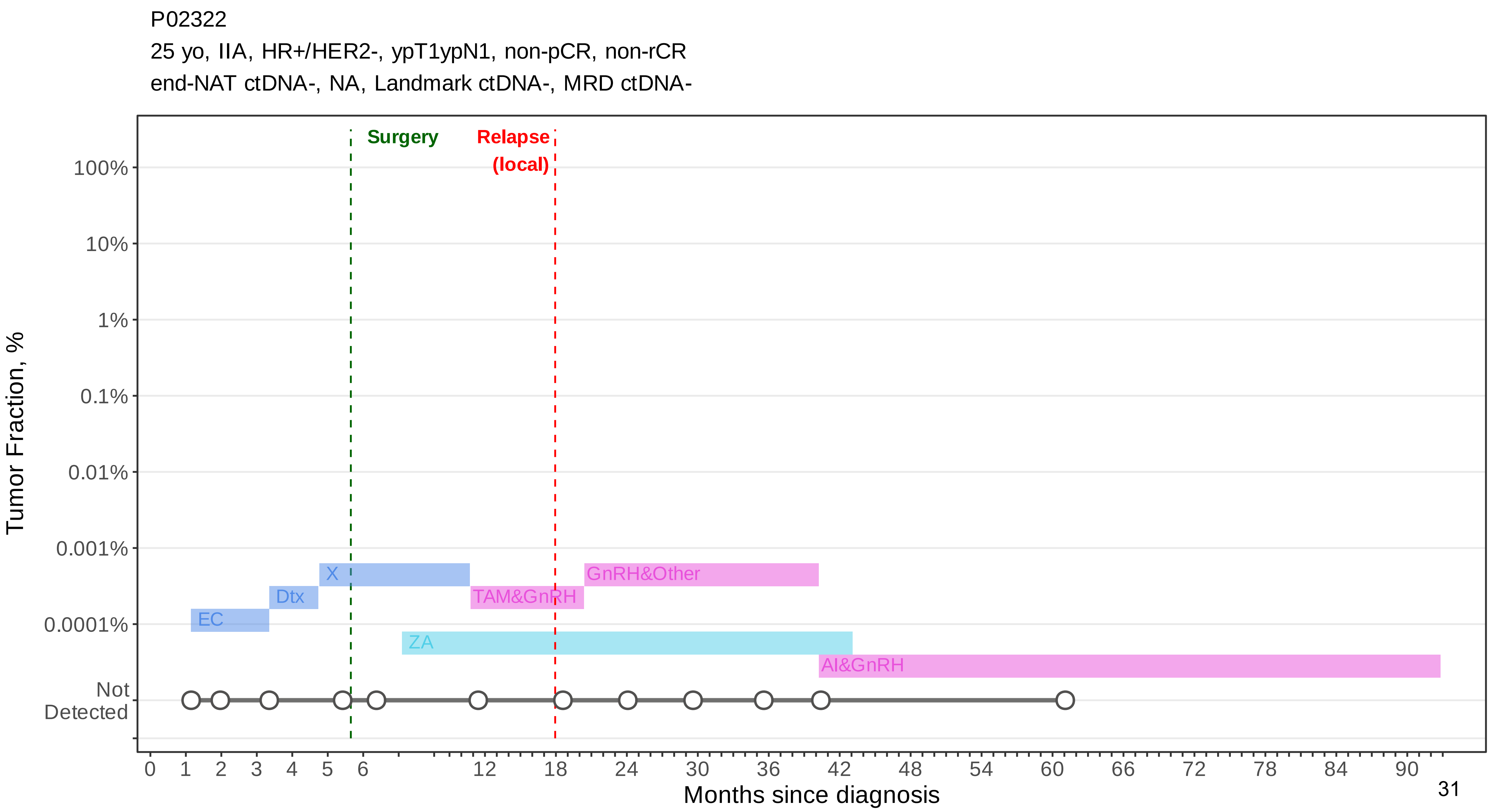
P02322
25 yo, IIA, HR+/HER2-, ypT1ypN1, non-pCR, non-rCR
end-NAT ctDNA-, NA, Landmark ctDNA-, MRD ctDNA-
Surgery
Relapse
(local)
100%
10%
1%
0.1%
0.01%
0.001%
0.0001%
Not
Detected
Tumor Fraction, %
X
Dtx
EC
TAM&GnRH
GnRH&Other
ZA
AI&GnRH
0 1 2 3 4 5 6 12 18 24 30 36 42 48 54 60 66 72 78 84 90
Months since diagnosis

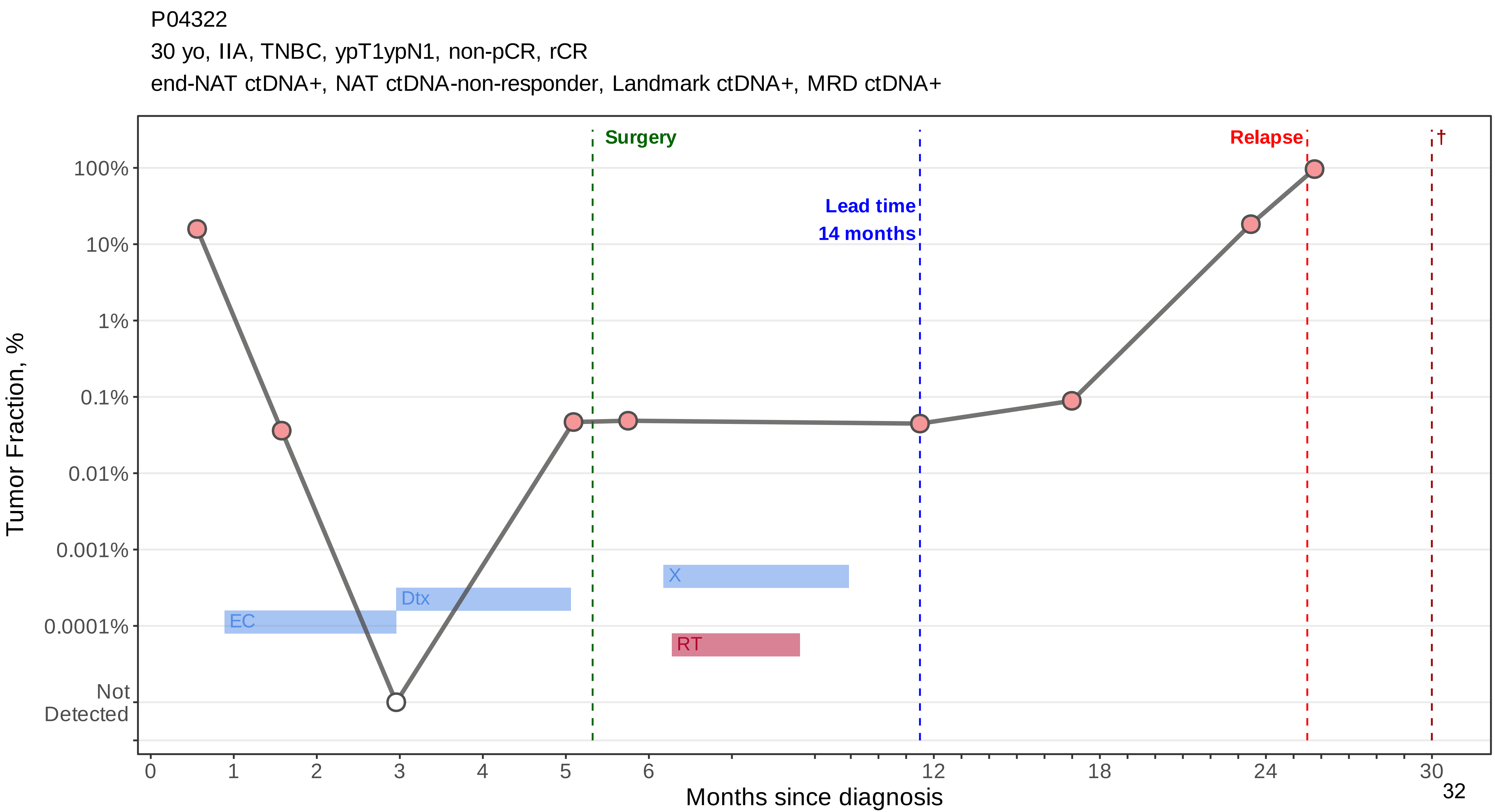
P04322
30 yo, IIA, TNBC, ypT1ypN1, non-pCR, rCR
end-NAT ctDNA+, NAT ctDNA-non-responder, Landmark ctDNA+, MRD ctDNA+
Surgery
Relapse
†
Lead time
14 months
100%
10%
1%
0.1%
0.01%
0.001%
0.0001%
Not Detected
EC
Dtx
X
RT
Tumor Fraction, %
0
1
2
3
4
5
6
12
18
24
30
Months since diagnosis

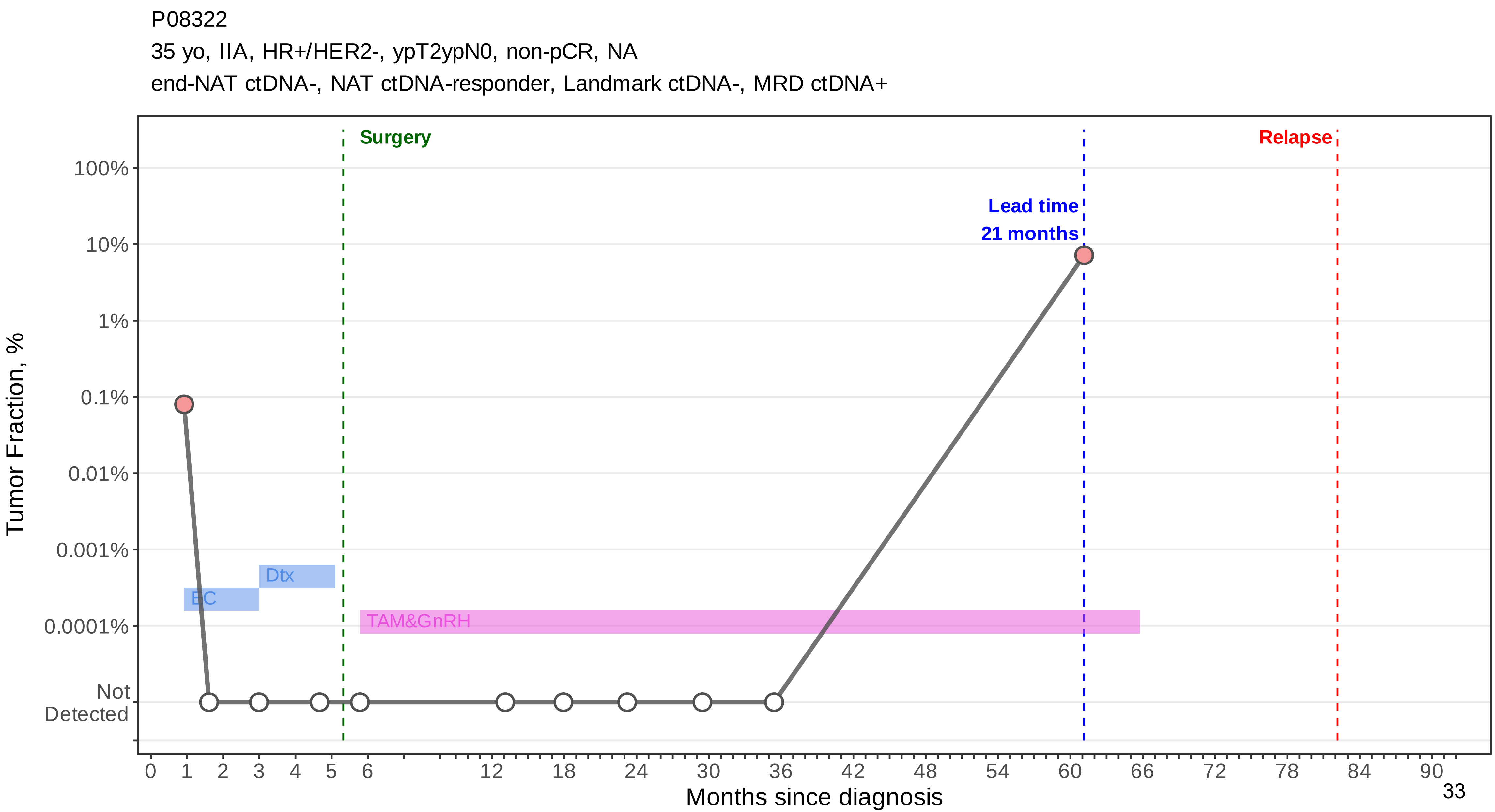

P08322
35 yo, IIA, HR+/HER2-, ypT2ypN0, non-pCR, NA
end-NAT ctDNA-, NAT ctDNA-responder, Landmark ctDNA-, MRD ctDNA+
Surgery
Relapse
Lead time
21 months
100%
10%
1%
0.1%
0.01%
0.001%
0.0001%
Not Detected
Tumor Fraction, %
EC
Dtx
TAM&GnRH
0 1 2 3 4 5 6 12 18 24 30 36 42 48 54 60 66 72 78 84 90
Months since diagnosis

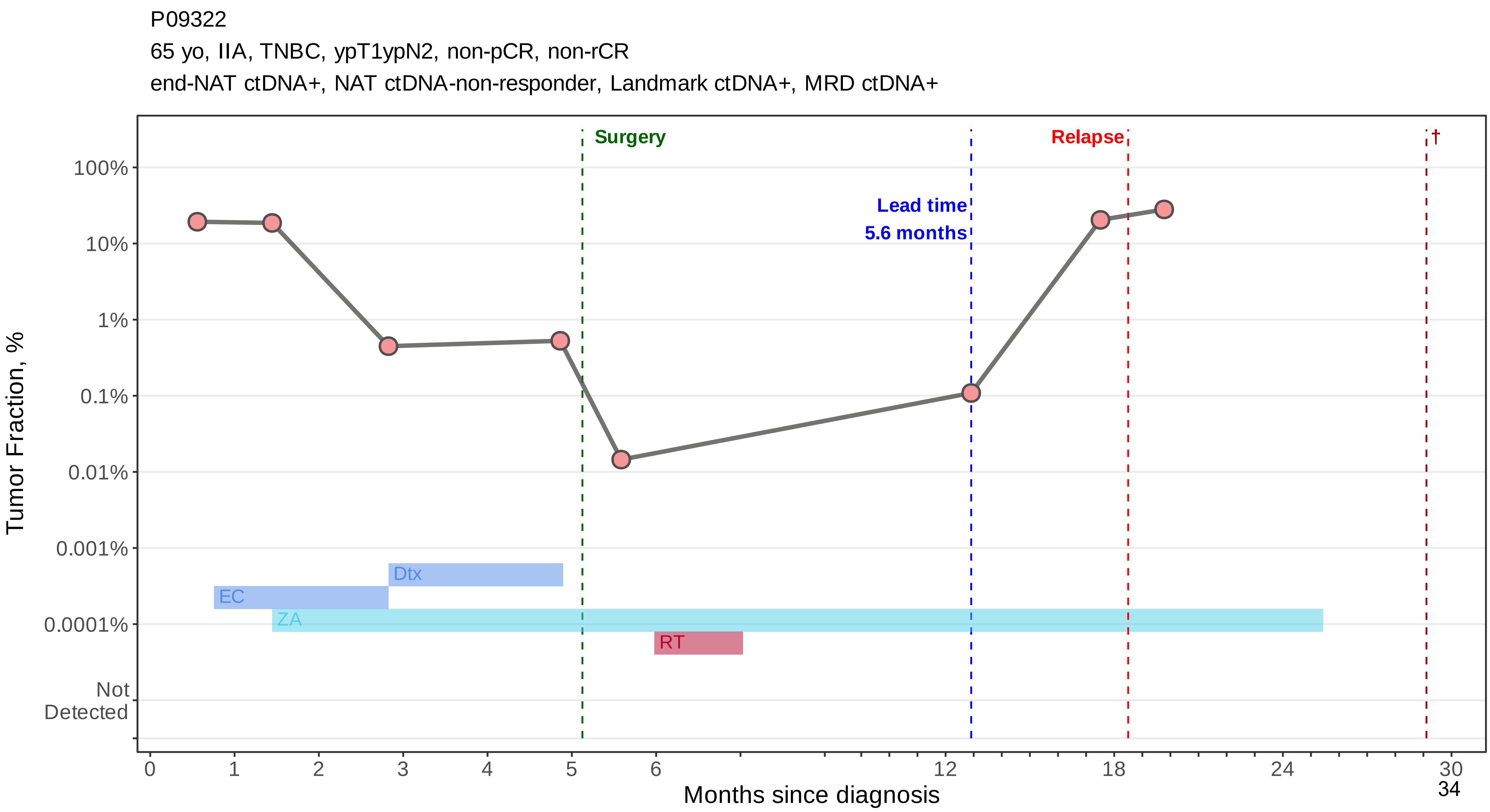
P09322
65 yo, IIA, TNBC, ypT1ypN2, non-pCR, non-rCR
end-NAT ctDNA+, NAT ctDNA-non-responder, Landmark ctDNA+, MRD ctDNA+
Surgery
Relapse
†
Lead time
5.6 months
100%
10%
1%
0.1%
0.01%
0.001%
0.0001%
Not Detected
Tumor Fraction, %
Dtx
EC
ZA
RT
0
1
2
3
4
5
6
12
18
24
30
Months since diagnosis

P06442
55 yo, IIA, TNBC, ypT0ypN0, pCR, rCR
end-NAT ctDNA-, NAT ctDNA-responder, Landmark ctDNA-, MRD ctDNA-

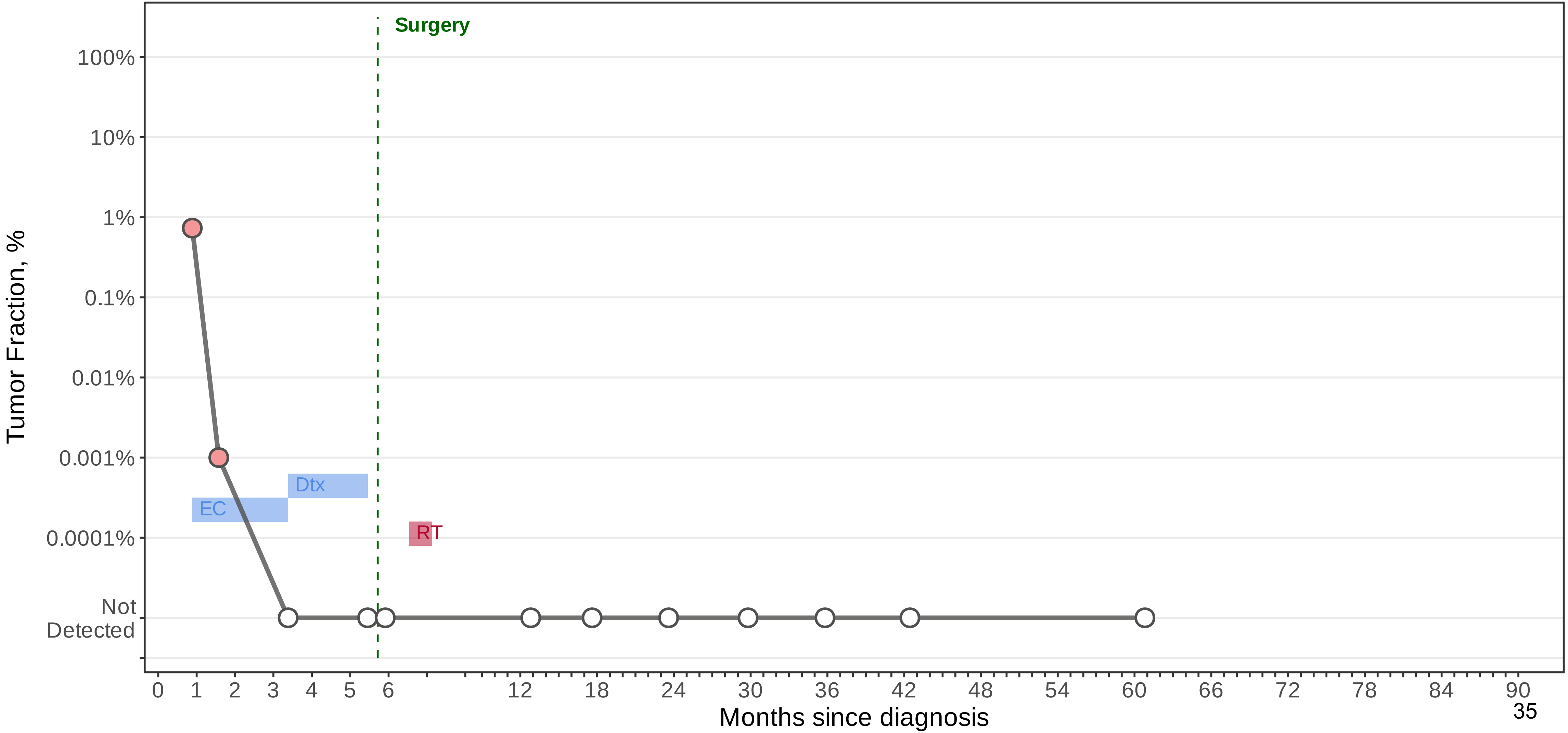

P08442
35 yo, IIA, TNBC, ypT0ypN0, pCR, rCR
end-NAT ctDNA-, NAT ctDNA-responder, Landmark ctDNA-, MRD ctDNA-

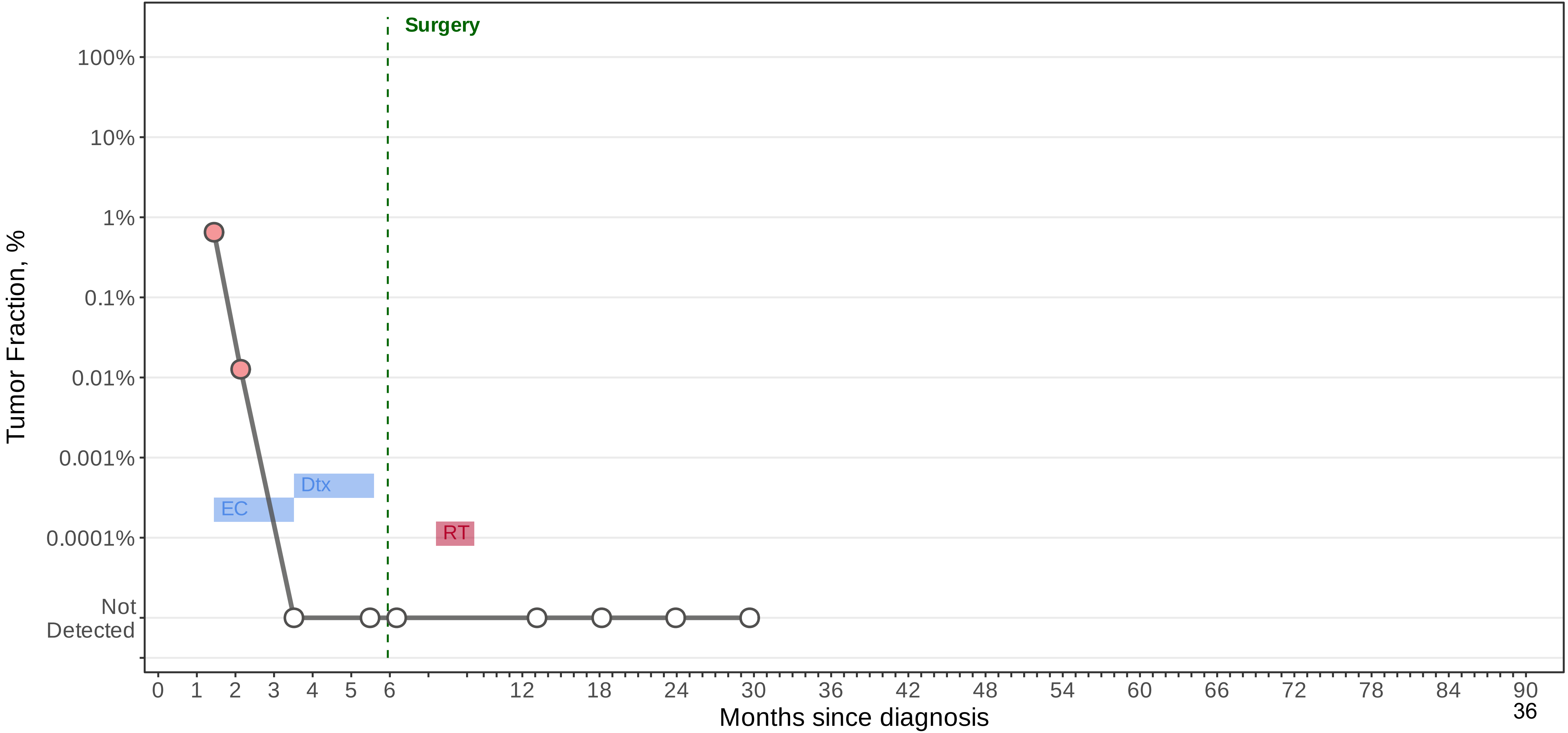

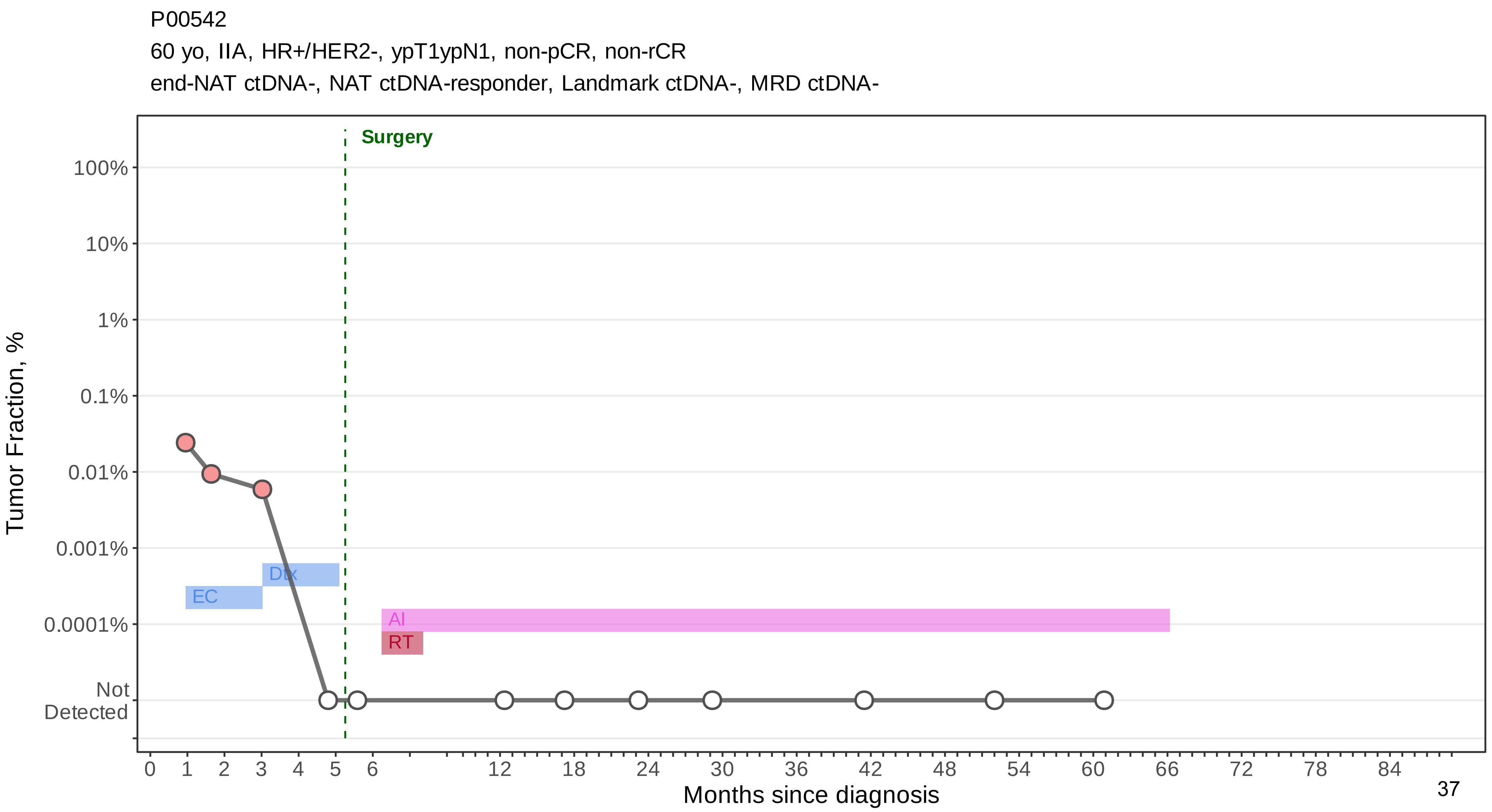
P00542
60 yo, IIA, HR+/HER2-, ypT1ypN1, non-pCR, non-rCR
end-NAT ctDNA-, NAT ctDNA-responder, Landmark ctDNA-, MRD ctDNA-
Surgery
100%
10%
1%
0.1%
0.01%
0.001%
0.0001%
Not Detected
Tumor Fraction, %
Dtx
EC
AI
RT
0 1 2 3 4 5 6 12 18 24 30 36 42 48 54 60 66 72 78 84
Months since diagnosis

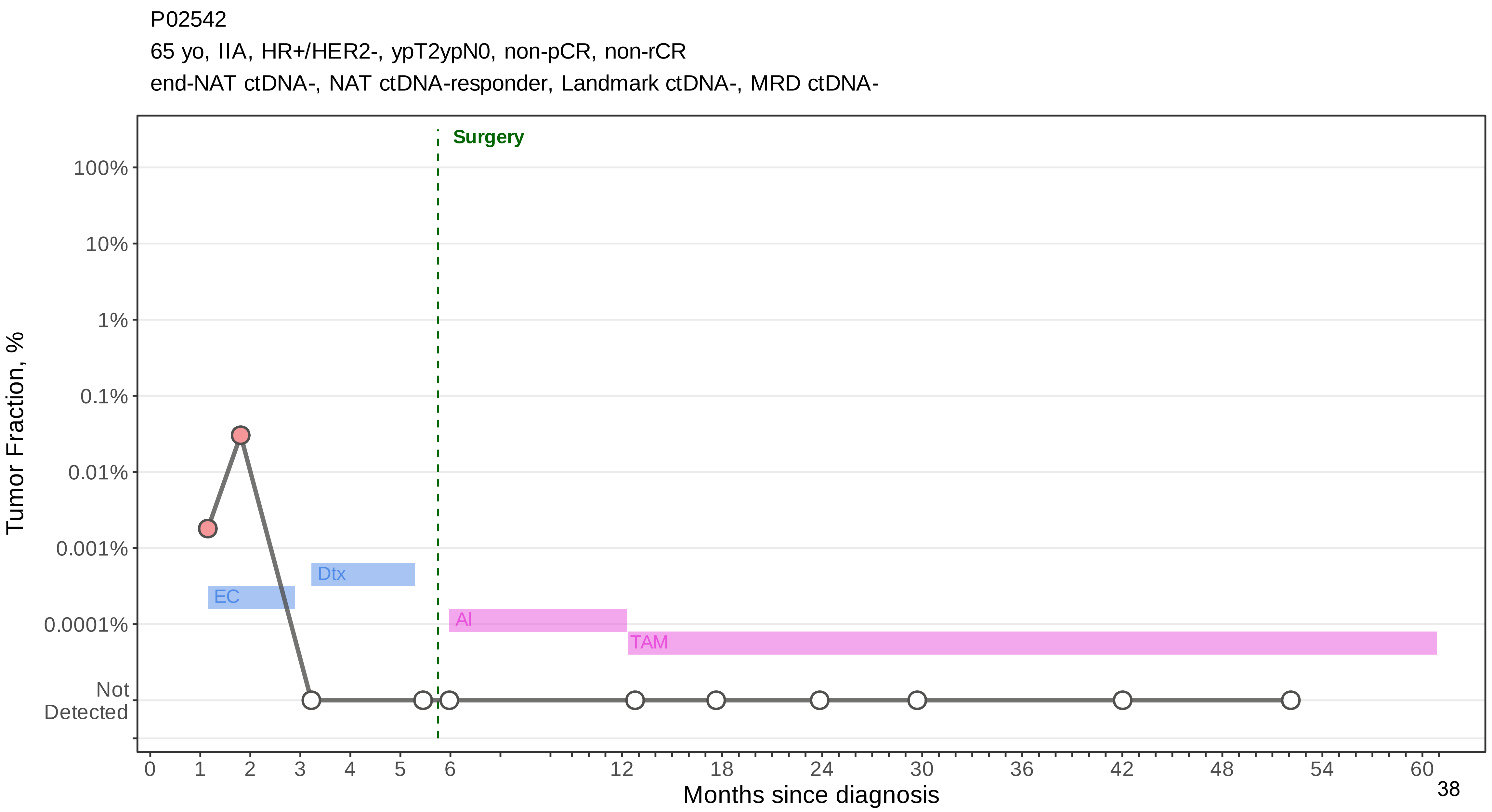
P02542
65 yo, IIA, HR+/HER2-, ypT2ypN0, non-pCR, non-rCR
end-NAT ctDNA-, NAT ctDNA-responder, Landmark ctDNA-, MRD ctDNA-
Surgery
100%
10%
1%
0.1%
0.01%
0.001%
0.0001%
Not Detected
Tumor Fraction, %
Dtx
EC
AI
TAM
0 1 2 3 4 5 6 12 18 24 30 36 42 48 54 60
Months since diagnosis

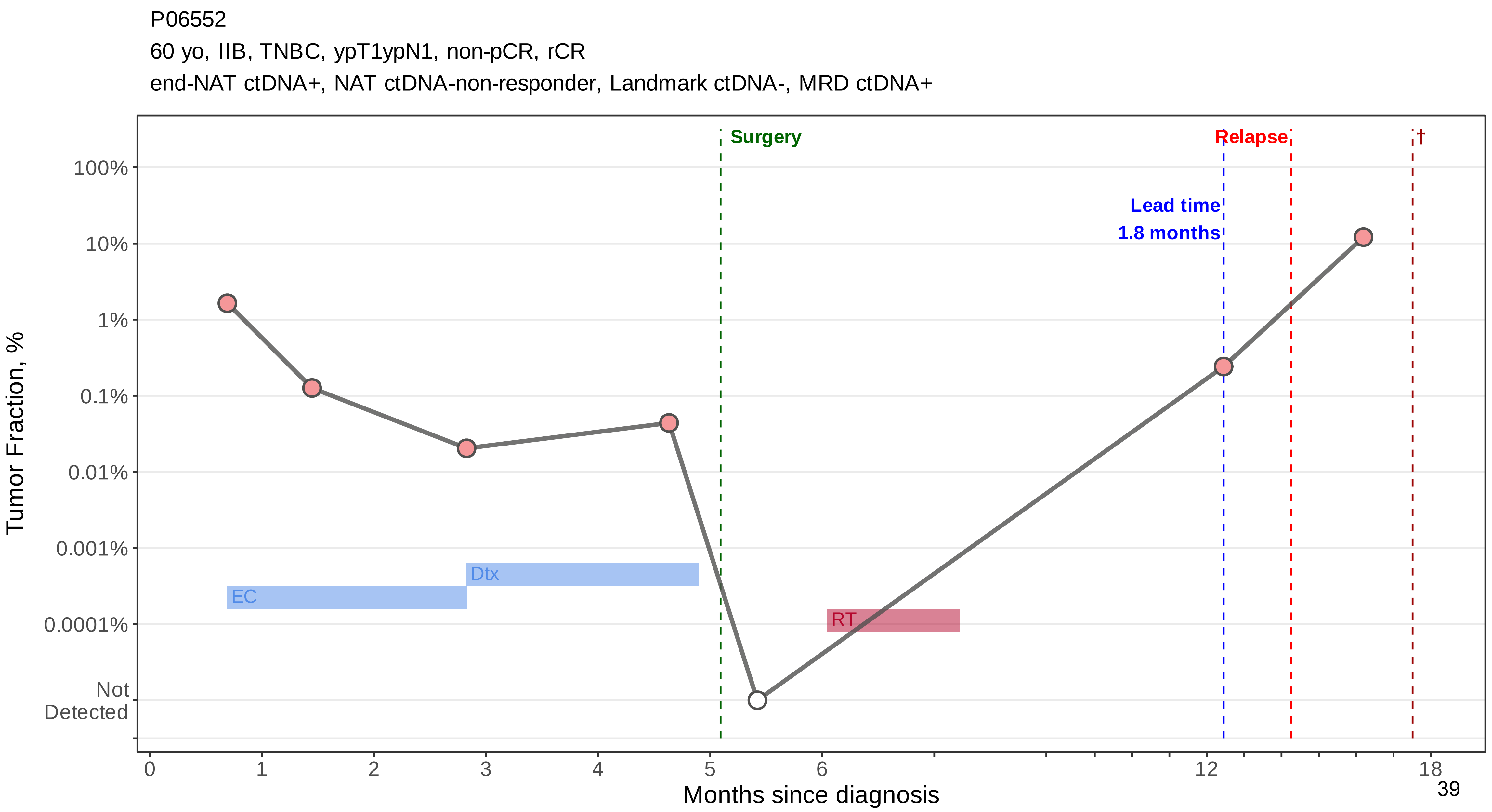
P06552
60 yo, IIB, TNBC, ypT1ypN1, non-pCR, rCR
end-NAT ctDNA+, NAT ctDNA-non-responder, Landmark ctDNA-, MRD ctDNA+
Surgery
Relapse
†
Lead time
1.8 months
EC
Dtx
RT
Tumor Fraction, %
100%
10%
1%
0.1%
0.01%
0.001%
0.0001%
Not Detected
Months since diagnosis
0
1
2
3
4
5
6
12
18

P07552
50 yo, IIB, HR+/HER2-, ypT1ypN3, non-pCR, non-rCR
end-NAT ctDNA-, NAT ctDNA-responder, Landmark ctDNA-, MRD ctDNA-

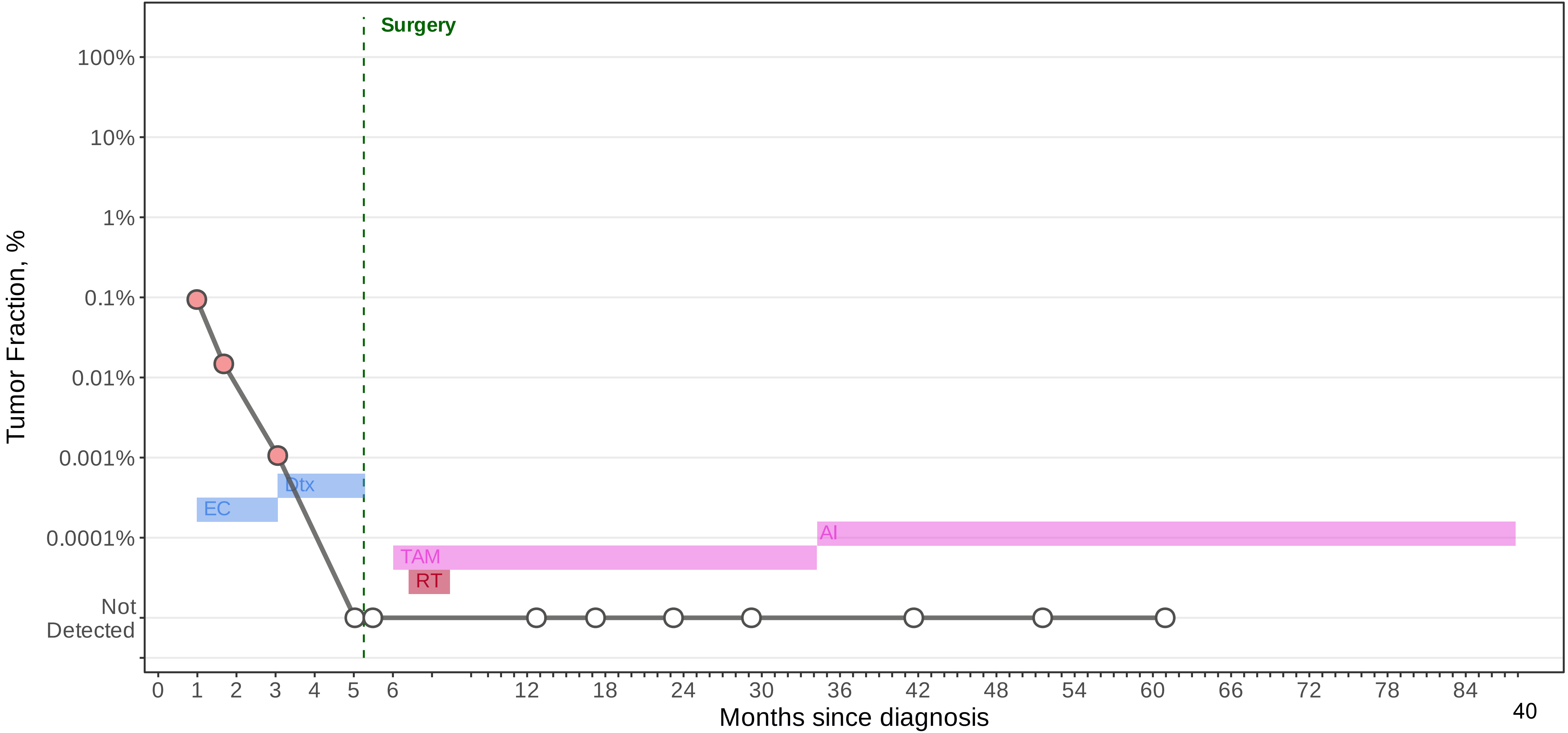

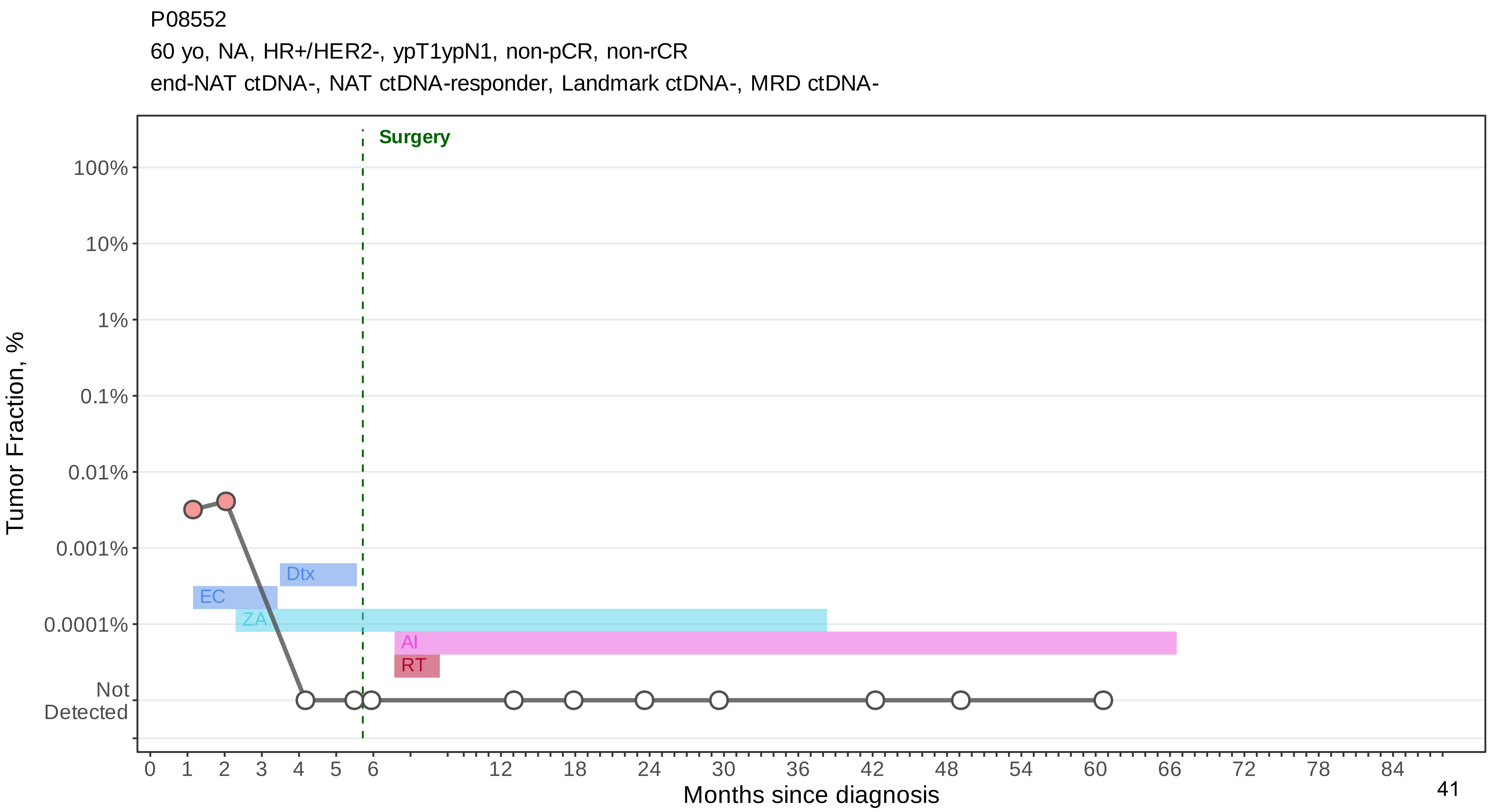
P08552
60 yo, NA, HR+/HER2-, ypT1ypN1, non-pCR, non-rCR
end-NAT ctDNA-, NAT ctDNA-responder, Landmark ctDNA-, MRD ctDNA-
Surgery
100%
10%
1%
0.1%
0.01%
0.001%
0.0001%
Not Detected
Tumor Fraction, %
Dtx
EC
ZA
AI
RT
0 1 2 3 4 5 6 12 18 24 30 36 42 48 54 60 66 72 78 84
Months since diagnosis

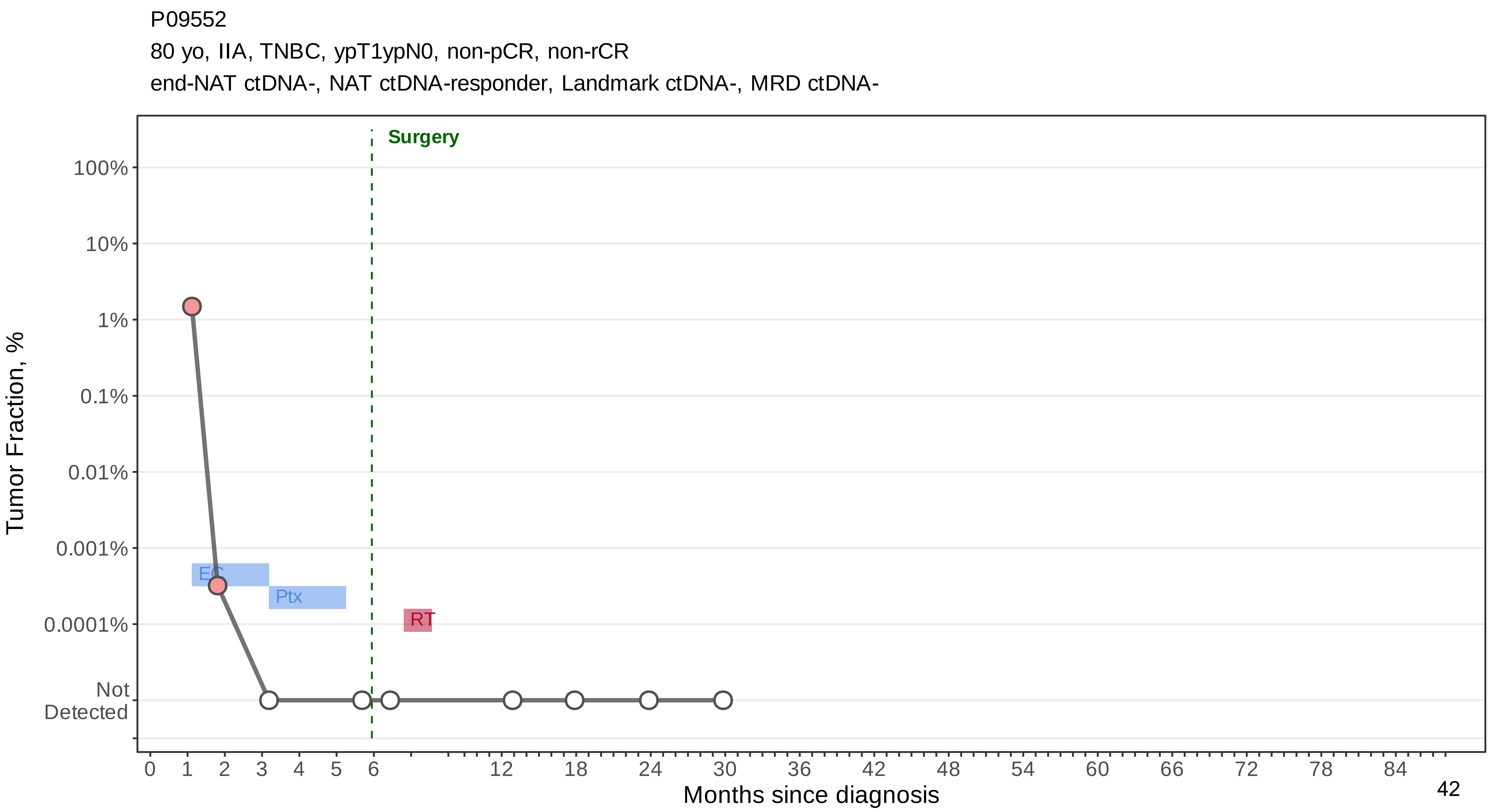

P09552
80 yo, IIA, TNBC, ypT1ypN0, non-pCR, non-rCR
end-NAT ctDNA-, NAT ctDNA-responder, Landmark ctDNA-, MRD ctDNA-
Surgery
100%
10%
1%
0.1%
0.01%
0.001%
0.0001%
Not Detected
EC
Ptx
RT
Tumor Fraction, %
0 1 2 3 4 5 6 12 18 24 30 36 42 48 54 60 66 72 78 84
Months since diagnosis

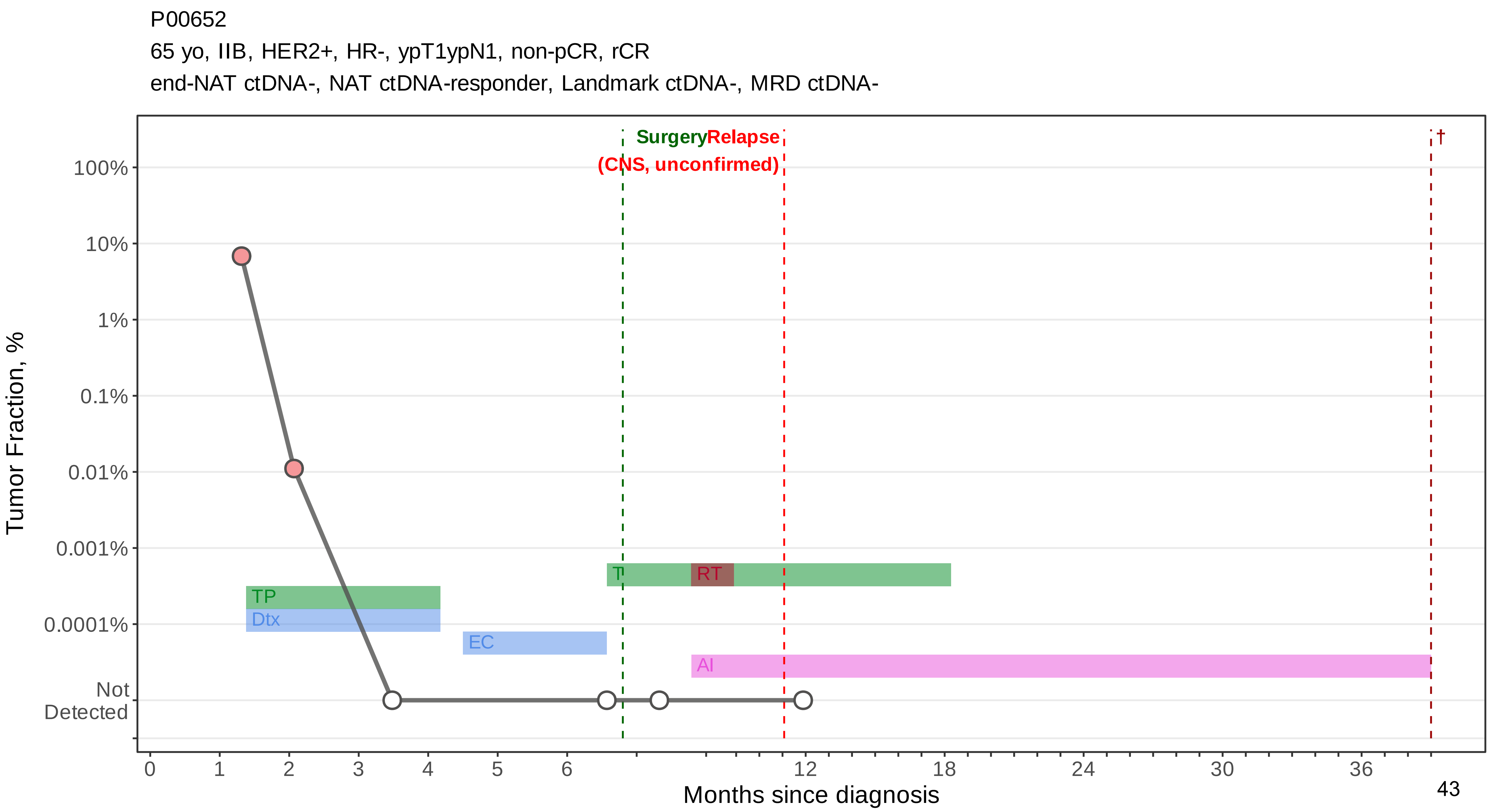

P00652
65 yo, IIB, HER2+, HR-, ypT1ypN1, non-pCR, rCR
end-NAT ctDNA-, NAT ctDNA-responder, Landmark ctDNA-, MRD ctDNA-
Surgery
Relapse
(CNS, unconfirmed)
†
100%
10%
1%
0.1%
0.01%
0.001%
0.0001%
Not Detected
Tumor Fraction, %
TP
Dtx
EC
T
RT
AI
0
1
2
3
4
5
6
12
18
24
30
36
Months since diagnosis

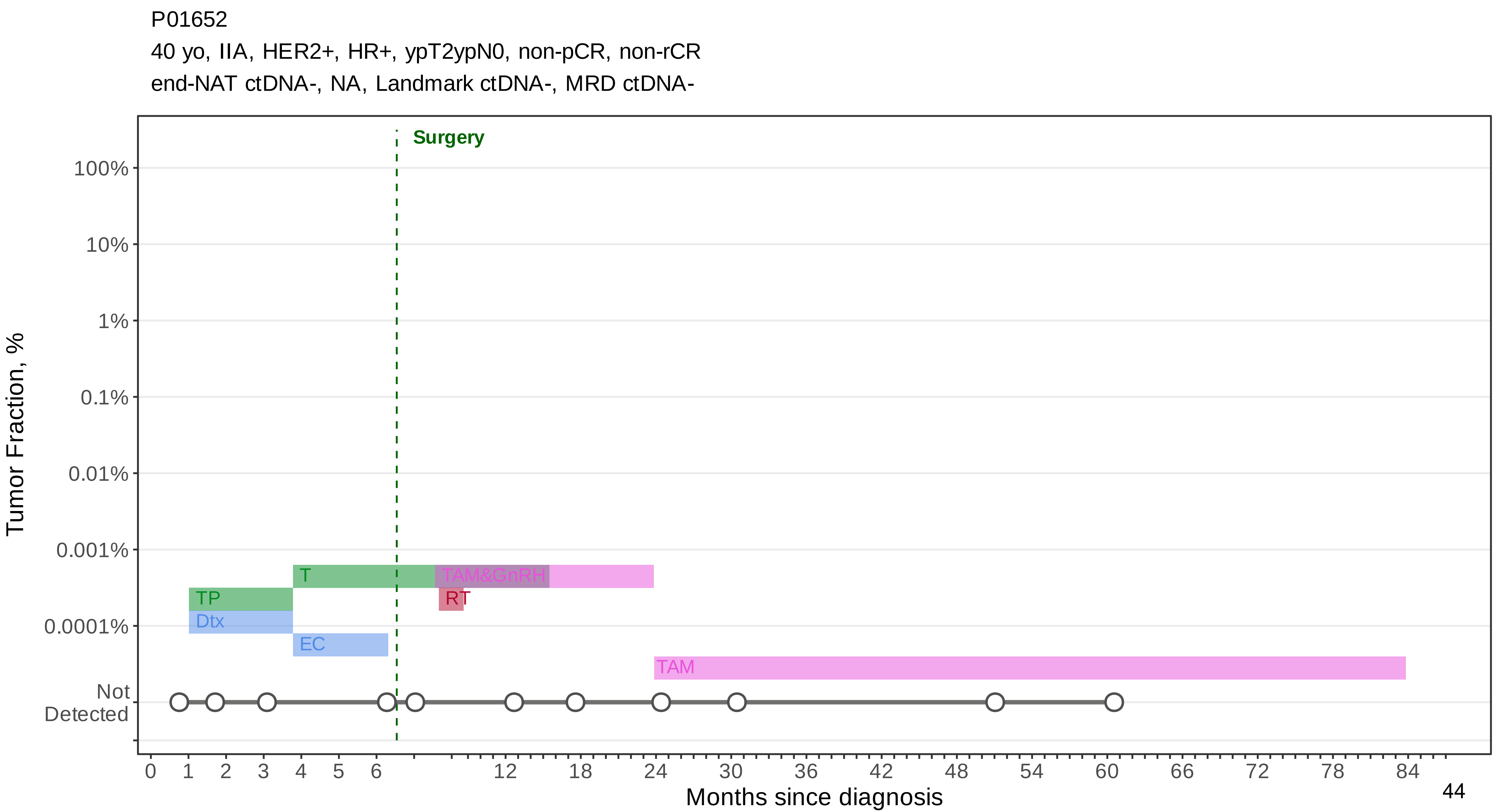
P01652
40 yo, IIA, HER2+, HR+, ypT2ypN0, non-pCR, non-rCR
end-NAT ctDNA-, NA, Landmark ctDNA-, MRD ctDNA-
Surgery
Tumor Fraction, %
100%
10%
1%
0.1%
0.01%
0.001%
0.0001%
Not Detected
T
TAM&GnRH
TP
RT
Dtx
EC
TAM
0 1 2 3 4 5 6 12 18 24 30 36 42 48 54 60 66 72 78 84
Months since diagnosis

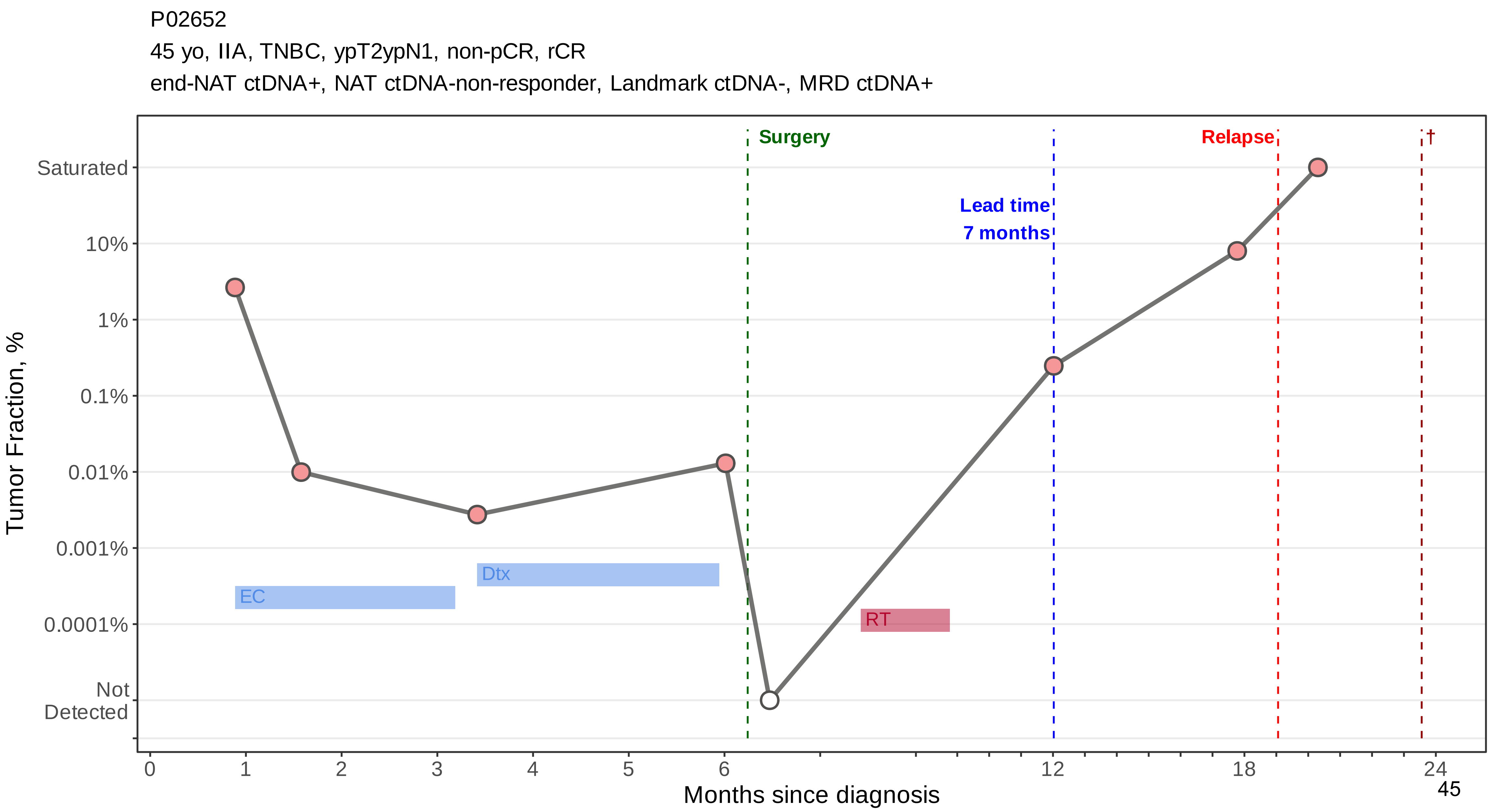
P02652
45 yo, IIA, TNBC, ypT2ypN1, non-pCR, rCR
end-NAT ctDNA+, NAT ctDNA-non-responder, Landmark ctDNA-, MRD ctDNA+
Surgery
Relapse
†
Lead time
7 months
Saturated
10%
1%
0.1%
0.01%
0.001%
0.0001%
Not Detected
Tumor Fraction, %
EC
Dtx
RT
0
1
2
3
4
5
6
12
18
24
Months since diagnosis

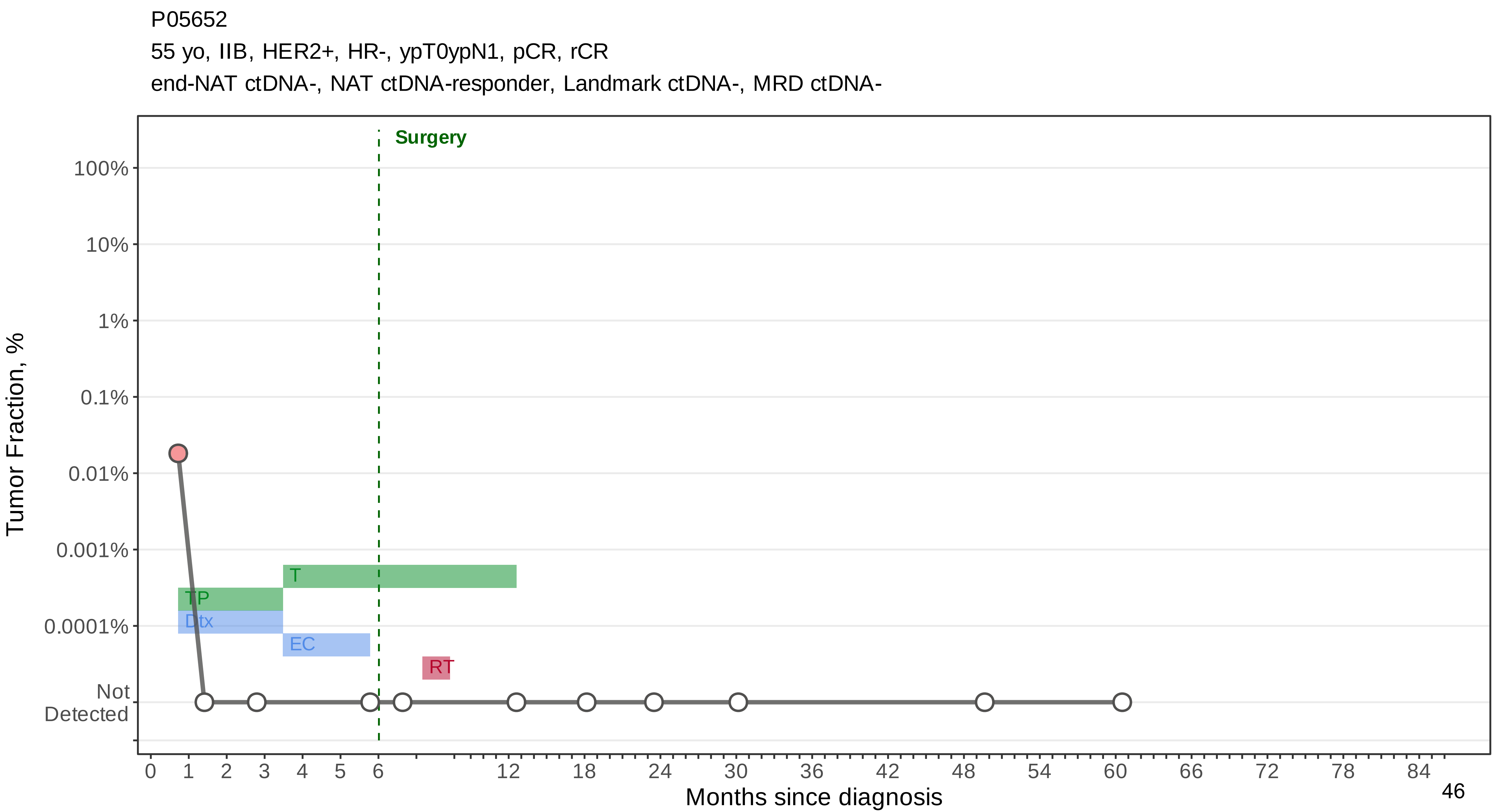
P05652
55 yo, IIB, HER2+, HR-, ypT0ypN1, pCR, rCR
end-NAT ctDNA-, NAT ctDNA-responder, Landmark ctDNA-, MRD ctDNA-
Surgery
Tumor Fraction, %
100%
10%
1%
0.1%
0.01%
0.001%
0.0001%
Not Detected
T
TP
Dtx
EC
RT
0
1
2
3
4
5
6
12
18
24
30
36
42
48
54
60
66
72
78
84
Months since diagnosis

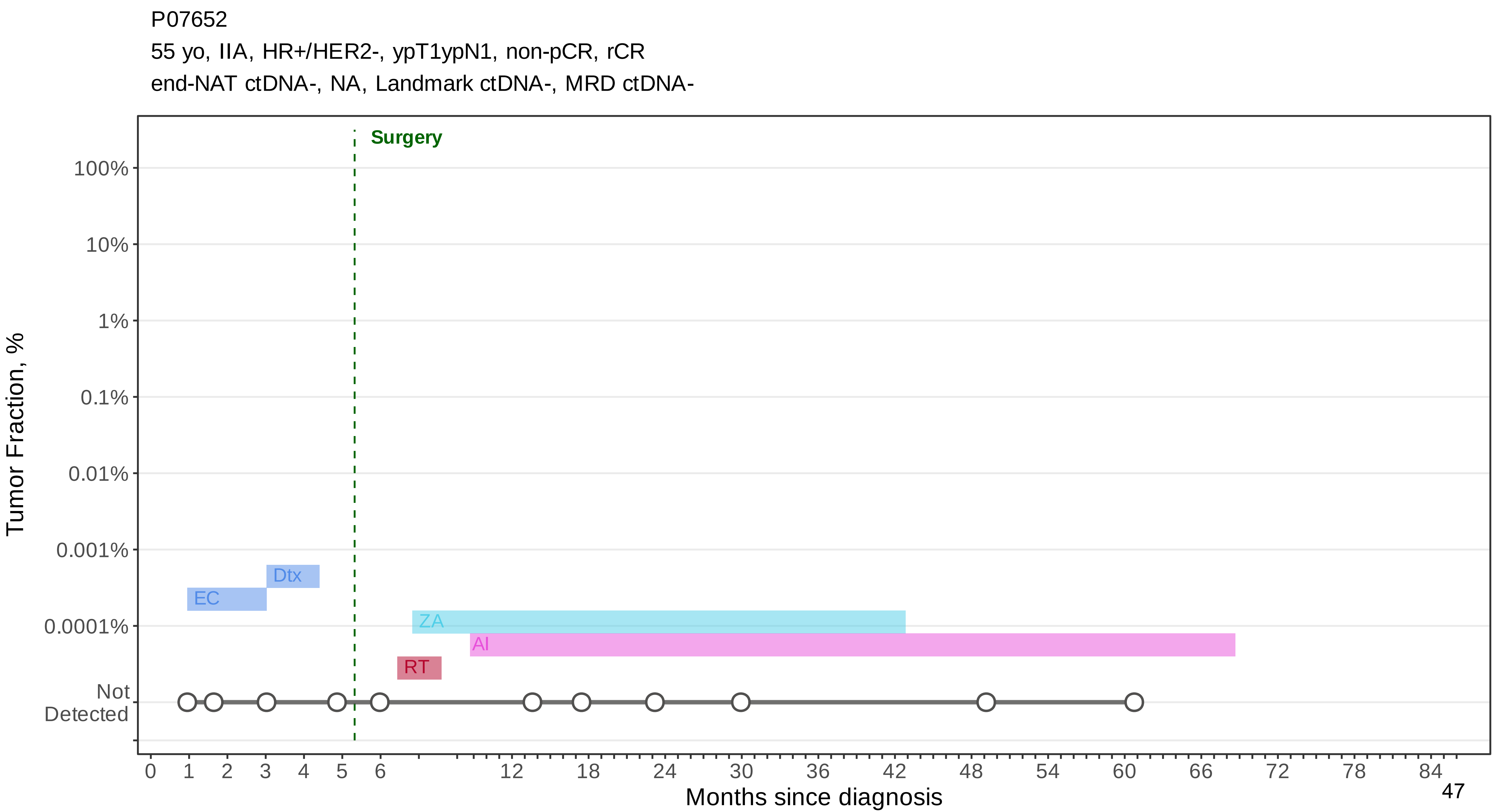
P07652
55 yo, IIA, HR+/HER2-, ypT1ypN1, non-pCR, rCR
end-NAT ctDNA-, NA, Landmark ctDNA-, MRD ctDNA-
Surgery
Tumor Fraction, %
100%
10%
1%
0.1%
0.01%
0.001%
0.0001%
Not Detected
Dtx
EC
ZA
AI
RT
0 1 2 3 4 5 6 12 18 24 30 36 42 48 54 60 66 72 78 84
Months since diagnosis

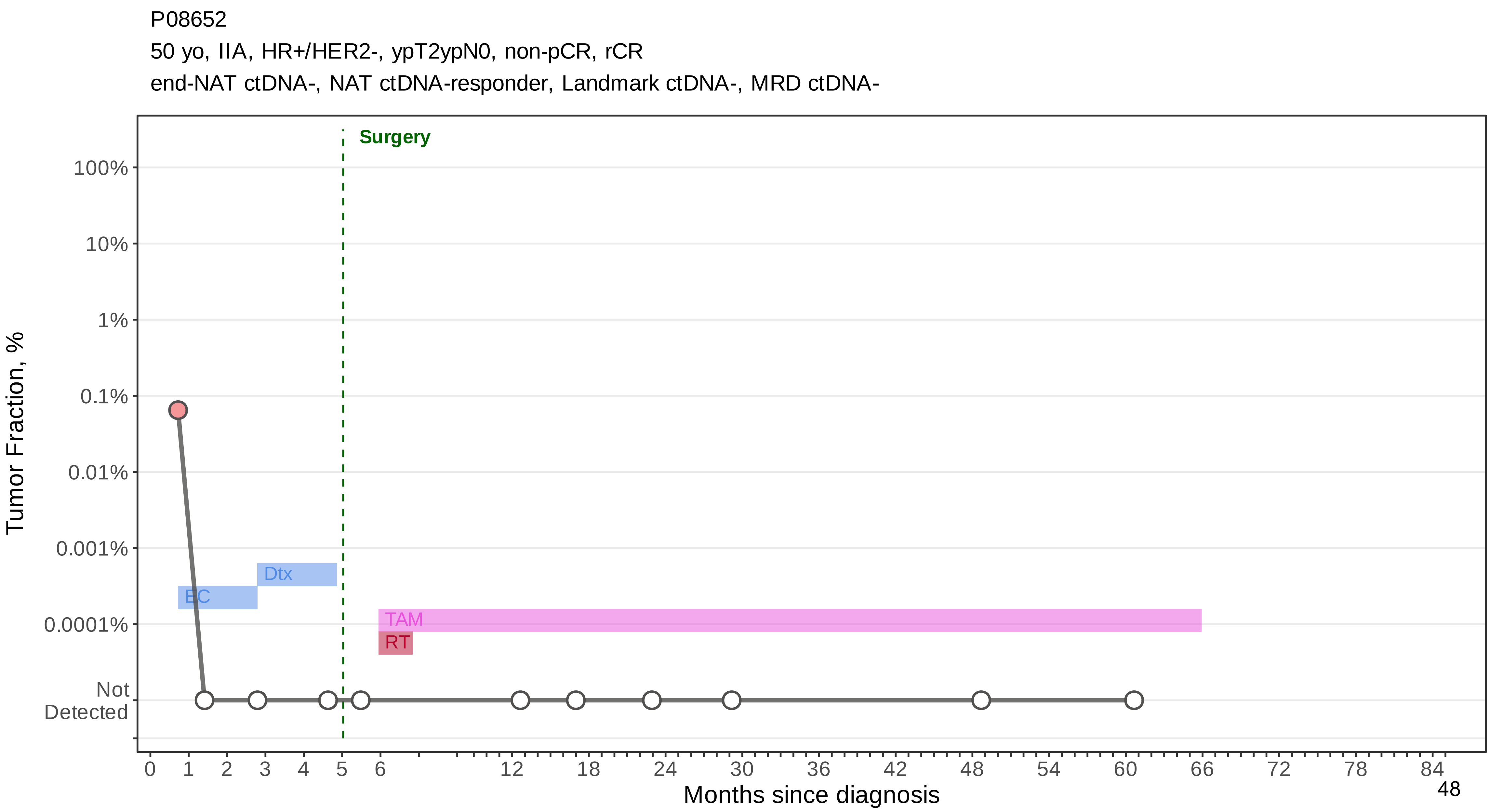
P08652
50 yo, IIA, HR+/HER2-, ypT2ypN0, non-pCR, rCR
end-NAT ctDNA-, NAT ctDNA-responder, Landmark ctDNA-, MRD ctDNA-
Surgery
100%
10%
1%
0.1%
0.01%
0.001%
0.0001%
Not Detected
Dtx
EC
TAM
RT
Tumor Fraction, %
0 1 2 3 4 5 6 12 18 24 30 36 42 48 54 60 66 72 78 84
Months since diagnosis

P09652
50 yo, IIB, HR+/HER2-, ypT1ypN1, non-pCR, non-rCR
end-NAT ctDNA-, NAT ctDNA-responder, Landmark ctDNA-, MRD ctDNA-

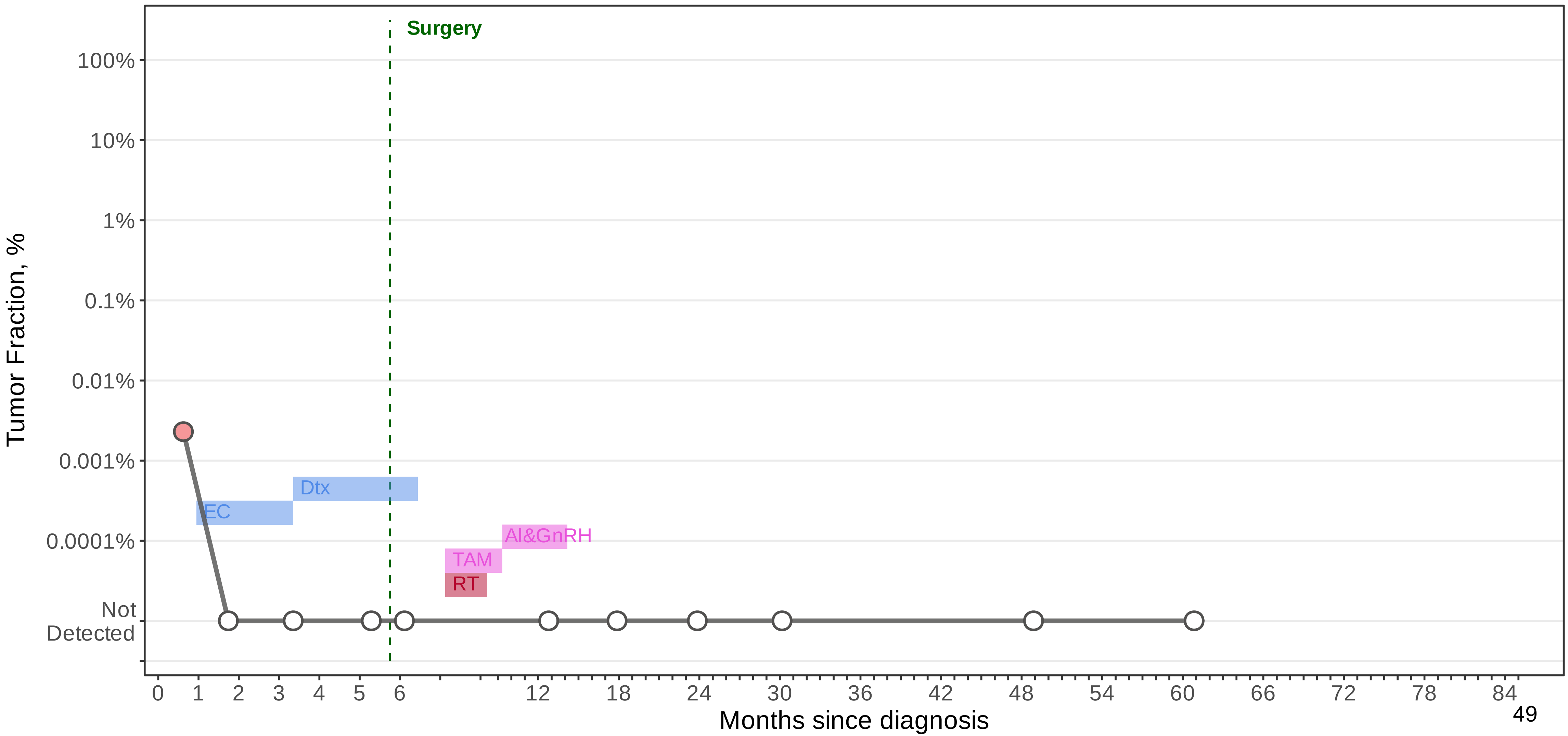

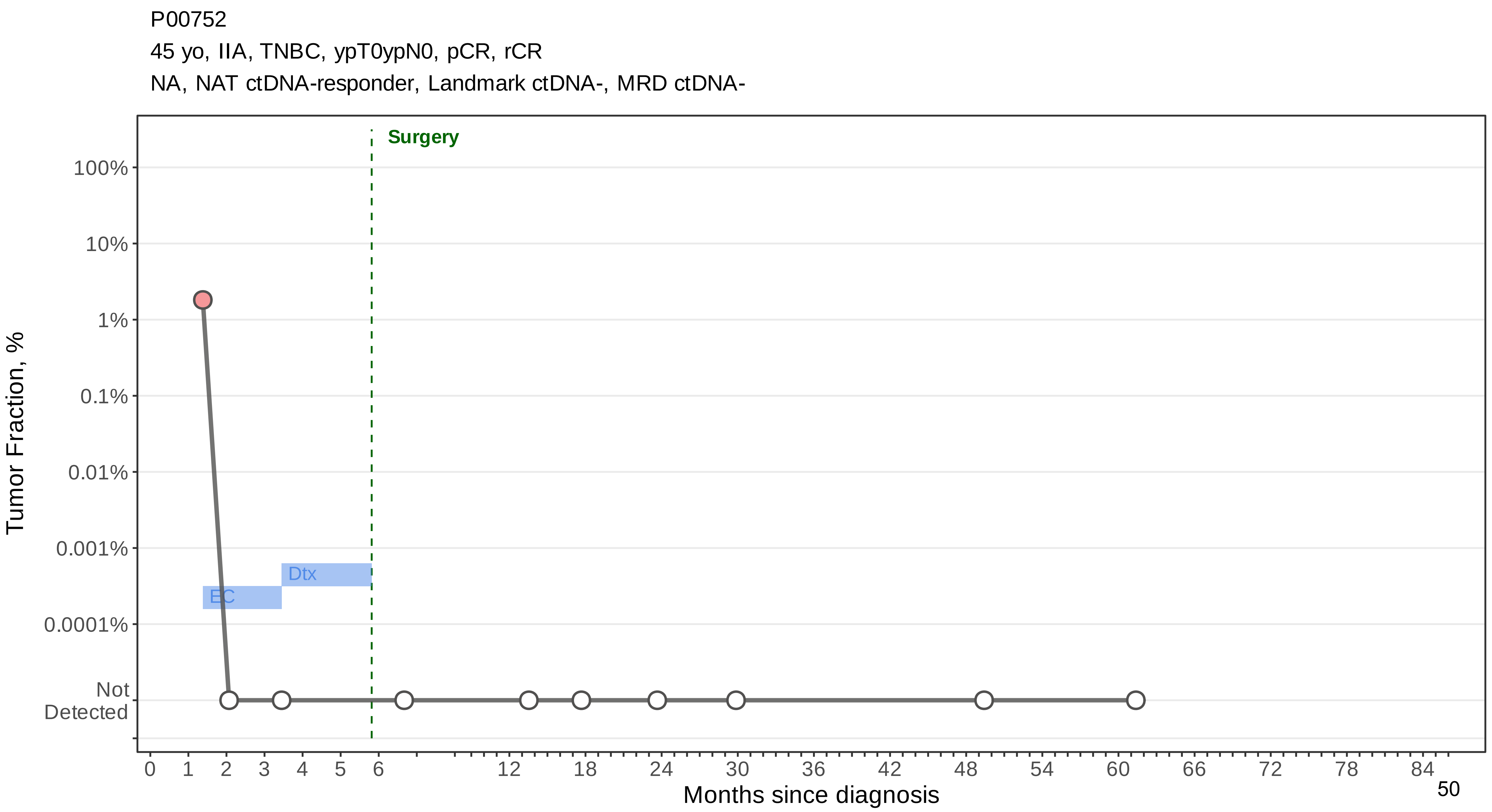

P00752
45 yo, IIA, TNBC, ypT0ypN0, pCR, rCR
NA, NAT ctDNA-responder, Landmark ctDNA-, MRD ctDNA-
Surgery
100%
10%
1%
0.1%
0.01%
0.001%
0.0001%
Not Detected
Tumor Fraction, %
EC
Dtx
0 1 2 3 4 5 6 12 18 24 30 36 42 48 54 60 66 72 78 84
Months since diagnosis

P01752
45 yo, IIB, TNBC, ypT1ypN0, non-pCR, non-rCR
end-NAT ctDNA-, NAT ctDNA-responder, Landmark ctDNA-, MRD ctDNA-

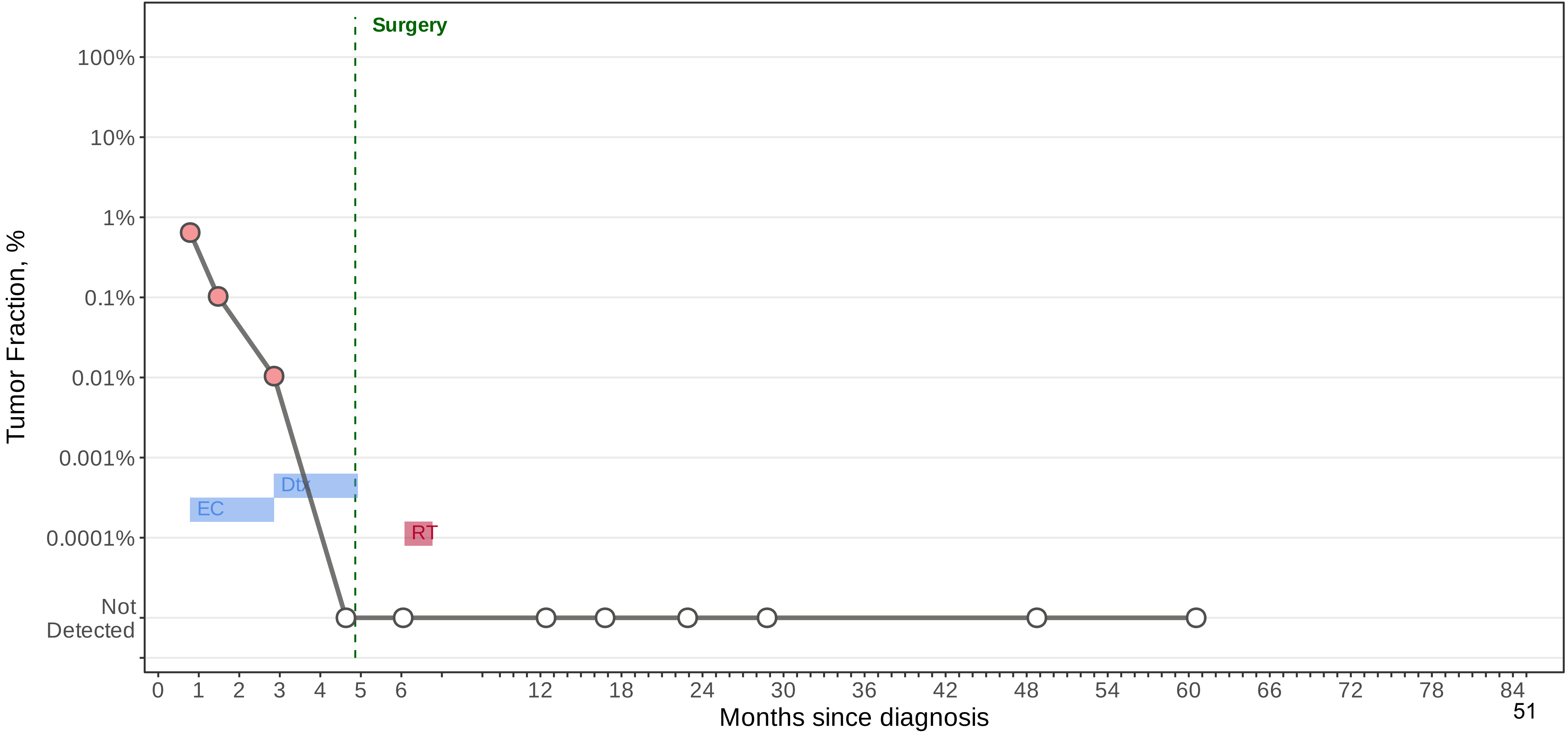

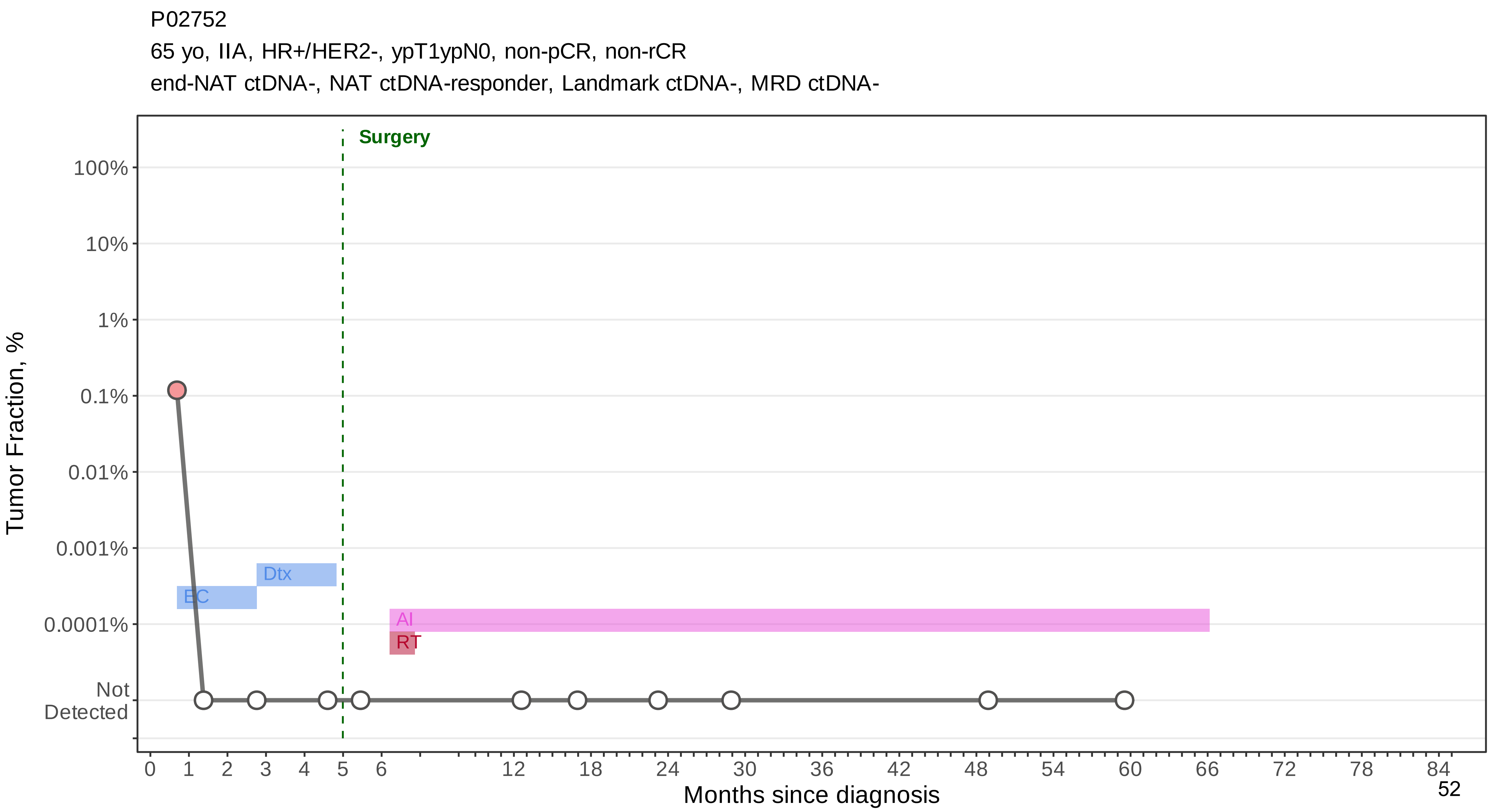
P02752
65 yo, IIA, HR+/HER2-, ypT1ypN0, non-pCR, non-rCR
end-NAT ctDNA-, NAT ctDNA-responder, Landmark ctDNA-, MRD ctDNA-
Surgery
100%
10%
1%
0.1%
0.01%
0.001%
0.0001%
Not Detected
Tumor Fraction, %
EC
Dtx
AI
RT
0 1 2 3 4 5 6 12 18 24 30 36 42 48 54 60 66 72 78 84
Months since diagnosis

P03752
75 yo, IIA, HER2+, HR+, ypT1ypN0, non-pCR, non-rCR
end-NAT ctDNA-, NAT ctDNA-responder, NA, MRD ctDNA-

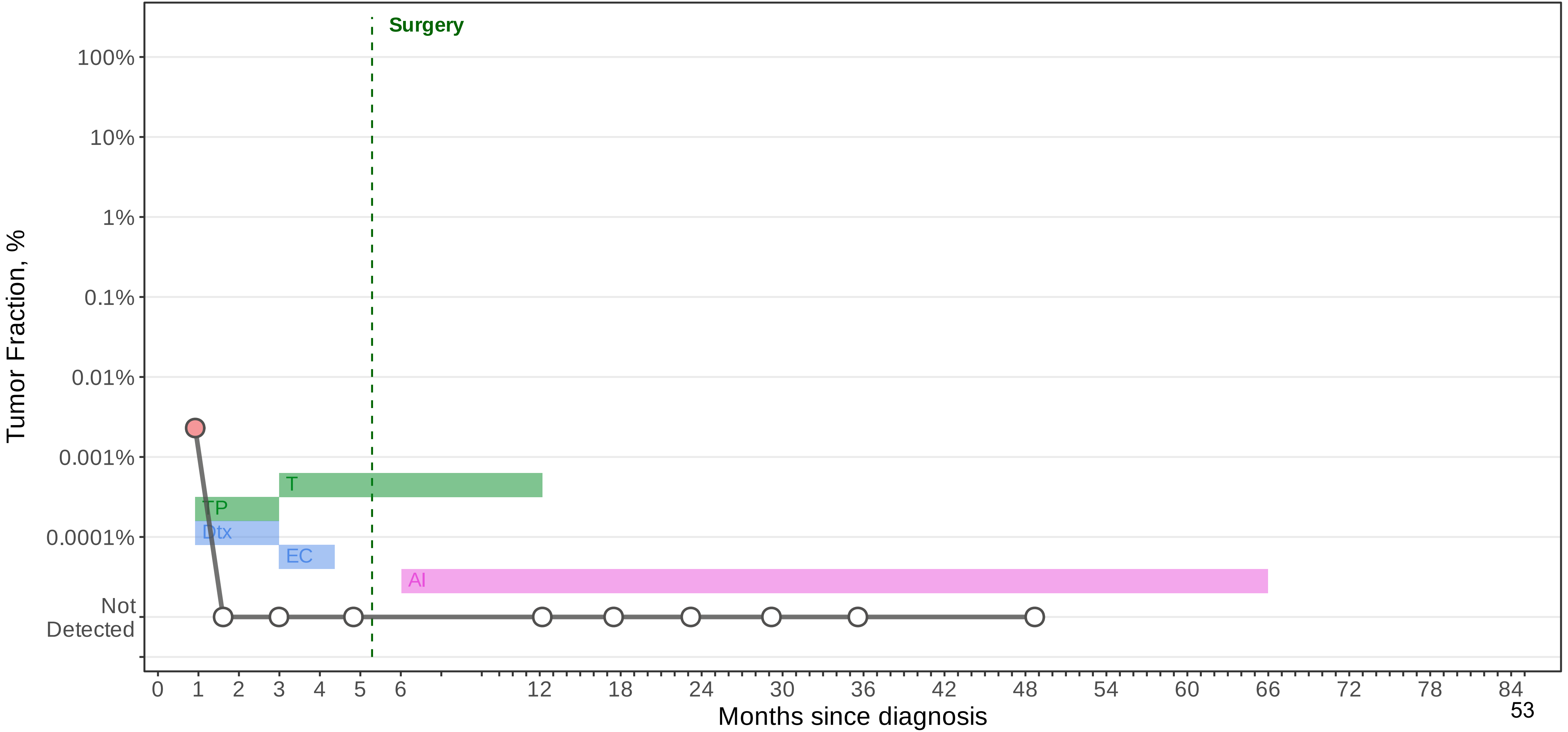

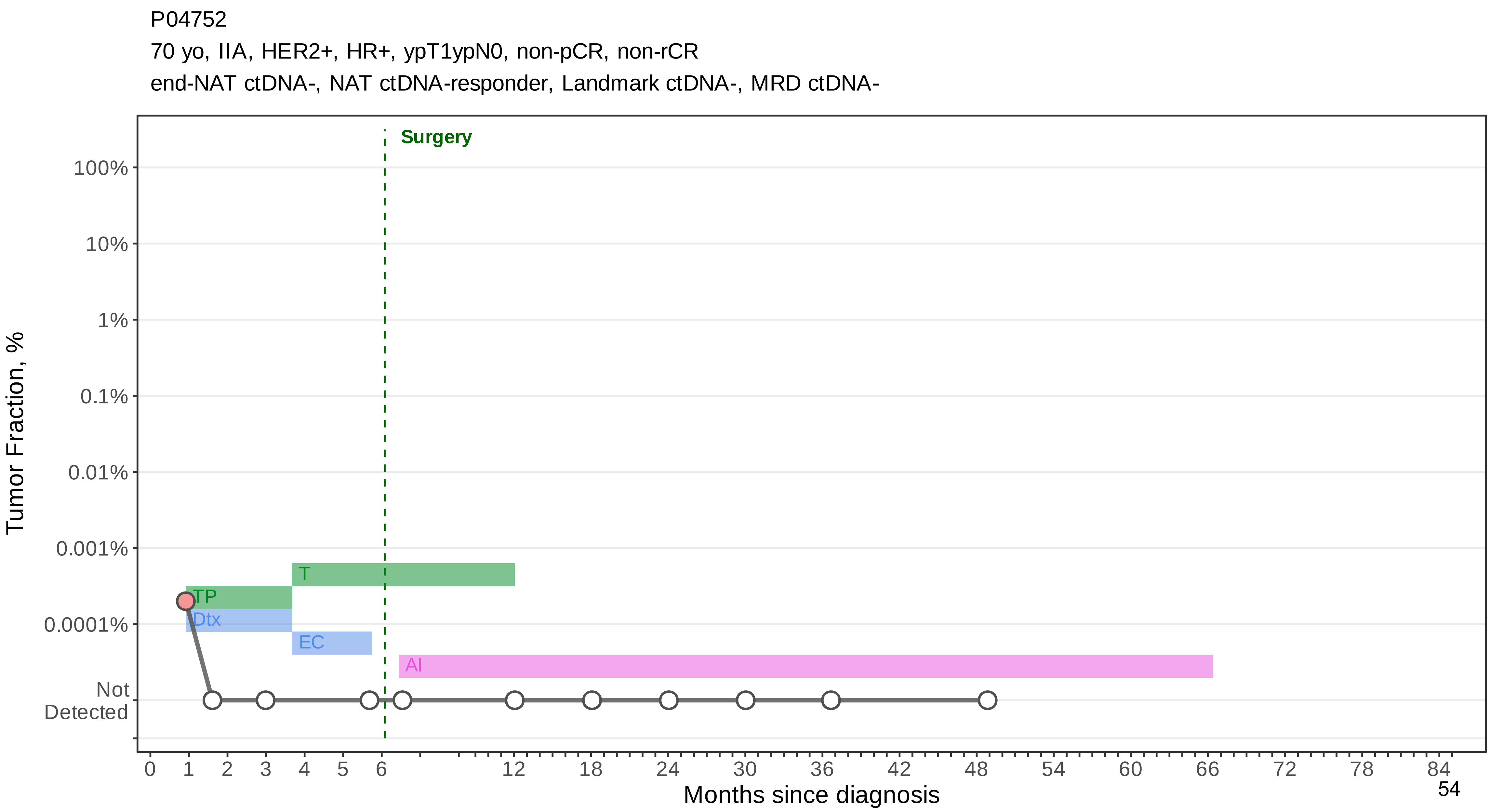
P04752
70 yo, IIA, HER2+, HR+, ypT1ypN0, non-pCR, non-rCR
end-NAT ctDNA-, NAT ctDNA-responder, Landmark ctDNA-, MRD ctDNA-
Surgery
100%
10%
1%
0.1%
0.01%
0.001%
0.0001%
Not Detected
Tumor Fraction, %
T
TP
Dtx
EC
AI
0
1
2
3
4
5
6
12
18
24
30
36
42
48
54
60
66
72
78
84
Months since diagnosis

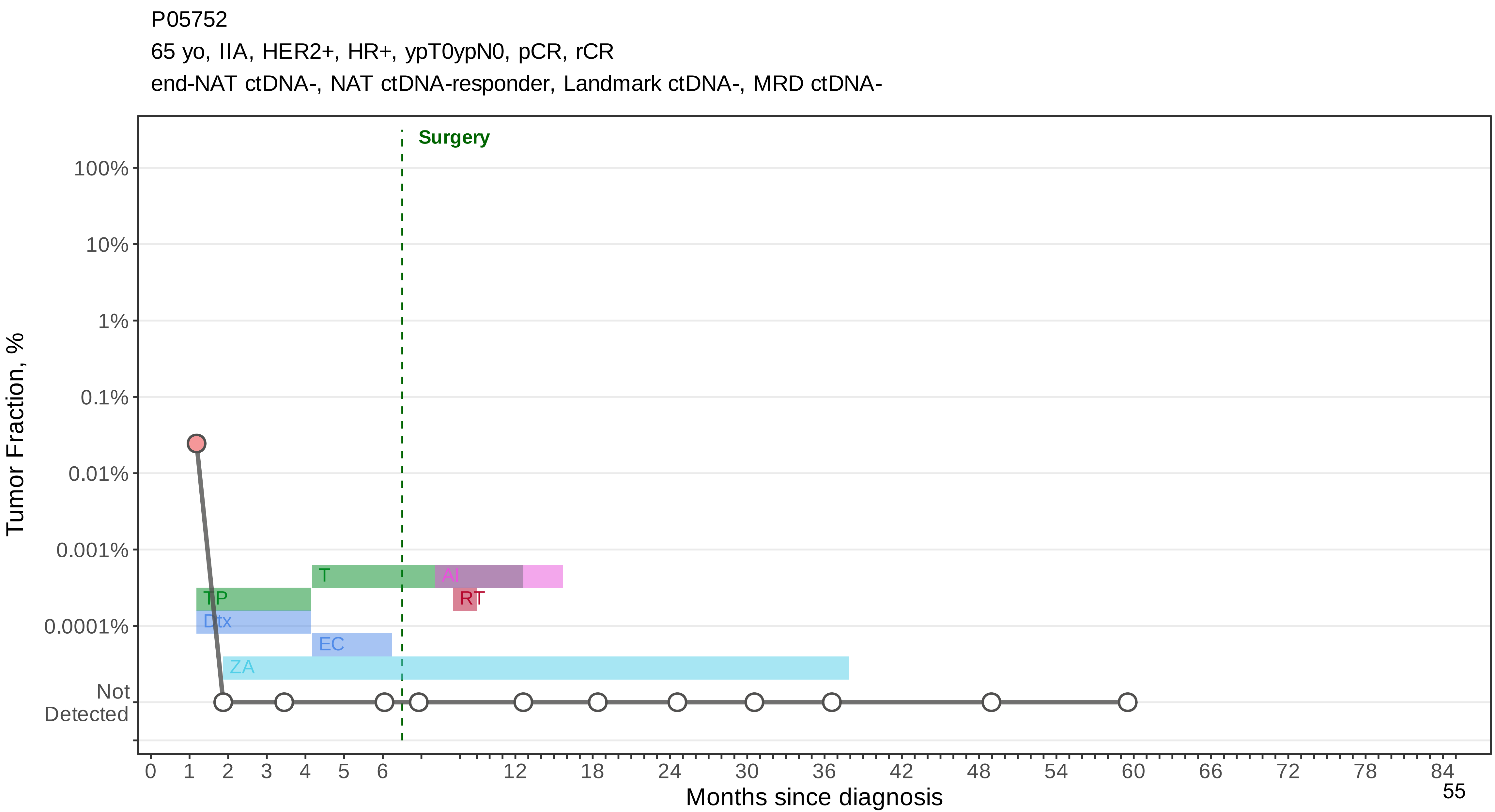
P05752
65 yo, IIA, HER2+, HR+, ypT0ypN0, pCR, rCR
end-NAT ctDNA-, NAT ctDNA-responder, Landmark ctDNA-, MRD ctDNA-
Surgery
100%
10%
1%
0.1%
0.01%
0.001%
0.0001%
Not Detected
Tumor Fraction, %
T
AI
TP
RT
Dtx
EC
ZA
0 1 2 3 4 5 6 12 18 24 30 36 42 48 54 60 66 72 78 84
Months since diagnosis

P07762
65 yo, IIA, HER2+, HR+, ypT1ypN1, non-pCR, non-rCR
end-NAT ctDNA-, NA, Landmark ctDNA-, MRD ctDNA-

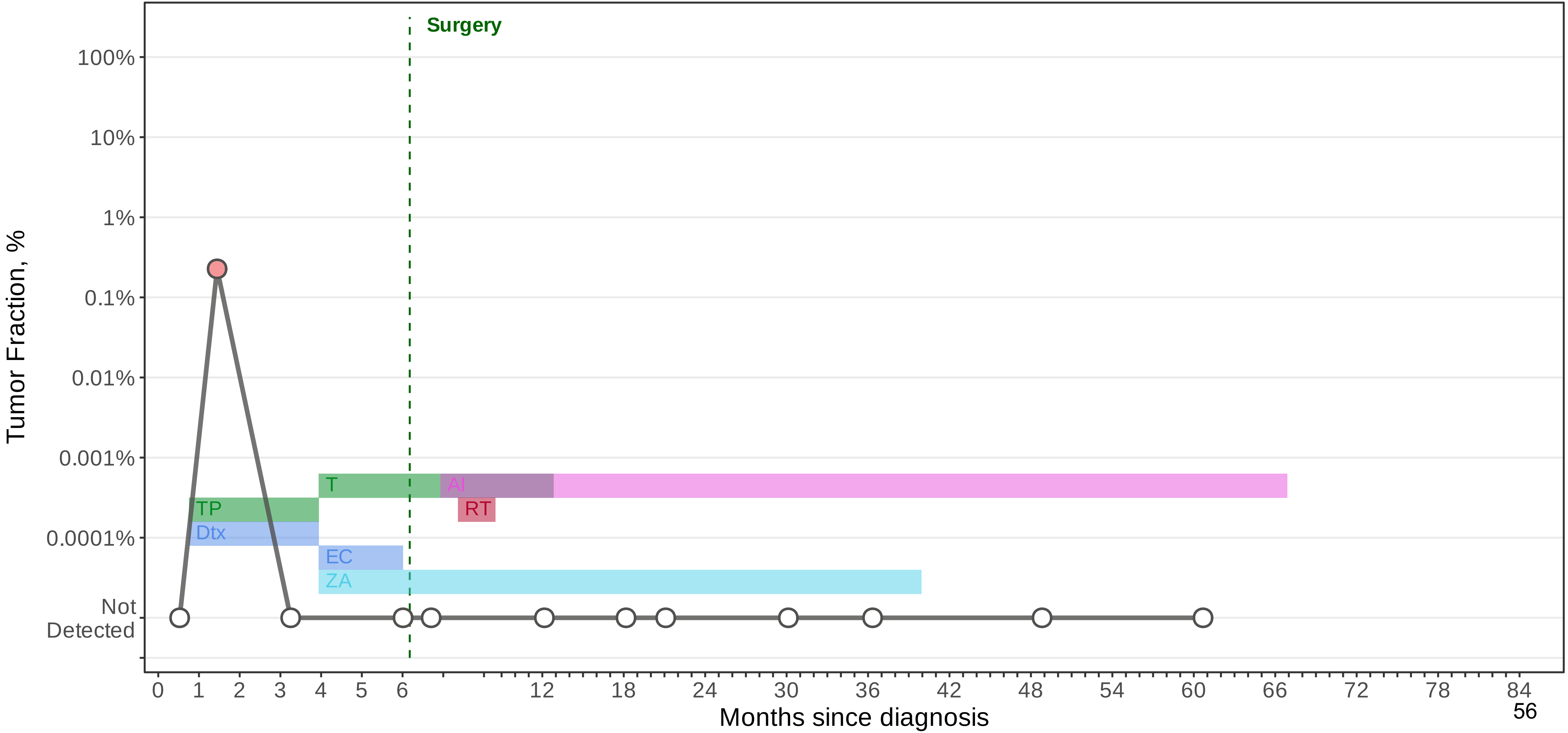

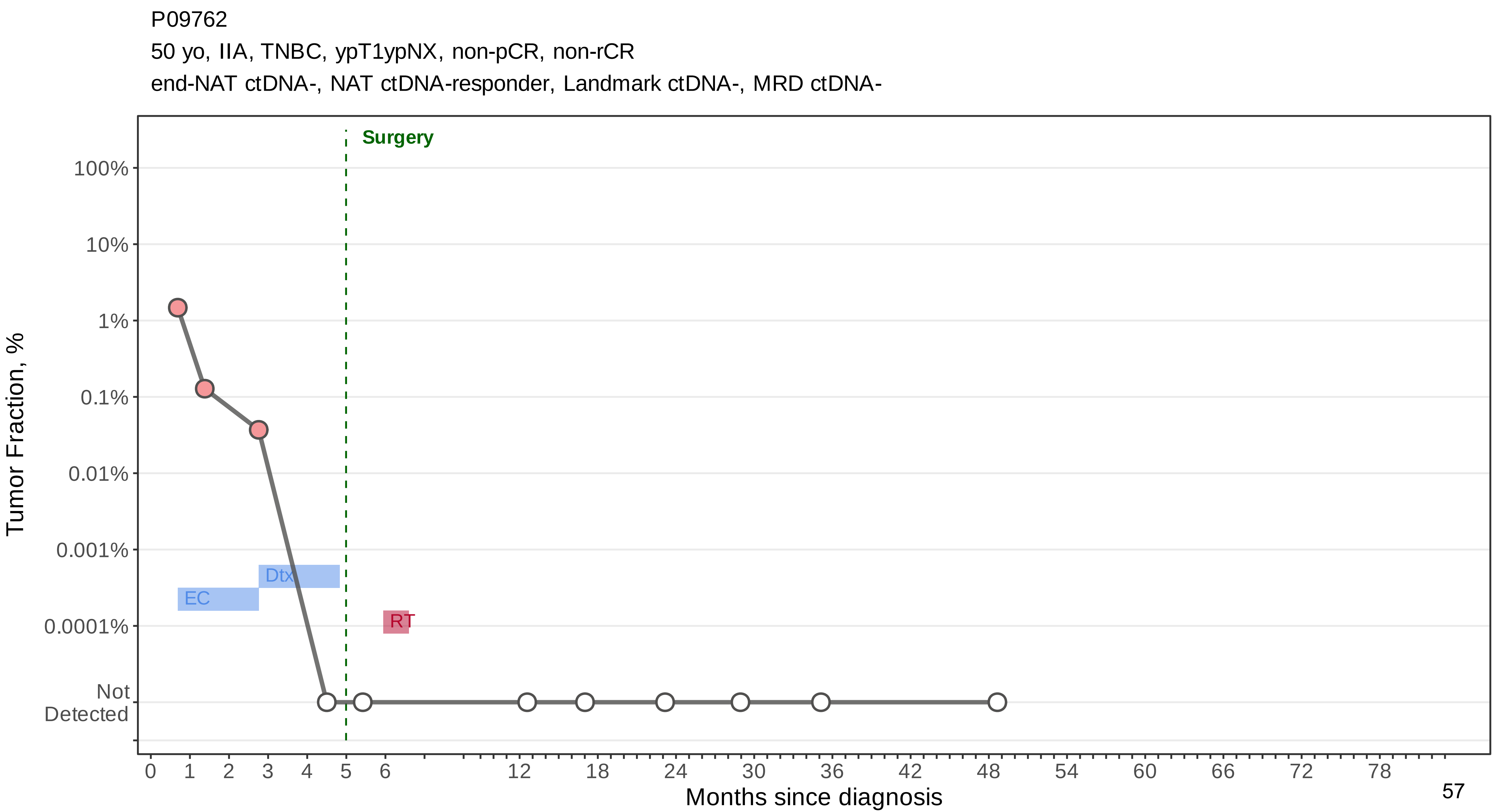

P09762
50 yo, IIA, TNBC, ypT1ypNX, non-pCR, non-rCR
end-NAT ctDNA-, NAT ctDNA-responder, Landmark ctDNA-, MRD ctDNA-
Surgery
100%
10%
1%
0.1%
0.01%
0.001%
0.0001%
Not Detected
Tumor Fraction, %
Dtx
EC
RT
0 1 2 3 4 5 6 12 18 24 30 36 42 48 54 60 66 72 78
Months since diagnosis

P00862
50 yo, IIA, HR+/HER2-, ypT1ypN0, non-pCR, non-rCR
end-NAT ctDNA-, NA, Landmark ctDNA-, MRD ctDNA-

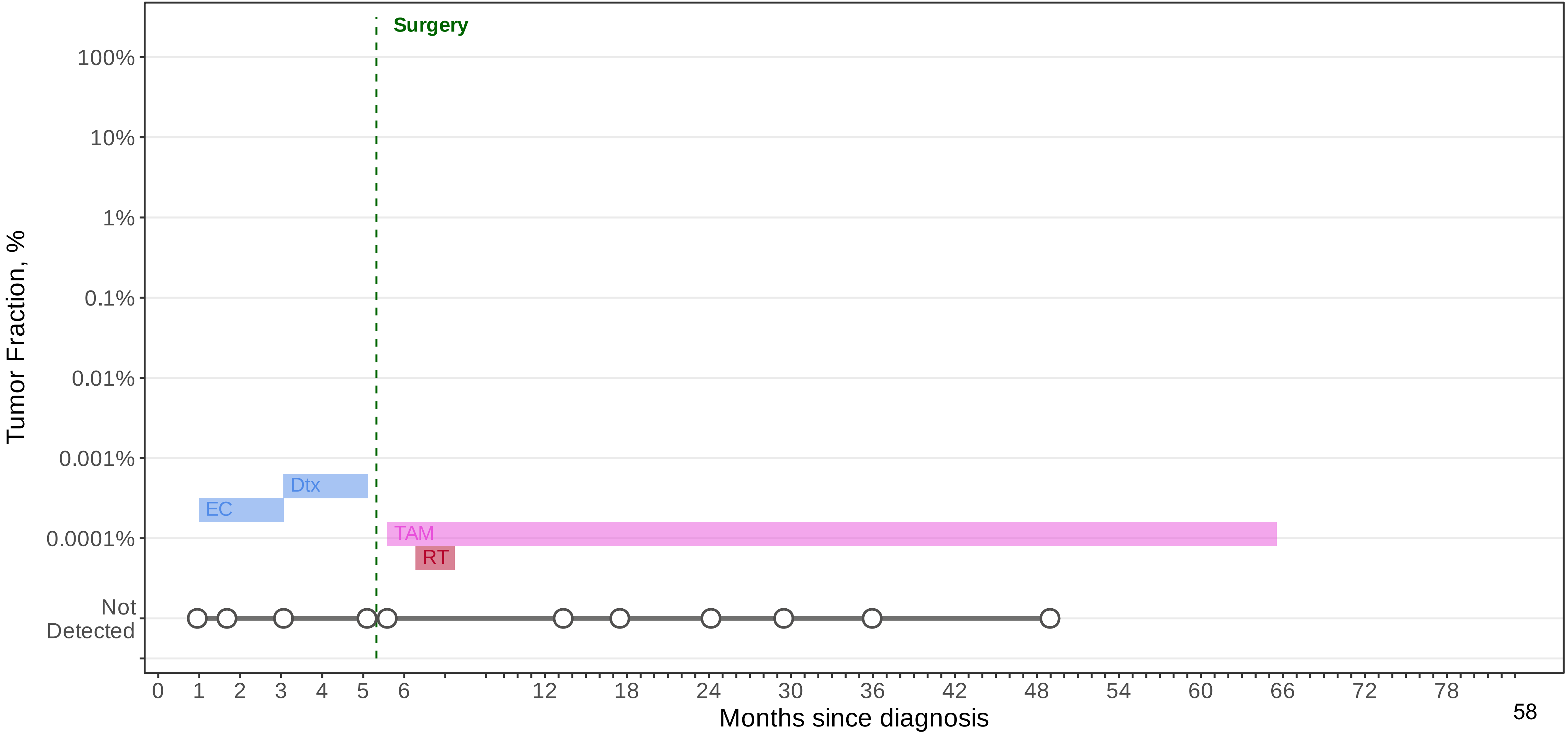

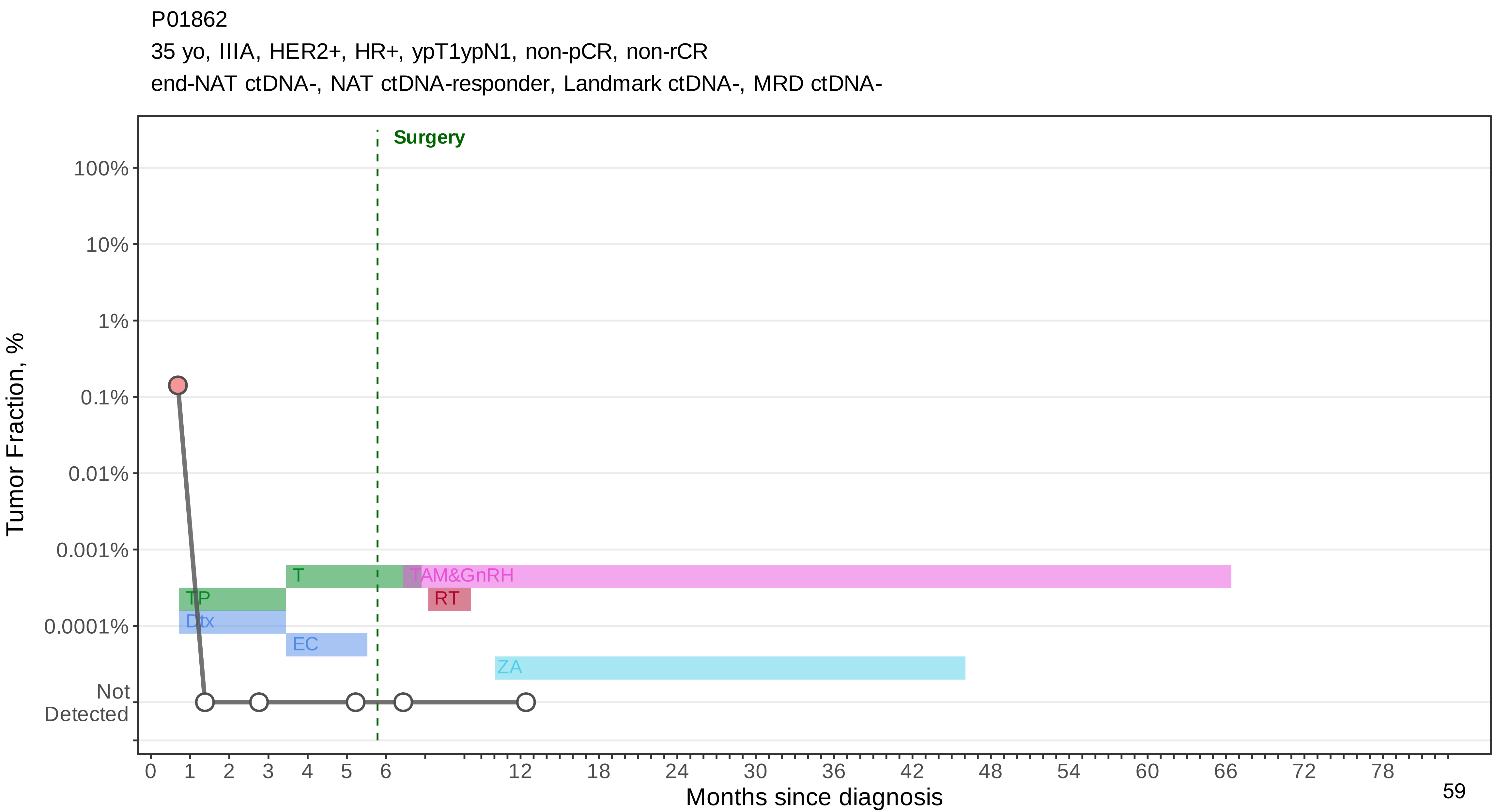
P01862
35 yo, IIIA, HER2+, HR+, ypT1ypN1, non-pCR, non-rCR
end-NAT ctDNA-, NAT ctDNA-responder, Landmark ctDNA-, MRD ctDNA-
Surgery
100%
10%
1%
0.1%
0.01%
0.001%
0.0001%
Not Detected
Tumor Fraction, %
T
TAM&GnRH
TP
RT
Dtx
EC
ZA
0 1 2 3 4 5 6 12 18 24 30 36 42 48 54 60 66 72 78
Months since diagnosis

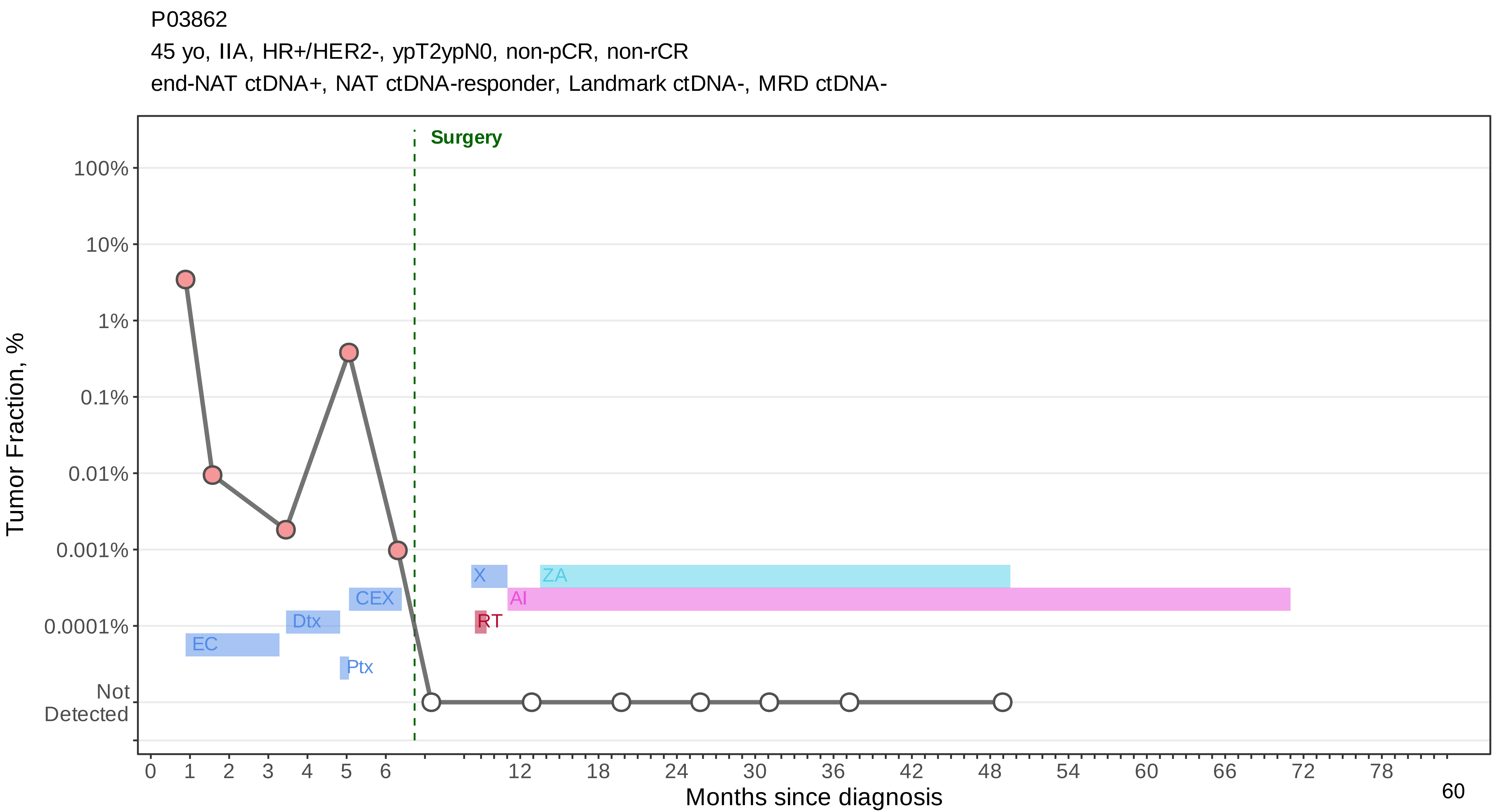
P03862
45 yo, IIA, HR+/HER2-, ypT2ypN0, non-pCR, non-rCR
end-NAT ctDNA+, NAT ctDNA-responder, Landmark ctDNA-, MRD ctDNA-
Surgery
Tumor Fraction, %
100%
10%
1%
0.1%
0.01%
0.001%
0.0001%
Not Detected
X
ZA
CEX
AI
Dtx
RT
EC
Ptx
0
1
2
3
4
5
6
12
18
24
30
36
42
48
54
60
66
72
78
Months since diagnosis

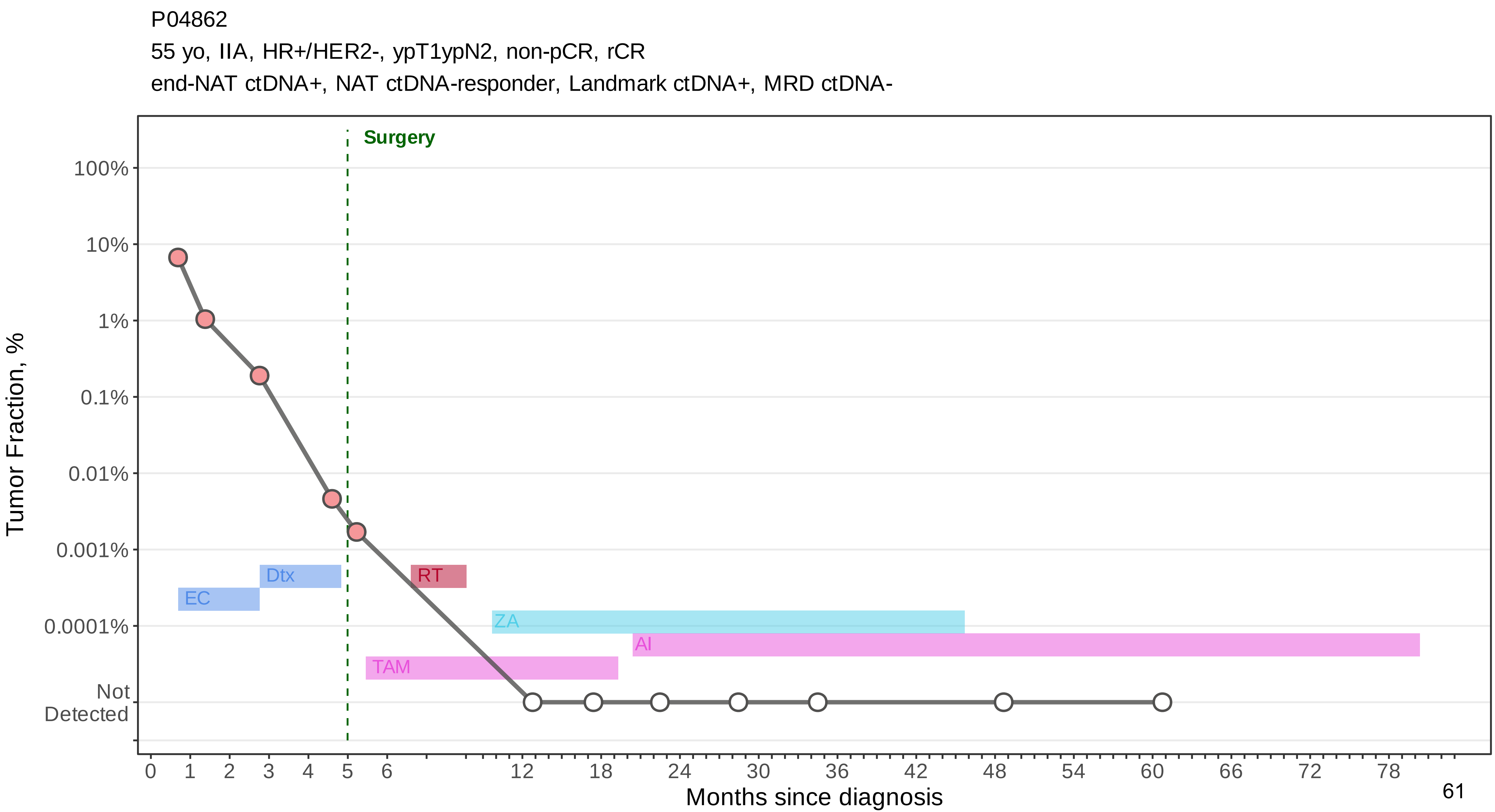

P04862
55 yo, IIA, HR+/HER2-, ypT1ypN2, non-pCR, rCR
end-NAT ctDNA+, NAT ctDNA-responder, Landmark ctDNA+, MRD ctDNA-
Surgery
100%
10%
1%
0.1%
0.01%
0.001%
0.0001%
Not Detected
Tumor Fraction, %
EC
Dtx
RT
ZA
AI
TAM
0 1 2 3 4 5 6 12 18 24 30 36 42 48 54 60 66 72 78
Months since diagnosis

P05862
45 yo, IA, HR+/HER2-, ypT1ypN0, non-pCR, non-rCR
end-NAT ctDNA+, NAT ctDNA-responder, Landmark ctDNA-, MRD ctDNA-

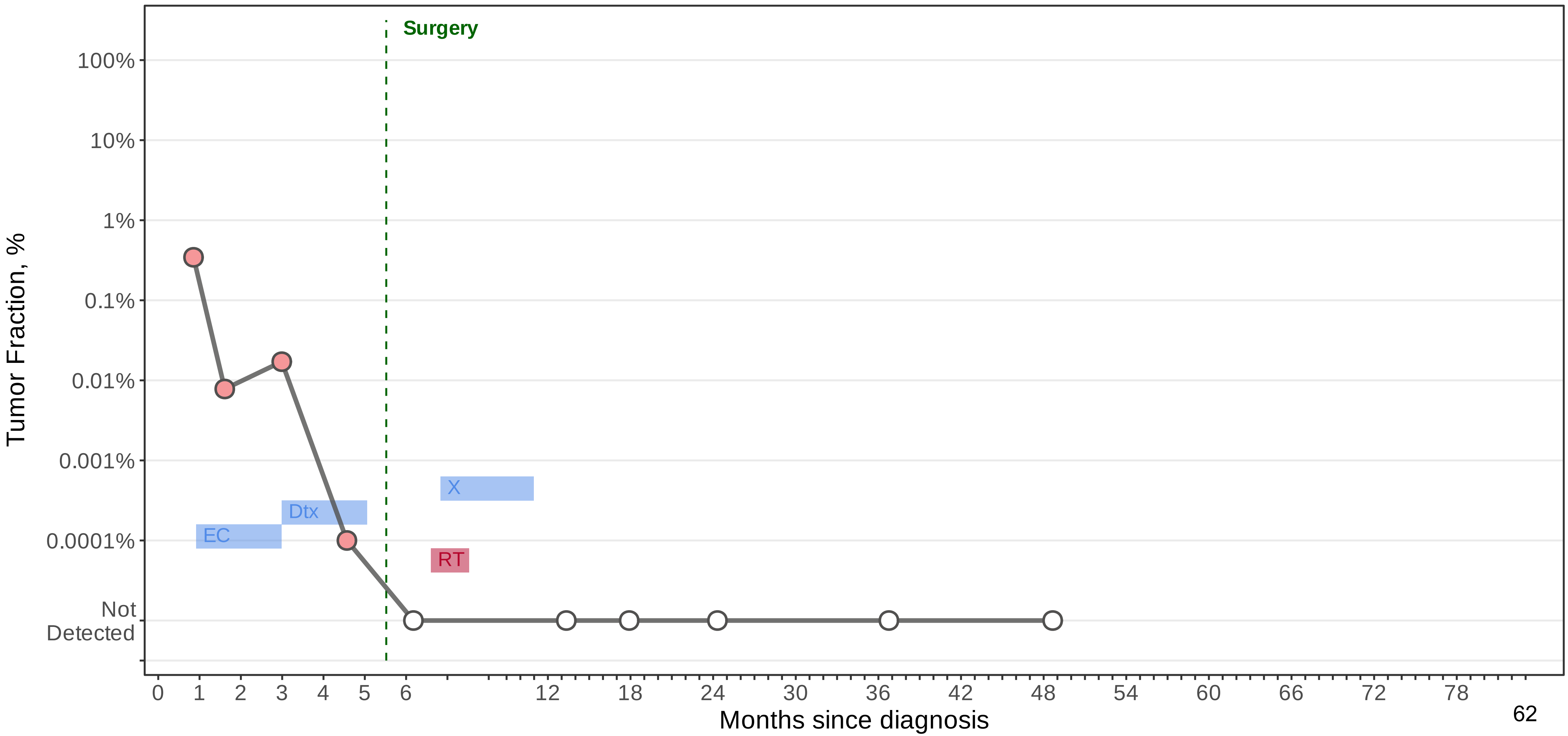

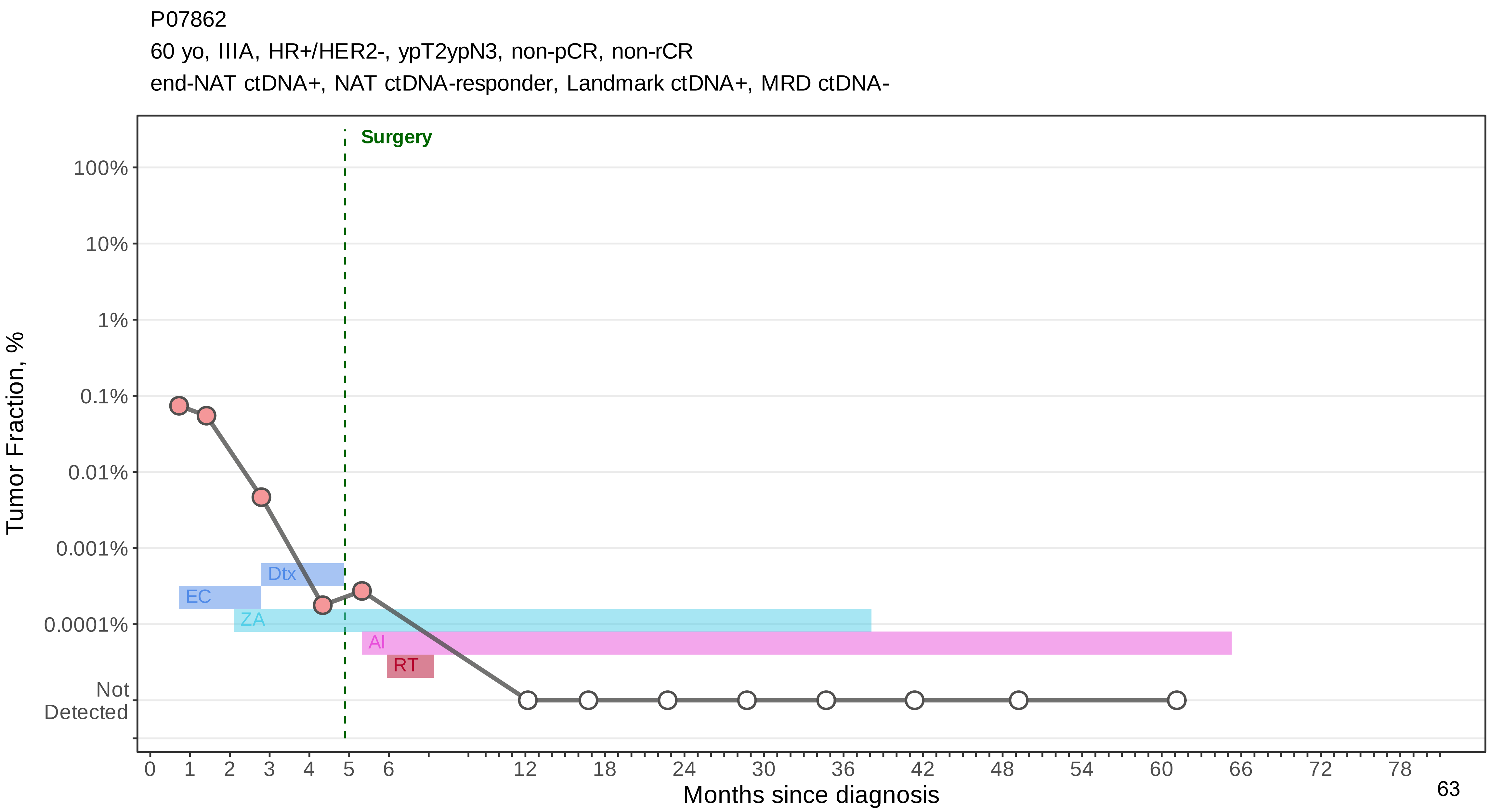
P07862
60 yo, IIIA, HR+/HER2-, ypT2ypN3, non-pCR, non-rCR
end-NAT ctDNA+, NAT ctDNA-responder, Landmark ctDNA+, MRD ctDNA-
Surgery
Tumor Fraction, %
100%
10%
1%
0.1%
0.01%
0.001%
0.0001%
Not Detected
Dtx
EC
ZA
AI
RT
0
1
2
3
4
5
6
12
18
24
30
36
42
48
54
60
66
72
78
Months since diagnosis

P08862

60 yo, IIA, HER2+, HR+, ypT1ypN1, NA, non-rCR

end-NAT ctDNA-, NAT ctDNA-responder, Landmark ctDNA-, MRD ctDNA-

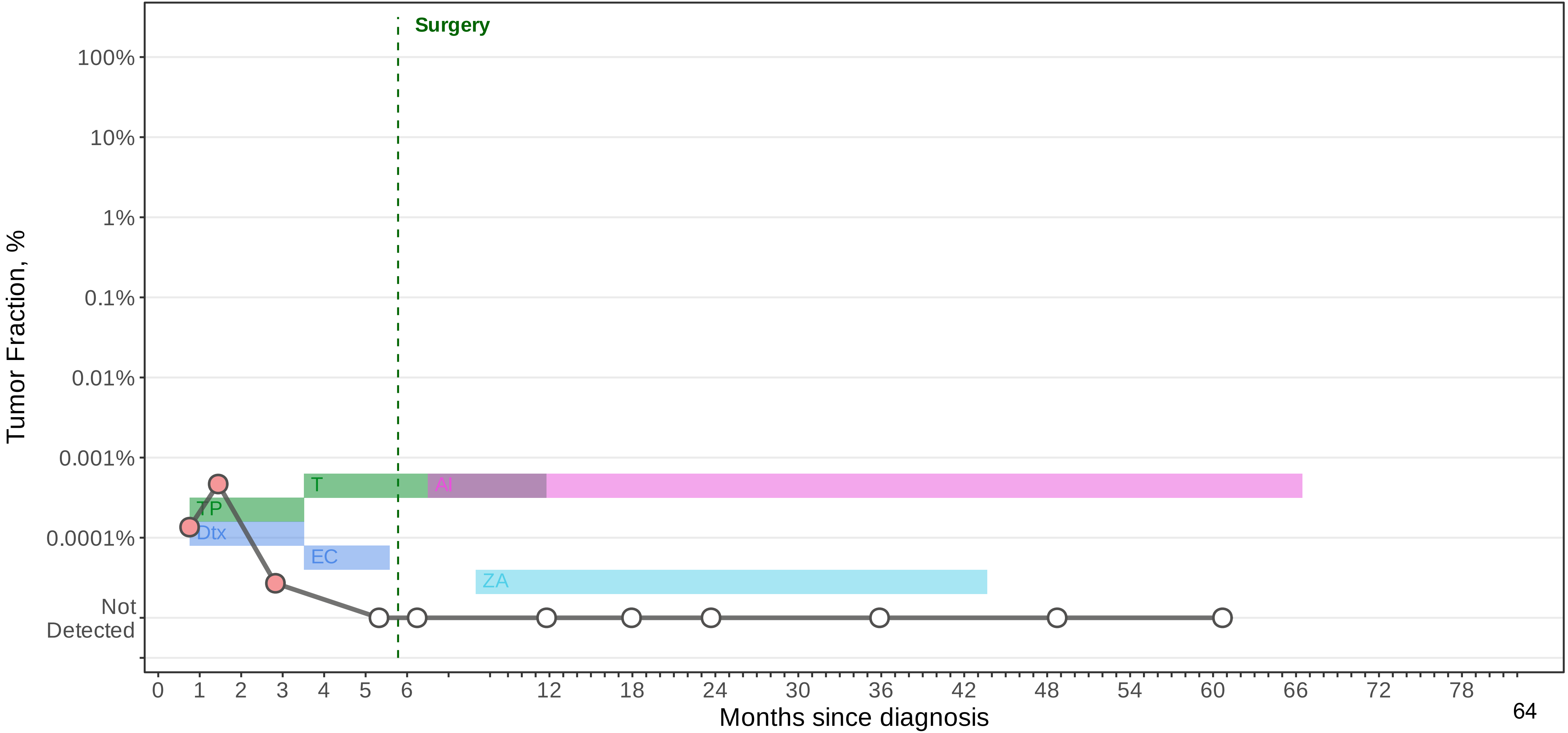

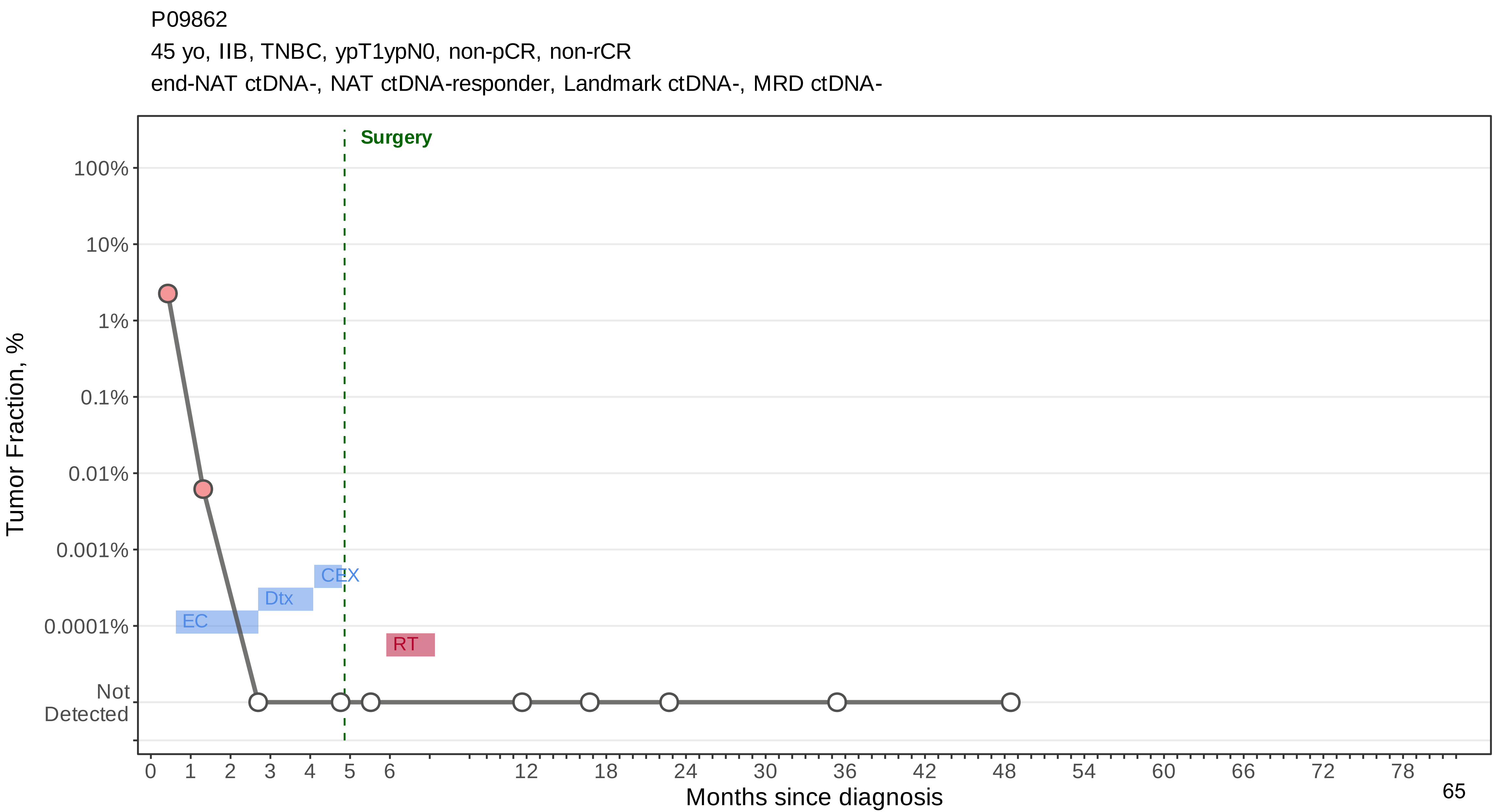
P09862
45 yo, IIB, TNBC, ypT1ypN0, non-pCR, non-rCR
end-NAT ctDNA-, NAT ctDNA-responder, Landmark ctDNA-, MRD ctDNA-
Surgery
100%
10%
1%
0.1%
0.01%
0.001%
0.0001%
Not Detected
Tumor Fraction, %
CEX
Dtx
EC
RT
0
1
2
3
4
5
6
12
18
24
30
36
42
48
54
60
66
72
78
Months since diagnosis

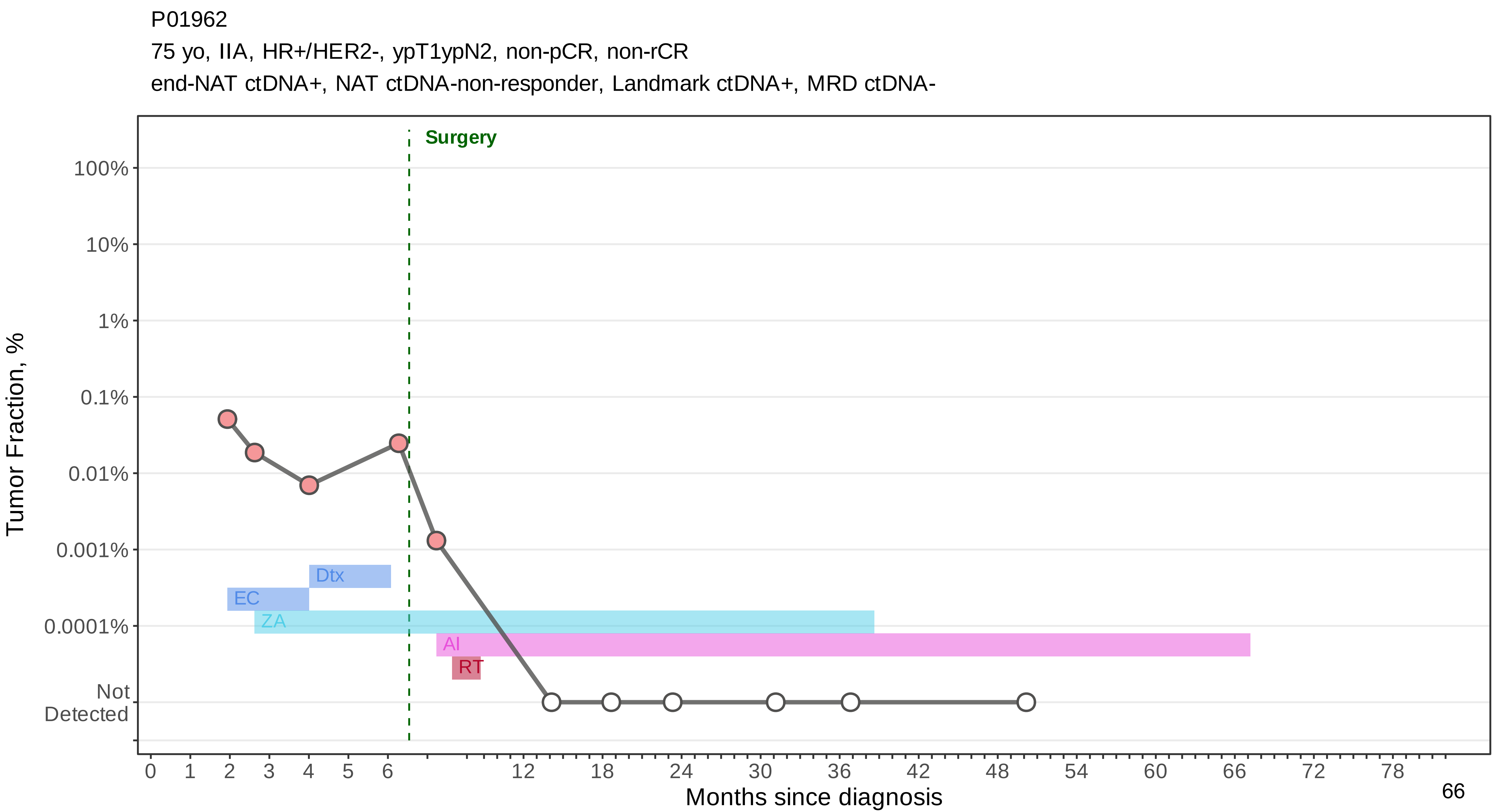
P01962
75 yo, IIA, HR+/HER2-, ypT1ypN2, non-pCR, non-rCR
end-NAT ctDNA+, NAT ctDNA-non-responder, Landmark ctDNA+, MRD ctDNA-
Surgery
100%
10%
1%
0.1%
0.01%
0.001%
0.0001%
Not Detected
Tumor Fraction, %
Dtx
EC
ZA
AI
RT
0
1
2
3
4
5
6
12
18
24
30
36
42
48
54
60
66
72
78
Months since diagnosis

P02962

35 yo, NA, HER2+, HR-, ypT1ypN0, non-pCR, rCR

end-NAT ctDNA-, NAT ctDNA-responder, Landmark ctDNA-, MRD ctDNA-

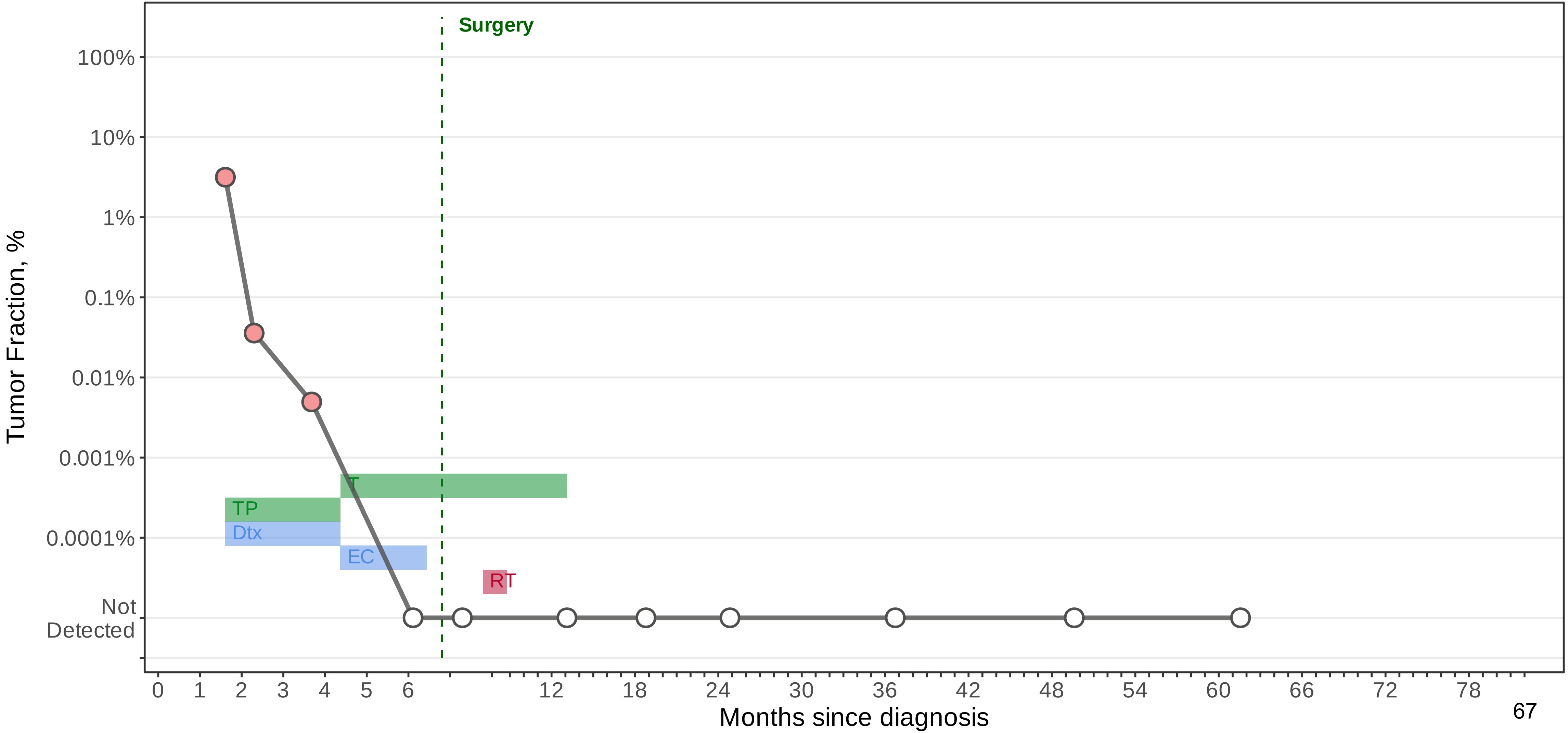

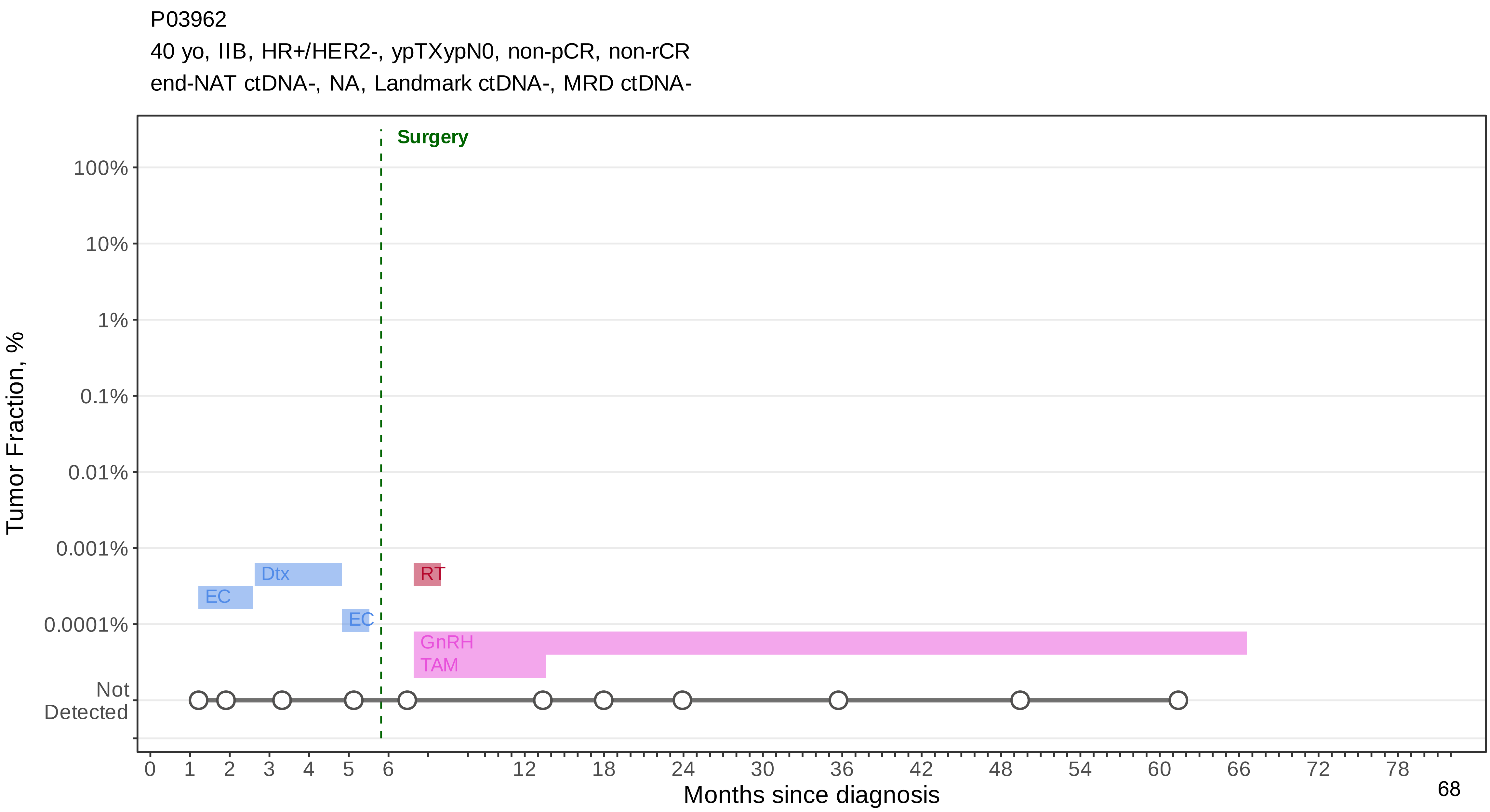
P03962
40 yo, IIB, HR+/HER2-, ypTXypN0, non-pCR, non-rCR
end-NAT ctDNA-, NA, Landmark ctDNA-, MRD ctDNA-
Surgery
100%
10%
1%
0.1%
0.01%
0.001%
0.0001%
Not Detected
Tumor Fraction, %
Dtx
EC
EC
RT
GnRH
TAM
0 1 2 3 4 5 6 12 18 24 30 36 42 48 54 60 66 72 78
Months since diagnosis

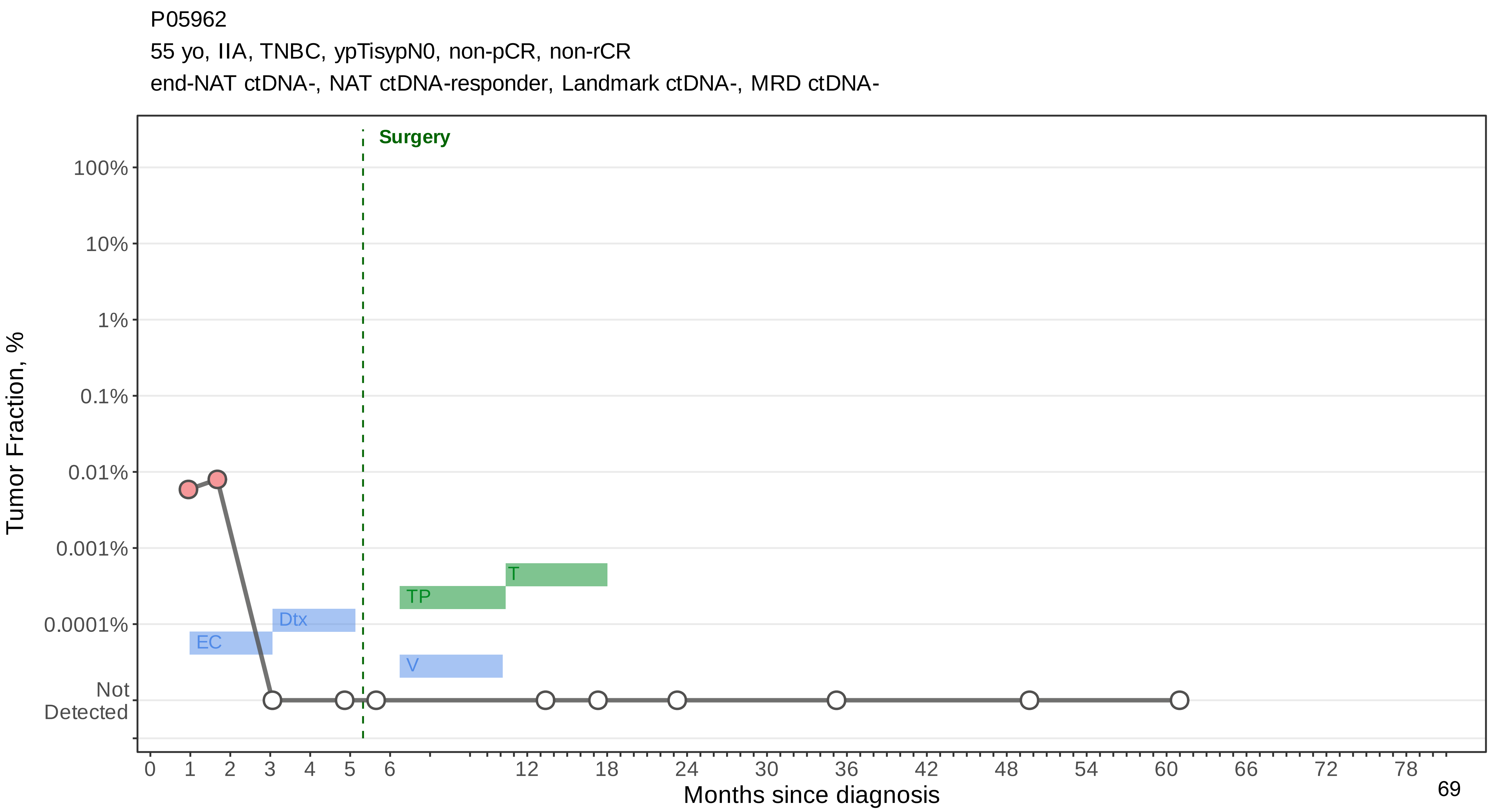

P05962
55 yo, IIA, TNBC, ypTisypN0, non-pCR, non-rCR
end-NAT ctDNA-, NAT ctDNA-responder, Landmark ctDNA-, MRD ctDNA-
Surgery
Tumor Fraction, %
100%
10%
1%
0.1%
0.01%
0.001%
0.0001%
Not Detected
EC
Dtx
TP
T
V
0
1
2
3
4
5
6
12
18
24
30
36
42
48
54
60
66
72
78
Months since diagnosis

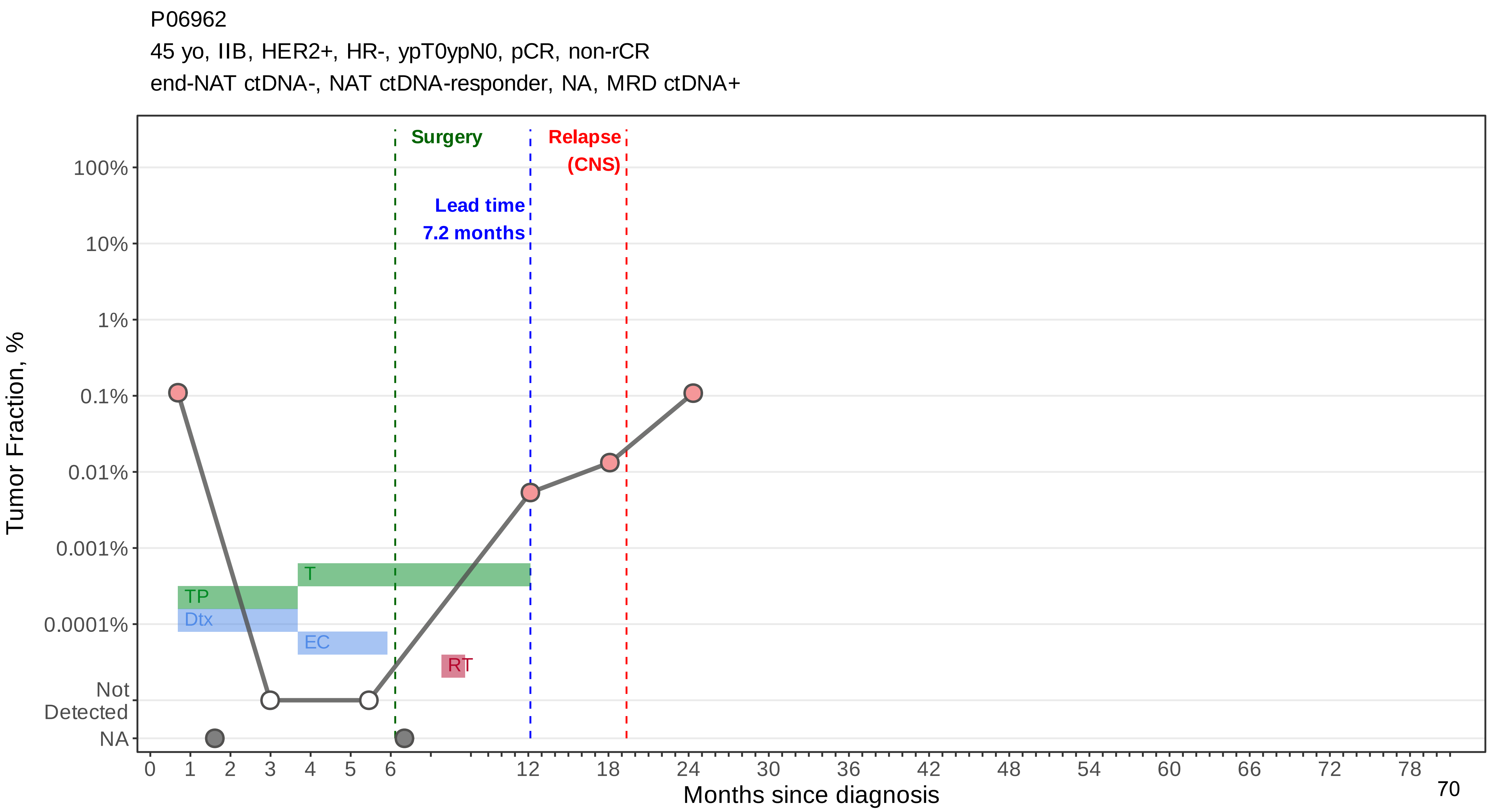

P06962
45 yo, IIB, HER2+, HR-, ypT0ypN0, pCR, non-rCR
end-NAT ctDNA-, NAT ctDNA-responder, NA, MRD ctDNA+
Surgery
Relapse
(CNS)
Lead time
7.2 months
100%
10%
1%
0.1%
0.01%
0.001%
0.0001%
Not Detected
NA
Tumor Fraction, %
T
TP
Dtx
EC
RT
0 1 2 3 4 5 6 12 18 24 30 36 42 48 54 60 66 72 78
Months since diagnosis

P07962
45 yo, IIA, TNBC, ypT1ypN0, non-pCR, non-rCR
end-NAT ctDNA-, NAT ctDNA-responder, Landmark ctDNA-, MRD ctDNA-

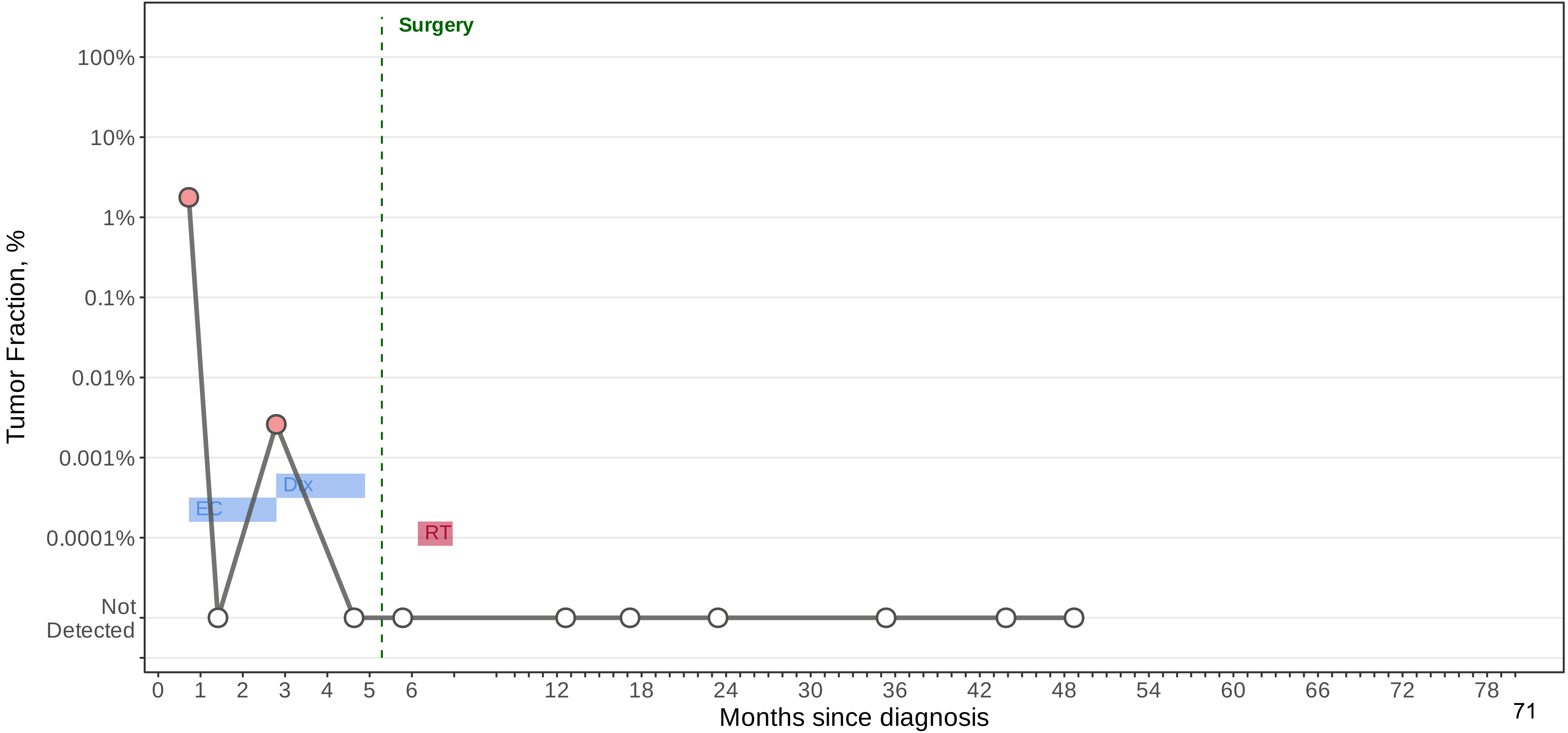

P08962
40 yo, IIB, TNBC, ypT1ypN0, non-pCR, non-rCR
end-NAT ctDNA-, NAT ctDNA-responder, Landmark ctDNA-, MRD ctDNA-

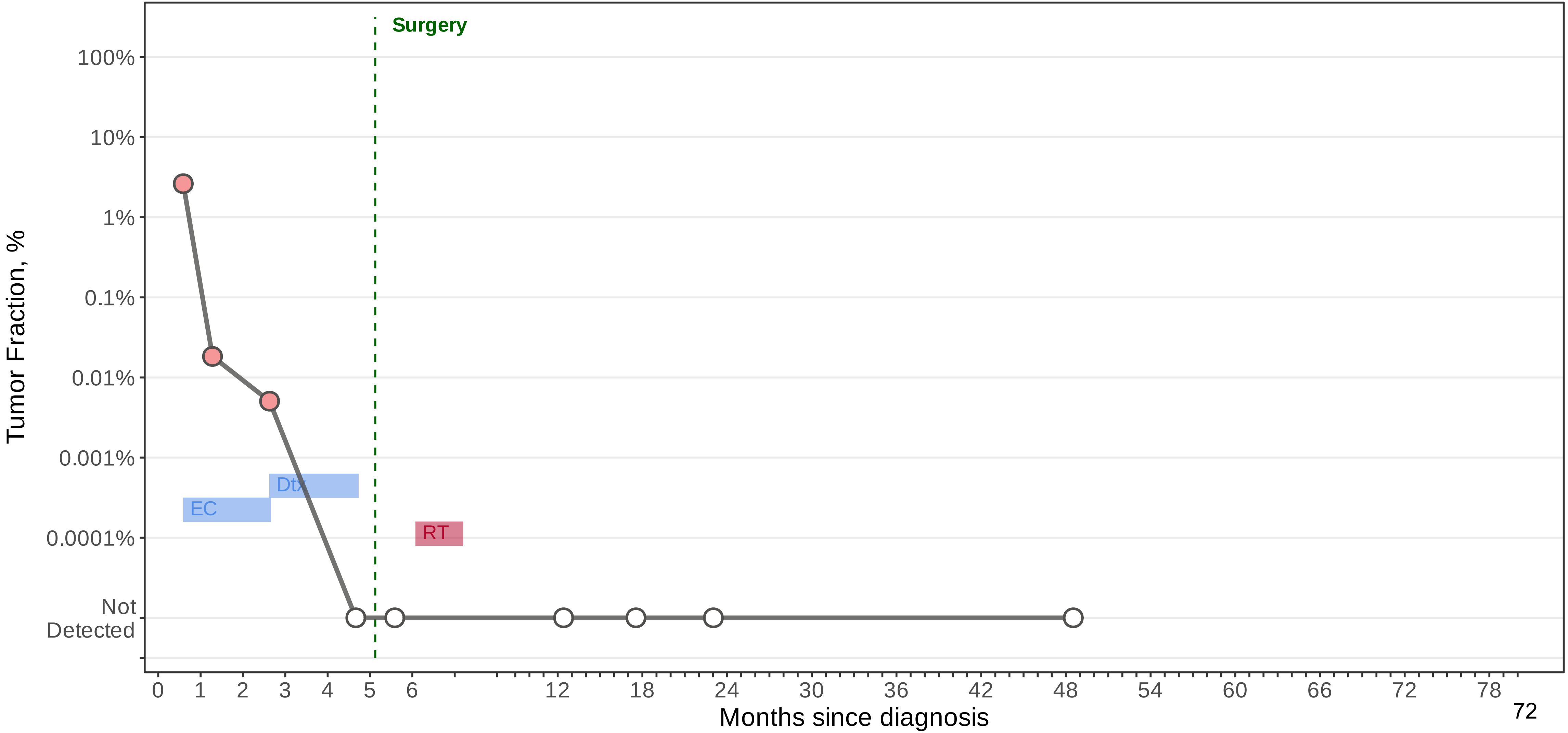

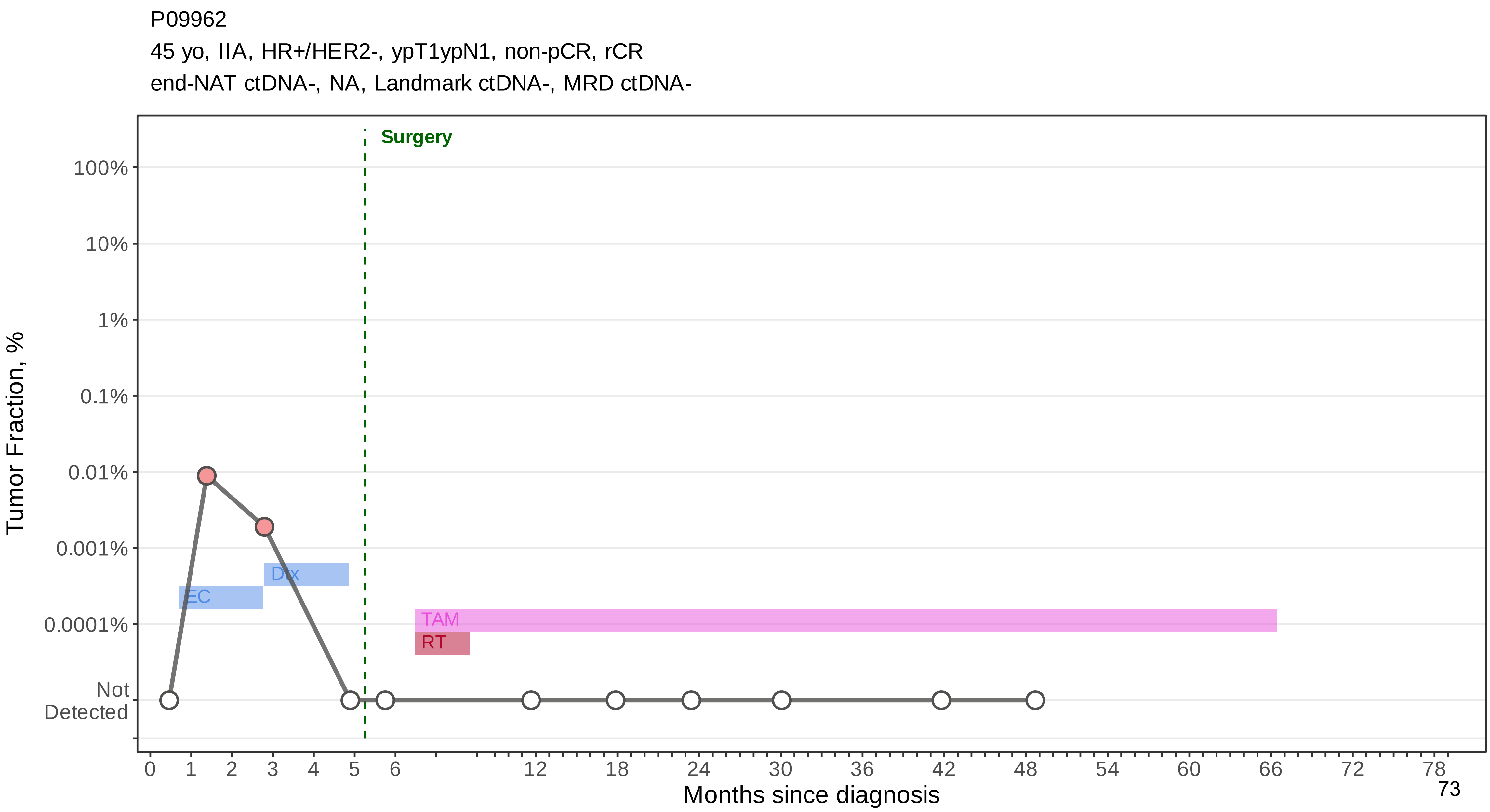

P09962
45 yo, IIA, HR+/HER2-, ypT1ypN1, non-pCR, rCR
end-NAT ctDNA-, NA, Landmark ctDNA-, MRD ctDNA-
Surgery
100%
10%
1%
0.1%
0.01%
0.001%
0.0001%
Not Detected
Tumor Fraction, %
EC
Dtx
TAM
RT
0 1 2 3 4 5 6 12 18 24 30 36 42 48 54 60 66 72 78
Months since diagnosis

P00072

55 yo, IIA, HR+/HER2-, ypT1ypN0, non-pCR, non-rCR

NA, NAT ctDNA-responder, Landmark ctDNA-, MRD ctDNA-

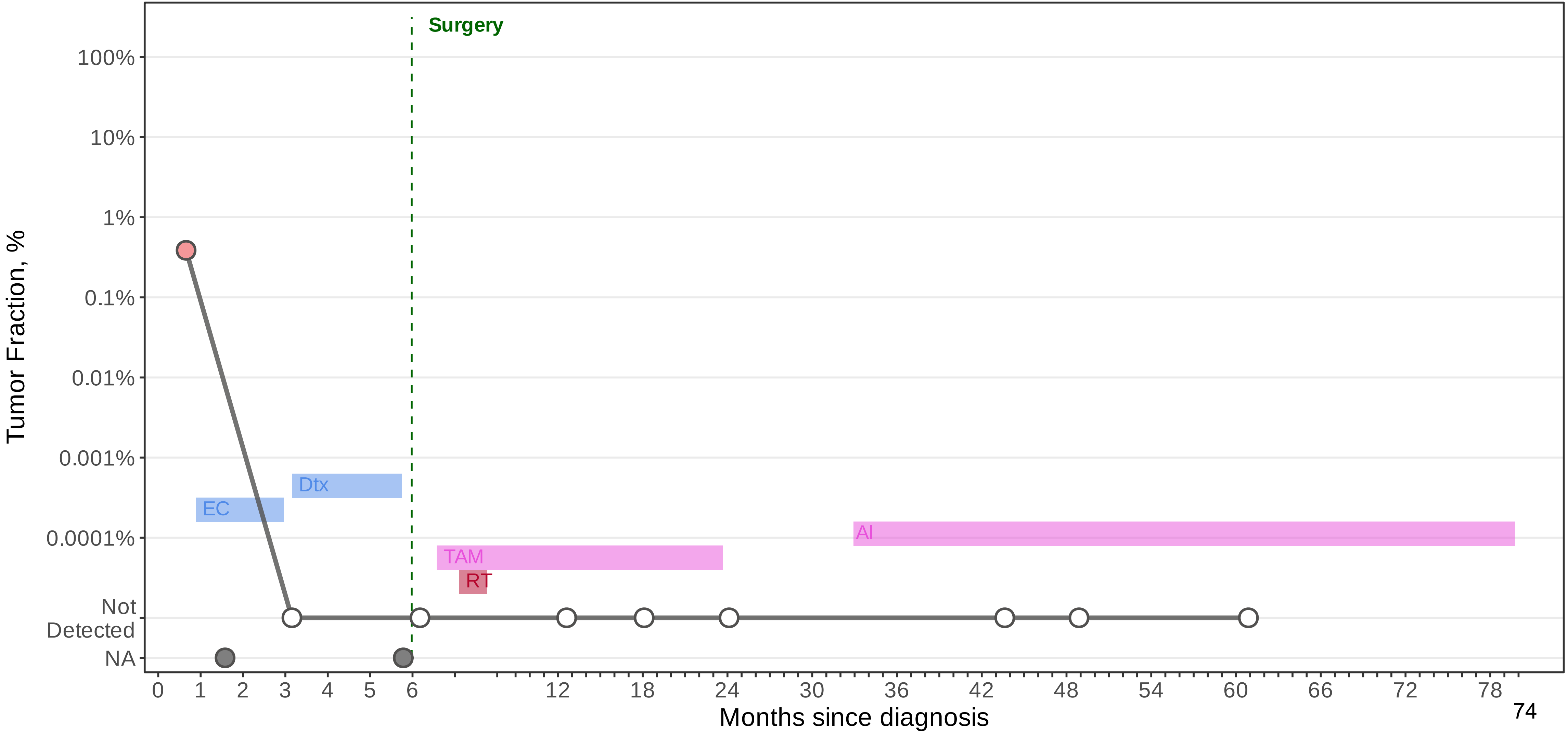

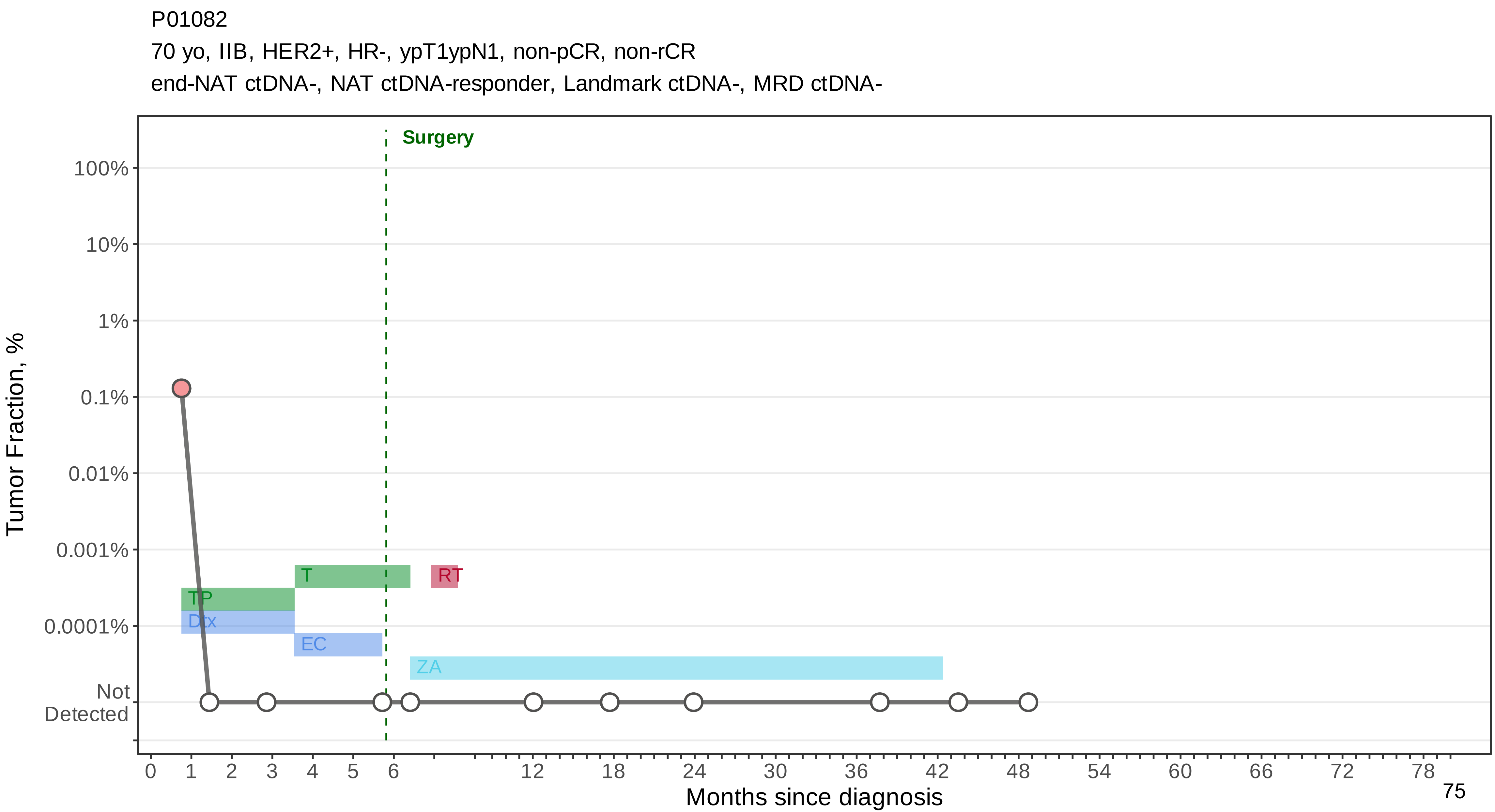
P01082
70 yo, IIB, HER2+, HR-, ypT1ypN1, non-pCR, non-rCR
end-NAT ctDNA-, NAT ctDNA-responder, Landmark ctDNA-, MRD ctDNA-
Surgery
Tumor Fraction, %
100%
10%
1%
0.1%
0.01%
0.001%
0.0001%
Not Detected
T
RT
TP
Dtx
EC
ZA
0 1 2 3 4 5 6 12 18 24 30 36 42 48 54 60 66 72 78
Months since diagnosis

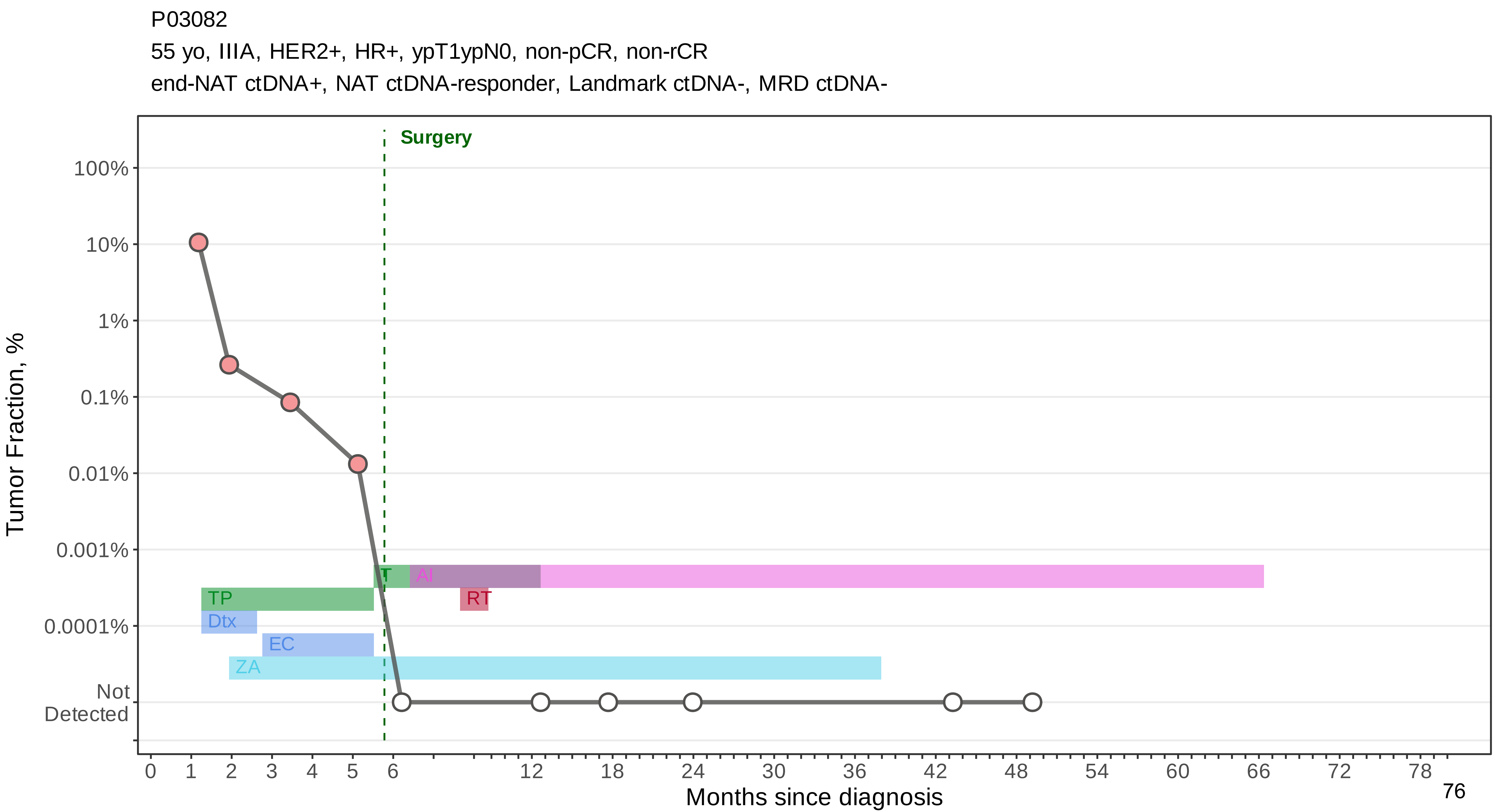
P03082
55 yo, IIIA, HER2+, HR+, ypT1ypN0, non-pCR, non-rCR
end-NAT ctDNA+, NAT ctDNA-responder, Landmark ctDNA-, MRD ctDNA-
Surgery
Tumor Fraction, %
100%
10%
1%
0.1%
0.01%
0.001%
0.0001%
Not Detected
T
AI
RT
TP
Dtx
EC
ZA
0 1 2 3 4 5 6 12 18 24 30 36 42 48 54 60 66 72 78
Months since diagnosis

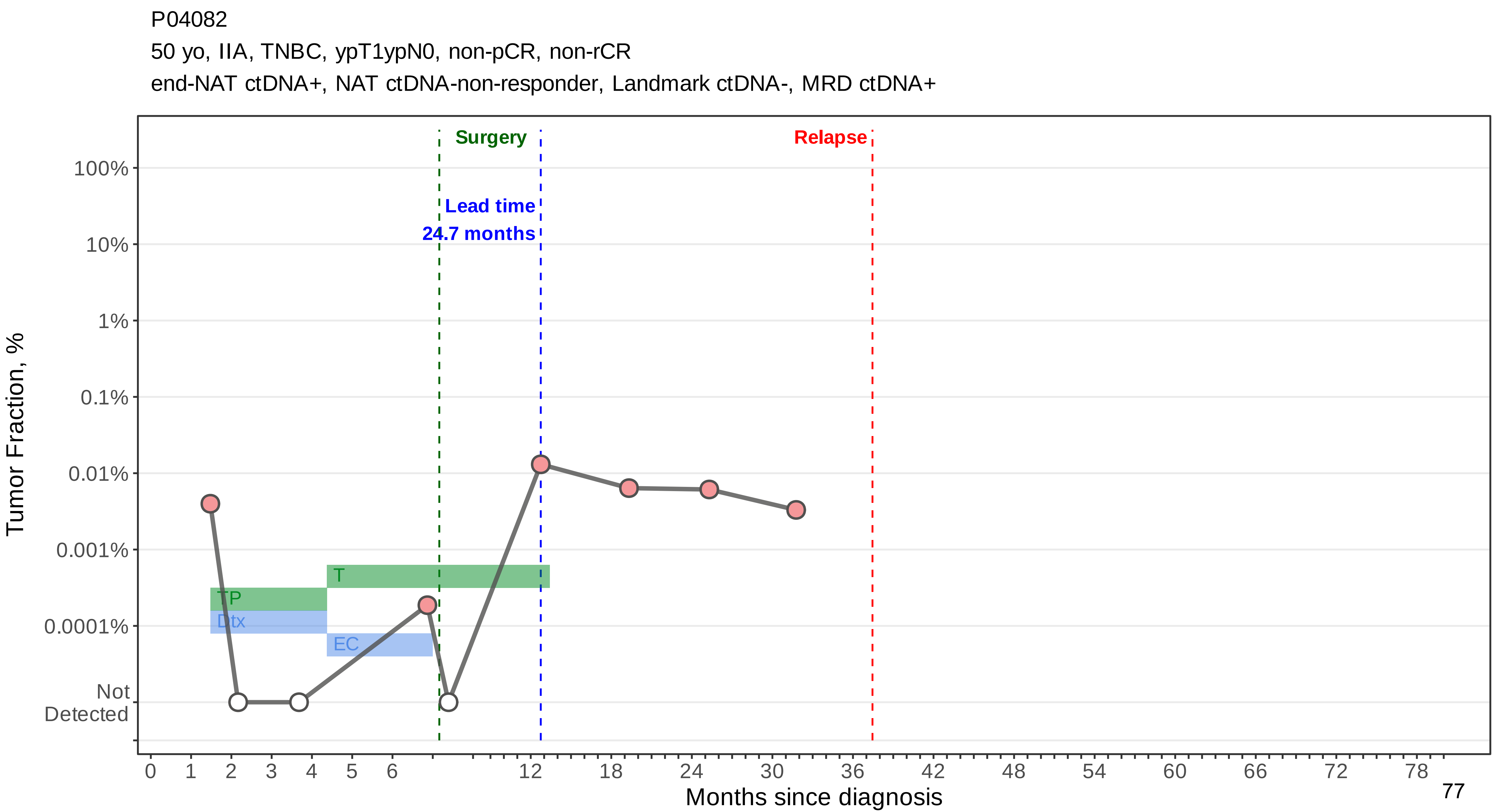

P04082
50 yo, IIA, TNBC, ypT1ypN0, non-pCR, non-rCR
end-NAT ctDNA+, NAT ctDNA-non-responder, Landmark ctDNA-, MRD ctDNA+
Surgery
Relapse
Lead time
24.7 months
Tumor Fraction, %
100%
10%
1%
0.1%
0.01%
0.001%
0.0001%
Not Detected
T
TP
Dtx
EC
0
1
2
3
4
5
6
12
18
24
30
36
42
48
54
60
66
72
78
Months since diagnosis

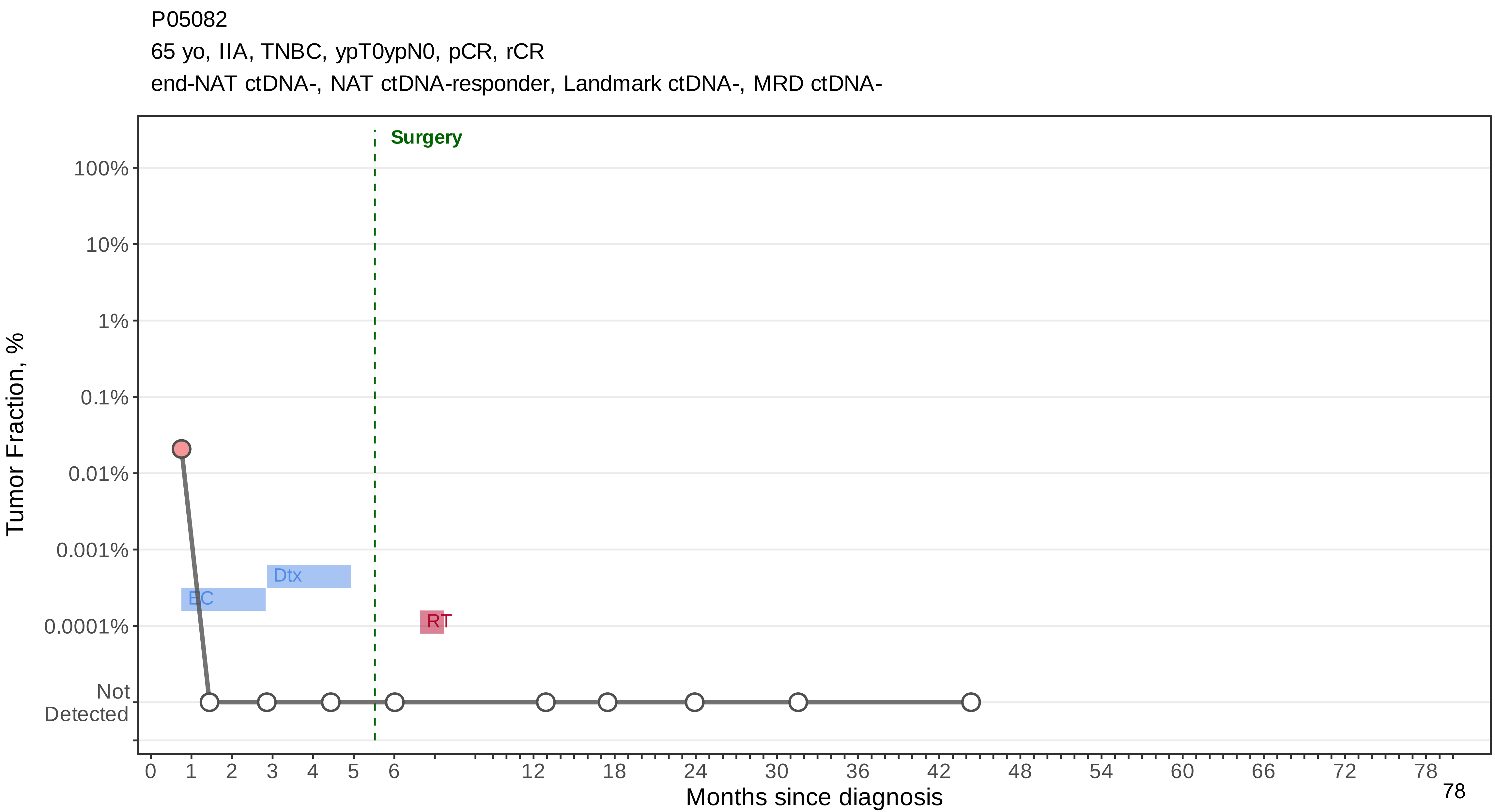
P05082
65 yo, IIA, TNBC, ypT0ypN0, pCR, rCR
end-NAT ctDNA-, NAT ctDNA-responder, Landmark ctDNA-, MRD ctDNA-
Surgery
100%
10%
1%
0.1%
0.01%
0.001%
0.0001%
Not Detected
Tumor Fraction, %
Dtx
EC
RT
0 1 2 3 4 5 6 12 18 24 30 36 42 48 54 60 66 72 78
Months since diagnosis

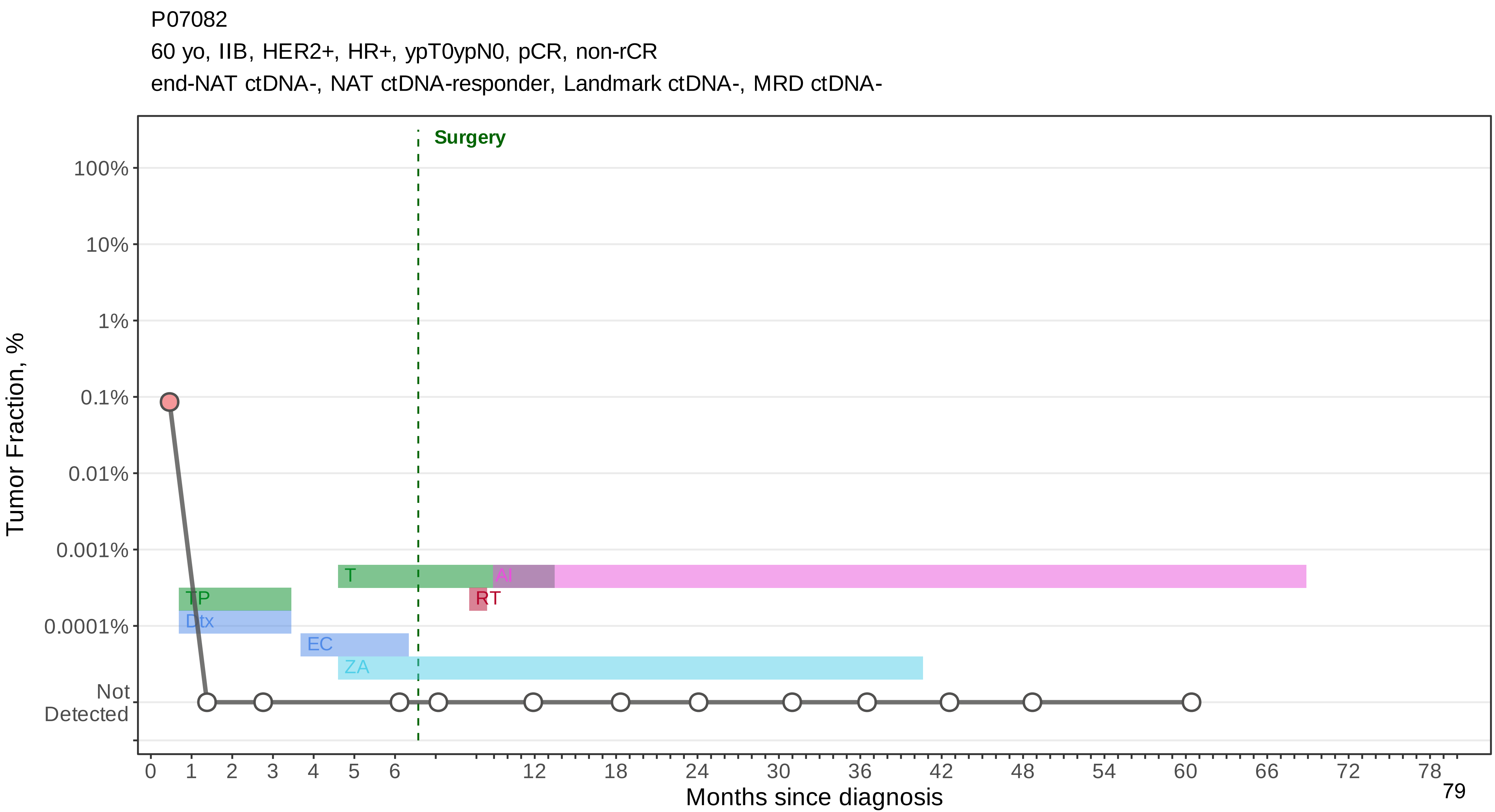
P07082
60 yo, IIB, HER2+, HR+, ypT0ypN0, pCR, non-rCR
end-NAT ctDNA-, NAT ctDNA-responder, Landmark ctDNA-, MRD ctDNA-
Surgery
100%
10%
1%
0.1%
0.01%
0.001%
0.0001%
Not Detected
Tumor Fraction, %
T
AI
TP
RT
Dtx
EC
ZA
0 1 2 3 4 5 6 12 18 24 30 36 42 48 54 60 66 72 78
Months since diagnosis

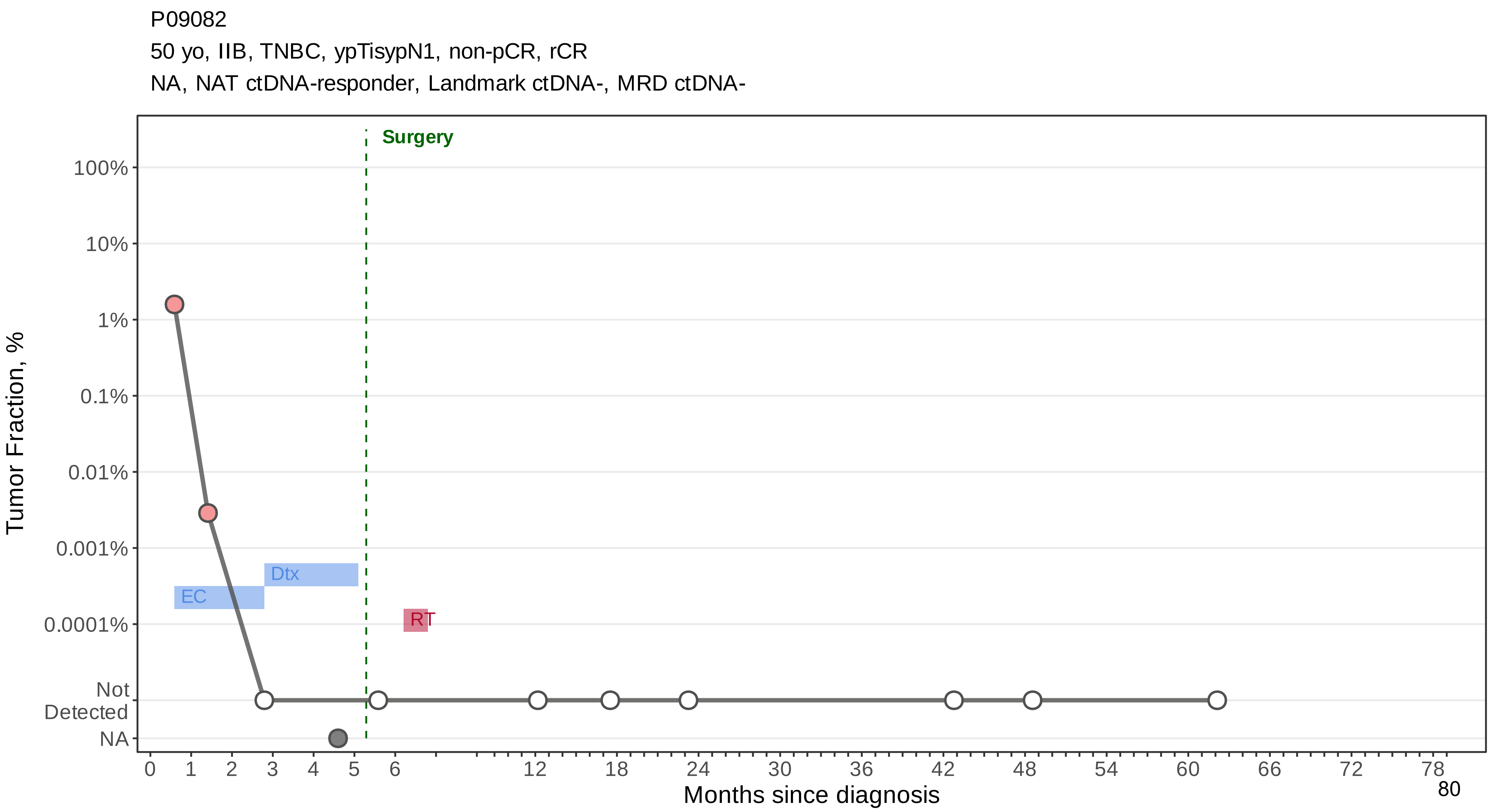
P09082
50 yo, IIB, TNBC, ypTisypN1, non-pCR, rCR
NA, NAT ctDNA-responder, Landmark ctDNA-, MRD ctDNA-
Surgery
100%
10%
1%
0.1%
0.01%
0.001%
0.0001%
Not Detected
NA
Dtx
EC
RT
Tumor Fraction, %
0 1 2 3 4 5 6 12 18 24 30 36 42 48 54 60 66 72 78
Months since diagnosis

P00182
50 yo, IIB, HER2+, HR-, ypT0ypN0, pCR, non-rCR
end-NAT ctDNA-, NAT ctDNA-responder, Landmark ctDNA-, MRD ctDNA-

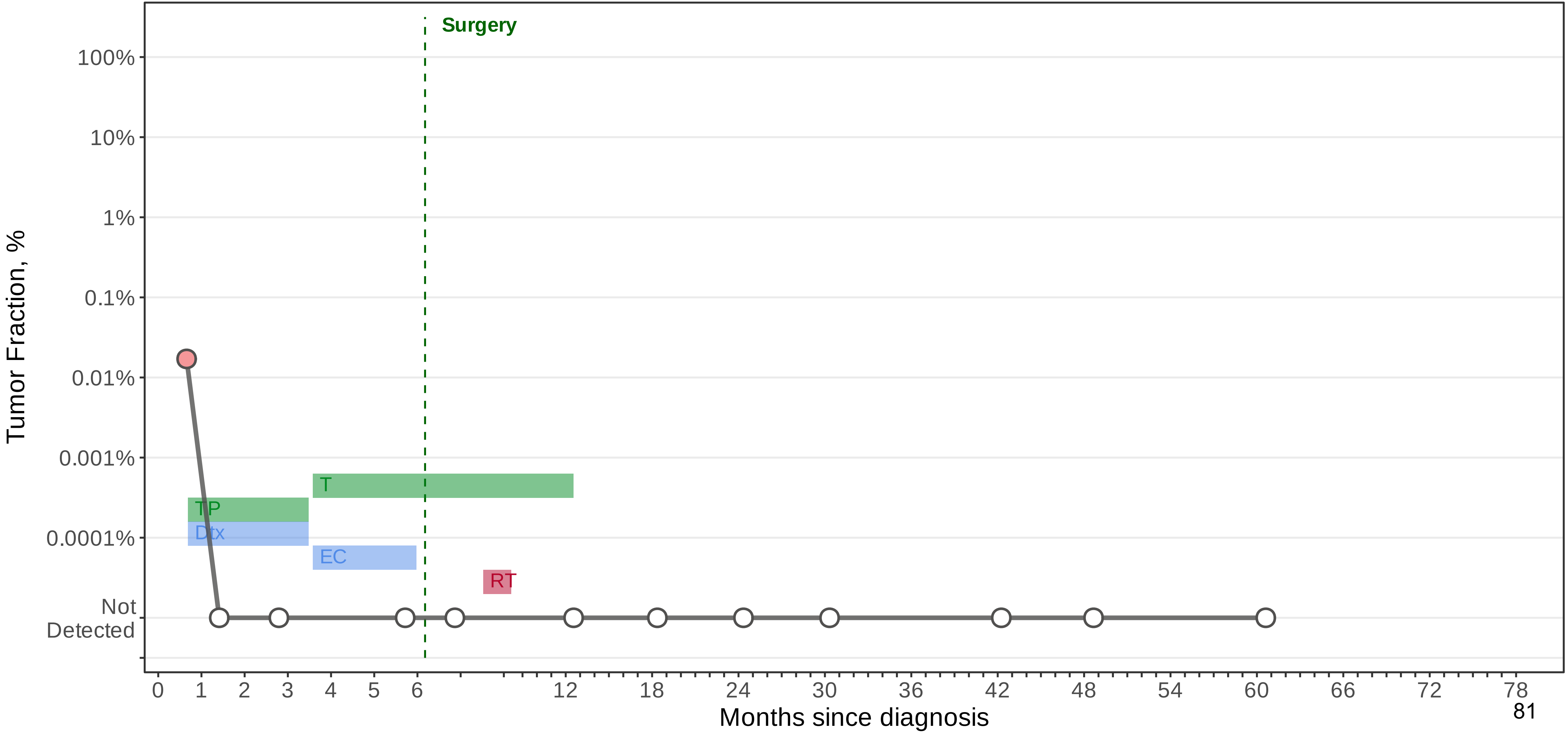

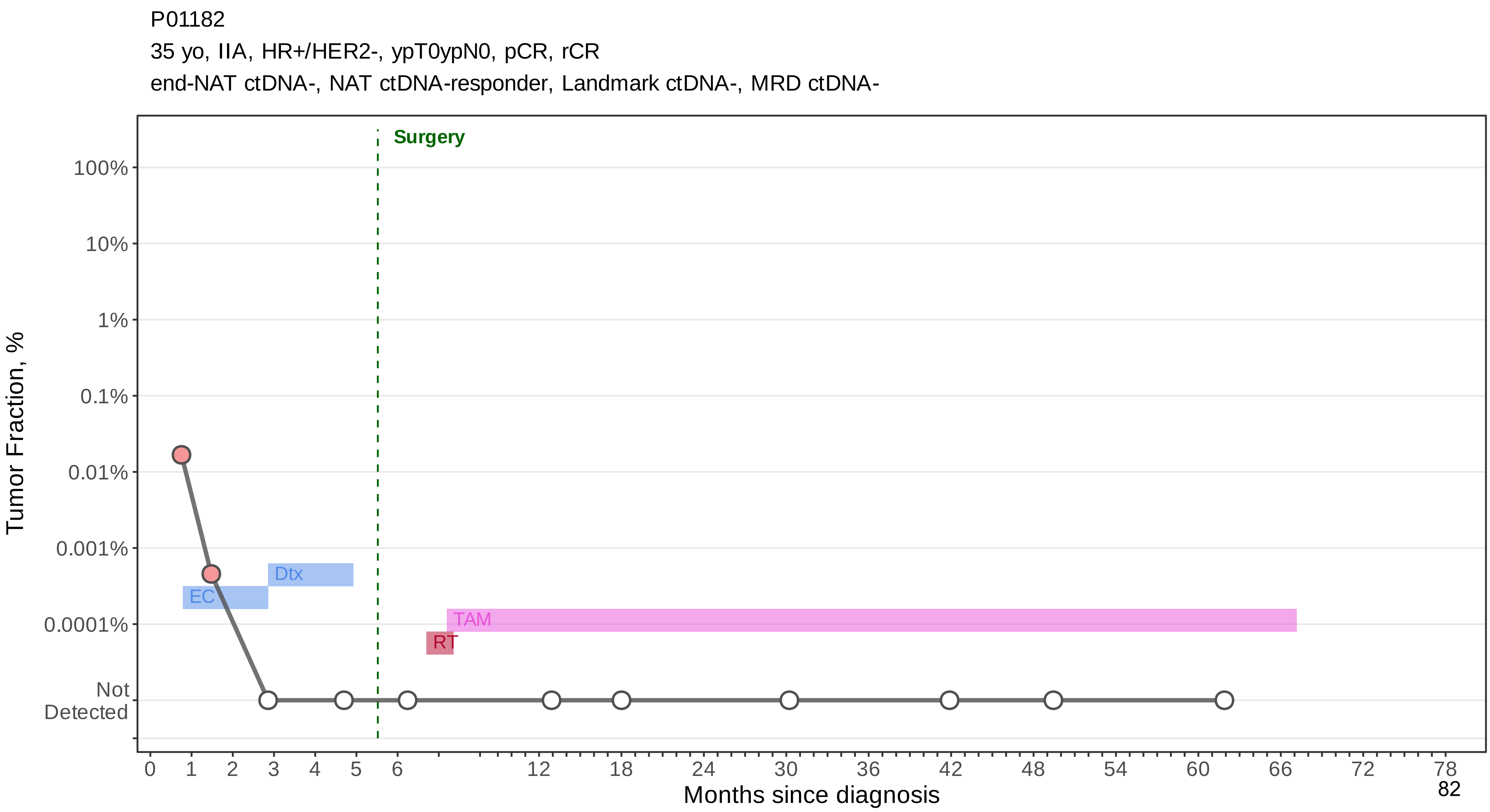
P01182
35 yo, IIA, HR+/HER2-, ypT0ypN0, pCR, rCR
end-NAT ctDNA-, NAT ctDNA-responder, Landmark ctDNA-, MRD ctDNA-
Surgery
100%
10%
1%
0.1%
0.01%
0.001%
0.0001%
Not Detected
Tumor Fraction, %
Dtx
EC
TAM
RT
0 1 2 3 4 5 6 12 18 24 30 36 42 48 54 60 66 72 78
Months since diagnosis

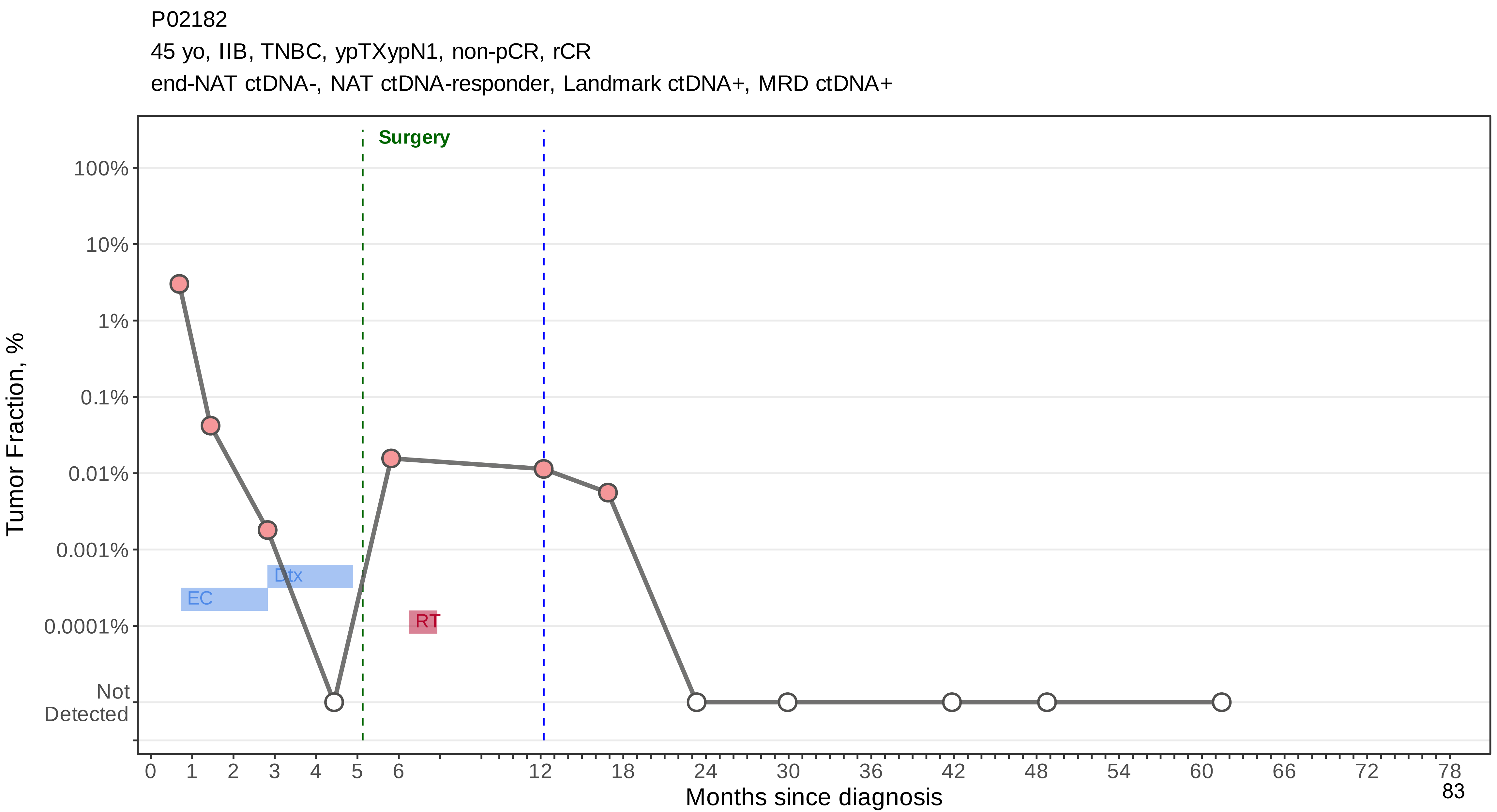
P02182
45 yo, IIB, TNBC, ypTXypN1, non-pCR, rCR
end-NAT ctDNA-, NAT ctDNA-responder, Landmark ctDNA+, MRD ctDNA+
Surgery
100%
10%
1%
0.1%
0.01%
0.001%
0.0001%
Not Detected
Tumor Fraction, %
EC
Dtx
RT
0
1
2
3
4
5
6
12
18
24
30
36
42
48
54
60
66
72
78
Months since diagnosis

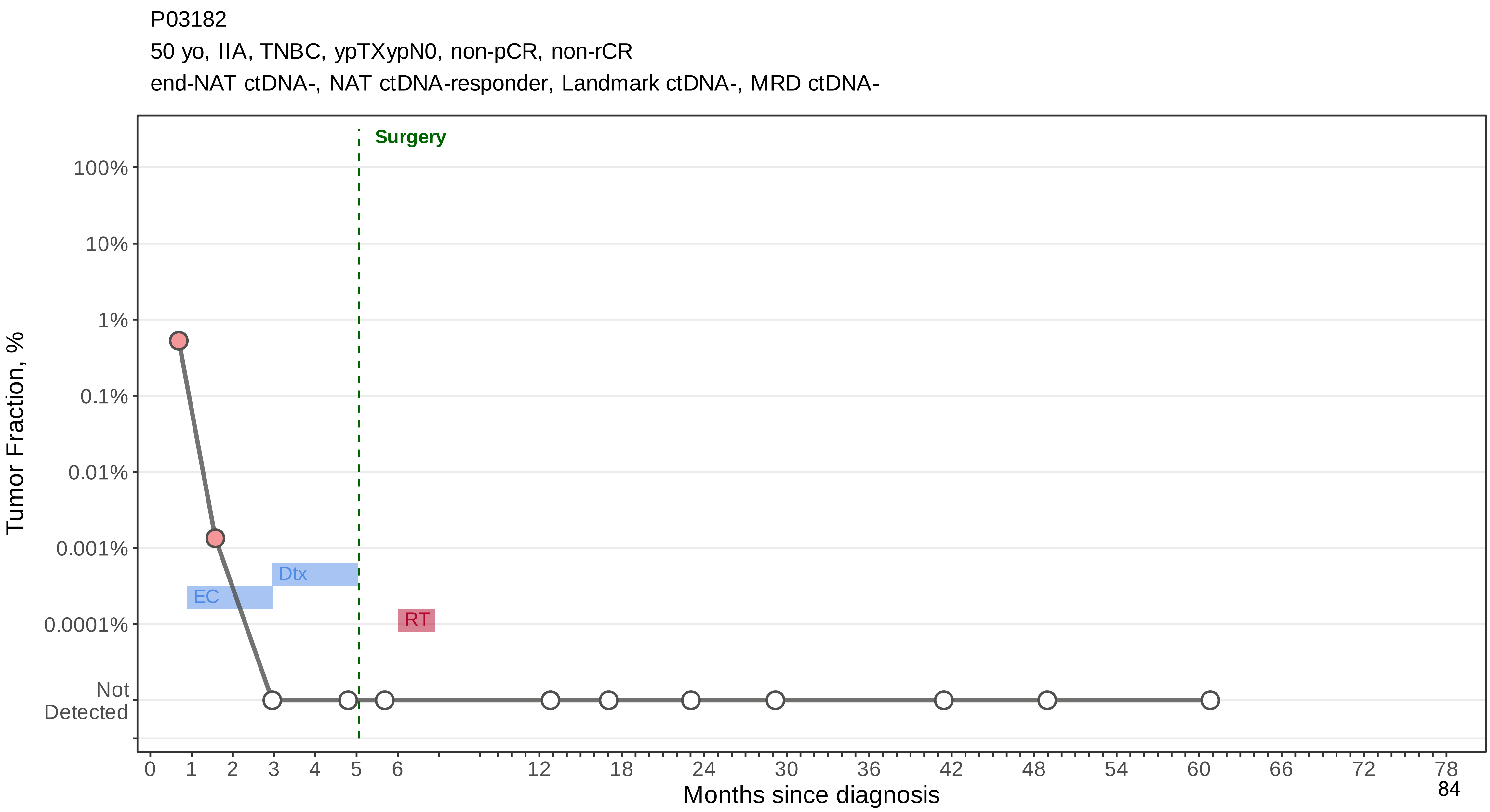
P03182
50 yo, IIA, TNBC, ypTXypN0, non-pCR, non-rCR
end-NAT ctDNA-, NAT ctDNA-responder, Landmark ctDNA-, MRD ctDNA-
Surgery
100%
10%
1%
0.1%
0.01%
0.001%
0.0001%
Not Detected
Tumor Fraction, %
Dtx
EC
RT
0 1 2 3 4 5 6 12 18 24 30 36 42 48 54 60 66 72 78
Months since diagnosis

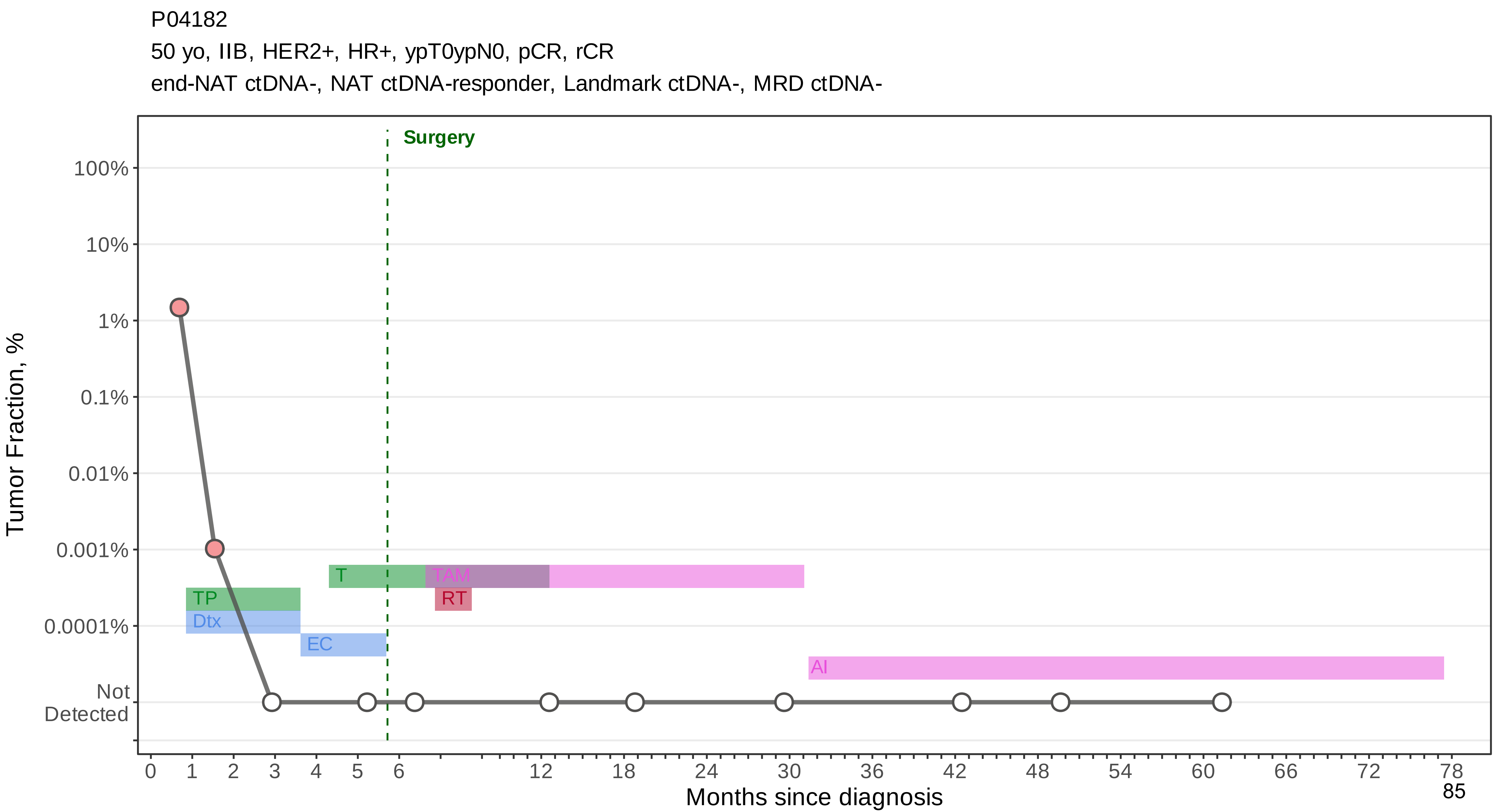
P04182
50 yo, IIB, HER2+, HR+, ypT0ypN0, pCR, rCR
end-NAT ctDNA-, NAT ctDNA-responder, Landmark ctDNA-, MRD ctDNA-
Surgery
100%
10%
1%
0.1%
0.01%
0.001%
0.0001%
Not Detected
Tumor Fraction, %
TP
Dtx
EC
T
TAM
RT
AI
0 1 2 3 4 5 6 12 18 24 30 36 42 48 54 60 66 72 78
Months since diagnosis

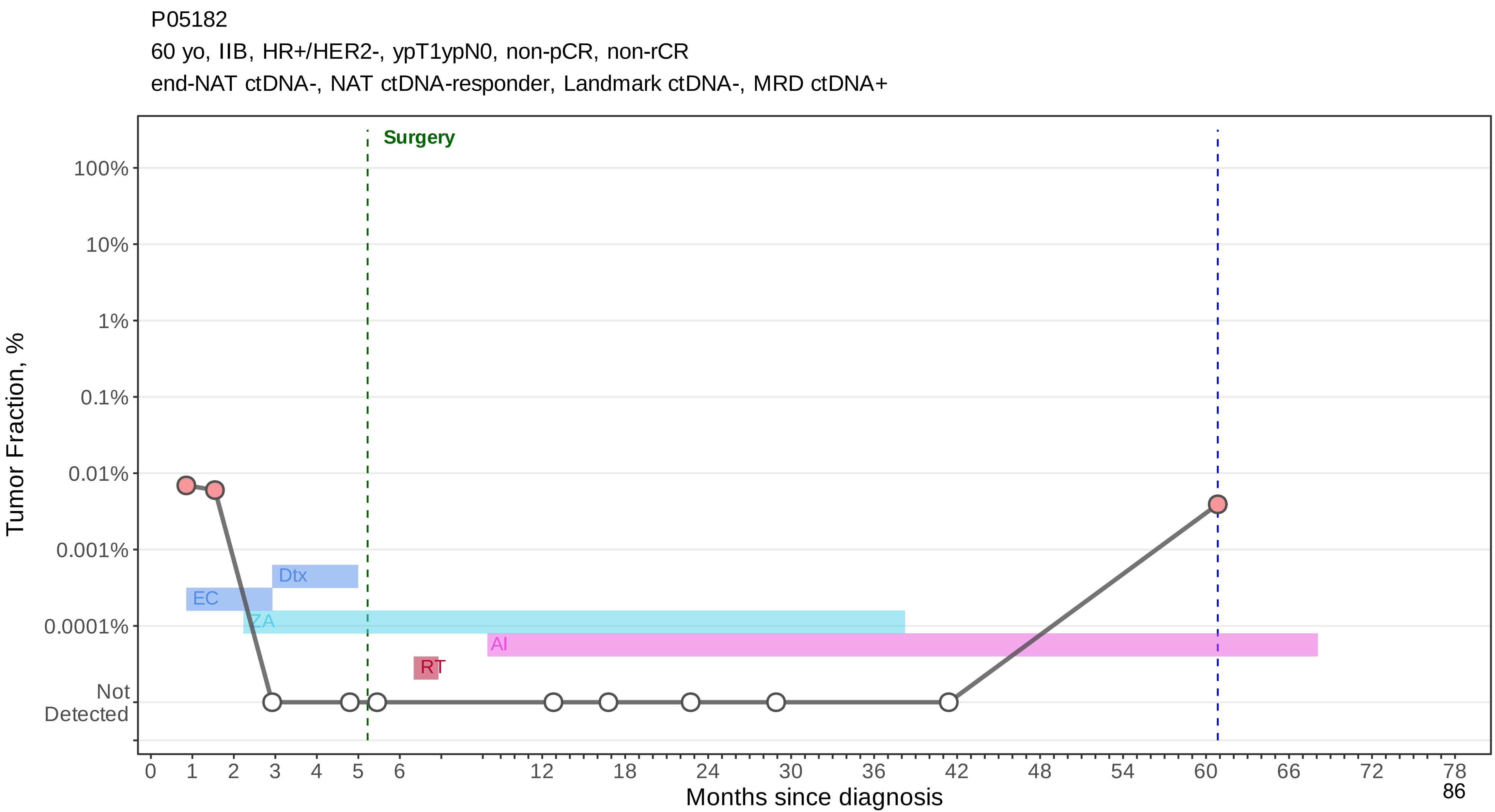
P05182
60 yo, IIB, HR+/HER2-, ypT1ypN0, non-pCR, non-rCR
end-NAT ctDNA-, NAT ctDNA-responder, Landmark ctDNA-, MRD ctDNA+
Surgery
100%
10%
1%
0.1%
0.01%
0.001%
0.0001%
Not Detected
Tumor Fraction, %
Dtx
EC
ZA
AI
RT
0 1 2 3 4 5 6 12 18 24 30 36 42 48 54 60 66 72 78
Months since diagnosis

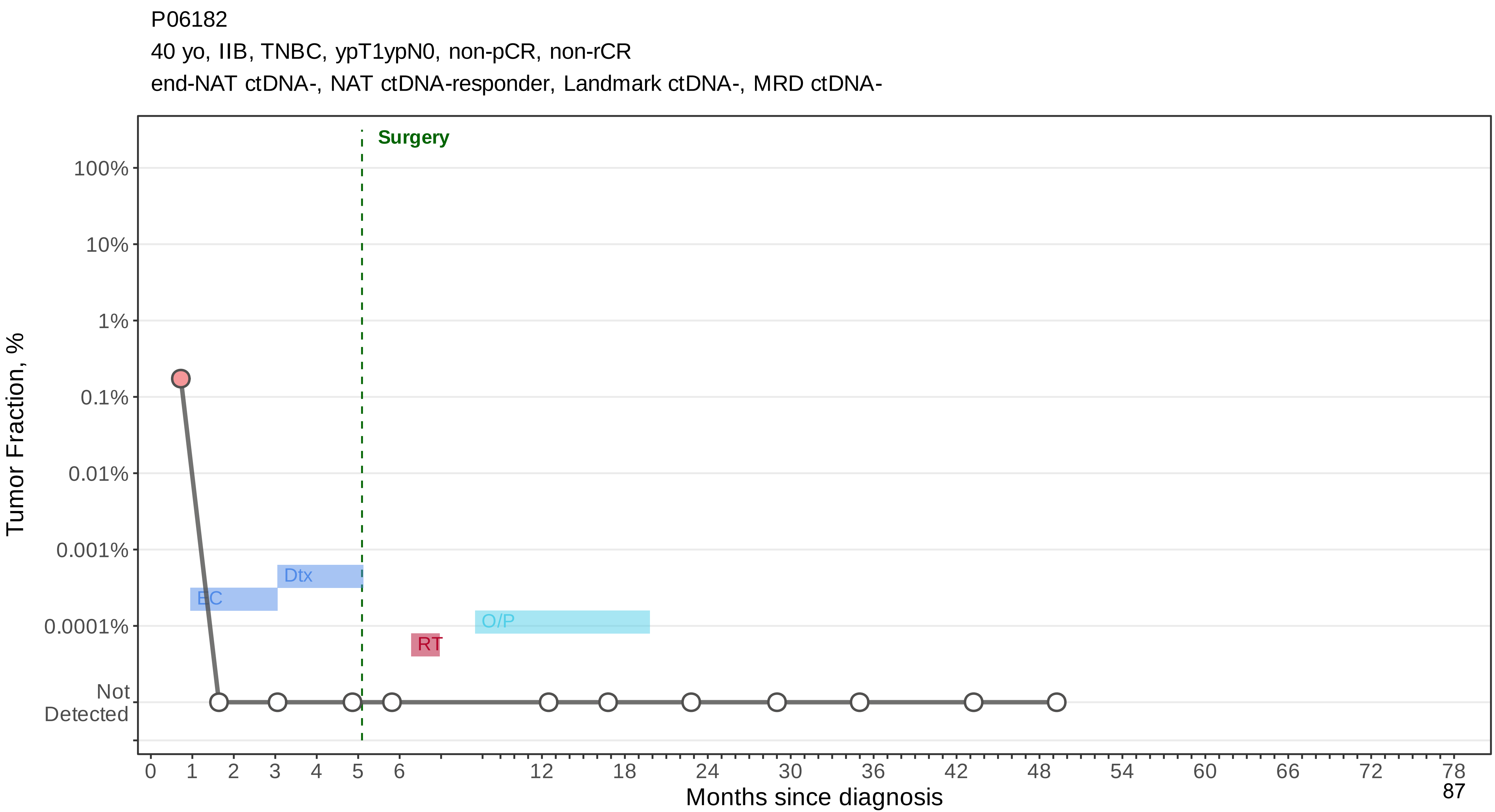
P06182
40 yo, IIB, TNBC, ypT1ypN0, non-pCR, non-rCR
end-NAT ctDNA-, NAT ctDNA-responder, Landmark ctDNA-, MRD ctDNA-
Surgery
Tumor Fraction, %
100%
10%
1%
0.1%
0.01%
0.001%
0.0001%
Not Detected
EC
Dtx
RT
O/P
0 1 2 3 4 5 6 12 18 24 30 36 42 48 54 60 66 72 78
Months since diagnosis

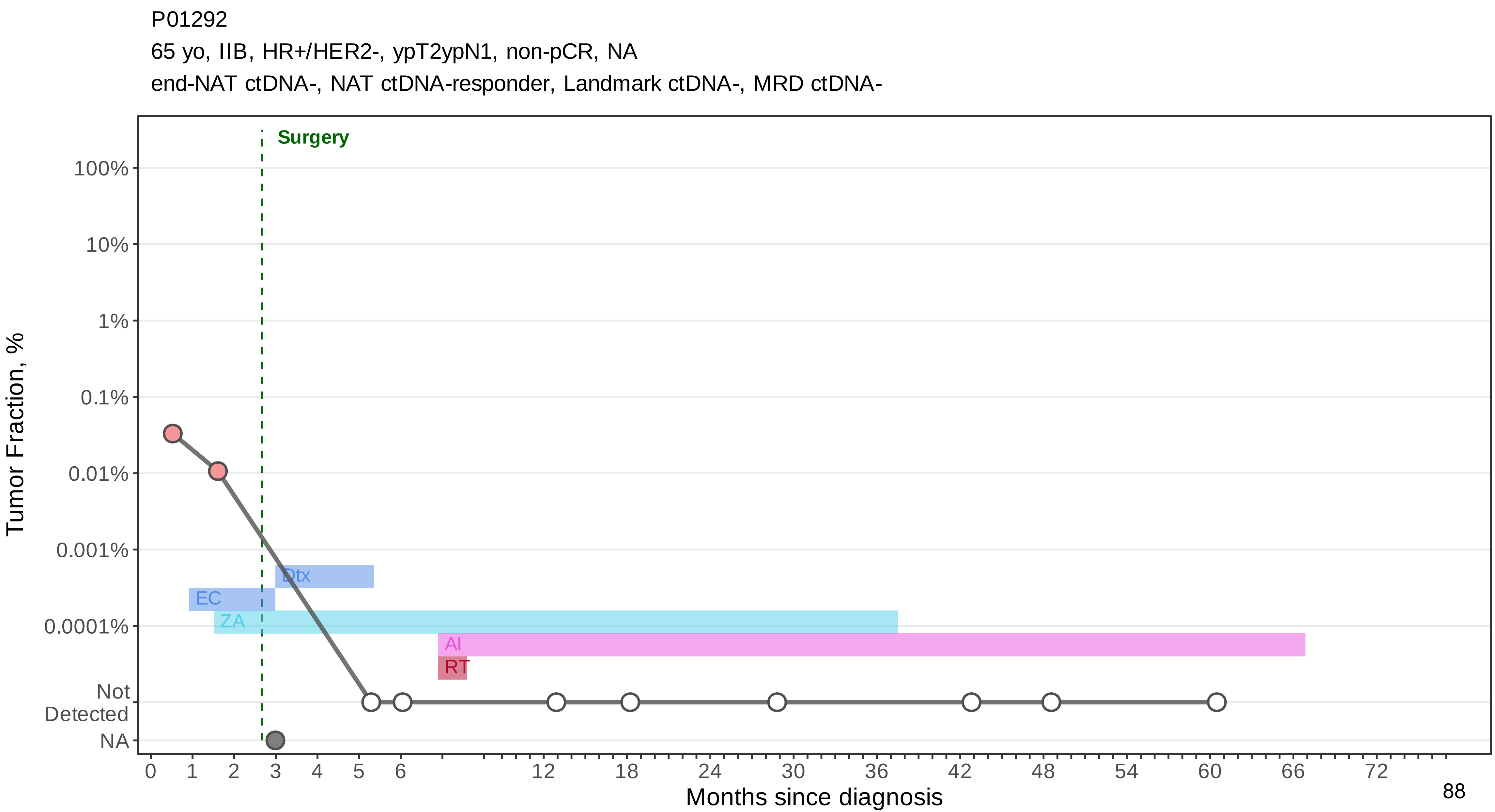
P01292
65 yo, IIB, HR+/HER2-, ypT2ypN1, non-pCR, NA
end-NAT ctDNA-, NAT ctDNA-responder, Landmark ctDNA-, MRD ctDNA-
Surgery
Tumor Fraction, %
100%
10%
1%
0.1%
0.01%
0.001%
0.0001%
Not Detected
NA
Dtx
EC
ZA
AI
RT
0 1 2 3 4 5 6 12 18 24 30 36 42 48 54 60 66 72
Months since diagnosis

P02292

35 yo, IIB, TNBC, ypT2ypN3, non-pCR, NA

end-NAT ctDNA+, NAT ctDNA-non-responder, Landmark ctDNA+, MRD ctDNA+

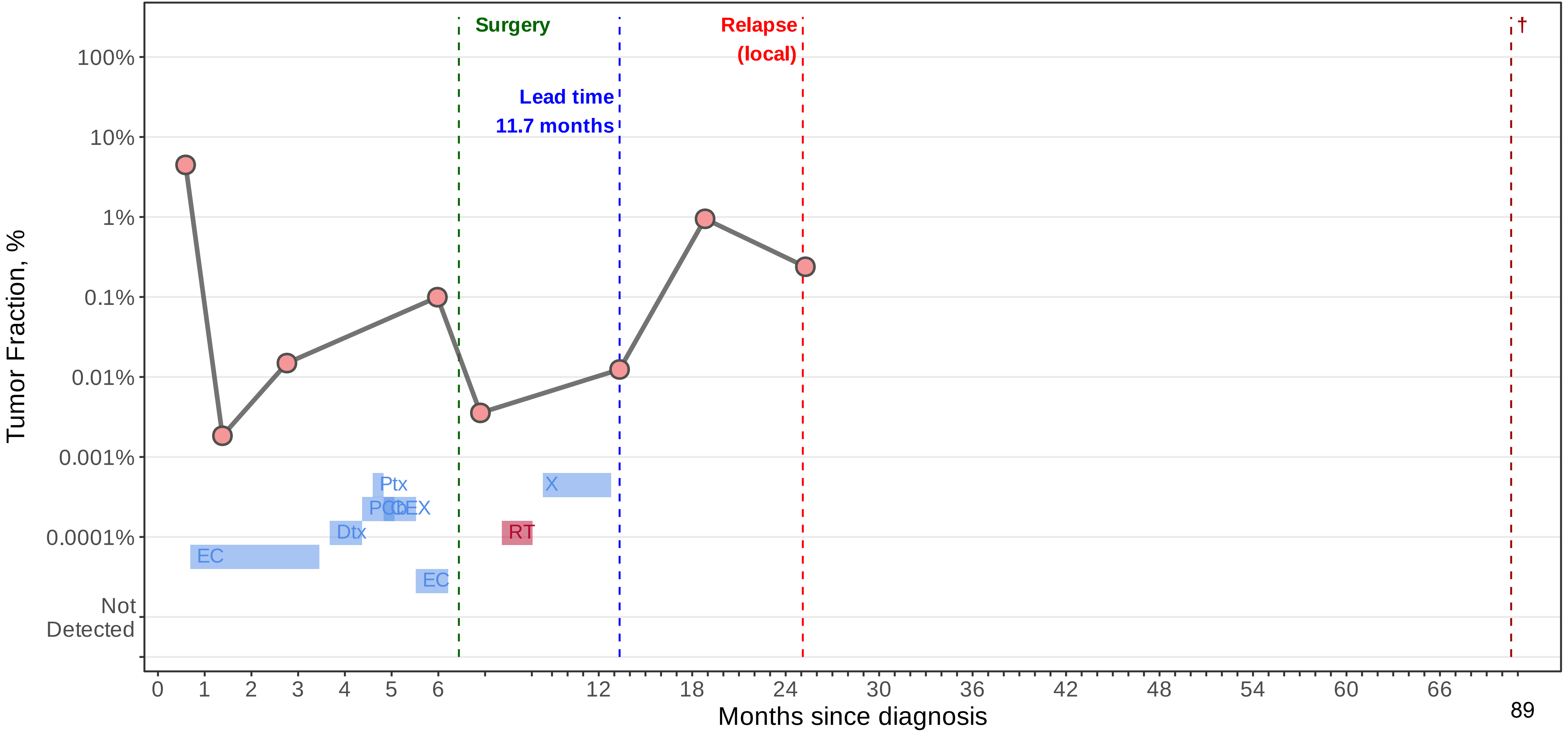

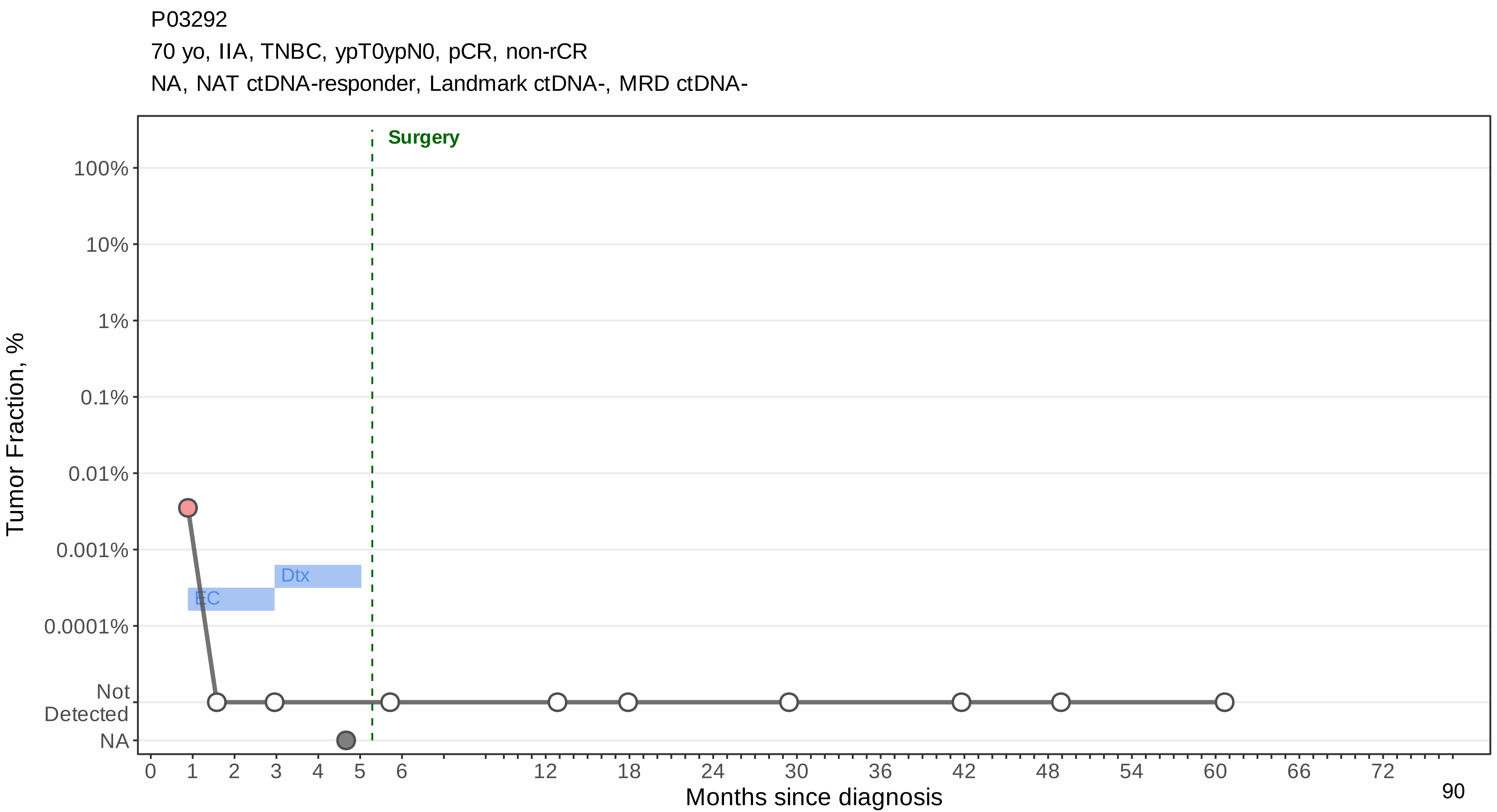

P03292
70 yo, IIA, TNBC, ypT0ypN0, pCR, non-rCR
NA, NAT ctDNA-responder, Landmark ctDNA-, MRD ctDNA-
Surgery
Tumor Fraction, %
100%
10%
1%
0.1%
0.01%
0.001%
0.0001%
Not Detected
NA
EC
Dtx
0
1
2
3
4
5
6
12
18
24
30
36
42
48
54
60
66
72
Months since diagnosis

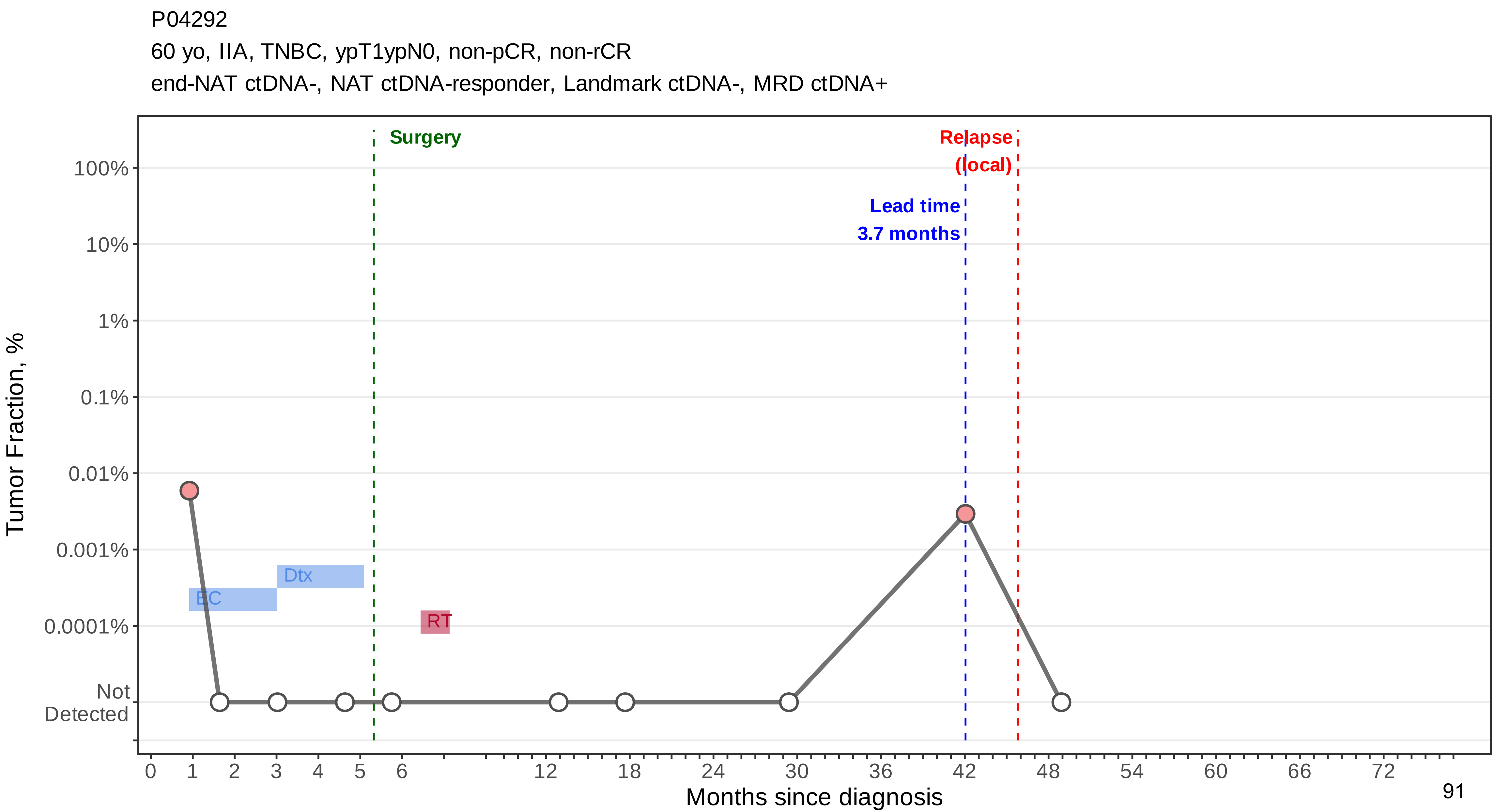
P04292
60 yo, IIA, TNBC, ypT1ypN0, non-pCR, non-rCR
end-NAT ctDNA-, NAT ctDNA-responder, Landmark ctDNA-, MRD ctDNA+
Surgery
Relapse
(local)
Lead time
3.7 months
Tumor Fraction, %
100%
10%
1%
0.1%
0.01%
0.001%
0.0001%
Not Detected
EC
Dtx
RT
0
1
2
3
4
5
6
12
18
24
30
36
42
48
54
60
66
72
Months since diagnosis

P05292

55 yo, NA, TNBC, ypT1ypN0, non-pCR, non-rCR

end-NAT ctDNA-, NAT ctDNA-responder, Landmark ctDNA-, MRD ctDNA-

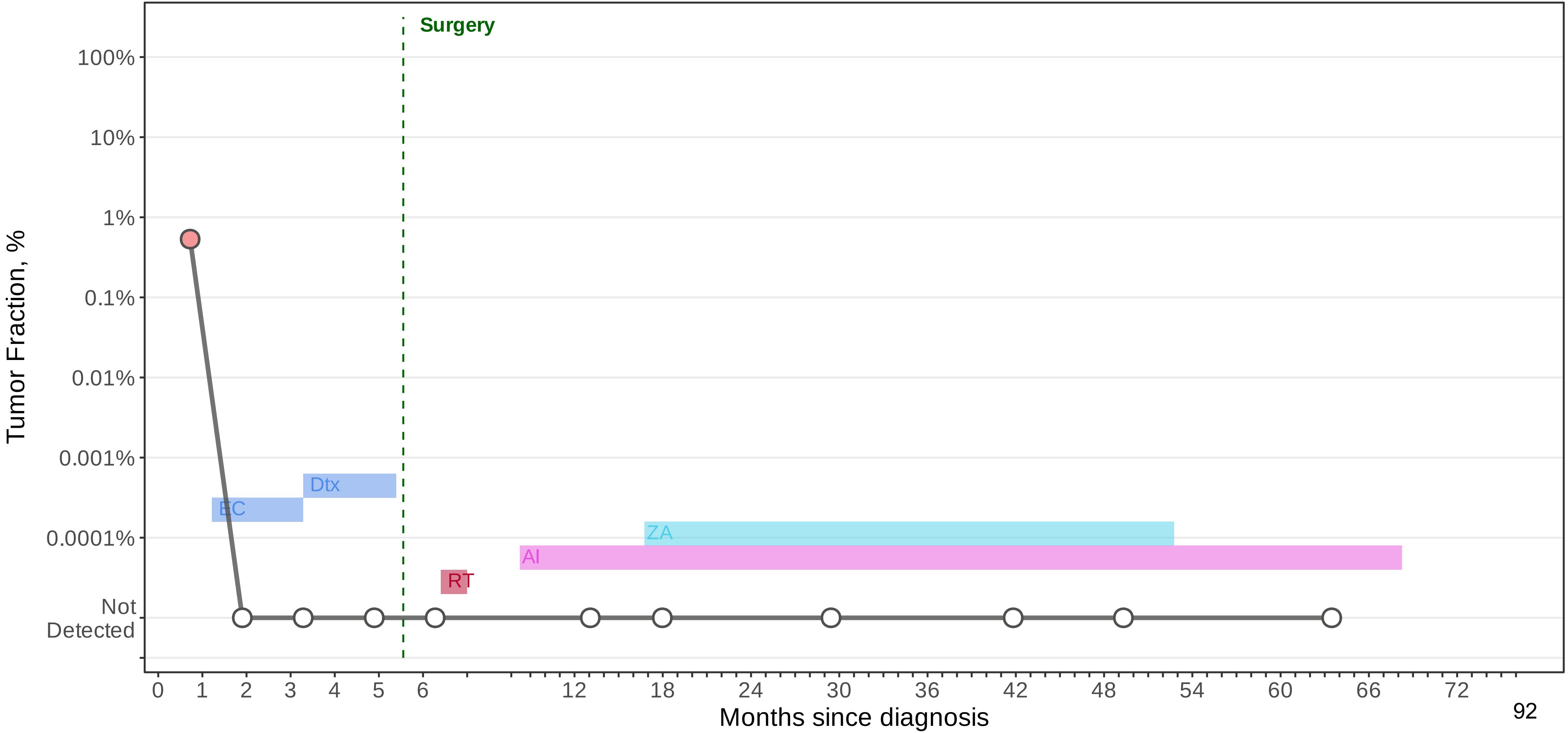

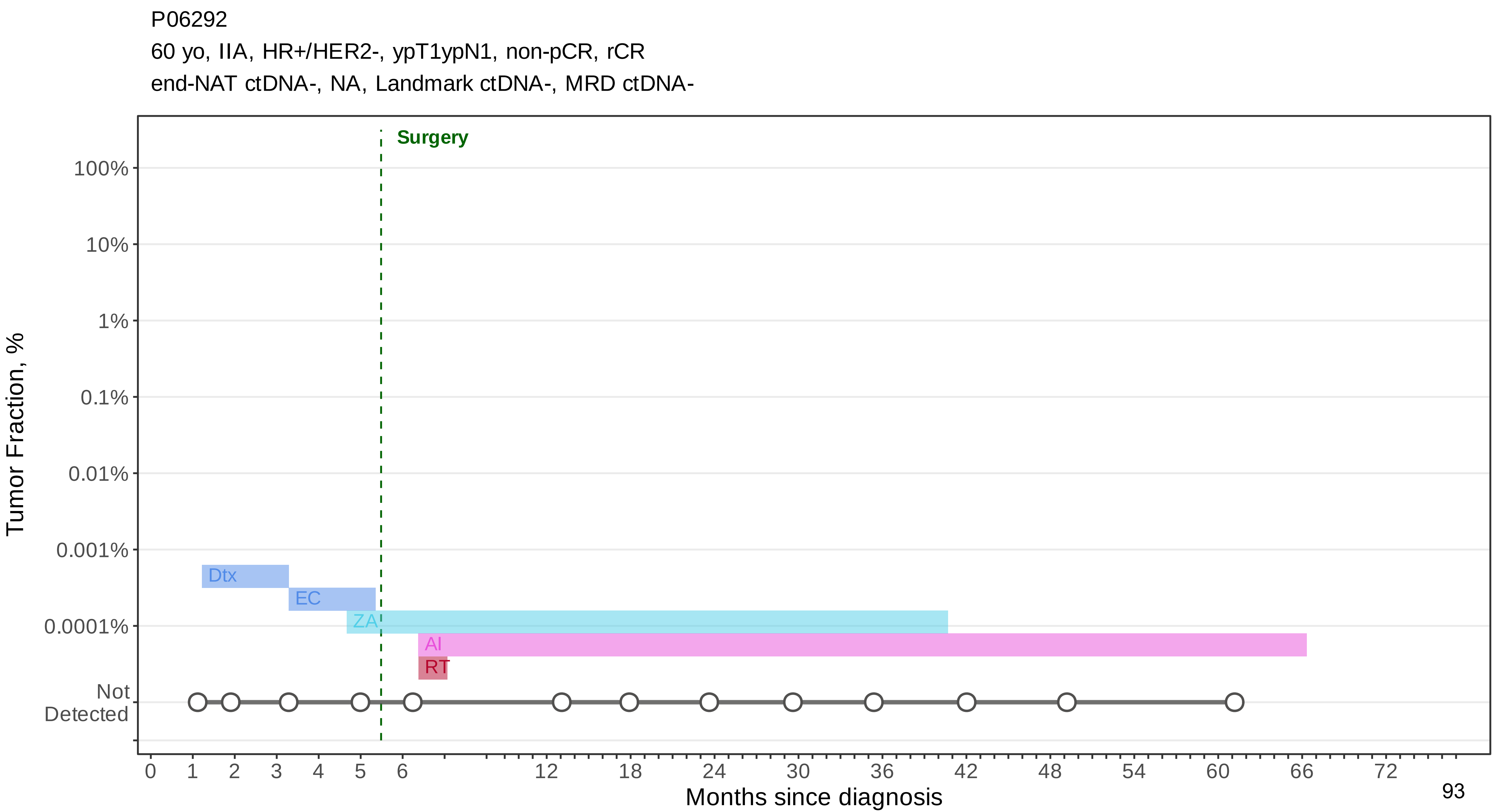

P06292
60 yo, IIA, HR+/HER2-, ypT1ypN1, non-pCR, rCR
end-NAT ctDNA-, NA, Landmark ctDNA-, MRD ctDNA-
Surgery
Tumor Fraction, %
100%
10%
1%
0.1%
0.01%
0.001%
0.0001%
Not Detected
Dtx
EC
ZA
AI
RT
0 1 2 3 4 5 6 12 18 24 30 36 42 48 54 60 66 72
Months since diagnosis

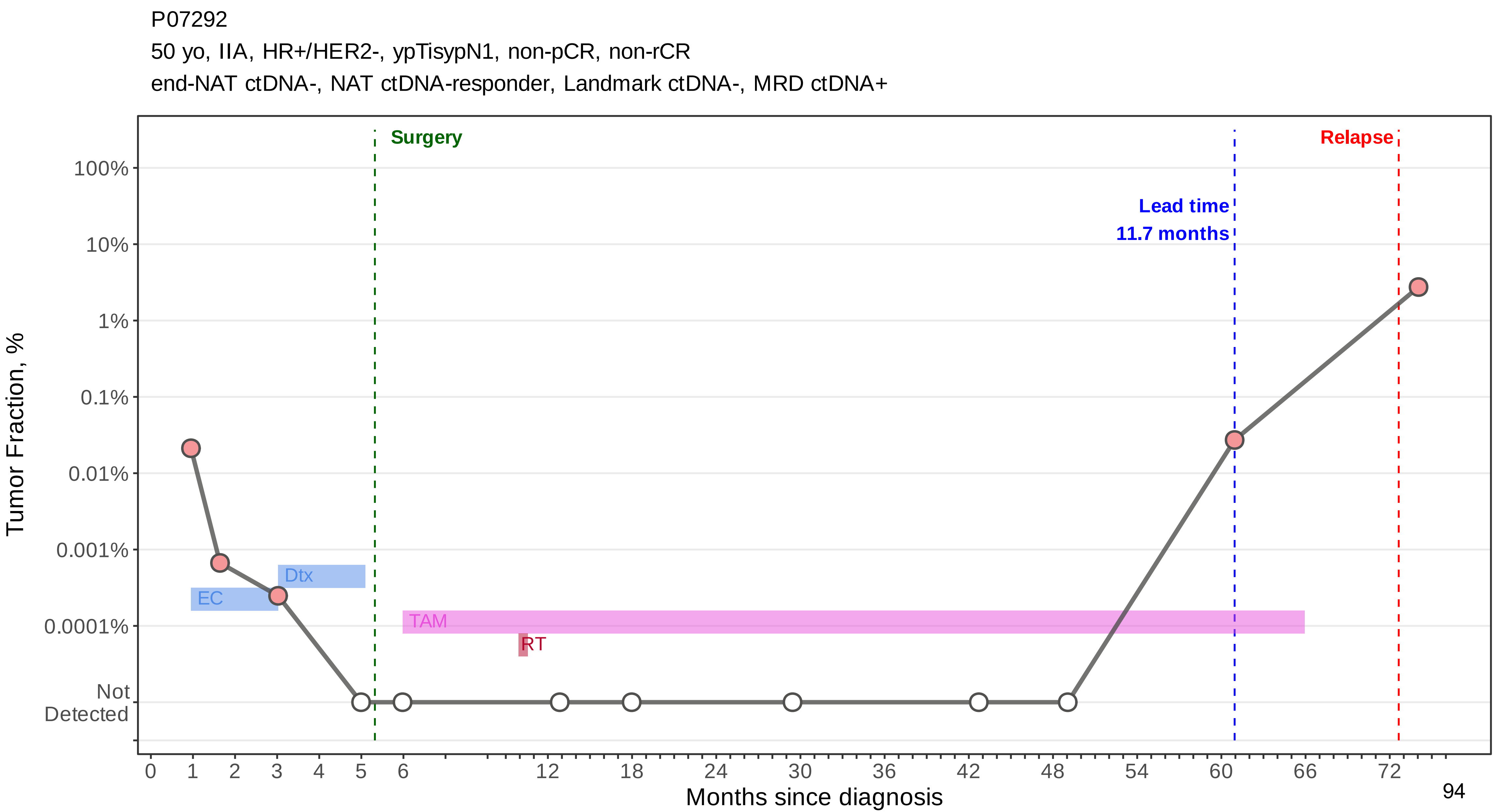
P07292
50 yo, IIA, HR+/HER2-, ypTisypN1, non-pCR, non-rCR
end-NAT ctDNA-, NAT ctDNA-responder, Landmark ctDNA-, MRD ctDNA+
Surgery
Relapse
Lead time
11.7 months
100%
10%
1%
0.1%
0.01%
0.001%
0.0001%
Not Detected
Tumor Fraction, %
Dtx
EC
TAM
RT
0 1 2 3 4 5 6 12 18 24 30 36 42 48 54 60 66 72
Months since diagnosis

P00392
65 yo, IIB, HER2+, HR+, ypT1ypN1, non-pCR, non-rCR
end-NAT ctDNA-, NAT ctDNA-responder, Landmark ctDNA-, MRD ctDNA-

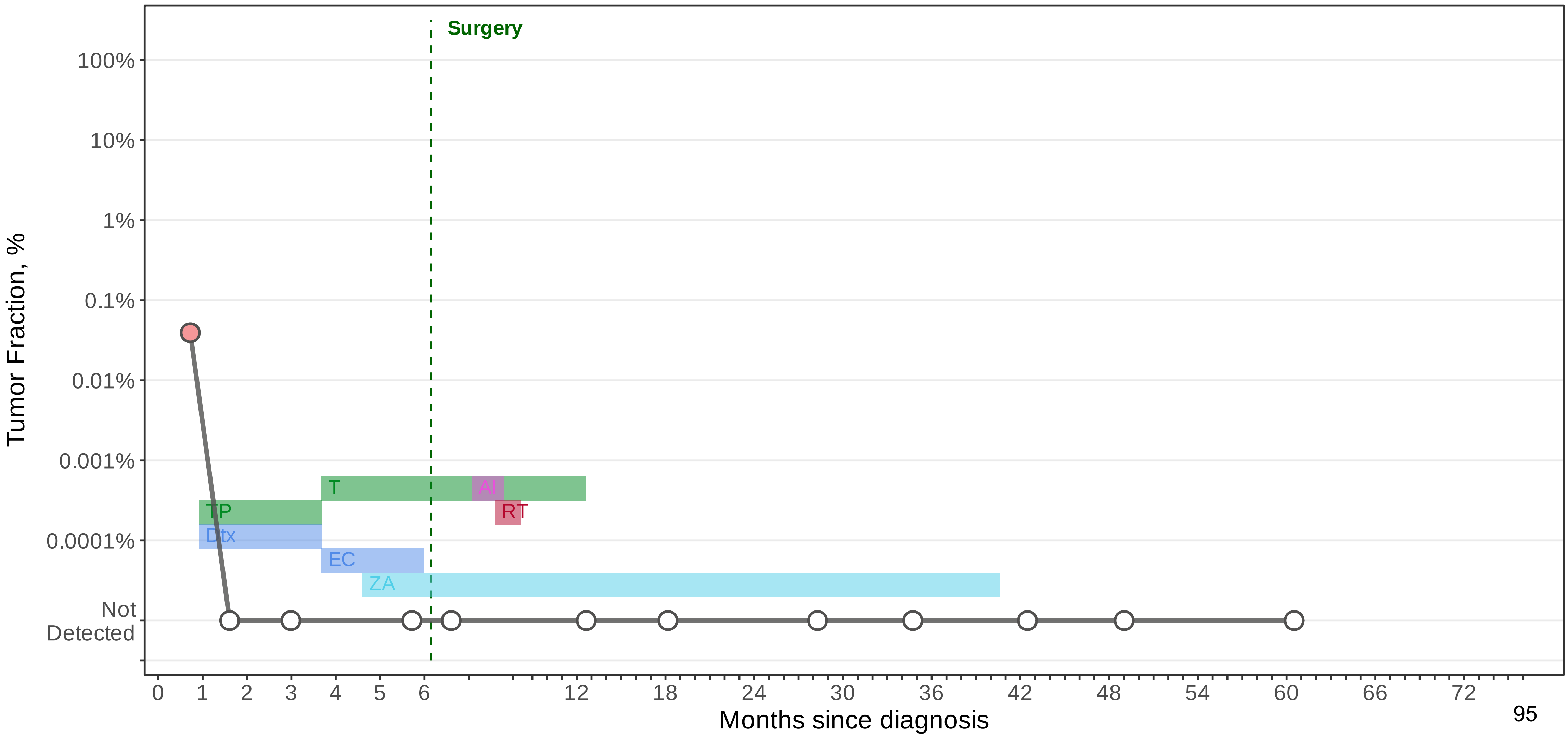

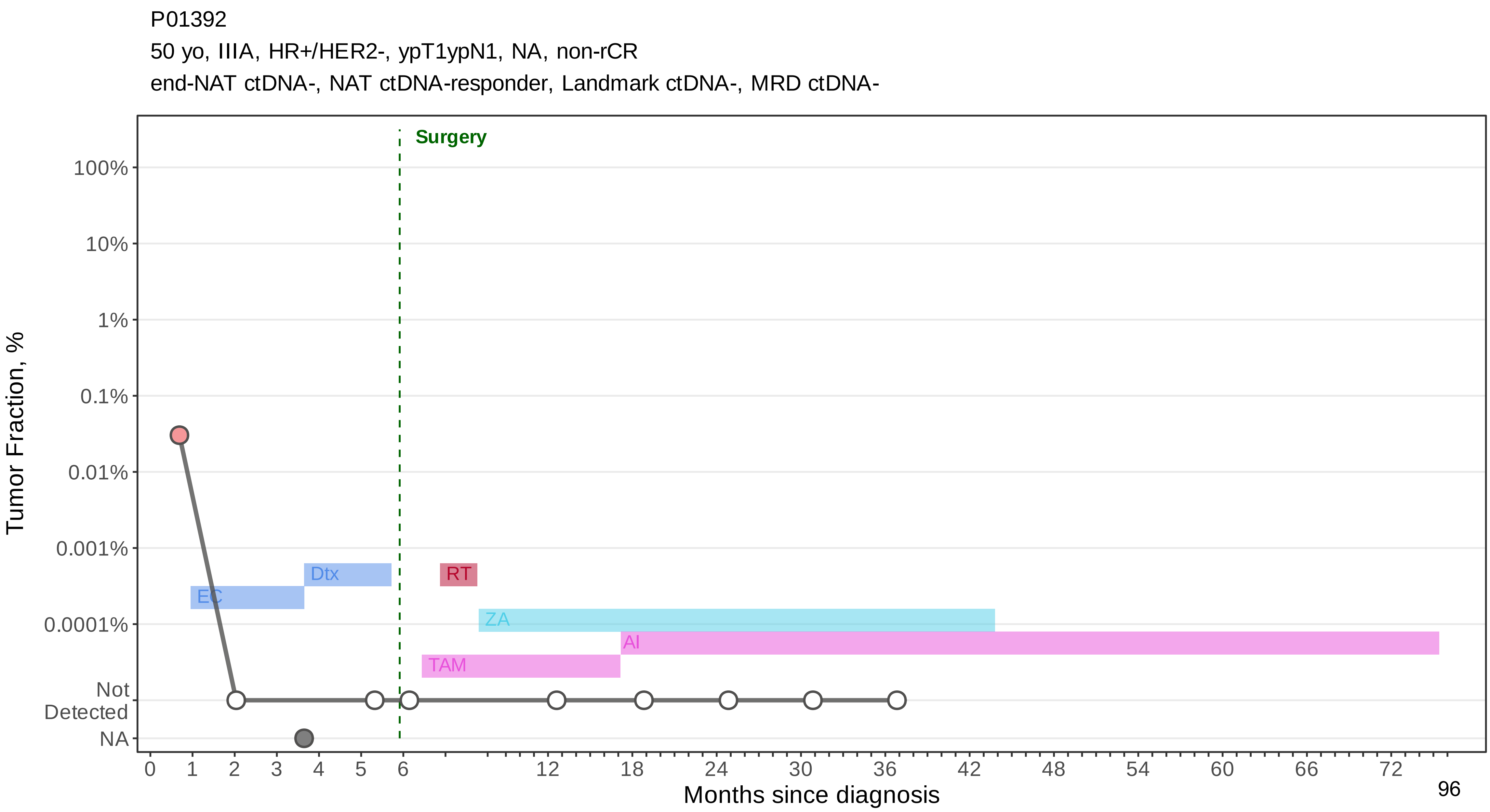
P01392
50 yo, IIIA, HR+/HER2-, ypT1ypN1, NA, non-rCR
end-NAT ctDNA-, NAT ctDNA-responder, Landmark ctDNA-, MRD ctDNA-
Surgery
100%
10%
1%
0.1%
0.01%
0.001%
0.0001%
Not Detected
NA
Tumor Fraction, %
Dtx
RT
EC
ZA
AI
TAM
0
1
2
3
4
5
6
12
18
24
30
36
42
48
54
60
66
72
Months since diagnosis

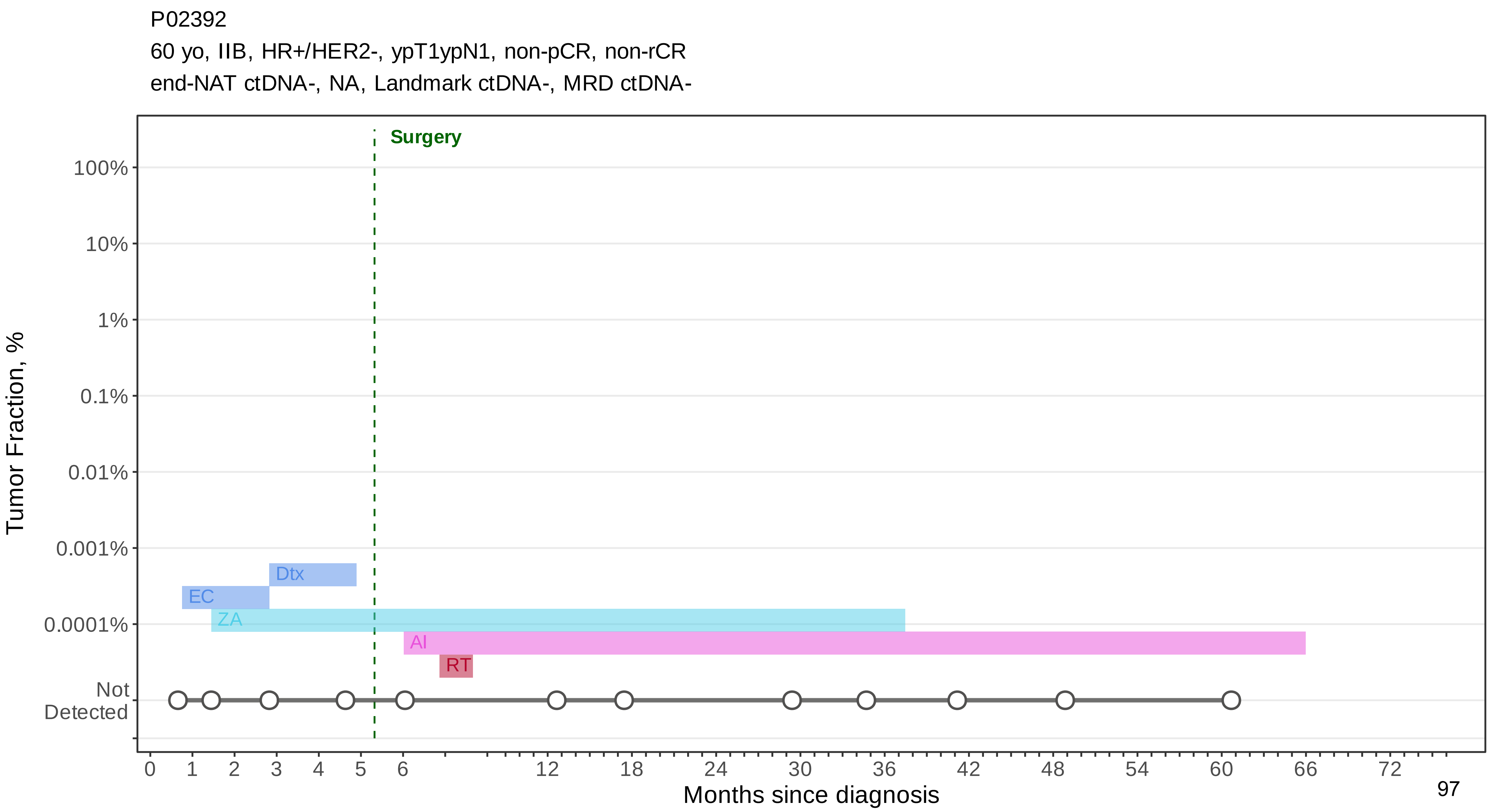
P02392
60 yo, IIB, HR+/HER2-, ypT1ypN1, non-pCR, non-rCR
end-NAT ctDNA-, NA, Landmark ctDNA-, MRD ctDNA-
Surgery
100%
10%
1%
0.1%
0.01%
0.001%
0.0001%
Not Detected
Tumor Fraction, %
Dtx
EC
ZA
AI
RT
0 1 2 3 4 5 6 12 18 24 30 36 42 48 54 60 66 72
Months since diagnosis

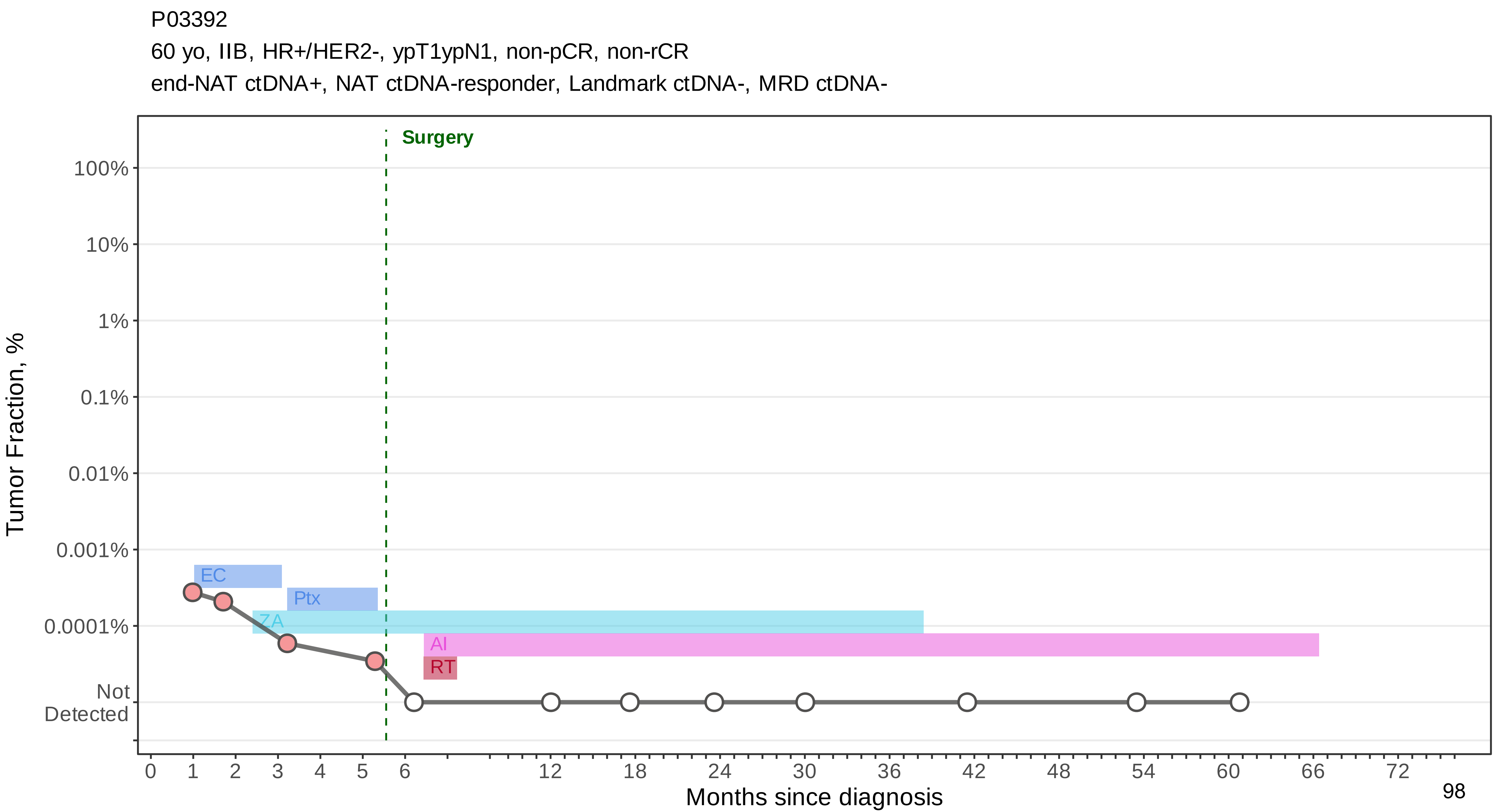
P03392
60 yo, IIB, HR+/HER2-, ypT1ypN1, non-pCR, non-rCR
end-NAT ctDNA+, NAT ctDNA-responder, Landmark ctDNA-, MRD ctDNA-
Surgery
Tumor Fraction, %
100%
10%
1%
0.1%
0.01%
0.001%
0.0001%
Not Detected
EC
Ptx
ZA
AI
RT
0
1
2
3
4
5
6
12
18
24
30
36
42
48
54
60
66
72
Months since diagnosis

P04392
55 yo, IIA, TNBC, ypTXypN0, non-pCR, rCR
end-NAT ctDNA-, NAT ctDNA-responder, Landmark ctDNA-, MRD ctDNA-

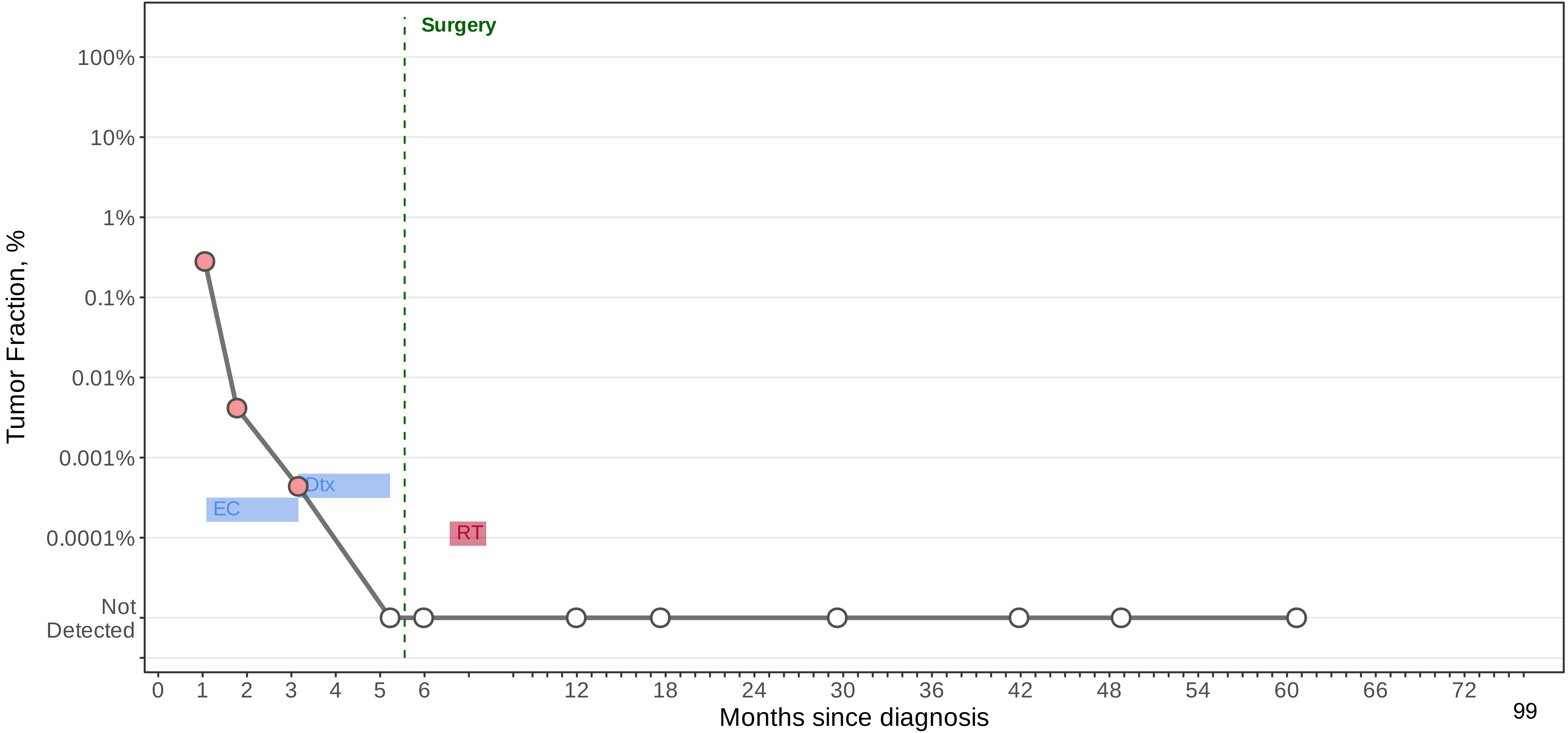

P05392
50 yo, IIB, HR+/HER2-, ypT1ypN1, non-pCR, rCR
end-NAT ctDNA-, NA, Landmark ctDNA-, MRD ctDNA-

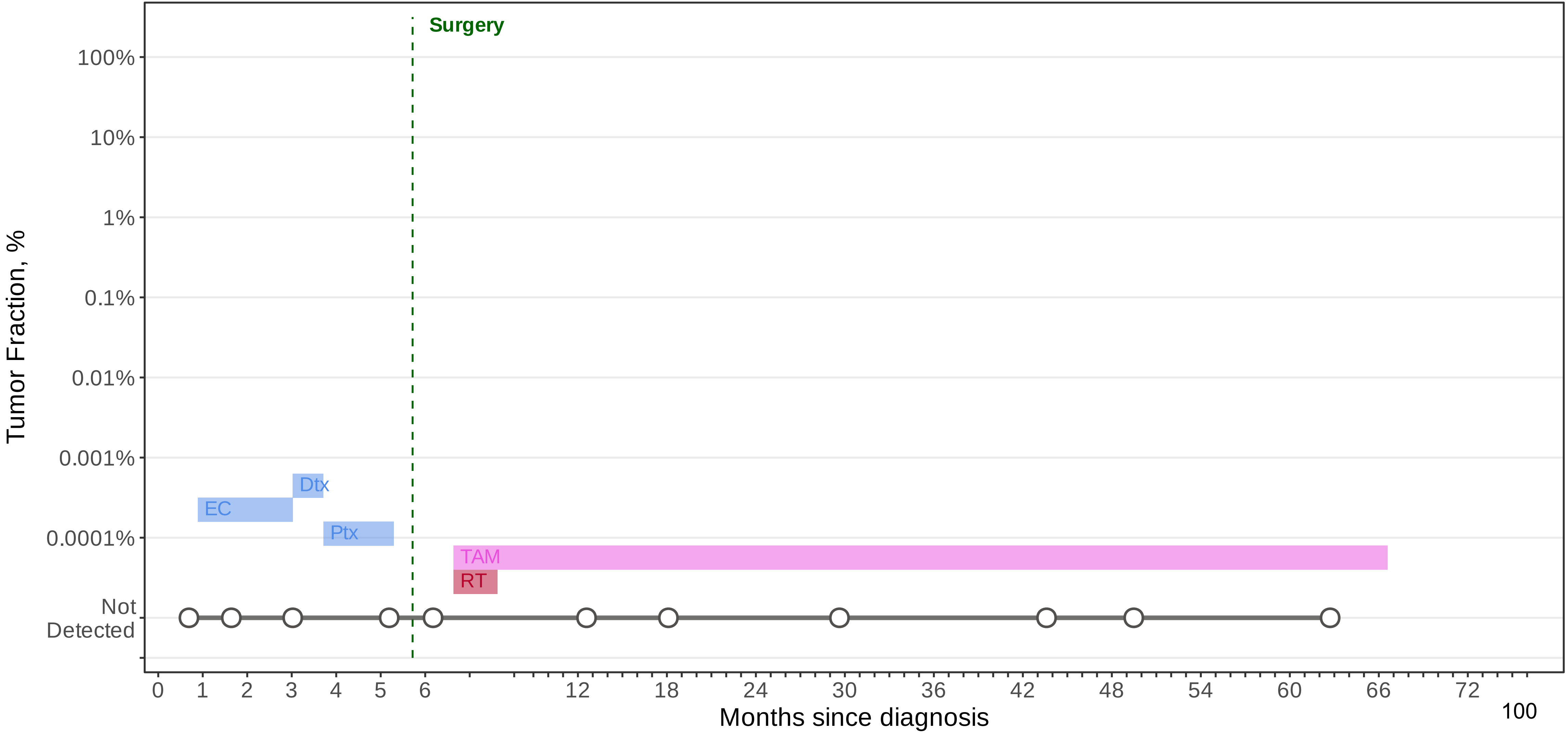

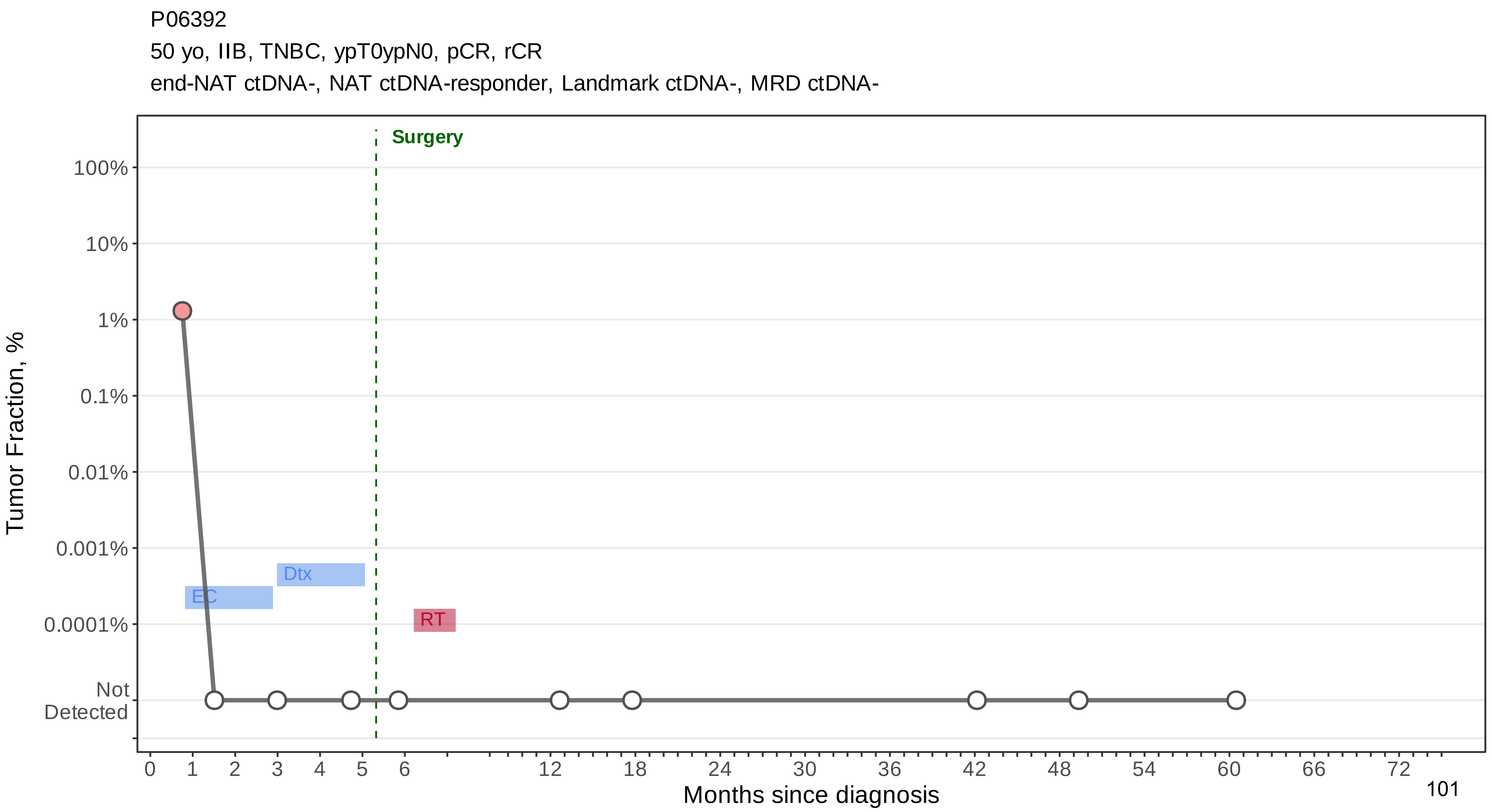
P06392
50 yo, IIB, TNBC, ypT0ypN0, pCR, rCR
end-NAT ctDNA-, NAT ctDNA-responder, Landmark ctDNA-, MRD ctDNA-
Surgery
100%
10%
1%
0.1%
0.01%
0.001%
0.0001%
Not Detected
Tumor Fraction, %
Dtx
EC
RT
0 1 2 3 4 5 6 12 18 24 30 36 42 48 54 60 66 72
Months since diagnosis

P07392

60 yo, IIB, TNBC, ypT1ypN0, non-pCR, non-rCR

end-NAT ctDNA-, NAT ctDNA-responder, Landmark ctDNA-, MRD ctDNA-

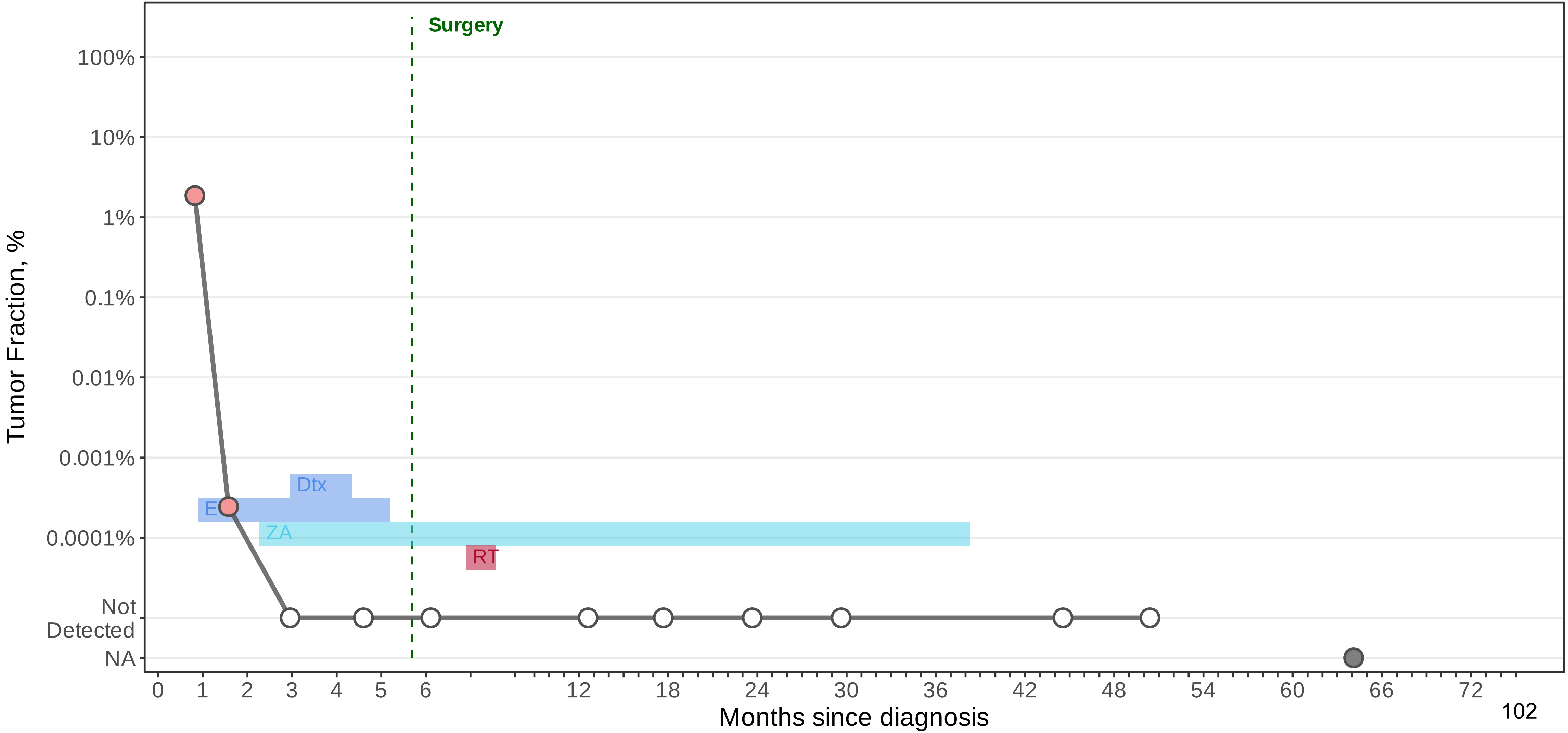

P08392
30 yo, IIB, HR+/HER2-, ypT2ypN1, non-pCR, non-rCR
end-NAT ctDNA+, NAT ctDNA-responder, Landmark ctDNA-, MRD ctDNA-

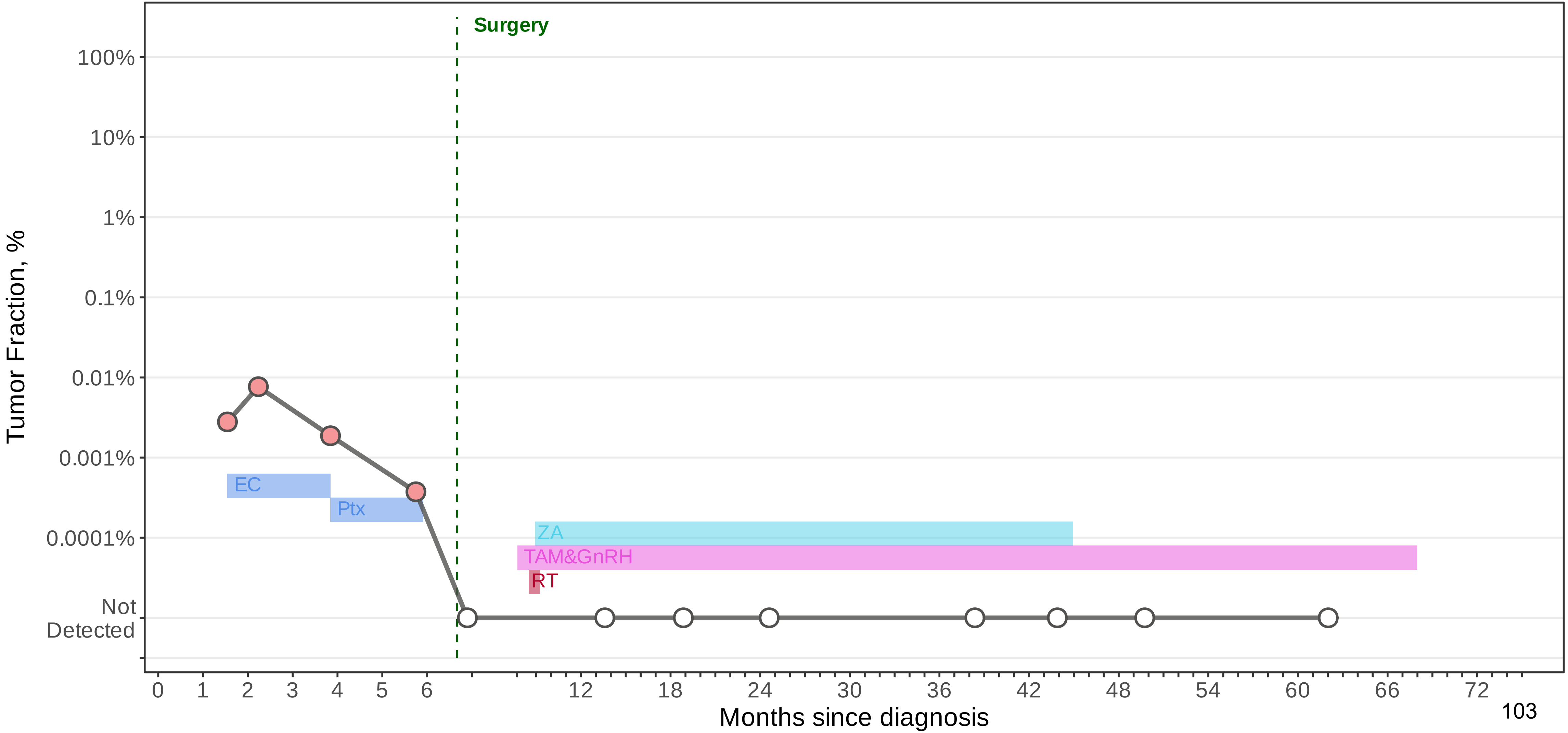

P00492
40 yo, IIA, TNBC, ypT1ypN0, non-pCR, non-rCR
end-NAT ctDNA+, NAT ctDNA-responder, Landmark ctDNA-, MRD ctDNA-

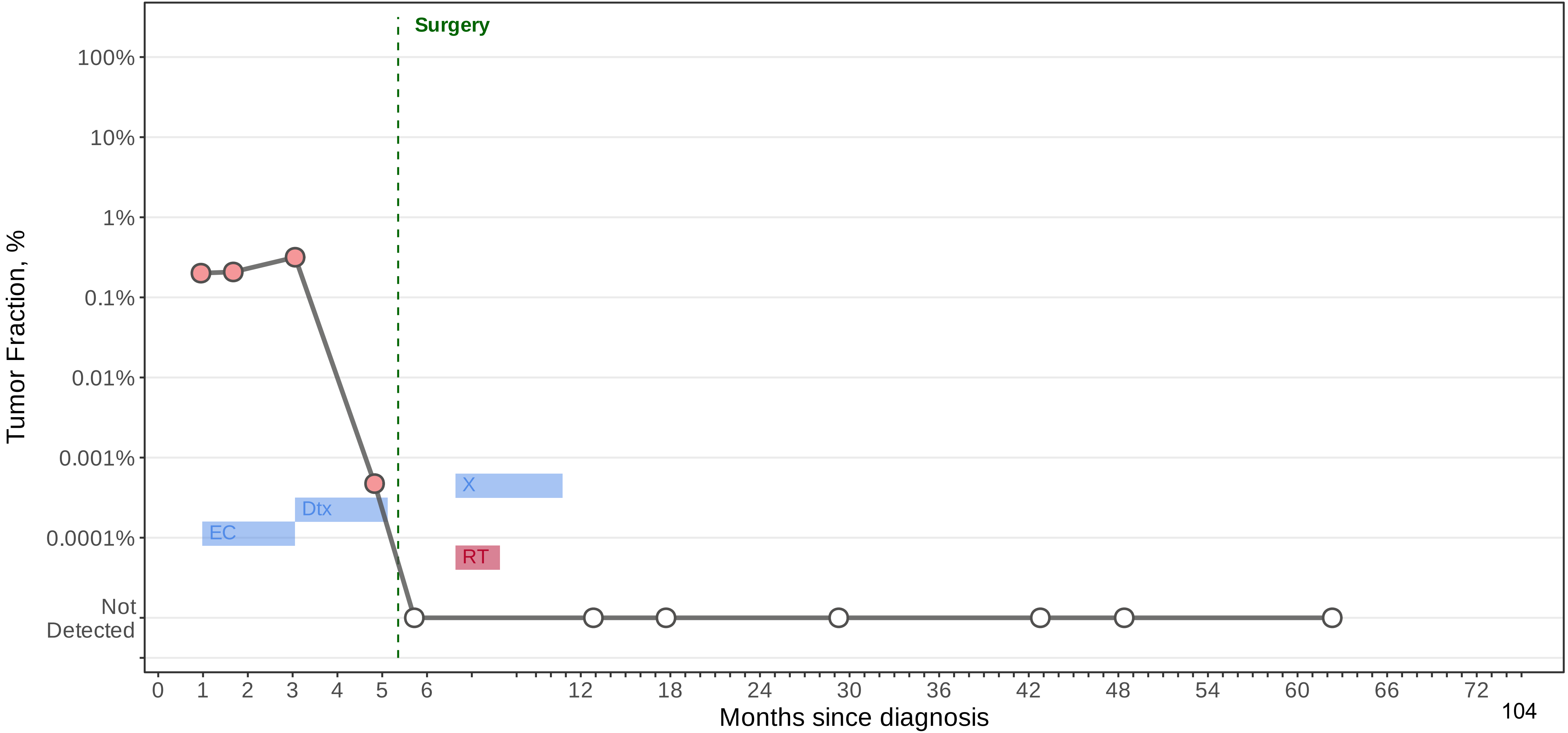

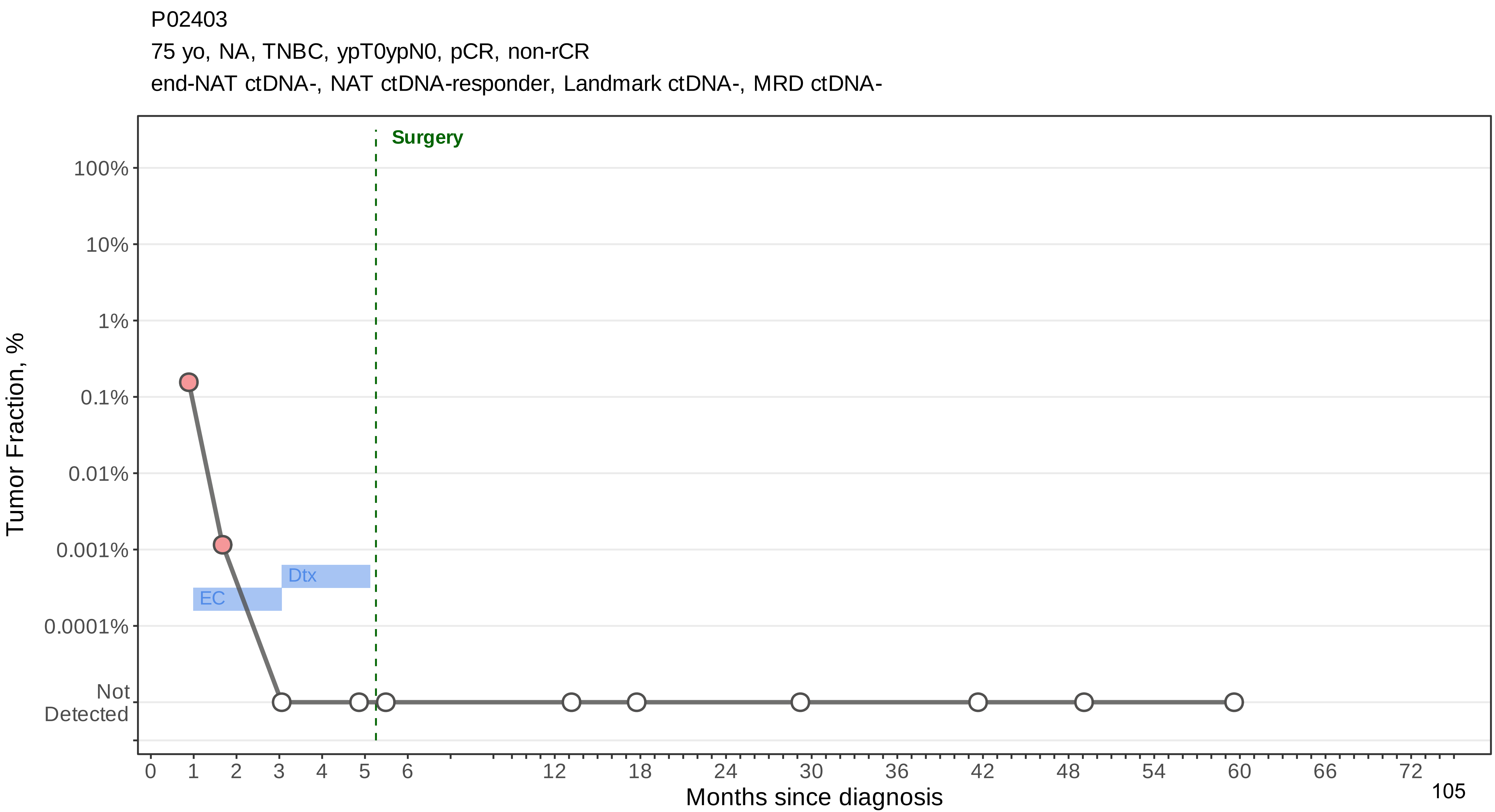

P02403
75 yo, NA, TNBC, ypT0ypN0, pCR, non-rCR
end-NAT ctDNA-, NAT ctDNA-responder, Landmark ctDNA-, MRD ctDNA-
Surgery
Tumor Fraction, %
100%
10%
1%
0.1%
0.01%
0.001%
0.0001%
Not Detected
Dtx
EC
0
1
2
3
4
5
6
12
18
24
30
36
42
48
54
60
66
72
Months since diagnosis

P05403

55 yo, IIA, HER2+, HR+, ypT1ypN0, non-pCR, rCR

end-NAT ctDNA-, NAT ctDNA-responder, Landmark ctDNA-, MRD ctDNA-

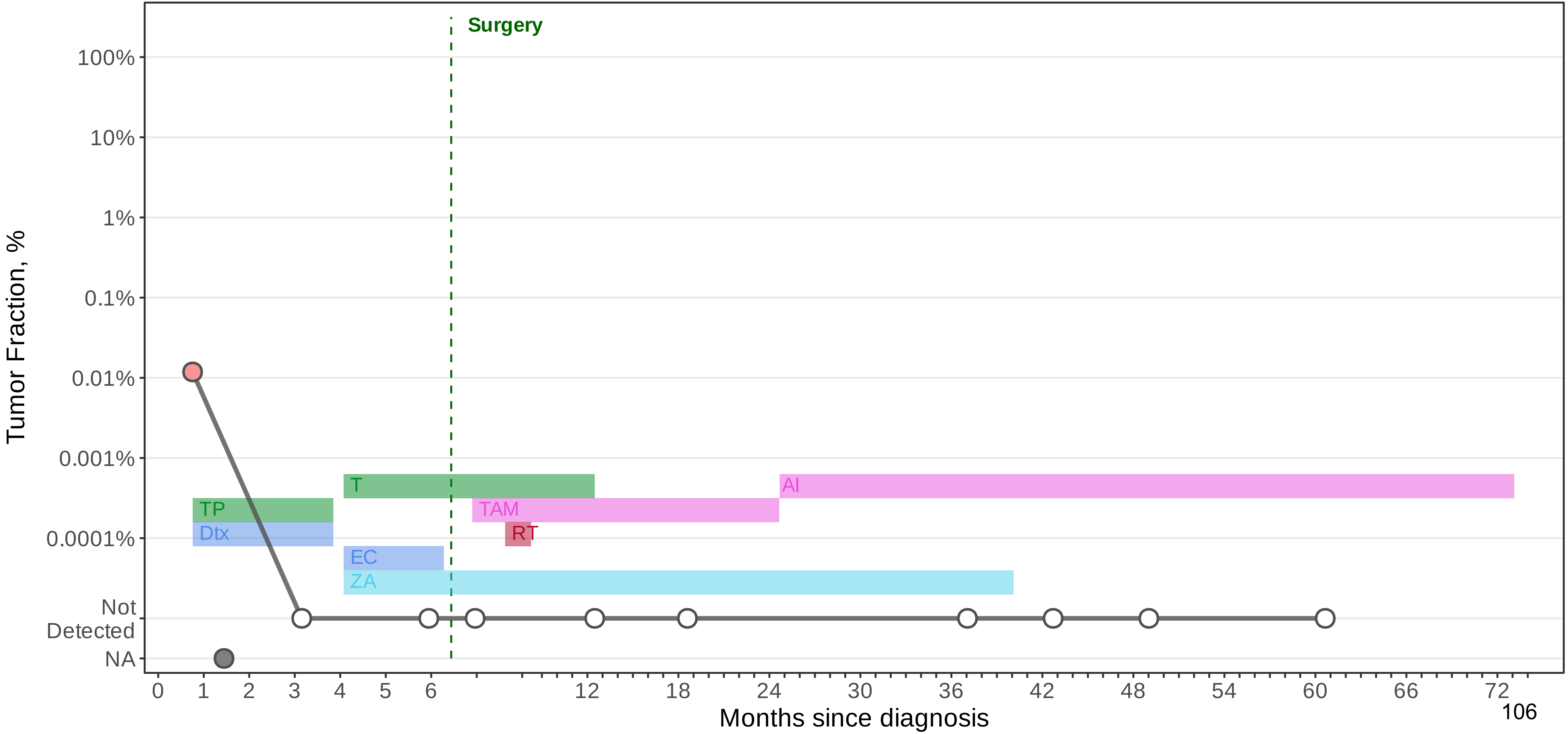

P06403
75 yo, IIB, HR+/HER2-, ypT2ypN3, non-pCR, non-rCR
end-NAT ctDNA+, NAT ctDNA-non-responder, Landmark ctDNA-, MRD ctDNA+

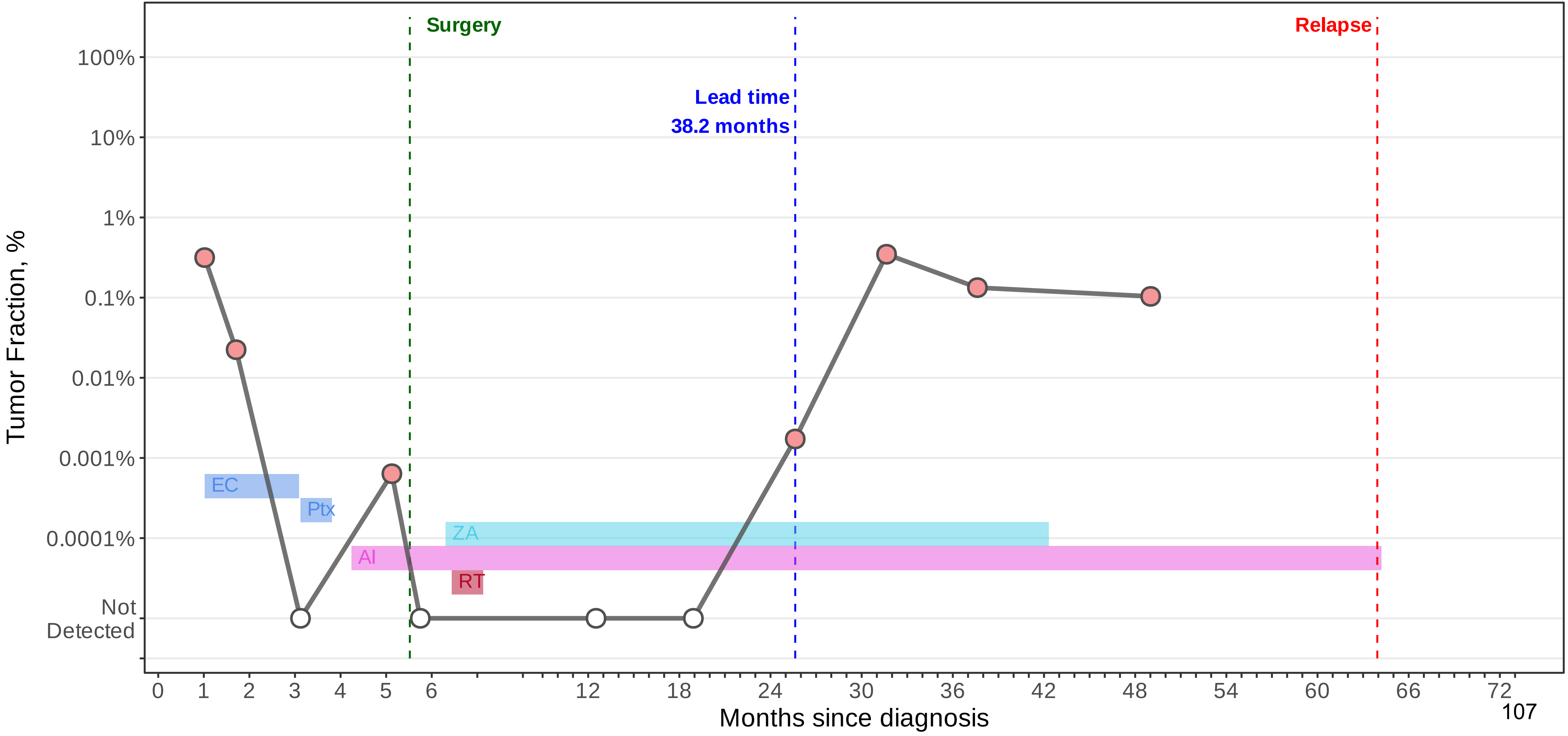

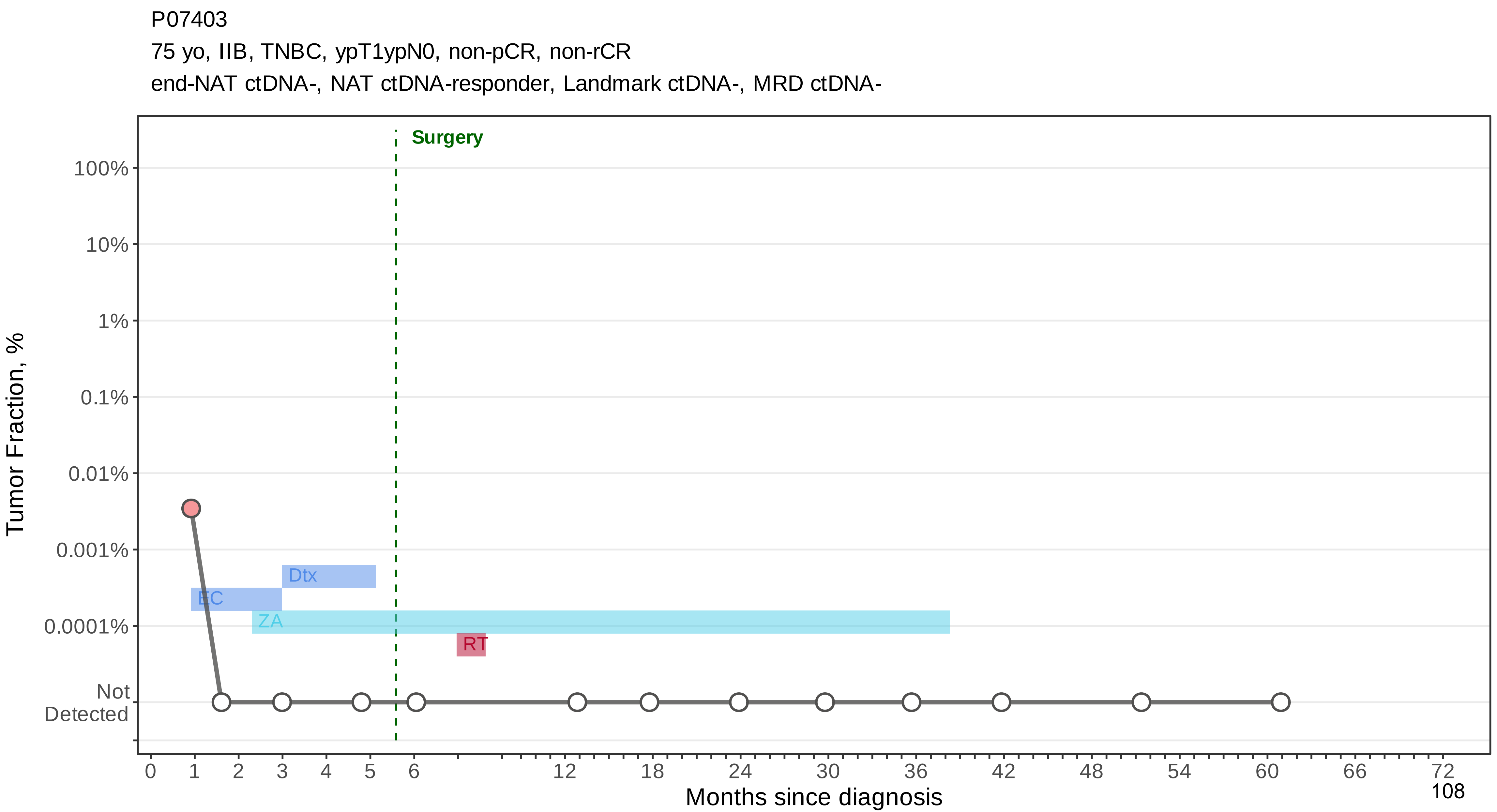
P07403
75 yo, IIB, TNBC, ypT1ypN0, non-pCR, non-rCR
end-NAT ctDNA-, NAT ctDNA-responder, Landmark ctDNA-, MRD ctDNA-
Surgery
100%
10%
1%
0.1%
0.01%
0.001%
0.0001%
Not Detected
Tumor Fraction, %
Dtx
EC
ZA
RT
0 1 2 3 4 5 6 12 18 24 30 36 42 48 54 60 66 72
Months since diagnosis

P08403

65 yo, IIIA, HR+/HER2-, ypTXypN2, non-pCR, non-rCR

end-NAT ctDNA-, NAT ctDNA-responder, Landmark ctDNA-, MRD ctDNA+

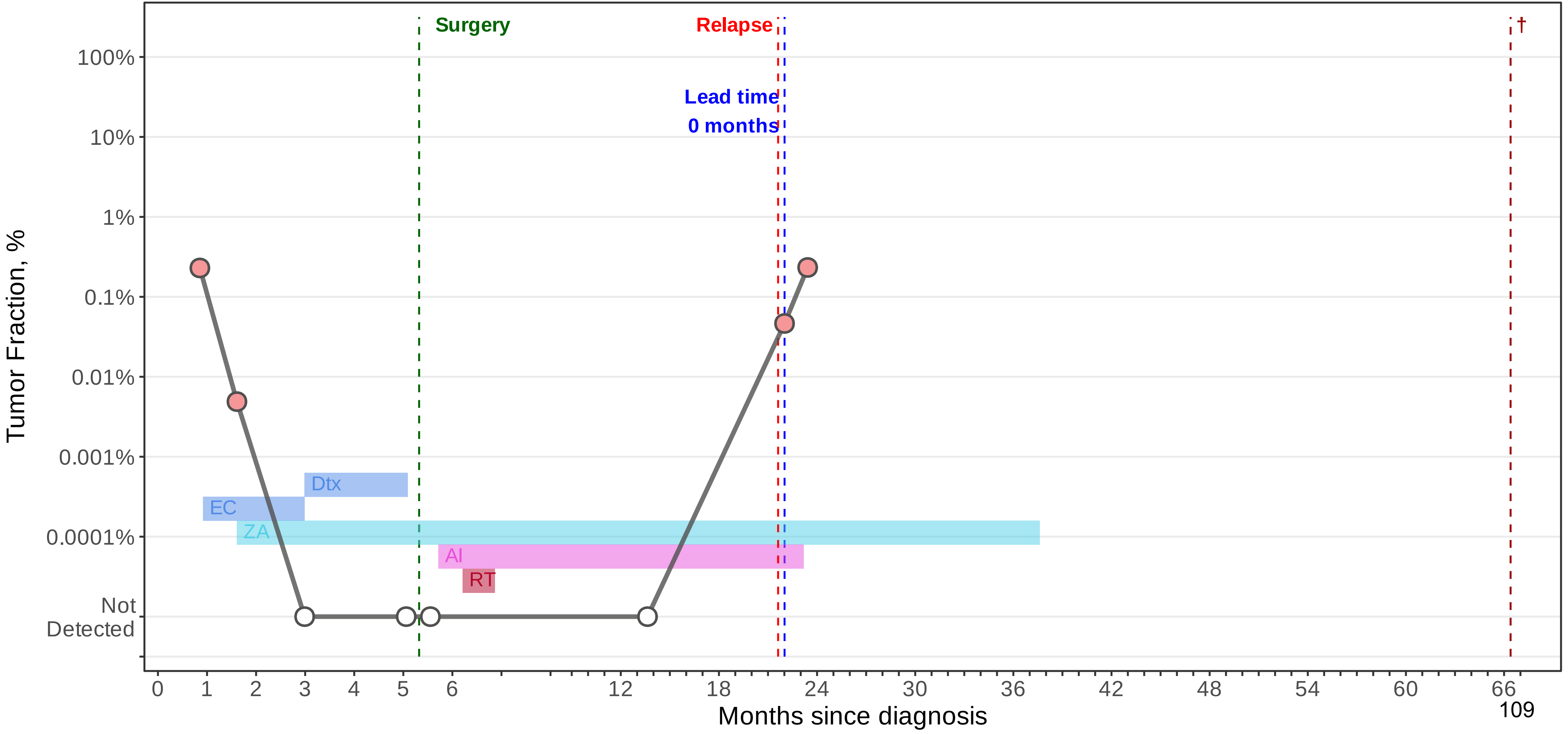

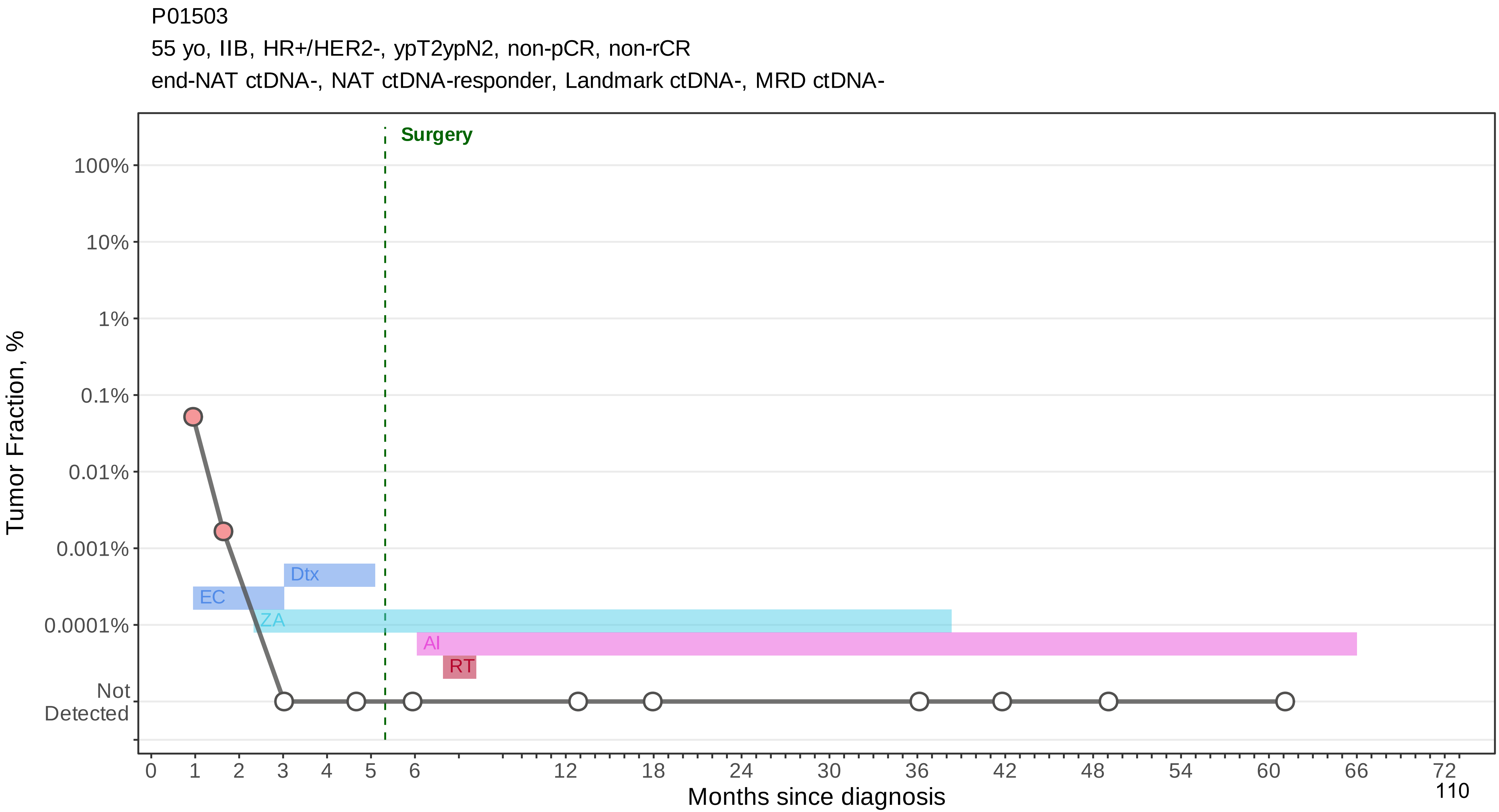

P01503
55 yo, IIB, HR+/HER2-, ypT2ypN2, non-pCR, non-rCR
end-NAT ctDNA-, NAT ctDNA-responder, Landmark ctDNA-, MRD ctDNA-
Surgery
Tumor Fraction, %
100%
10%
1%
0.1%
0.01%
0.001%
0.0001%
Not Detected
Dtx
EC
ZA
AI
RT
0 1 2 3 4 5 6 12 18 24 30 36 42 48 54 60 66 72
Months since diagnosis

P02503
35 yo, IIIA, HER2+, HR+, ypT1ypN1, non-pCR, non-rCR
end-NAT ctDNA-, NAT ctDNA-responder, Landmark ctDNA-, MRD ctDNA-

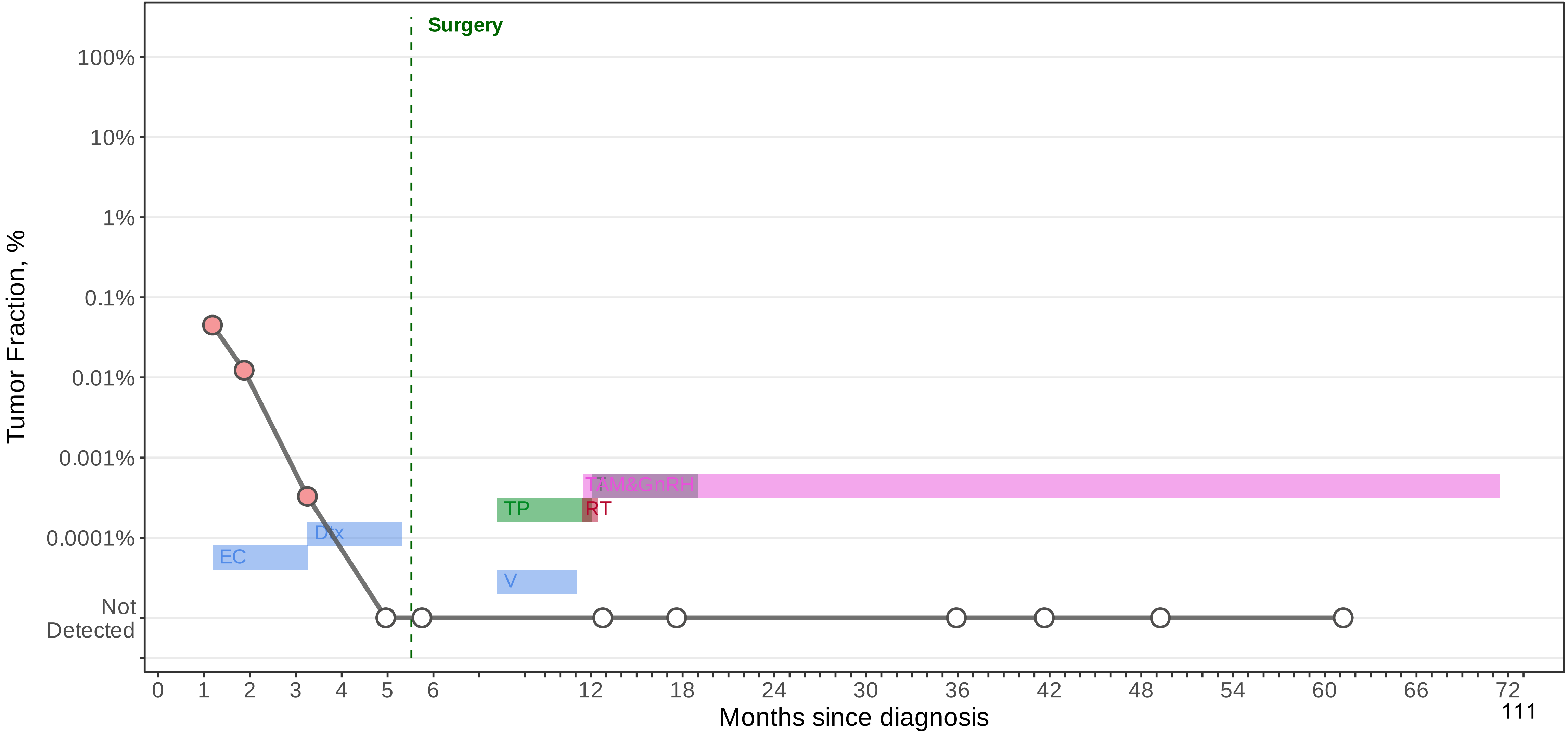

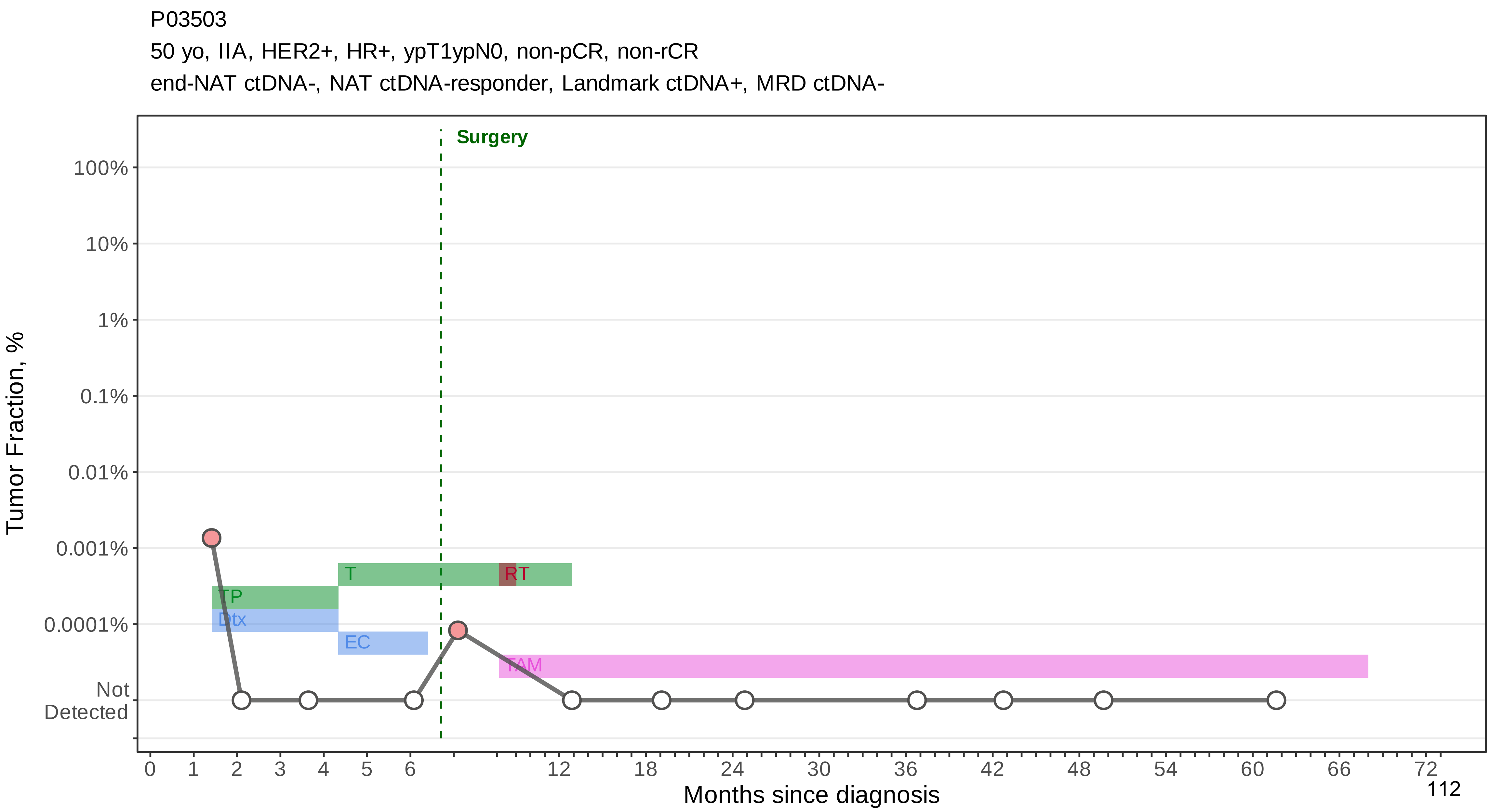

P03503
50 yo, IIA, HER2+, HR+, ypT1ypN0, non-pCR, non-rCR
end-NAT ctDNA-, NAT ctDNA-responder, Landmark ctDNA+, MRD ctDNA-
Surgery
100%
10%
1%
0.1%
0.01%
0.001%
0.0001%
Not Detected
Tumor Fraction, %
T
RT
TP
Dtx
EC
TAM
0 1 2 3 4 5 6 12 18 24 30 36 42 48 54 60 66 72
Months since diagnosis

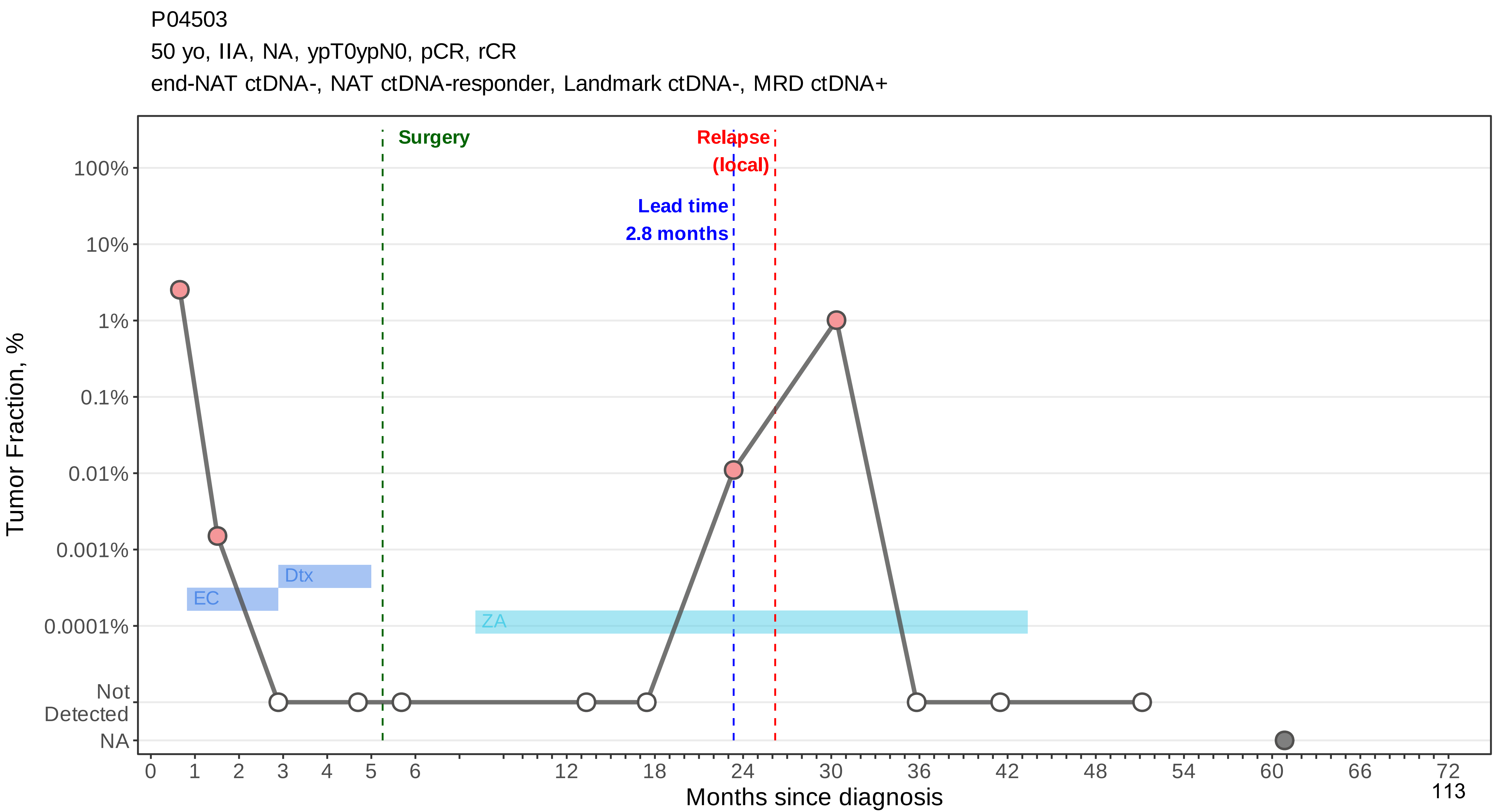
P04503
50 yo, IIA, NA, ypT0ypN0, pCR, rCR
end-NAT ctDNA-, NAT ctDNA-responder, Landmark ctDNA-, MRD ctDNA+
Surgery
Relapse
(local)
Lead time
2.8 months
100%
10%
1%
0.1%
0.01%
0.001%
0.0001%
Not Detected
NA
Dtx
EC
ZA
Tumor Fraction, %
0 1 2 3 4 5 6 12 18 24 30 36 42 48 54 60 66 72
Months since diagnosis

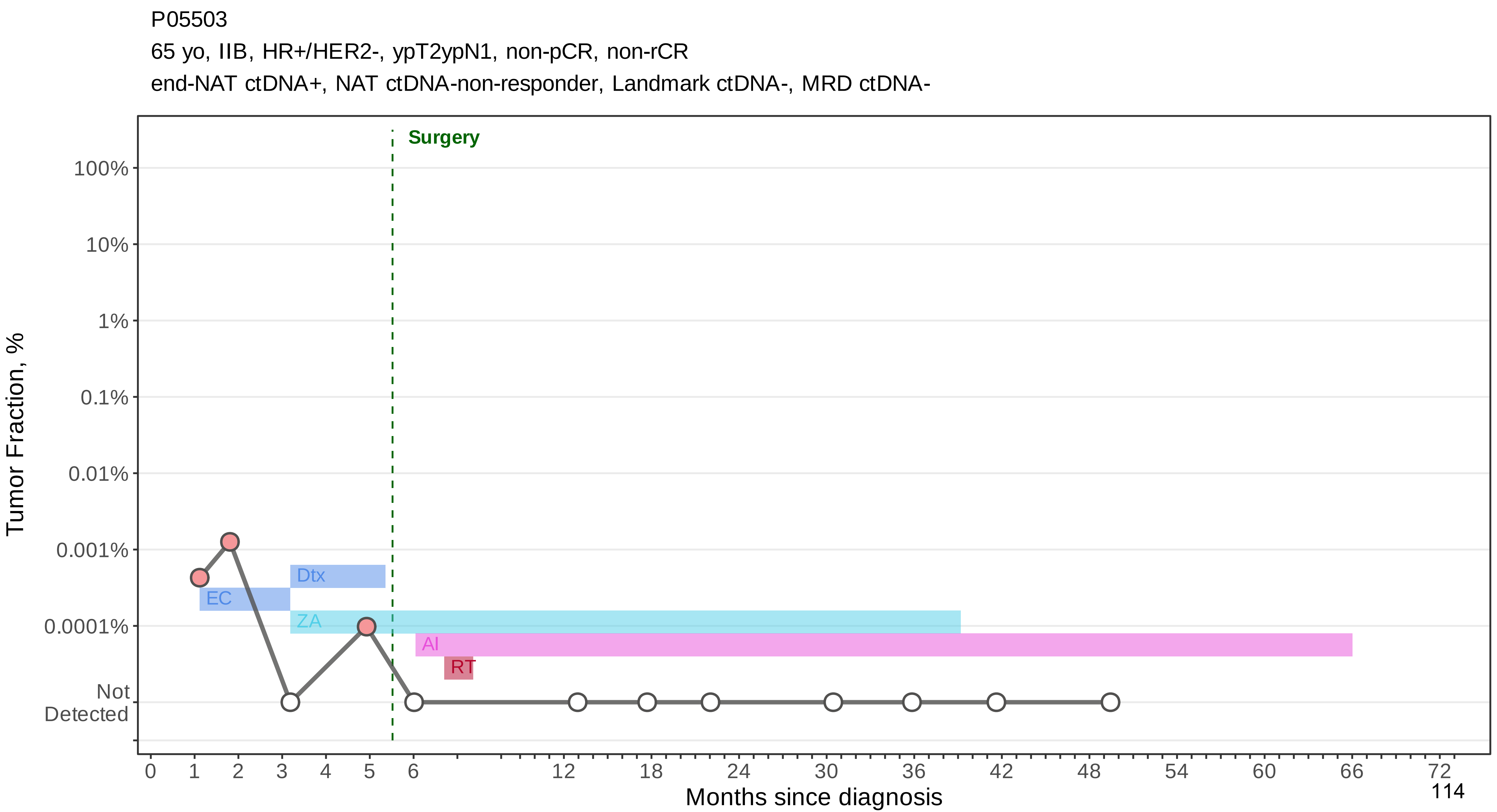

P05503
65 yo, IIB, HR+/HER2-, ypT2ypN1, non-pCR, non-rCR
end-NAT ctDNA+, NAT ctDNA-non-responder, Landmark ctDNA-, MRD ctDNA-
Surgery
Tumor Fraction, %
100%
10%
1%
0.1%
0.01%
0.001%
0.0001%
Not Detected
Dtx
EC
ZA
AI
RT
0
1
2
3
4
5
6
12
18
24
30
36
42
48
54
60
66
72
Months since diagnosis

P06503
55 yo, IIIA, HR+/HER2-, ypT2ypN1, non-pCR, non-rCR
end-NAT ctDNA-, NAT ctDNA-responder, Landmark ctDNA-, MRD ctDNA-

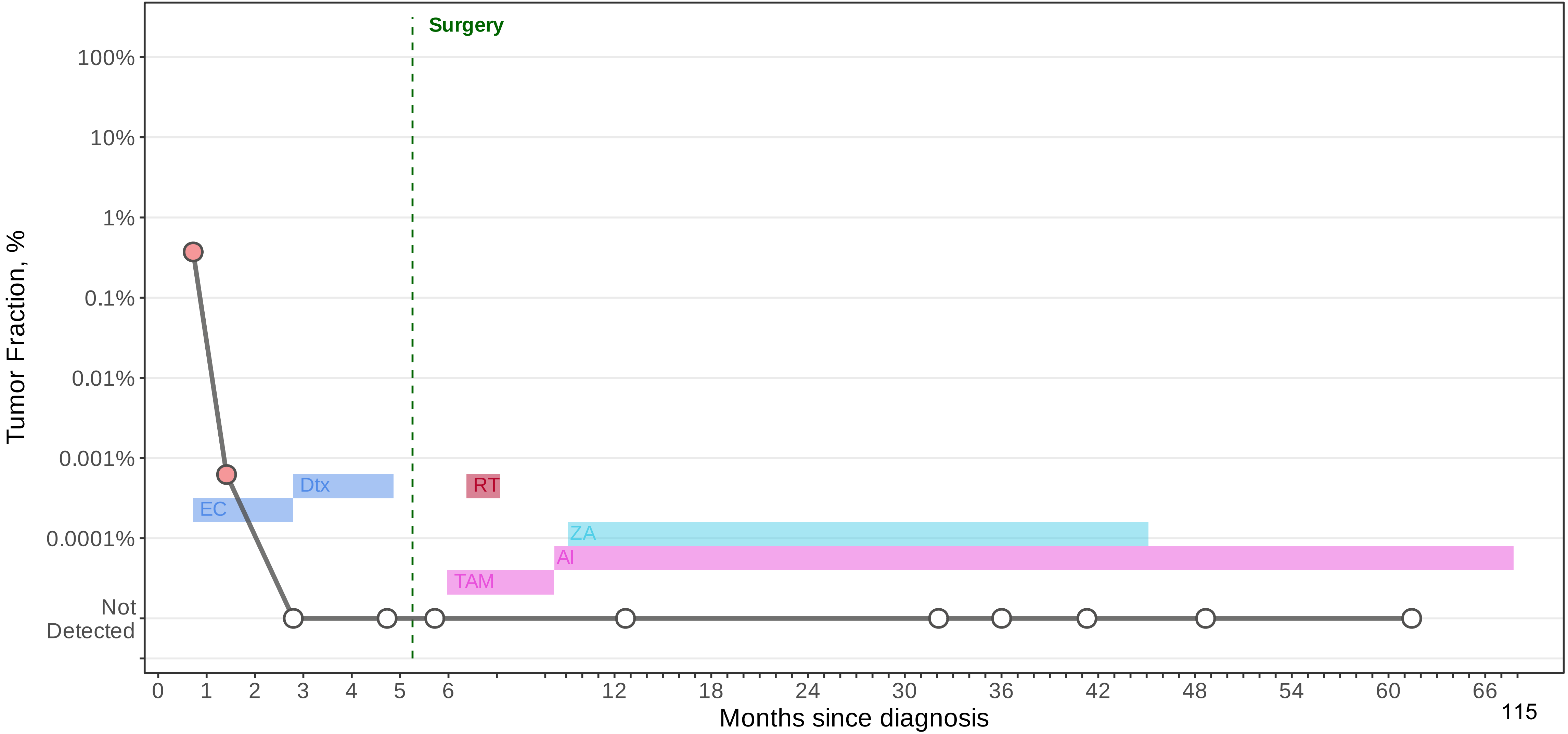

P08503
75 yo, IIB, HR+/HER2-, ypT2ypN3, non-pCR, non-rCR
end-NAT ctDNA-, NAT ctDNA-responder, Landmark ctDNA-, MRD ctDNA-

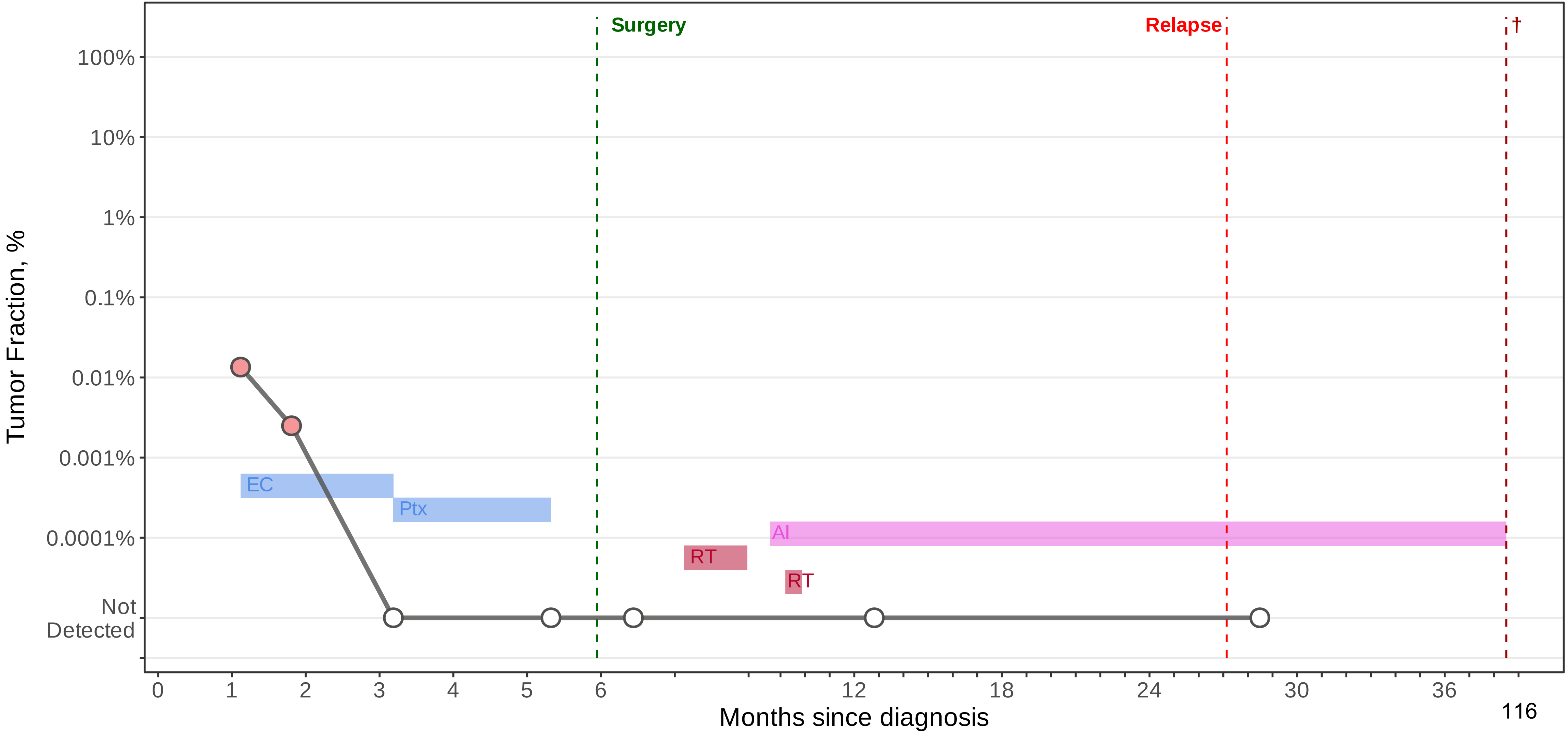

P09503
60 yo, IIB, TNBC, ypT0ypN0, pCR, rCR
end-NAT ctDNA-, NAT ctDNA-responder, Landmark ctDNA+, MRD ctDNA+

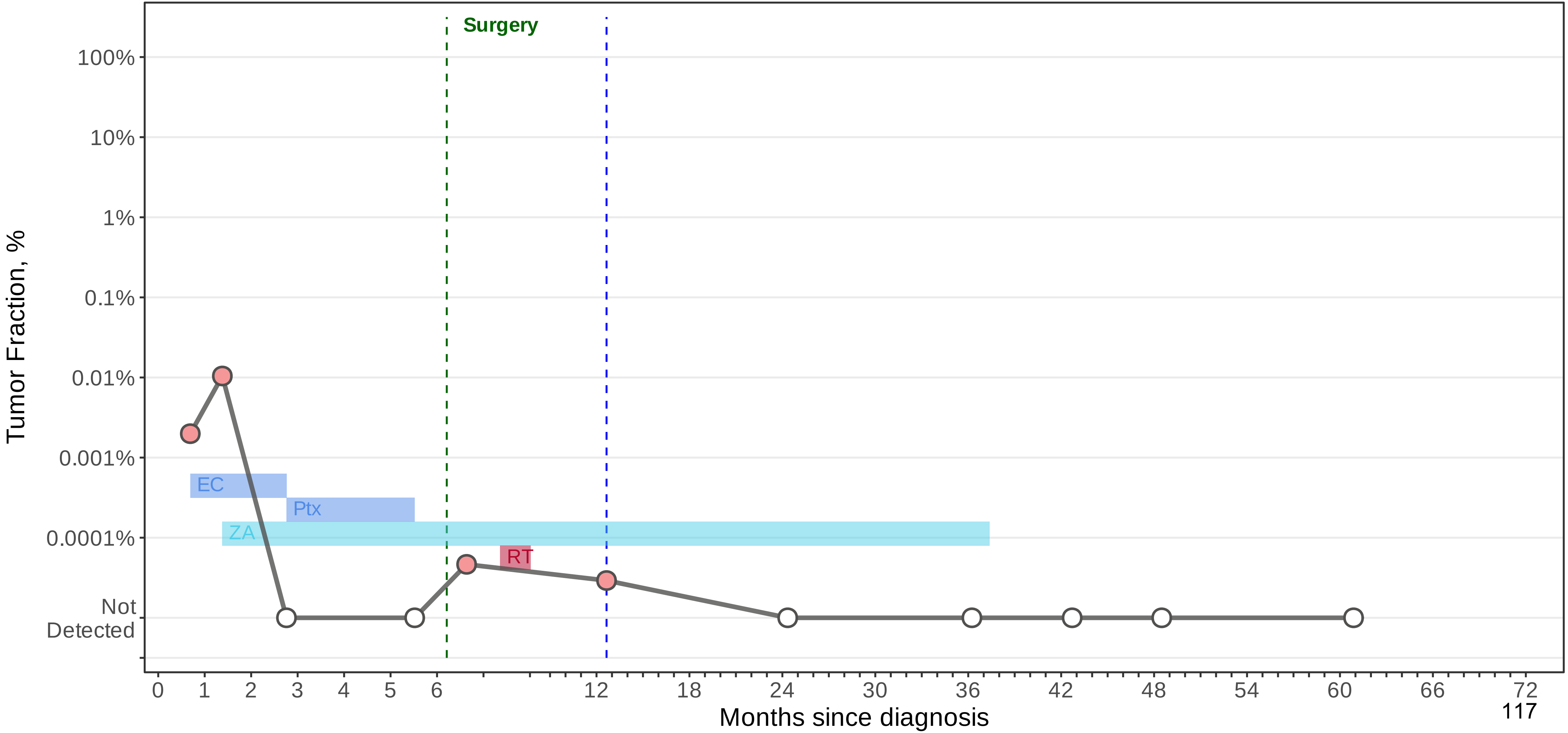

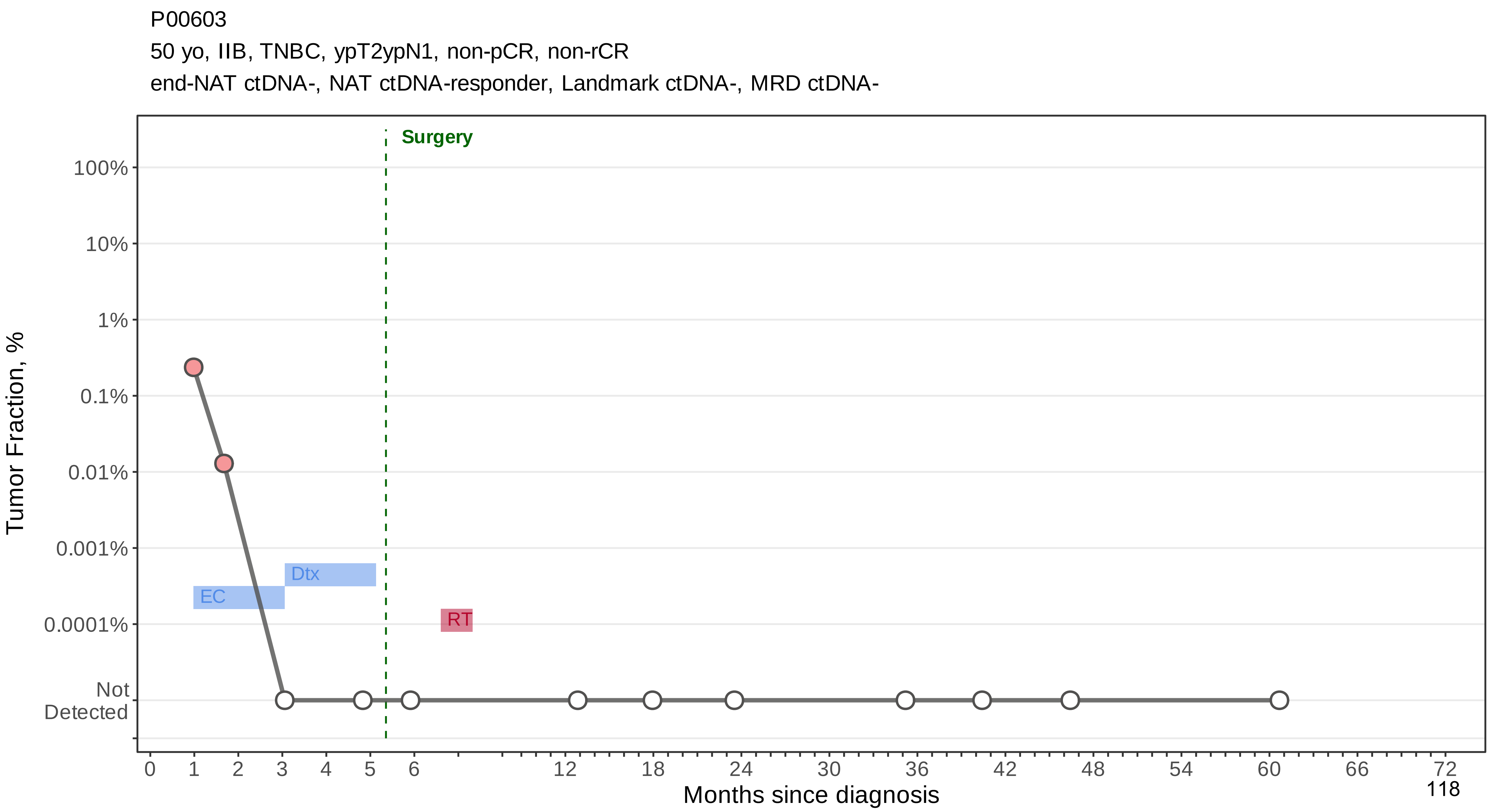
P00603
50 yo, IIB, TNBC, ypT2ypN1, non-pCR, non-rCR
end-NAT ctDNA-, NAT ctDNA-responder, Landmark ctDNA-, MRD ctDNA-
Surgery
Tumor Fraction, %
100%
10%
1%
0.1%
0.01%
0.001%
0.0001%
Not Detected
Dtx
EC
RT
0 1 2 3 4 5 6 12 18 24 30 36 42 48 54 60 66 72
Months since diagnosis

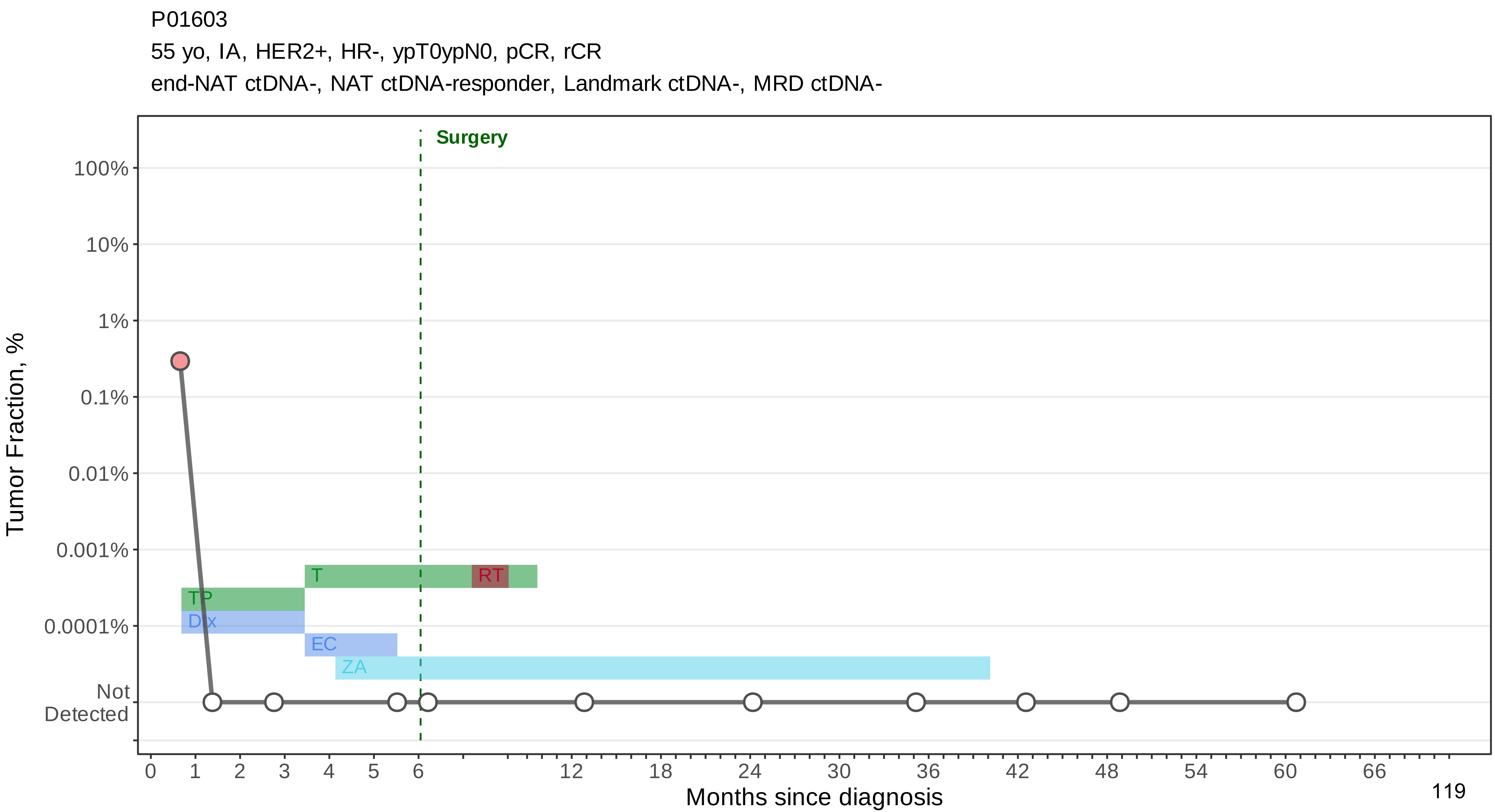
P01603
55 yo, IA, HER2+, HR-, ypT0ypN0, pCR, rCR
end-NAT ctDNA-, NAT ctDNA-responder, Landmark ctDNA-, MRD ctDNA-
Surgery
100%
10%
1%
0.1%
0.01%
0.001%
0.0001%
Not Detected
Tumor Fraction, %
T
RT
TP
Dtx
EC
ZA
0
1
2
3
4
5
6
12
18
24
30
36
42
48
54
60
66
Months since diagnosis

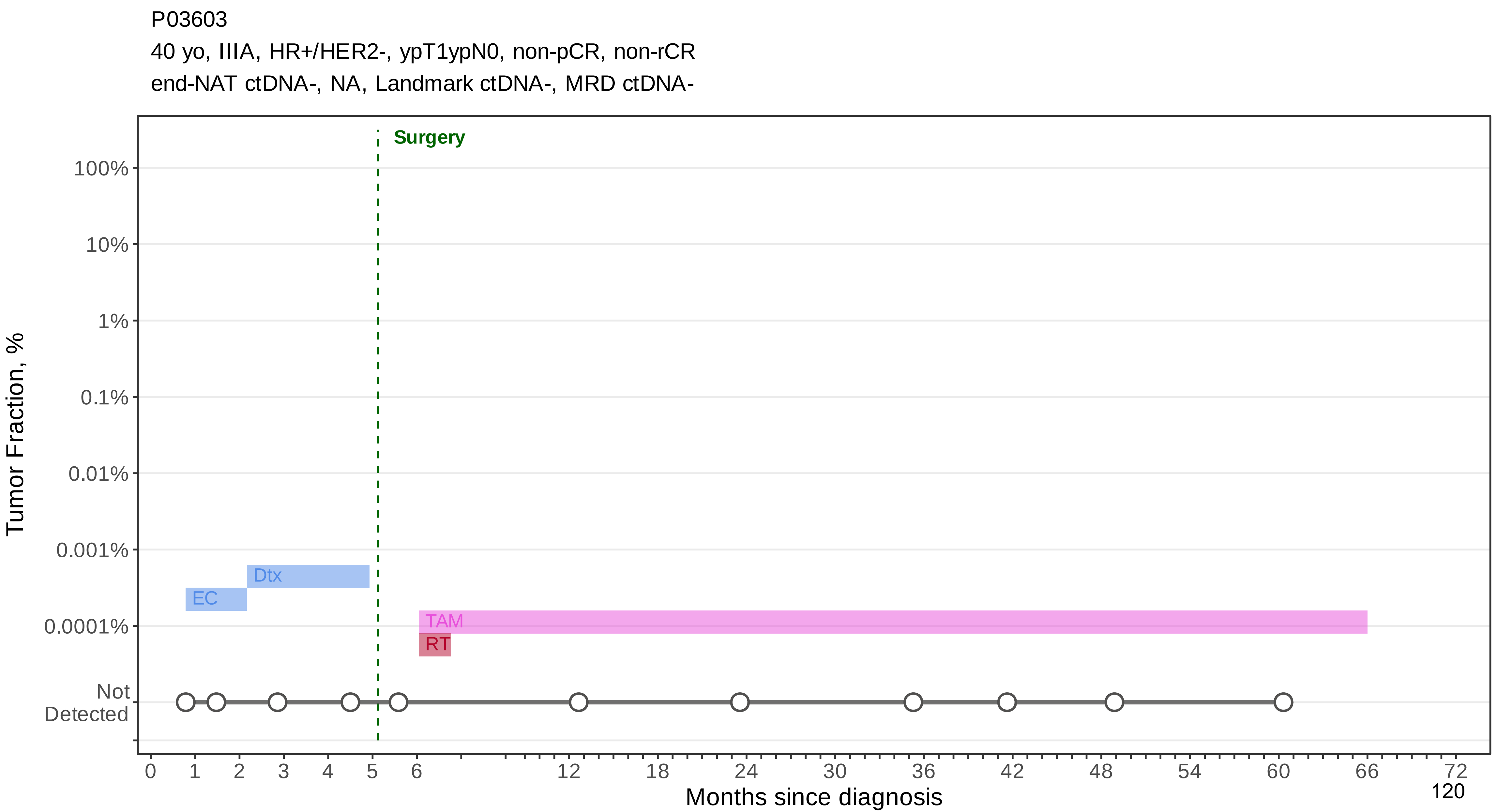
P03603
40 yo, IIIA, HR+/HER2-, ypT1ypN0, non-pCR, non-rCR
end-NAT ctDNA-, NA, Landmark ctDNA-, MRD ctDNA-
Surgery
Tumor Fraction, %
100%
10%
1%
0.1%
0.01%
0.001%
0.0001%
Not Detected
Dtx
EC
TAM
RT
0
1
2
3
4
5
6
12
18
24
30
36
42
48
54
60
66
72
Months since diagnosis

P04603
65 yo, IIA, HER2+, HR-, ypT0ypN1, pCR, NA
end-NAT ctDNA-, NAT ctDNA-responder, Landmark ctDNA-, MRD ctDNA-

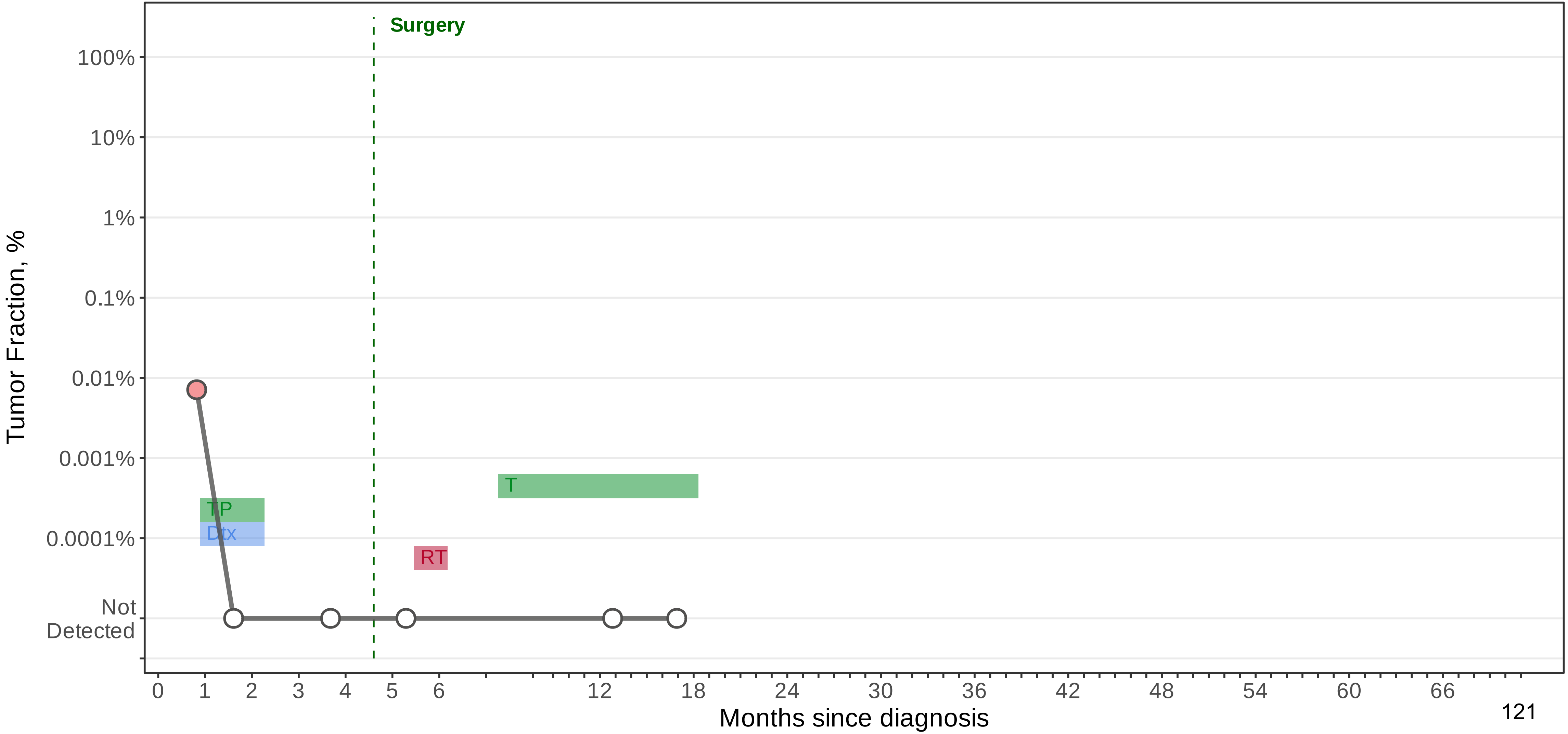

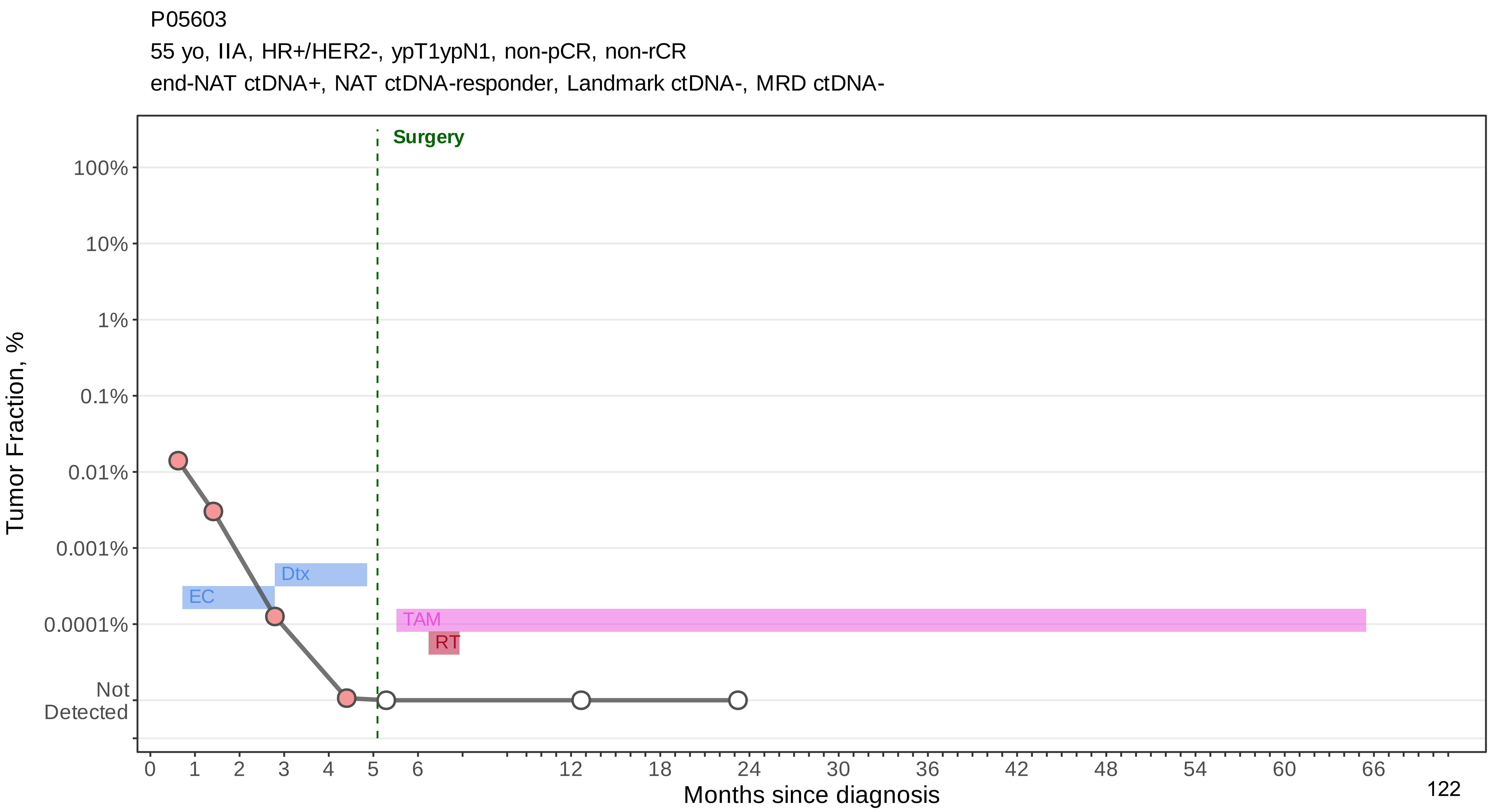
P05603
55 yo, IIA, HR+/HER2-, ypT1ypN1, non-pCR, non-rCR
end-NAT ctDNA+, NAT ctDNA-responder, Landmark ctDNA-, MRD ctDNA-
Surgery
Tumor Fraction, %
100%
10%
1%
0.1%
0.01%
0.001%
0.0001%
Not Detected
EC
Dtx
TAM
RT
0
1
2
3
4
5
6
12
18
24
30
36
42
48
54
60
66
Months since diagnosis

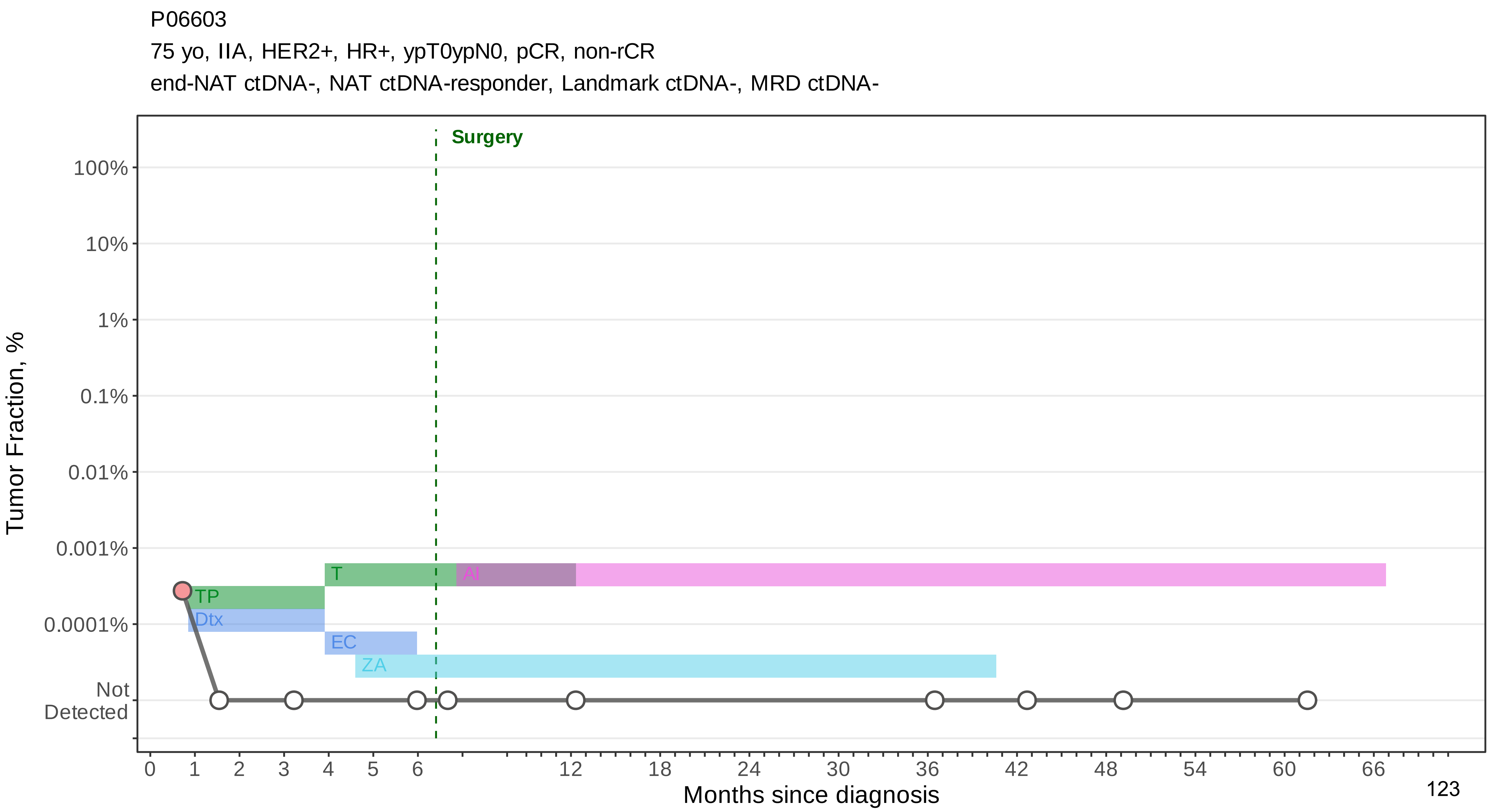
P06603
75 yo, IIA, HER2+, HR+, ypT0ypN0, pCR, non-rCR
end-NAT ctDNA-, NAT ctDNA-responder, Landmark ctDNA-, MRD ctDNA-
Surgery
100%
10%
1%
0.1%
0.01%
0.001%
0.0001%
Not Detected
Tumor Fraction, %
T
AI
TP
Dtx
EC
ZA
0
1
2
3
4
5
6
12
18
24
30
36
42
48
54
60
66
Months since diagnosis

P07603
40 yo, IIA, HR+/HER2-, ypT1ypN0, non-pCR, non-rCR
end-NAT ctDNA-, NAT ctDNA-responder, Landmark ctDNA-, MRD ctDNA-

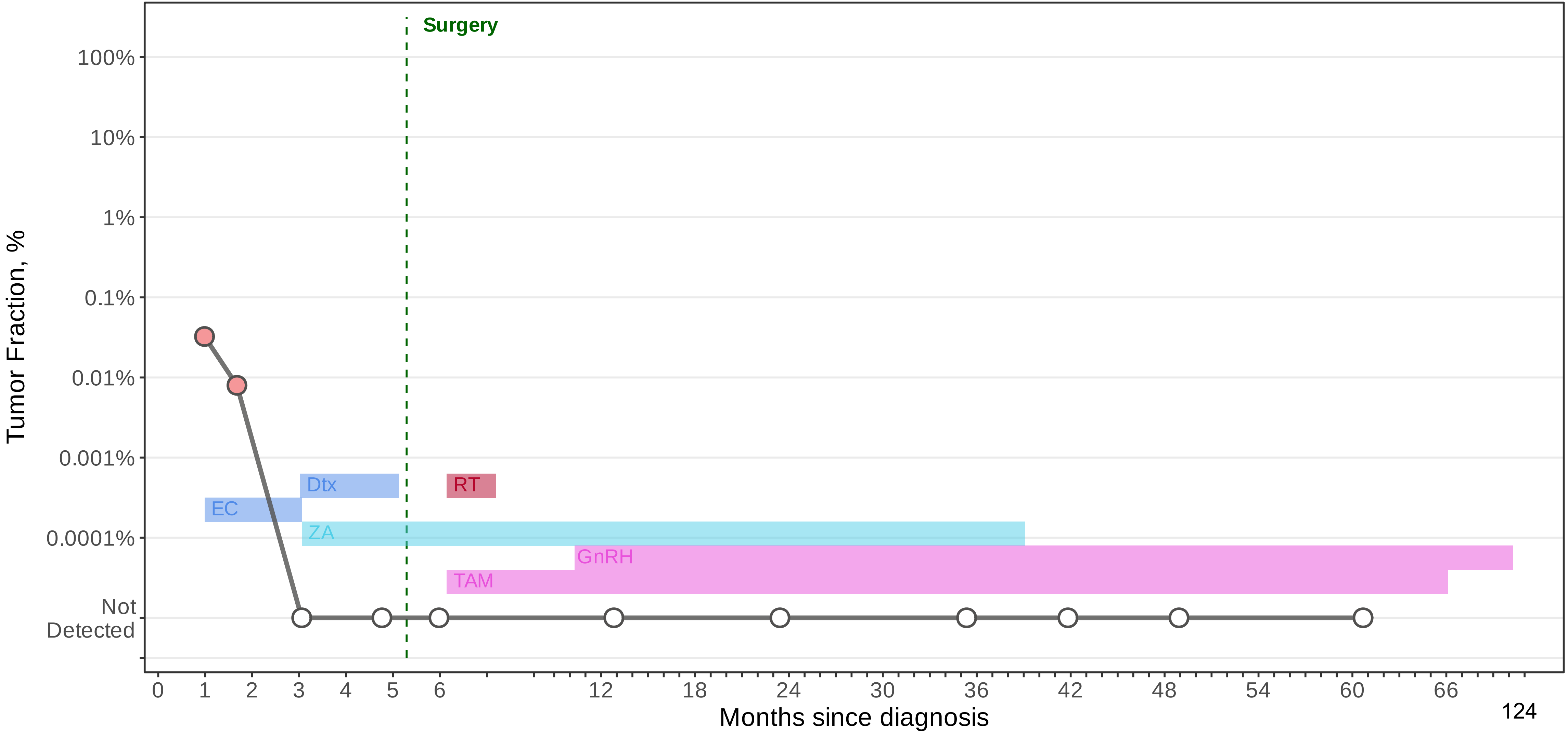

P00703
80 yo, IIA, HER2+, HR+, ypT1ypN0, non-pCR, rCR
end-NAT ctDNA-, NAT ctDNA-responder, Landmark ctDNA-, MRD ctDNA-

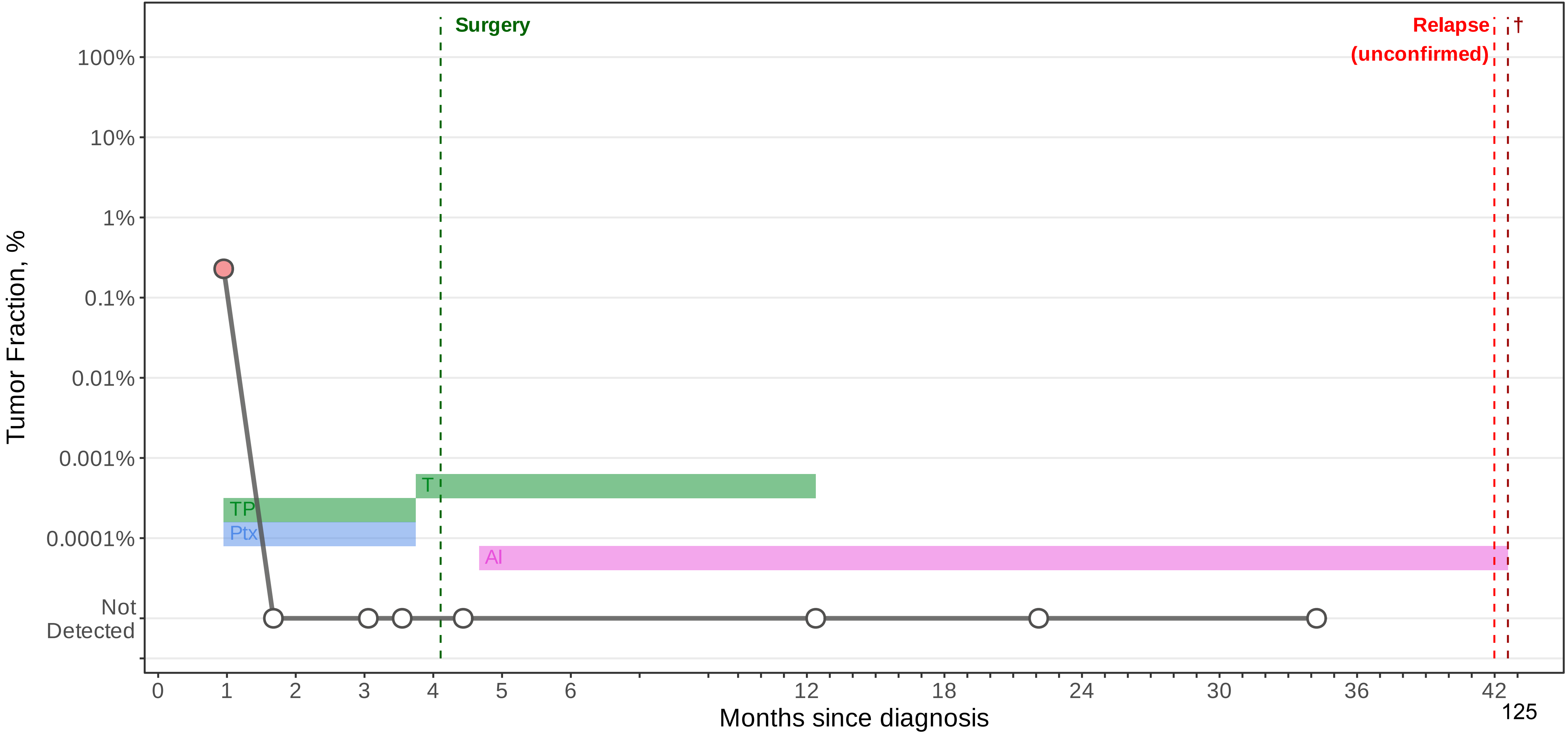

P01703
55 yo, IIIA, HR+/HER2-, ypTXypN3, non-pCR, rCR
end-NAT ctDNA+, NAT ctDNA-non-responder, Landmark ctDNA-, MRD ctDNA-

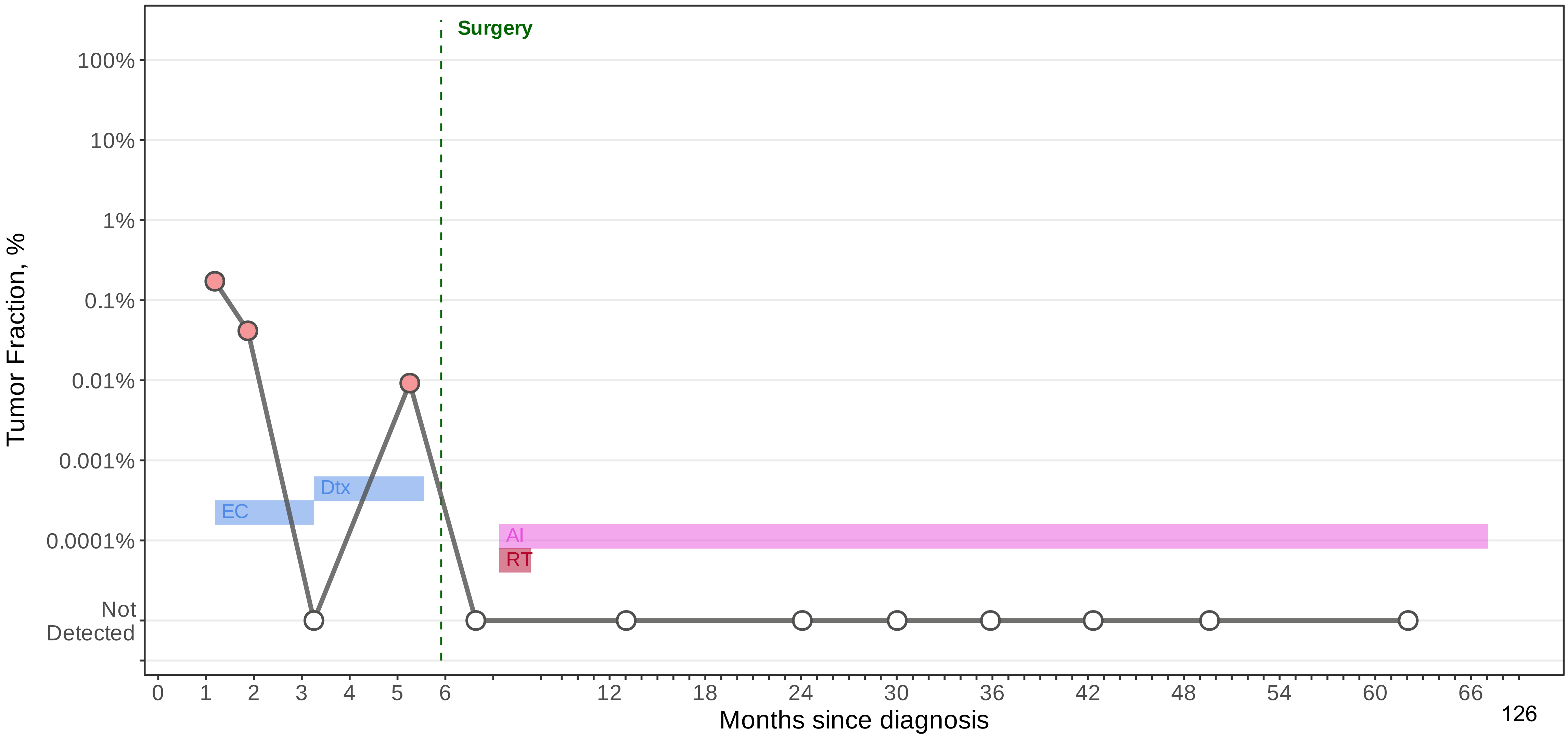

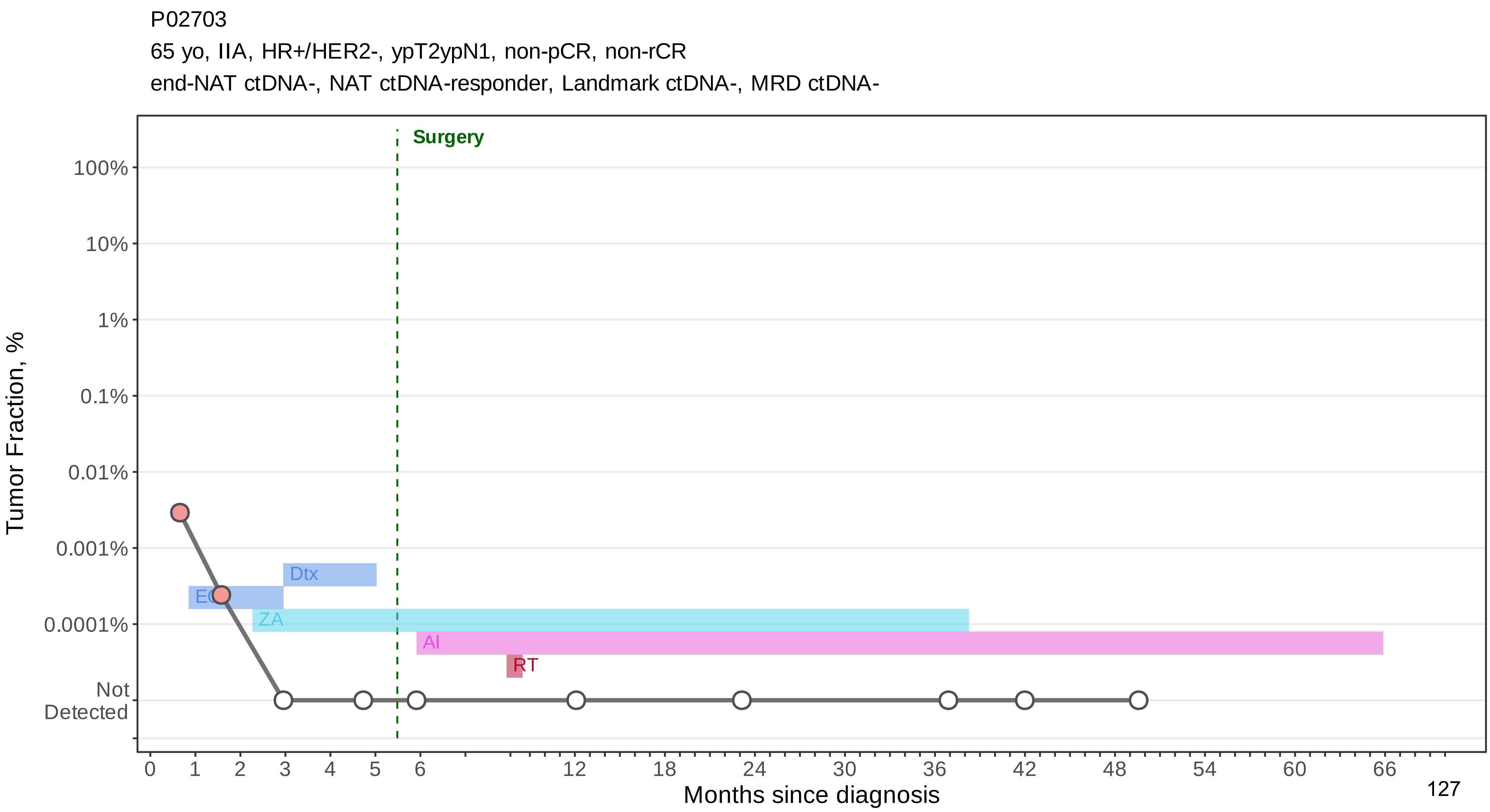

P02703
65 yo, IIA, HR+/HER2-, ypT2ypN1, non-pCR, non-rCR
end-NAT ctDNA-, NAT ctDNA-responder, Landmark ctDNA-, MRD ctDNA-
Surgery
100%
10%
1%
0.1%
0.01%
0.001%
0.0001%
Not Detected
Tumor Fraction, %
Dtx
EC
ZA
AI
RT
0 1 2 3 4 5 6 12 18 24 30 36 42 48 54 60 66
Months since diagnosis

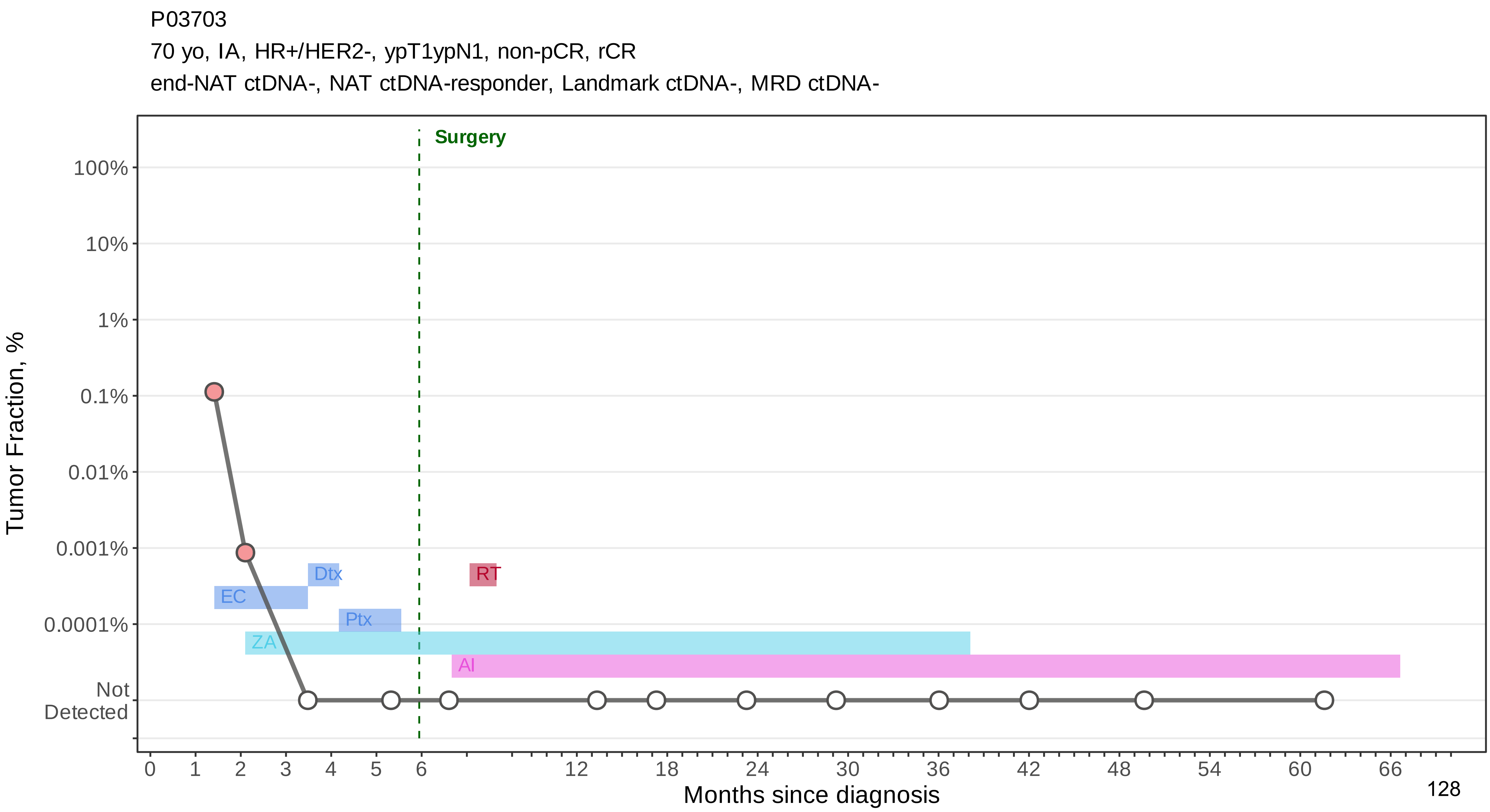
P03703
70 yo, IA, HR+/HER2-, ypT1ypN1, non-pCR, rCR
end-NAT ctDNA-, NAT ctDNA-responder, Landmark ctDNA-, MRD ctDNA-
Surgery
100%
10%
1%
0.1%
0.01%
0.001%
0.0001%
Not Detected
Tumor Fraction, %
Dtx
RT
EC
Ptx
ZA
AI
0
1
2
3
4
5
6
12
18
24
30
36
42
48
54
60
66
Months since diagnosis

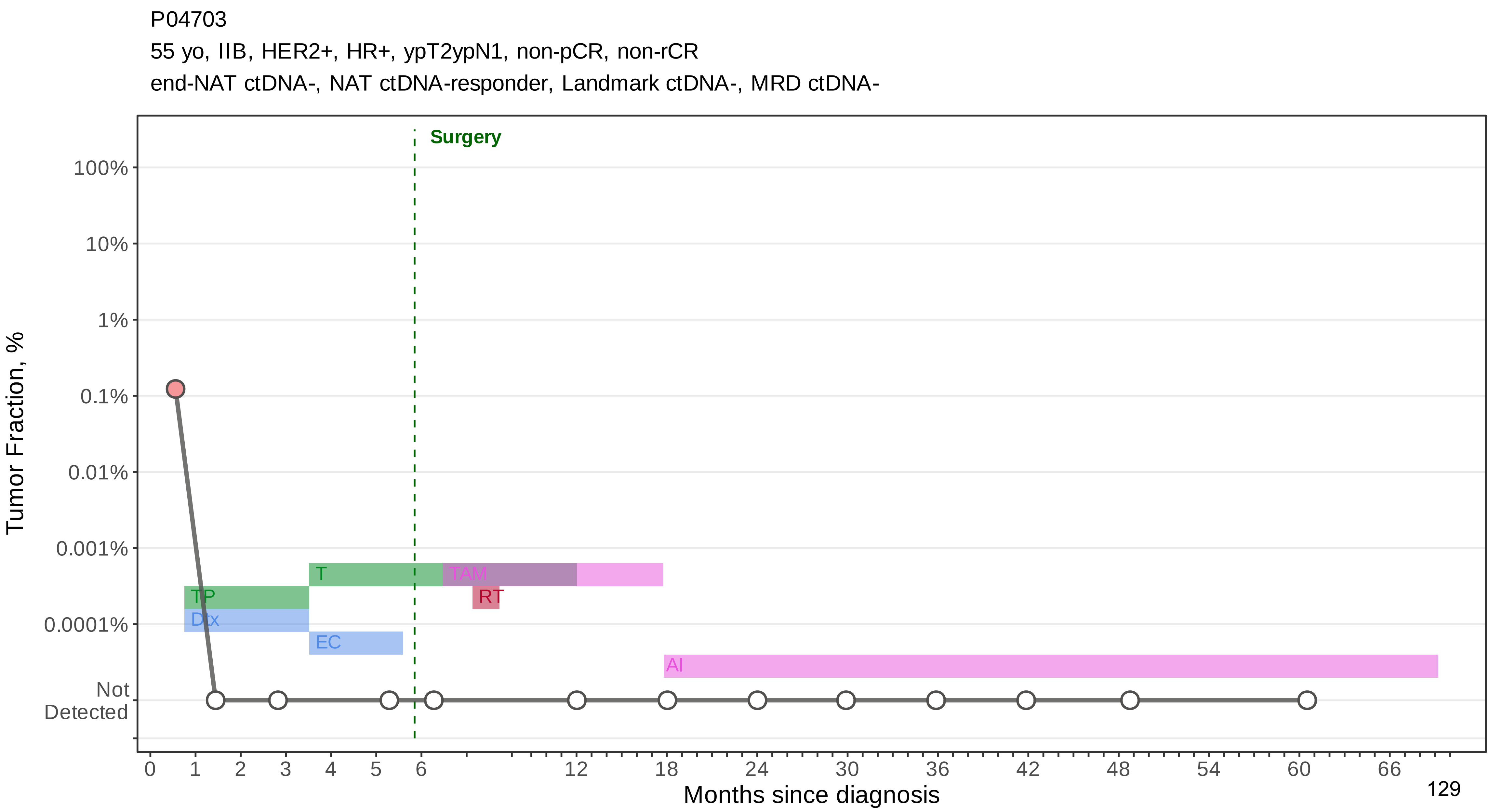

P04703
55 yo, IIB, HER2+, HR+, ypT2ypN1, non-pCR, non-rCR
end-NAT ctDNA-, NAT ctDNA-responder, Landmark ctDNA-, MRD ctDNA-
Surgery
Tumor Fraction, %
100%
10%
1%
0.1%
0.01%
0.001%
0.0001%
Not Detected
T
TAM
TP
RT
Dtx
EC
AI
0 1 2 3 4 5 6 12 18 24 30 36 42 48 54 60 66
Months since diagnosis

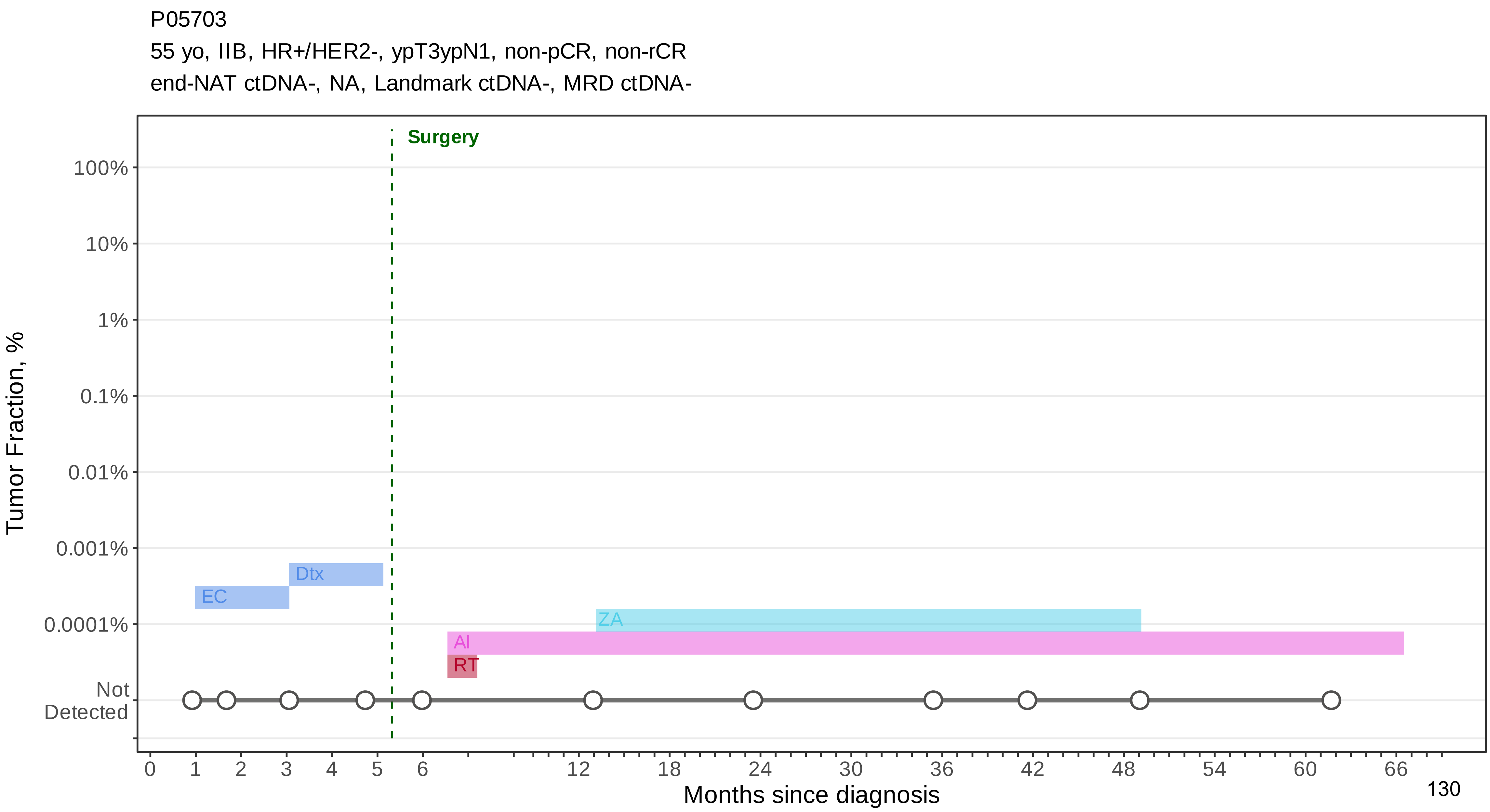
P05703
55 yo, IIB, HR+/HER2-, ypT3ypN1, non-pCR, non-rCR
end-NAT ctDNA-, NA, Landmark ctDNA-, MRD ctDNA-
Surgery
100%
10%
1%
0.1%
0.01%
0.001%
0.0001%
Not Detected
Tumor Fraction, %
Dtx
EC
ZA
AI
RT
0 1 2 3 4 5 6 12 18 24 30 36 42 48 54 60 66
Months since diagnosis

P06703
55 yo, IIA, HR+/HER2-, ypT1ypN1, non-pCR, non-rCR
end-NAT ctDNA-, NAT ctDNA-responder, Landmark ctDNA-, MRD ctDNA-

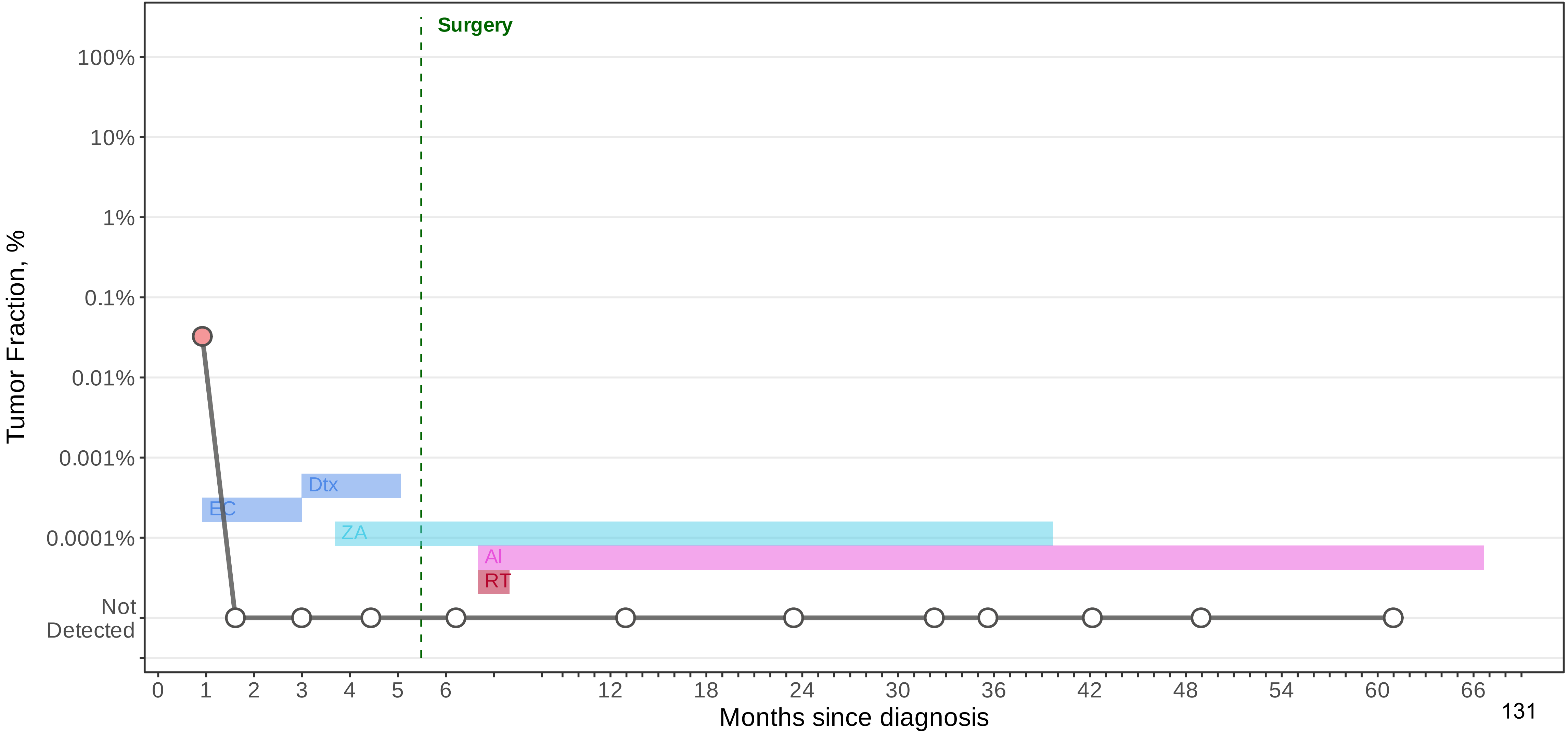

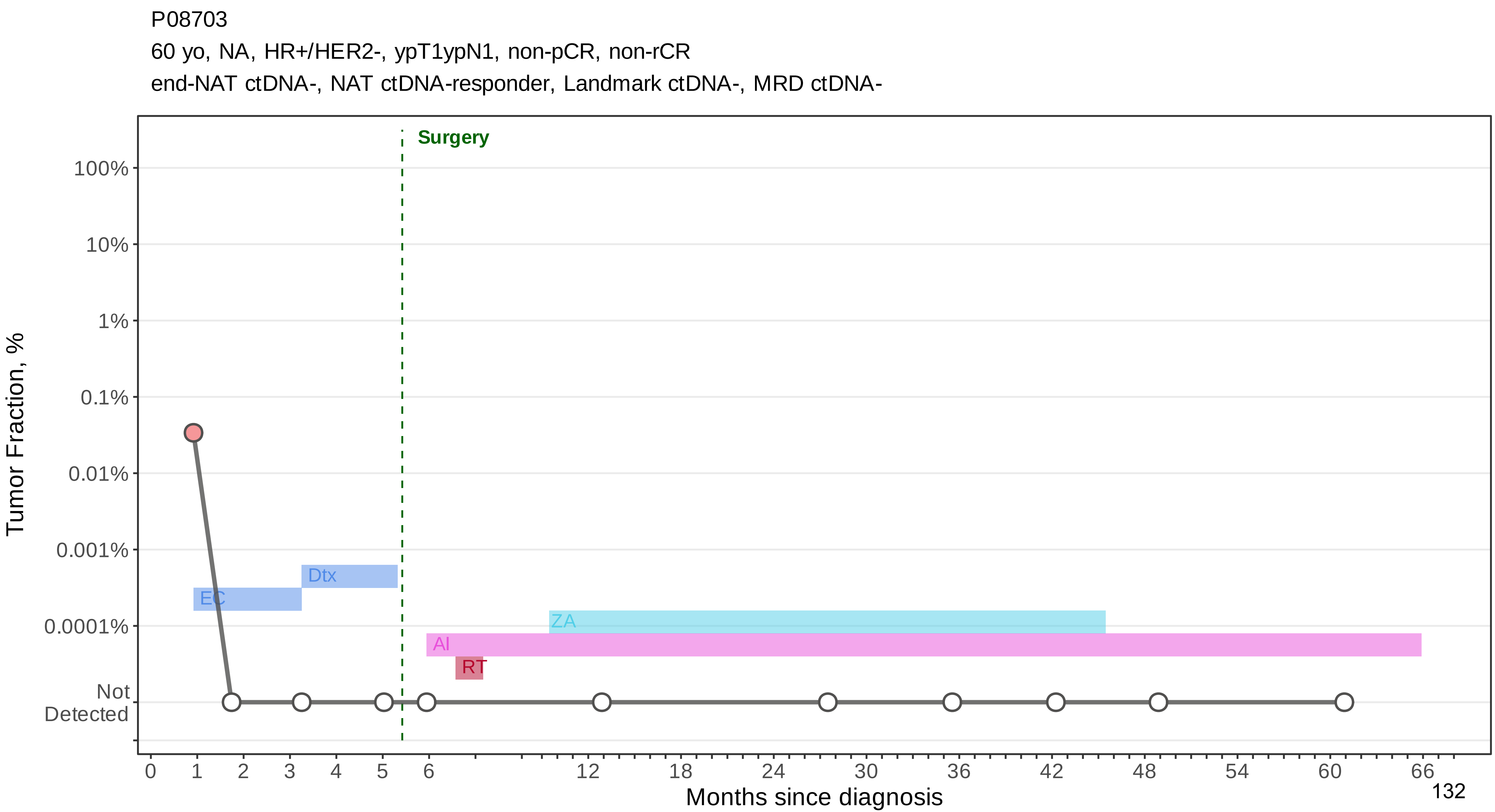

P08703
60 yo, NA, HR+/HER2-, ypT1ypN1, non-pCR, non-rCR
end-NAT ctDNA-, NAT ctDNA-responder, Landmark ctDNA-, MRD ctDNA-
Surgery
100%
10%
1%
0.1%
0.01%
0.001%
0.0001%
Not Detected
Tumor Fraction, %
Dtx
EC
ZA
AI
RT
0
1
2
3
4
5
6
12
18
24
30
36
42
48
54
60
66
Months since diagnosis

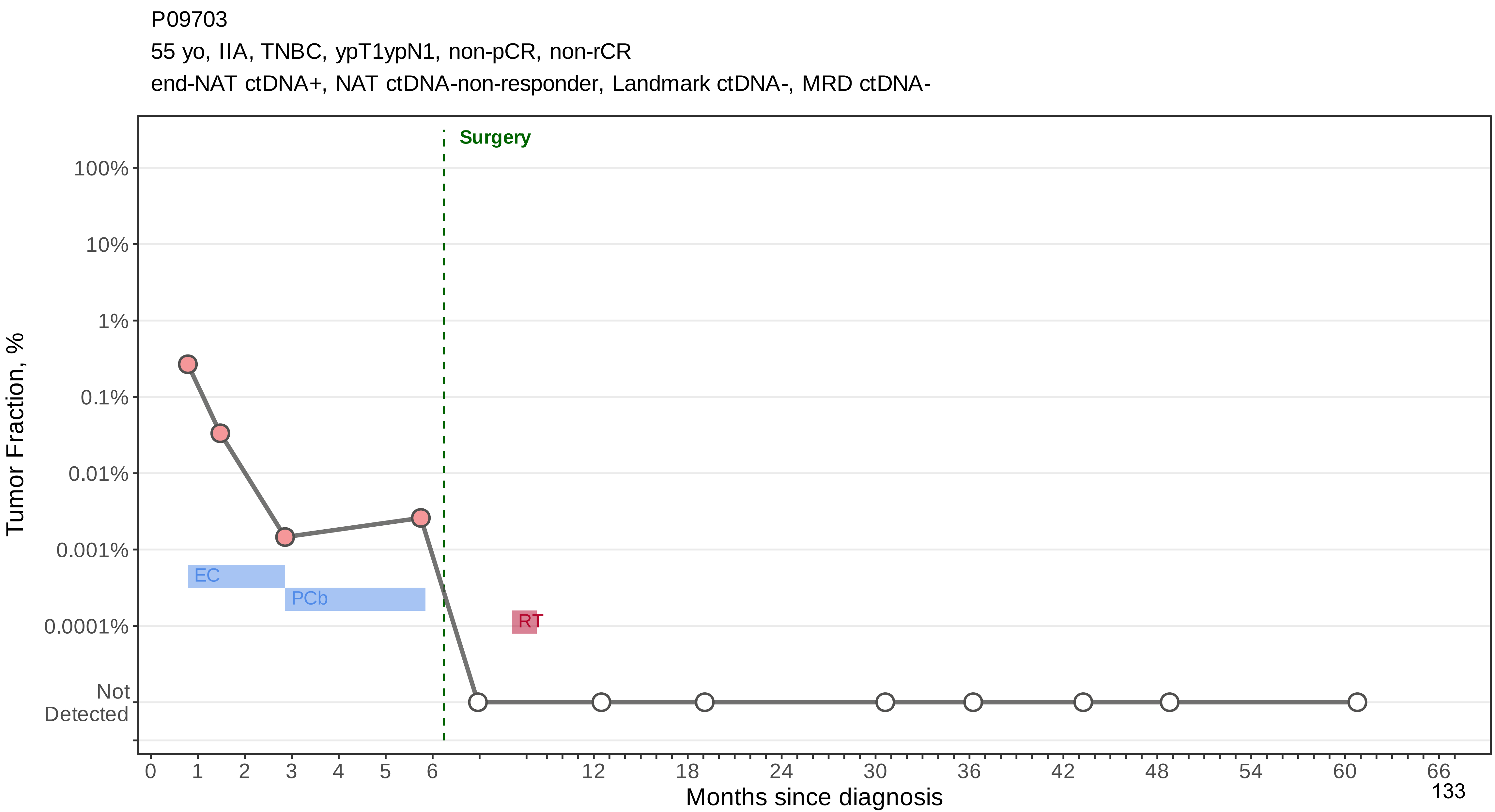
P09703
55 yo, IIA, TNBC, ypT1ypN1, non-pCR, non-rCR
end-NAT ctDNA+, NAT ctDNA-non-responder, Landmark ctDNA-, MRD ctDNA-
Surgery
100%
10%
1%
0.1%
0.01%
0.001%
0.0001%
Not Detected
Tumor Fraction, %
EC
PCb
RT
0 1 2 3 4 5 6 12 18 24 30 36 42 48 54 60 66
Months since diagnosis

P00803
60 yo, IIA, HER2+, HR-, ypT0ypN1, pCR, non-rCR
end-NAT ctDNA-, NAT ctDNA-responder, Landmark ctDNA-, MRD ctDNA-

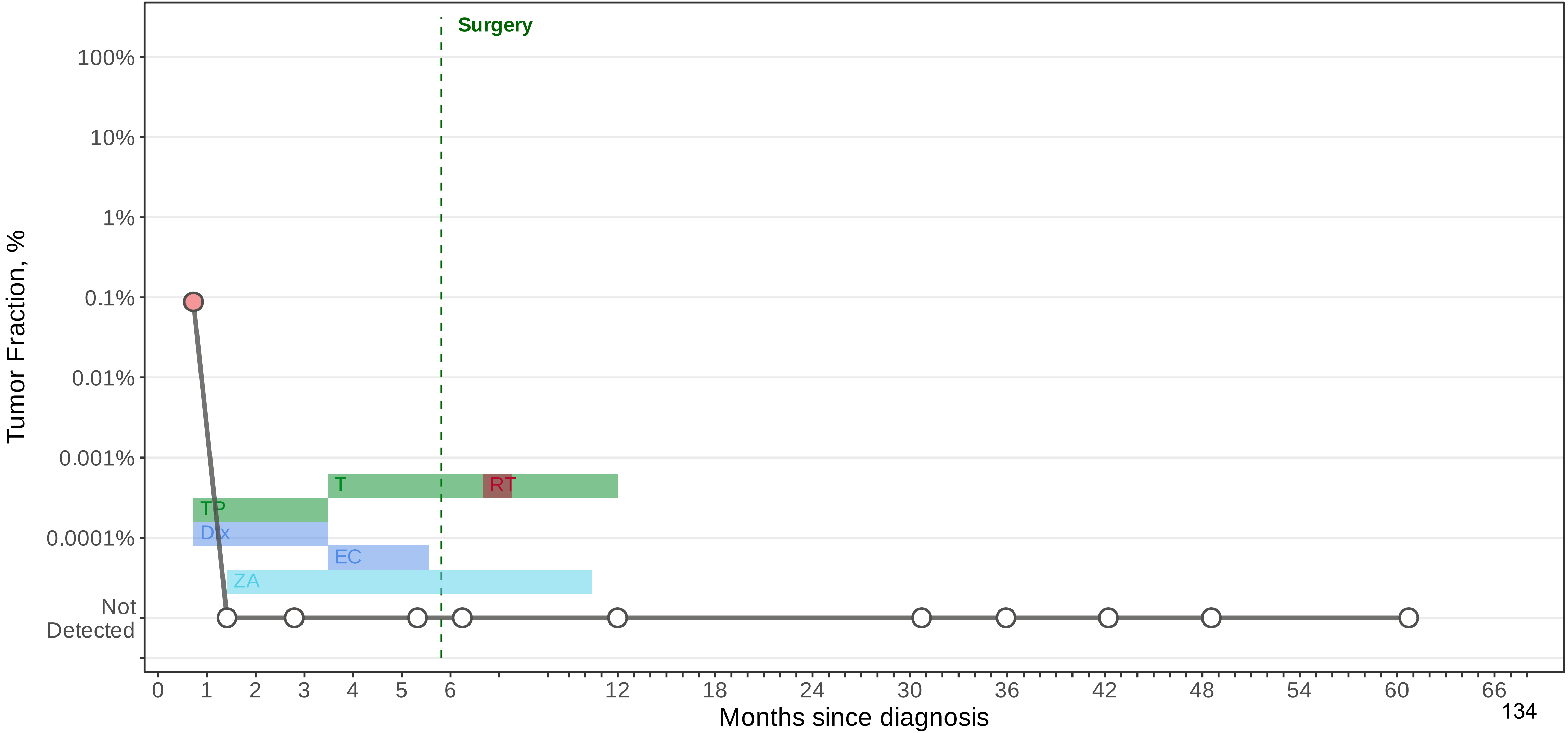

P03803
55 yo, IA, HER2+, HR+, ypT0ypN0, pCR, rCR
end-NAT ctDNA-, NA, Landmark ctDNA-, MRD ctDNA-

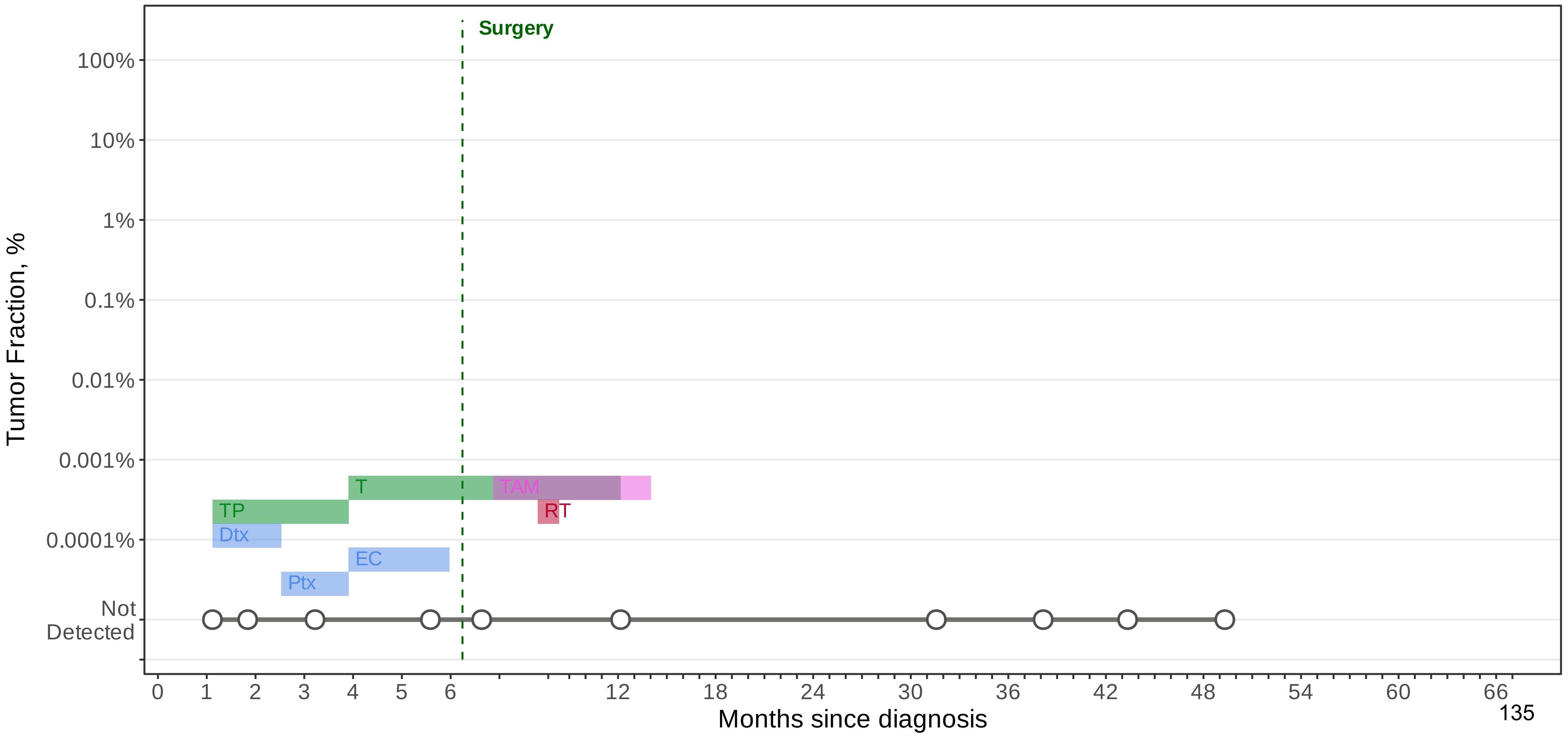

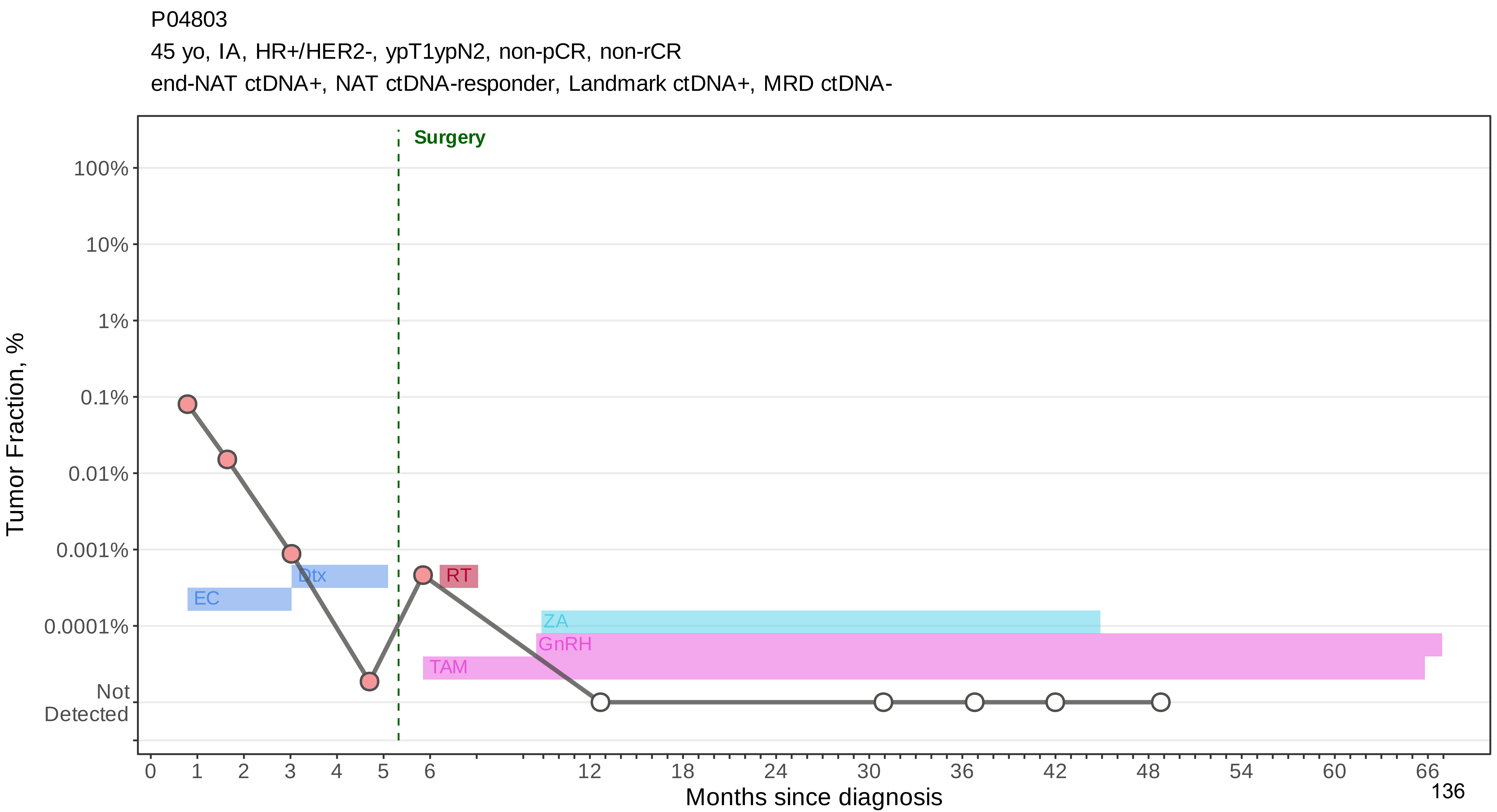
P04803
45 yo, IA, HR+/HER2-, ypT1ypN2, non-pCR, non-rCR
end-NAT ctDNA+, NAT ctDNA-responder, Landmark ctDNA+, MRD ctDNA-
Surgery
100%
10%
1%
0.1%
0.01%
0.001%
0.0001%
Not Detected
Tumor Fraction, %
Dtx
EC
RT
ZA
GnRH
TAM
0 1 2 3 4 5 6 12 18 24 30 36 42 48 54 60 66
Months since diagnosis

P05803
60 yo, IIA, TNBC, ypT1ypN0, non-pCR, non-rCR
end-NAT ctDNA-, NAT ctDNA-responder, Landmark ctDNA-, MRD ctDNA-

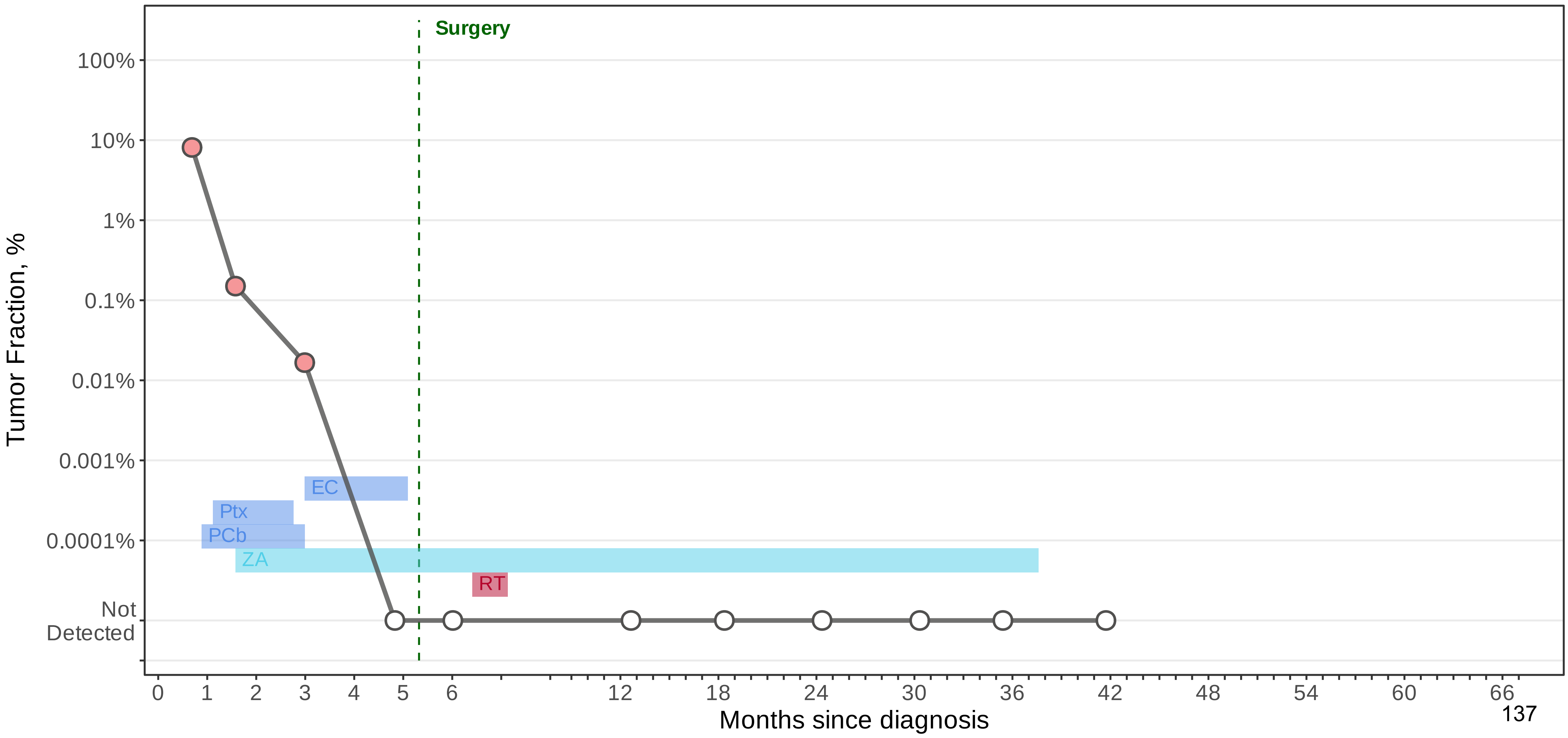

P06803
55 yo, IIB, HER2+, HR-, ypT0ypN0, pCR, rCR
end-NAT ctDNA+, NAT ctDNA-non-responder, Landmark ctDNA-, MRD ctDNA-

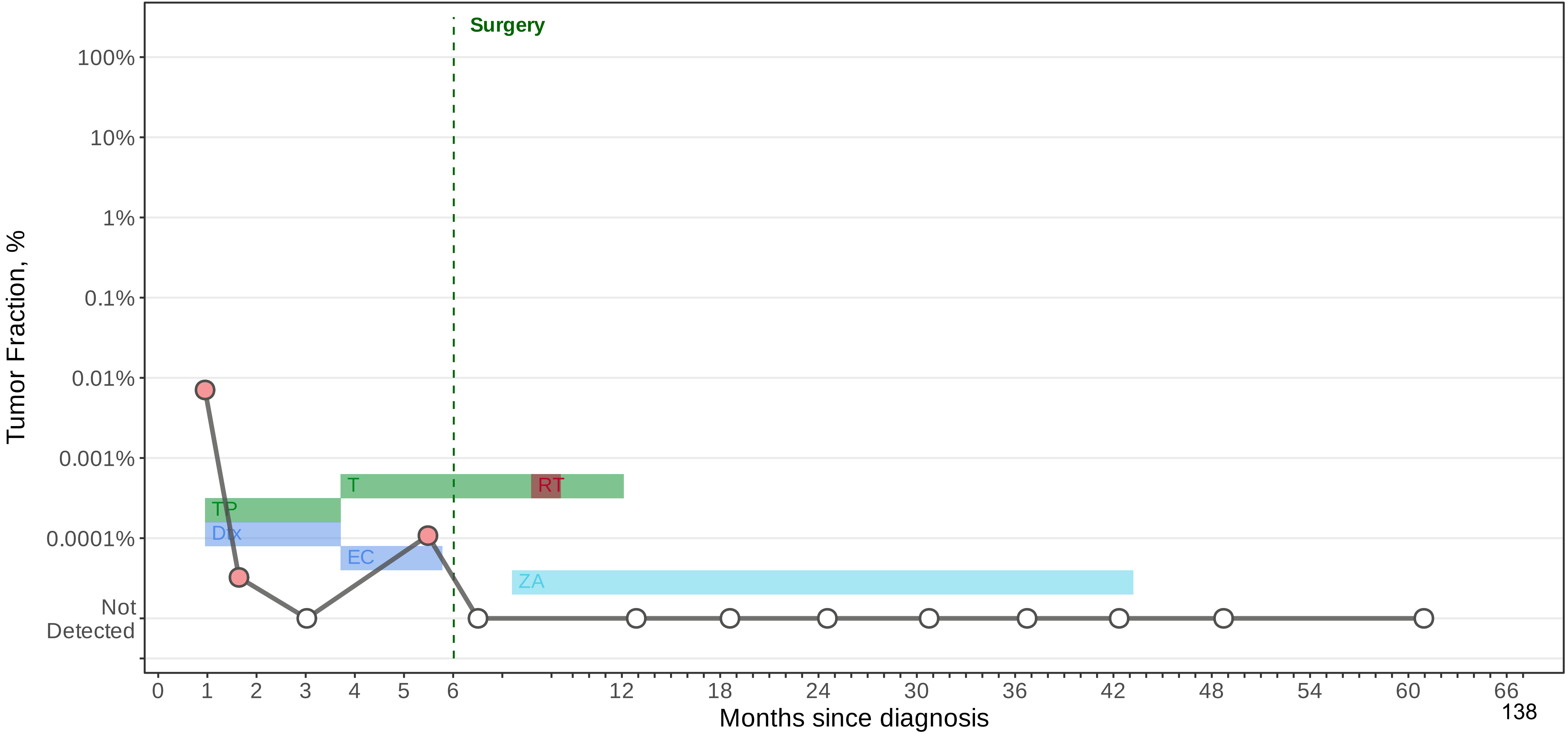

Appendix Figure S2: Kaplan-Meier survival estimates for the ER+/HER2– subgroup.

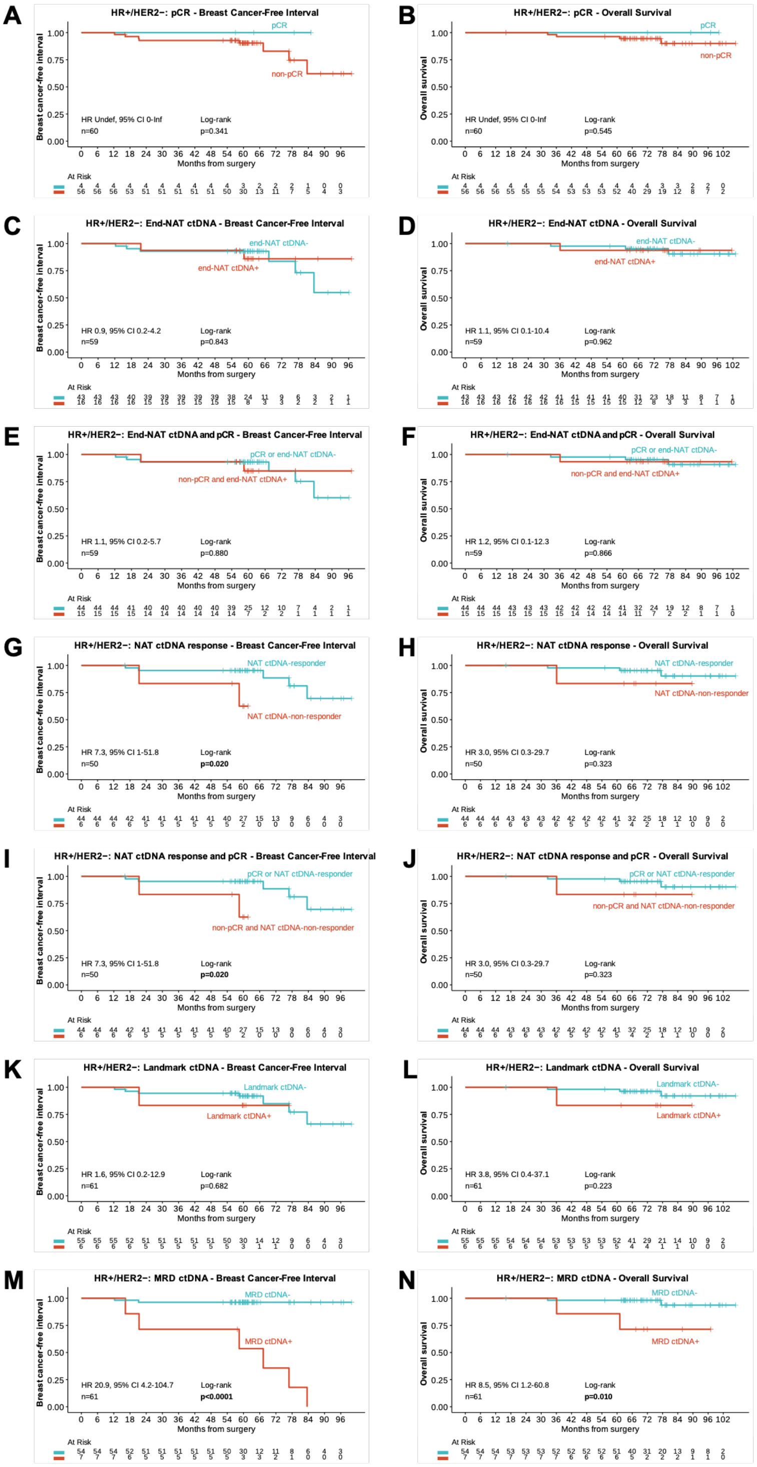

For breast cancer-free interval (**A**, **C**, **E**, **G**, **I**, **K**, **M**) and overall survival (**B**, **D**, **F**, **H**, **J**, **L**, **N**), survival curves are plotted for: (**A**-**B**) pCR, (**C**-**D**) End-NAT ctDNA, (**E**-**F**) End-NAT ctDNA and pCR combined, (**G**-**J**) NAT ctDNA response, (**I**-**J**) NAT ctDNA response and pCR combined, **(K-L)** Landmark ctDNA timepoint, (**M**-**N**) Follow-up MRD. P-values calculated using the log-rank test.

Appendix Figure S3: Kaplan-Meier survival estimates for the HER2+ subgroup.

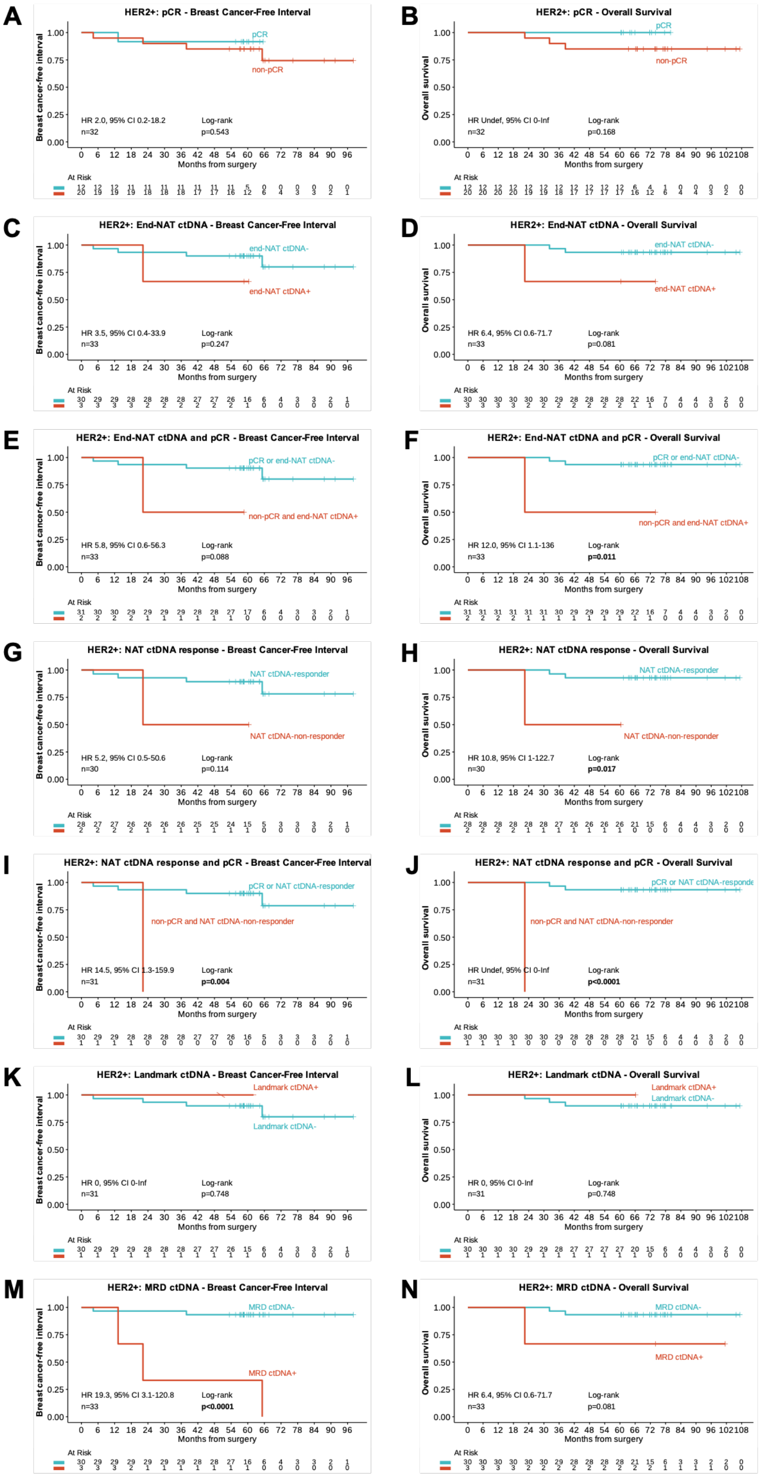

For breast cancer-free interval (**A**, **C**, **E**, **G**, **I**, **K**, **M**) and overall survival (**B**, **D**, **F**, **H**, **J**, **L**, **N**), survival curves are plotted for: (**A**-**B**) pCR, (**C**-**D**) End-NAT ctDNA, (**E**-**F**) End-NAT ctDNA and pCR combined, (**G**-**J**) NAT ctDNA response, (**I**-**J**) NAT ctDNA response and pCR combined, **(K-L)** Landmark ctDNA timepoint, (**M**-**N**) Follow-up MRD. P-values calculated using the log-rank test.

Appendix Figure S4: Kaplan-Meier survival estimates for the TNBC subgroup.

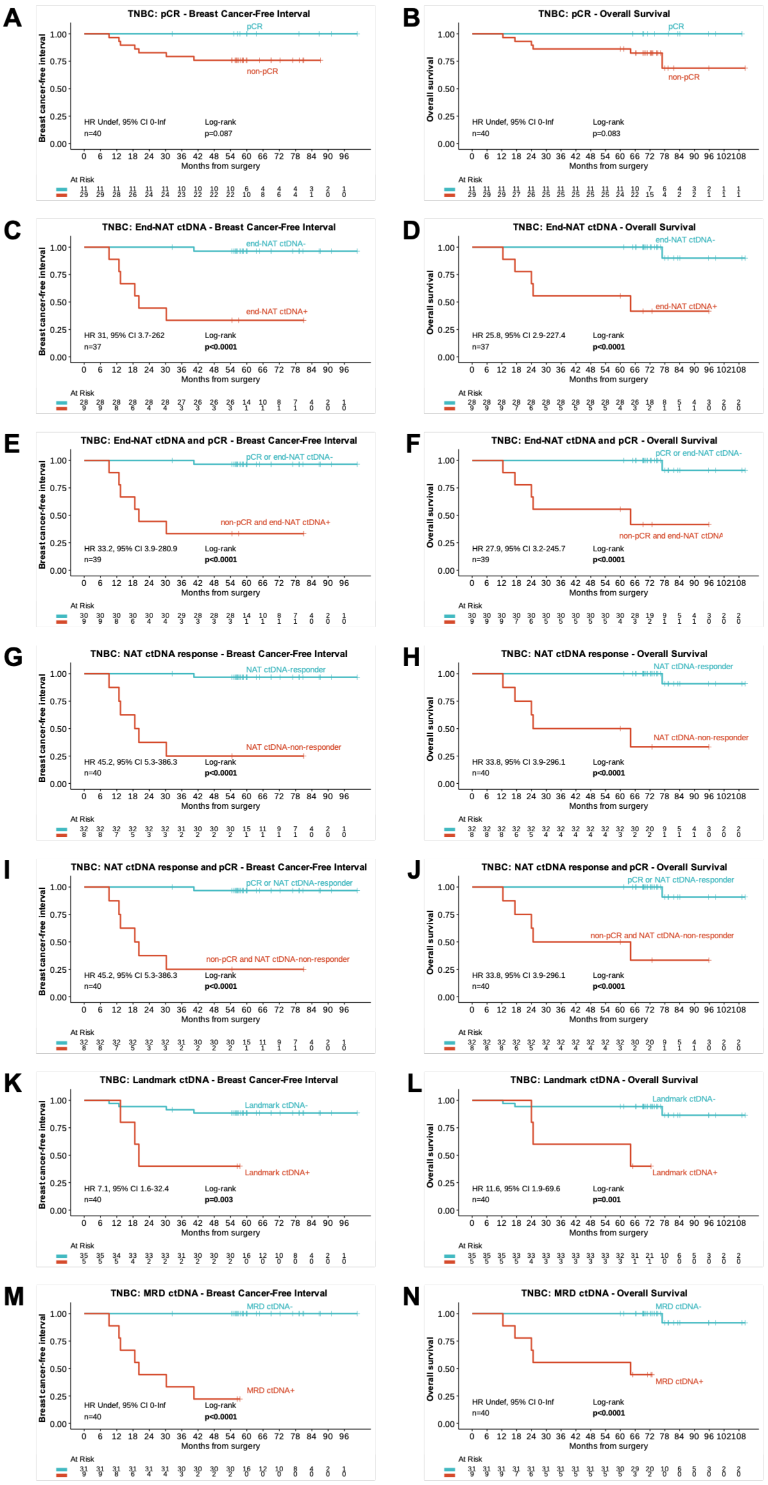

For breast cancer-free interval (**A**, **C**, **E**, **G**, **I**, **K**, **M**) and overall survival (**B**, **D**, **F**, **H**, **J**, **L**, **N**), survival curves are plotted for: (**A**-**B**) pCR, (**C**-**D**) End-NAT ctDNA, (**E**-**F**) End-NAT ctDNA and pCR combined, (**G**-**J**) NAT ctDNA response, (**I**-**J**) NAT ctDNA response and pCR combined, **(K**-**L)** Landmark ctDNA timepoint, (**M**-**N**) Follow-up MRD. P-values calculated using the log-rank test.

Appendix Table S1: Per-patient clinicopathological characteristics.

Patient ID	Age (5-year bins)	Clinical stage	Clinical subtype	Nottingham histological grade	Tumor size (mm)	Lymph node status	Tumor tissue	Neoadjuvant regimen	Adjuvant regimen	Follow-up time (months)	Type of BC-specific recurrence	Site(s) of metastasis	Death event
P00072	55	IIA	HR+/HER2-	3	10	Negative	FFPE	Chemo	Endocrine, RT	79.6	No		No
P00161	55	IIA	HR+/HER2-	NA	NA	n/a	FFPE	Chemo	Endocrine, RT	112	No		No
P00182	50	IIB	HER2+	NA	NA	Negative	FFPE	Anti-HER2, Chemo	Anti-HER2, RT	77.7	No		No
P00261	40	IIA	HR+/HER2-	NA	10	Negative	FFPE	Chemo	Endocrine	22.1	No		No
P00291	55	IIB	HR+/HER2-	NA	0	Negative	FF	Chemo	Endocrine, RT	102.5	No		No
P00392	65	IIB	HER2+	2	11	1 to 3	FFPE	Anti-HER2, Chemo, Supportive	Anti-HER2, Supportive, Endocrine, RT	75.2	No		No
P00492	40	IIA	TNBC	2	19	Negative	FFPE	Chemo	Chemo, RT	74.4	No		No
P00542	60	IIA	HR+/HER2-	2	6	1 to 3	FF	Chemo	Endocrine, RT	87.8	No		No
P00603	50	IIB	TNBC	2	35	1 to 3	FFPE	Chemo	RT	71.3	No		No
P00652	65	IIB	HER2+	NA	3	1 to 3	FFPE	Anti-HER2, Chemo	Anti-HER2, Endocrine, RT	38.9	CNS-only relapse	CNS	Yes
P00703	80	IIA	HER2+	NA	4	Negative	FFPE	Anti-HER2, Chemo	Anti-HER2, Endocrine	42.5	Distant relapse	lung,other	Yes
P00752	45	IIA	TNBC	NA	NA	Negative	FFPE	Chemo	None	85.1	No		No
P00803	60	IIA	HER2+	NA	NA	1 to 3	FFPE	Anti-HER2, Chemo, Supportive	Anti-HER2, Supportive, RT	67	No		No
P00862	50	IIA	HR+/HER2-	2	16	Negative	FF	Chemo	Endocrine, RT	82.8	No		No
P01002	45	IIB	HR+/HER2-	NA	22	1 to 3	FF	Chemo	Endocrine, RT	102.6	Distant relapse	skeleton	No
P01061	35	IIIA	HER2+	NA	23	≥ 4	FF	Anti-HER2, Chemo	Anti-HER2, Endocrine, RT	27.5	Distant relapse	lung,liver	Yes
P01082	70	IIB	HER2+	2	6	1 to 3	FF	Anti-HER2, Chemo	Anti-HER2, Supportive, RT	79.4	No		No
P01091	55	IIIA	HR+/HER2-	NA	10	Negative	FFPE	Chemo	Endocrine, RT	106.7	No		No
P01182	35	IIA	HR+/HER2-	NA	NA	Negative	FFPE	Chemo	Endocrine, RT	77.4	No		No
P01292	65	IIB	HR+/HER2-	2	25	1 to 3	FFPE	Chemo, Supportive	Supportive, Endocrine, RT	76.7	No		No
P01392	50	IIIA	HR+/HER2-	1	10	1 to 3	FFPE	Chemo	Supportive, Endocrine, RT	75.2	No		No
P01503	55	IIB	HR+/HER2-	2	31	≥ 4	FFPE	Chemo, Supportive	Supportive, Endocrine, RT	72	No		No
P01603	55	IA	HER2+	NA	NA	Negative	FFPE	Anti-HER2, Chemo, Supportive	Anti-HER2, Supportive, RT	70.5	No		No
P01652	40	IIA	HER2+	1	22	Negative	FF	Anti-HER2, Chemo	Anti-HER2, Endocrine, RT	86.8	No		No
P01703	55	IIIA	HR+/HER2-	NA	NA	≥ 4	FFPE	Chemo	Endocrine, RT	68.5	No		No
P01752	45	IIB	TNBC	2	20	Negative	FF	Chemo	RT	84	No		No
P01862	35	IIIA	HER2+	1	8	1 to 3	FF	Anti-HER2, Chemo	Anti-HER2, Supportive, Endocrine, RT	82.6	No		No
P01962	75	IIA	HR+/HER2-	2	1	≥ 4	FF	Chemo, Supportive	Supportive, Endocrine, RT	81.7	No		No
P02002	50	IIIC	HR+/HER2-	NA	5	≥ 4	FFPE	Chemo	Endocrine, RT	101.8	No		No
P02061	40	IIA	TNBC	NA	7	1 to 3	FF	Chemo	RT	115.9	No		No
P02091	70	IIA	HR+/HER2-	NA	20	1 to 3	FF	Chemo	Supportive, Endocrine, RT	106.5	No		No
P02182	45	IIB	TNBC	NA	NA	1 to 3	FFPE	Chemo	RT	77.4	No		No
P02191	50	IIA	TNBC	NA	NA	Negative	FFPE	Chemo	None	104.4	No	other	No
P02292	35	IIB	TNBC	3	50	≥ 4	FFPE	Chemo	Chemo, RT	70.4	Local-only relapse	local	Yes
P02322	25	IIA	HR+/HER2-	NA	20	1 to 3	FF	Chemo	Chemo, Supportive, Endocrine	92.6	Local-only relapse	local	No
P02392	60	IIB	HR+/HER2-	2	12	1 to 3	FFPE	Chemo, Supportive	Supportive, Endocrine, RT	75.3	No		No
P02403	75	NA	TNBC	NA	NA	Negative	FFPE	Chemo	None	74.1	No		No
P02503	35	IIIA	HER2+	2	12	1 to 3	FFPE	Chemo	Anti-HER2, Chemo, Endocrine, RT	72.2	No		No
P02542	65	IIA	HR+/HER2-	3	27	Negative	FF	Chemo	Endocrine	60.7	No		No
P02652	45	IIA	TNBC	3	27	1 to 3	FFPE	Chemo	RT	23.5	Distant relapse	liver,skeleton,other	Yes
P02703	65	IIA	HR+/HER2-	2	22	1 to 3	FFPE	Chemo, Supportive	Supportive, Endocrine, RT	69.4	No	other	No
P02752	65	IIA	HR+/HER2-	2	3	Negative	FF	Chemo	Endocrine, RT	83.9	No		No
P02962	35	NA	HER2+	3	10	Negative	FFPE	Anti-HER2, Chemo	Anti-HER2, RT	81.1	No		No
P03002	50	IIB	TNBC	NA	7	1 to 3	FF	Chemo	RT	101.8	No		No
P03082	55	IIIA	HER2+	2	1	Negative	FF	Anti-HER2, Chemo, Supportive	Anti-HER2, Supportive, Endocrine, RT	79.6	No		No
P03091	70	IIB	HR+/HER2-	NA	10	1 to 3	FF	Chemo	Supportive, Endocrine, RT	106.4	No		No
P03161	35	IIA	HER2+	NA	7	Negative	FFPE	Anti-HER2, Chemo	Anti-HER2, Endocrine, RT	112	No		No
P03182	50	IIA	TNBC	NA	NA	Negative	FFPE	Chemo	RT	77.3	No		No
P03292	70	IIA	TNBC	NA	NA	Negative	FFPE	Chemo	None	76.2	No		No
P03392	60	IIB	HR+/HER2-	2	11	1 to 3	FFPE	Chemo, Supportive	Supportive, Endocrine, RT	75.1	No		No
P03503	50	IIA	HER2+	2	9	Negative	FFPE	Anti-HER2, Chemo	Anti-HER2, Endocrine, RT	72.7	No		No
P03603	40	IIIA	HR+/HER2-	2	7	Negative	FFPE	Chemo	Endocrine, RT	71	No		No
P03703	70	IA	HR+/HER2-	1	15	1 to 3	FFPE	Chemo, Supportive	Supportive, Endocrine, RT	69	No		No
P03752	75	IIA	HER2+	2	10	Negative	FF	Anti-HER2, Chemo	Anti-HER2, Endocrine	83.9	No		No
P03803	55	IA	HER2+	NA	NA	Negative	FFPE	Anti-HER2, Chemo	Anti-HER2, Endocrine, RT	66.8	No		No
P03862	45	IIA	HR+/HER2-	3	25	Negative	FF	Chemo	Chemo, Supportive, Endocrine, RT	82.6	No		No
P03962	40	IIB	HR+/HER2-	2	NA	Negative	FFPE	Chemo	Endocrine, RT	81	No		No
P04002	45	IIA	TNBC	NA	NA	n/a	FFPE	Chemo	None	101.4	No		No
P04082	50	IIA	TNBC	NA	3	Negative	FFPE	Anti-HER2, Chemo	Anti-HER2	79.8	Distant relapse	lymphatic	No
P04091	70	IIB	HR+/HER2-	NA	44	1 to 3	FF	Chemo	Supportive, Endocrine, RT	41.4	Distant relapse	CNS,lymphatic,liver,skeleton	Yes
P04182	50	IIB	HER2+	NA	NA	Negative	FFPE	Anti-HER2, Chemo	Anti-HER2, Endocrine, RT	77.3	No		No
P04222	70	IIB	HR+/HER2-	NA	5	1 to 3	FFPE	Chemo, Supportive	Supportive, Endocrine, RT	94.8	No		No
P04292	60	IIA	TNBC	2	14	Negative	FFPE	Chemo	RT	76.2	Local-only relapse	local	No
P04322	30	IIA	TNBC	NA	6	1 to 3	FF	Chemo	Chemo, RT	29.9	Distant relapse	liver	Yes
P04392	55	IIA	TNBC	NA	NA	Negative	FFPE	Chemo	RT	75.2	No		No
P04503	50	IIA	NA	3	NA	Negative	FFPE	Chemo	Supportive	71.5	Local-only relapse	local	No
P04603	65	IIA	HER2+	3	NA	1 to 3	FFPE	Anti-HER2, Chemo	Anti-HER2, RT	70.4	No		No
P04703	55	IIB	HER2+	2	21	1 to 3	FFPE	Anti-HER2, Chemo	Anti-HER2, Endocrine, RT	69.1	No		No
P04752	70	IIA	HER2+	2	10	Negative	FF	Anti-HER2, Chemo	Anti-HER2, Endocrine	83.9	No		No
P04803	45	IA	HR+/HER2-	2	12	≥ 4	FFPE	Chemo	Supportive, Endocrine, RT	66.8	No		No
P04862	55	IIA	HR+/HER2-	3	10	≥ 4	FFPE	Chemo	Supportive, Endocrine, RT	82.1	No	other	No
P05082	65	IIA	TNBC	NA	NA	Negative	FFPE	Chemo	RT	79.2	No		No
P05182	60	IIB	HR+/HER2-	2	14	Negative	FFPE	Chemo, Supportive	Supportive, Endocrine, RT	77	No		No
P05222	75	IIA	HR+/HER2-	NA	17	1 to 3	FFPE	Chemo, Supportive	Supportive, Endocrine, RT	83.2	No	other	Yes
P05292	55	NA	TNBC	3	9	Negative	FFPE	Chemo	Supportive, Endocrine, RT	75.7	No		No
P05392	50	IIB	HR+/HER2-	1	12	1 to 3	FFPE	Chemo	Endocrine, RT	75	No		No
P05403	55	IIA	HER2+	2	8	Negative	FFPE	Anti-HER2, Chemo, Supportive	Anti-HER2, Supportive, Endocrine, RT	73	No		No
P05503	65	IIB	HR+/HER2-	2	27	1 to 3	FFPE	Chemo, Supportive	Supportive, Endocrine, RT	72.1	No	other	No
P05603	55	IIA	HR+/HER2-	2	17	1 to 3	FFPE	Chemo	Endocrine, RT	70.2	No		No
P05652	55	IIB	HER2+	NA	0	1 to 3	FF	Anti-HER2, Chemo	Anti-HER2, RT	85.8	No		No
P05703	55	IIB	HR+/HER2-	2	70	1 to 3	FFPE	Chemo	Supportive, Endocrine, RT	68.6	No		No
P05752	65	IIA	HER2+	3	0	Negative	FF	Anti-HER2, Chemo, Supportive	Anti-HER2, Supportive, Endocrine, RT	84	No		No
P05803	60	IIA	TNBC	2	6	Negative	FFPE	Chemo, Supportive	Supportive, RT	66.5	No		No
P05862	45	IA	HR+/HER2-	3	11	Negative	FF	Chemo	Chemo, RT	82.1	No		No
P05962	55	IIA	TNBC	3	NA	Negative	FFPE	Chemo	Anti-HER2, Chemo	80.3	No		No
P06061	65	IIB	NA	NA	NA	Negative	FFPE	Chemo	RT	65.2	No		No
P06091	75	IIIA	HER2+	NA	50	1 to 3	FF	Anti-HER2, Chemo	Anti-HER2, Supportive, Endocrine, RT	107.2	Distant relapse	other	No
P06182	40	IIB	TNBC	2	3	Negative	FFPE	Chemo	Supportive, RT	77.1	No		No
P06292	60	IIA	HR+/HER2-	1	20	1 to 3	FFPE	Chemo, Supportive	Supportive, Endocrine, RT	75.9	No		No
P06392	50	IIB	TNBC	NA	NA	Negative	FFPE	Chemo	RT	74.6	No		No
P06403	75	IIB	HR+/HER2-	2	22	≥ 4	FFPE	Chemo, Endocrine	Supportive, Endocrine, RT	72.8	Distant relapse	skeleton	No
P06442	55	IIA	TNBC	NA	0	Negative	FF	Chemo	RT	89.6	No		No
P06503	55	IIIA	HR+/HER2-	2	25	1 to 3	FFPE	Chemo	Supportive, Endocrine, RT	67.6	No		No
P06552	60	IIB	TNBC	3	5	1 to 3	FF	Chemo	RT	17.5	Distant relapse	liver,skeleton	Yes
P06603	75	IIA	HER2+	NA	NA	Negative	FFPE	Anti-HER2, Chemo, Supportive	Anti-HER2, Supportive, Endocrine	70.2	No		No
P06703	55	IIA	HR+/HER2-	1	16	1 to 3	FFPE	Chemo, Supportive	Supportive, Endocrine, RT	68.4	No		No
P06803	55	IIB	HER2+	3	NA	Negative	FFPE	Anti-HER2, Chemo	Anti-HER2, Supportive, RT	66.3	No		No
P06962	45	IIB	HER2+	NA	NA	Negative	FFPE	Anti-HER2, Chemo	Anti-HER2, RT	80	CNS-only relapse	CNS	No
P07061	45	IIIA	TNBC	NA	NA	1 to 3	FFPE	Chemo	RT	114.9	No		No
P07082	60	IIB	HER2+	NA	NA	Negative	FFPE	Anti-HER2, Chemo, Supportive	Anti-HER2, Supportive, Endocrine, RT	78.9	No		No
P07091	60	IIA	TNBC	NA	4	Negative	FF	Chemo	None	82	No		Yes
P07222	65	IIB	HR+/HER2-	NA	2	≥ 4	FF	Chemo, Supportive	Supportive, Endocrine, RT	94.5	No		No
P07292	50	IIA	HR+/HER2-	3	NA	1 to 3	FFPE	Chemo	Endocrine, RT	75.7	Distant relapse	lymphatic,skeleton	No
P07392	60	IIB	TNBC	2	2	Negative	FFPE	Chemo, Supportive	Supportive, RT	74.8	No		No
P07403	75	IIB	TNBC	NA	10	Negative	FFPE	Chemo, Supportive	Supportive, RT	71.8	No		No
P07552	50	IIB	HR+/HER2-	2	16	≥ 4	FF	Chemo	Endocrine, RT	87.6	No		No
P07603	40	IIA	HR+/HER2-	2	4	Negative	FFPE	Chemo, Supportive	Supportive, Endocrine, RT	70.2	No		No
P07652	55	IIA	HR+/HER2-	NA	2	1 to 3	FFPE	Chemo	Supportive, Endocrine, RT	84.9	No		No
P07762	65	IIA	HER2+	2	12	1 to 3	FFPE	Anti-HER2, Chemo, Supportive	Anti-HER2, Supportive, Endocrine, RT	83.5	No		No
P07862	60	IIIA	HR+/HER2-	2	38	≥ 4	FFPE	Chemo, Supportive	Supportive, Endocrine, RT	80.8	No		No
P07962	45	IIA	TNBC	NA	13	Negative	FF	Chemo	RT	79.8	No		No
P08091	65	IIB	HR+/HER2-	NA	0	Negative	FF	Chemo	Supportive, Endocrine, RT	105.7	No		No
P08102	40	IIA	HER2+	NA	35	≥ 4	FF	Anti-HER2, Chemo	Anti-HER2, Supportive, Endocrine, RT	100	No		No
P08222	55	NA	HR+/HER2-	NA	NA	Negative	FF	Chemo	Supportive, Endocrine, RT	95.2	No		No
P08322	35	IIA	HR+/HER2-	NA	23	Negative	FFPE	Chemo	Endocrine	90.9	Distant relapse	contralateral,liver,skeleton	No
P08392	30	IIB	HR+/HER2-	2	40	1 to 3	FFPE	Chemo	Supportive, Endocrine, RT	74.3	No		No
P08403	65	IIIA	HR+/HER2-	2	NA	≥ 4	FFPE	Chemo, Supportive	Supportive, Endocrine, RT	66.3	Distant relapse	liver,skeleton	Yes
P08442	35	IIA	TNBC	NA	NA	Negative	FFPE	Chemo	RT	89	No		No
P08503	75	IIB	HR+/HER2-	2	40	≥ 4	FFPE	Chemo	Endocrine, RT	38.4	Distant relapse	skeleton	Yes
P08552	60	NA	HR+/HER2-	1	9	1 to 3	FF	Chemo, Supportive	Supportive, Endocrine, RT	87.6	No		No
P08652	50	IIA	HR+/HER2-	NA	23	Negative	FF	Chemo	Endocrine, RT	84.4	No	other	No
P08703	60	NA	HR+/HER2-	2	17	1 to 3	FFPE	Chemo	Supportive, Endocrine, RT	67.1	No		No
P08862	60	IIA	HER2+	1	20	1 to 3	FFPE	Anti-HER2, Chemo	Anti-HER2, Supportive, Endocrine	81.7	No		No
P08962	40	IIB	TNBC	2	8	Negative	FF	Chemo	RT	79.6	No		No
P09061	35	IIB	HER2+	NA	15	1 to 3	FF	Anti-HER2, Chemo	Anti-HER2, Endocrine, RT	112.7	No		No
P09082	50	IIB	TNBC	2	NA	1 to 3	FFPE	Chemo	RT	78.3	No		No
P09102	65	IIB	HR+/HER2-	NA	10	≥ 4	FFPE	Chemo, Supportive	Supportive, Endocrine, RT	102	No		No
P09161	35	IA	HR+/HER2-	NA	13	1 to 3	FFPE	Chemo	Endocrine, RT	108.8	No		No
P09322	65	IIA	TNBC	NA	8	≥ 4	FF	Chemo, Supportive	Supportive, RT	29	Distant relapse	CNS,contralateral,lymphatic,liver,skeleton	Yes
P09503	60	IIB	TNBC	NA	NA	Negative	FFPE	Chemo, Supportive	Supportive, RT	71.1	No		No
P09552	80	IIA	TNBC	3	10	Negative	FF	Chemo	RT	87.3	No		No
P09652	50	IIB	HR+/HER2-	2	15	1 to 3	FF	Chemo	Chemo, Endocrine, RT	84.6	No		No
P09703	55	IIA	TNBC	2	14	1 to 3	FFPE	Chemo	RT	66.1	No		No
P09762	50	IIA	TNBC	2	11	n/a	FF	Chemo	RT	82.8	No		No
P09862	45	IIB	TNBC	NA	7	Negative	FF	Chemo	RT	81	No		No
P09962	45	IIA	HR+/HER2-	2	8	1 to 3	FF	Chemo	Endocrine, RT	78.2	No		No

Appendix Table S2: Exact P-values.

Figure and Panel	P-value	Statistical test
Fig. 4 B	0.000600	Log-rank
Fig. 4 D	0.00000000761	Log-rank
Fig. 4 E	0.0000000279	Log-rank
Fig. 6 A	0.0000000000000000000001	Log-rank
Fig. 6 B	0.0000000151	Log-rank
Fig. EV3 E	0.0000817	Fisher's Exact Test (two sided)
Fig. EV4 E	0.000000000744	Log-rank
Fig. EV4 F	0.00000000598	Log-rank
Appendix Fig. S2 M	0.000000213	Log-rank
Appendix Fig. S3 J	0.0000000432	Log-rank
Appendix Fig. S3 M	0.0000136	Log-rank
Appendix Fig. S4 C	0.00000155	Log-rank
Appendix Fig. S4 D	0.0000211	Log-rank
Appendix Fig. S4 E	0.00000063	Log-rank
Appendix Fig. S4 F	0.00000995	Log-rank
Appendix Fig. S4 G	0.000000012	Log-rank
Appendix Fig. S4 H	0.000000872	Log-rank
Appendix Fig. S4 I	0.000000012	Log-rank
Appendix Fig. S4 J	0.000000872	Log-rank
Appendix Fig. S4 M	0.00000000102	Log-rank
Appendix Fig. S4 N	0.00000247	Log-rank
