# Supplementary material for: NeoCircle: pre- and post-operative circulating tumor DNA dynamics predicts survival in neoadjuvant-treated early breast cancer
Source: EMBO Mol Med. 2026 May 26;18(7):2617–34. doi: 10.1038/s44321-026-00447-z (PMC13365804; doi:10.1038/s44321-026-00447-z)
Supplement: Supplementary file 4 — Expanded View Figures [file 44321_2026_447_MOESM4_ESM.pdf]

Expanded View Figures

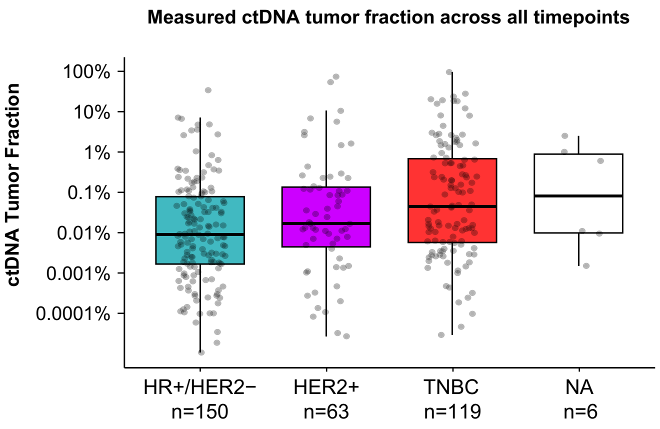

Figure EV1. Measured ctDNA tumor fraction across all timepoints.

Boxplots of every ctDNA tumor fraction measured across all 1497 plasma samples for all patients, according to the tumor clinical subtype.

© The Author(s)

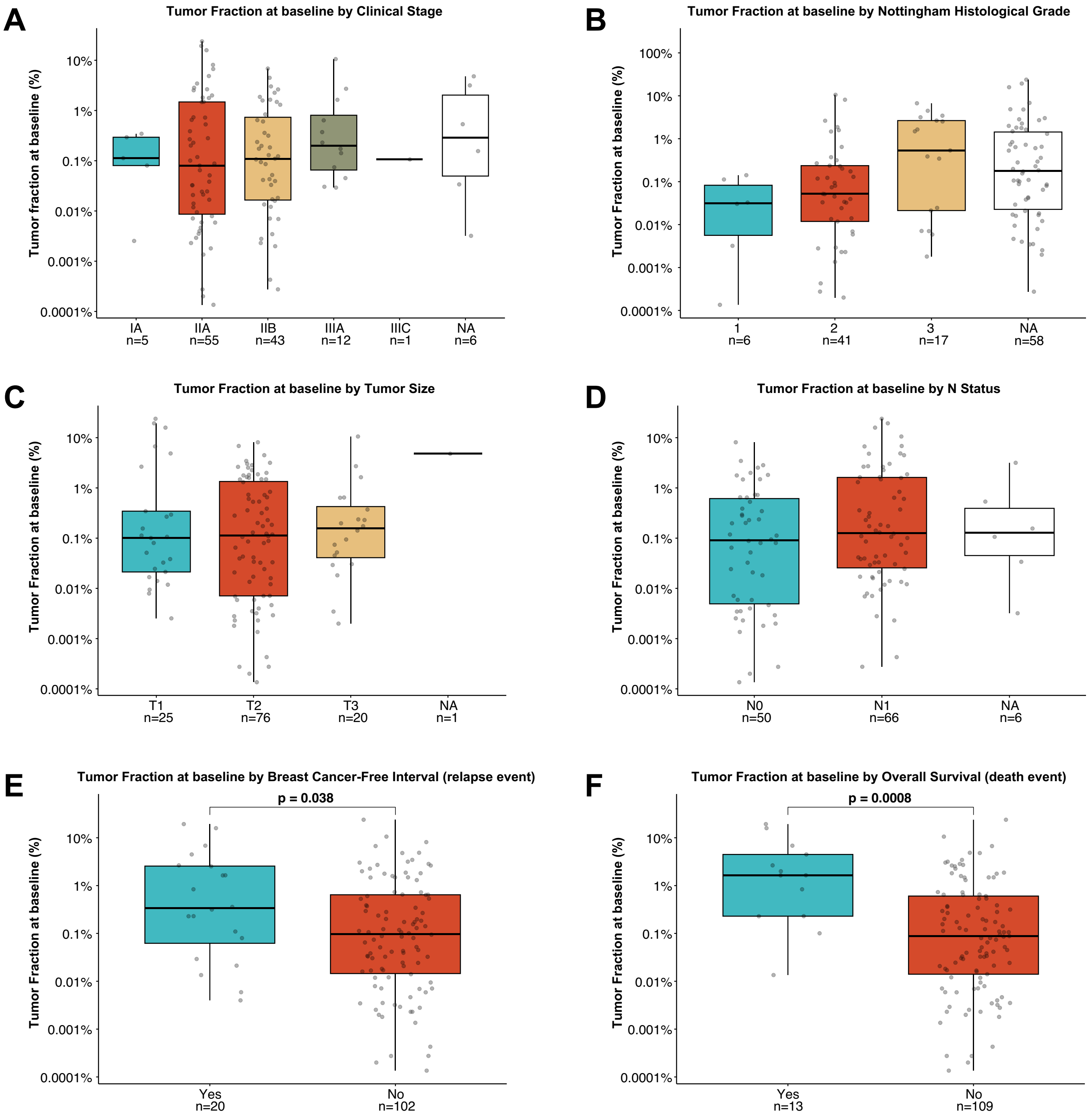

Figure EV2. Measured ctDNA tumor fraction at baseline timepoint by clinicopathological categories.

(**A**) Tumor fraction boxplots at baseline by clinical stage. (**B**) Tumor fraction boxplots at baseline by Nottingham Histological Grade. (**C**) Tumor fraction boxplots at baseline by clinical Tumor size. (**D**) Tumor fraction boxplots at baseline by clinical Node stage. (**E**) Tumor fraction boxplots at baseline by breast cancer-free interval. (**F**) Tumor fraction boxplots at baseline by overall survival. *P*-values calculated using the Mann-Whitney U test.

© The Author(s)

A

p=0.013	Recurrence +	Recurrence -	Event rate
end-NAT ctDNA+	9	19	32.1%
end-NAT ctDNA−	12	91	11.5%

B

p=0.007	Death +	Death -	Event rate
end-NAT ctDNA+	7	21	25.0%
end-NAT ctDNA−	6	97	5.8%

C

p=0.250	Recurrence +	Recurrence -	Event rate
non-pCR	19	86	18.1%
pCR	2	27	6.9%

D

p=0.070	Death +	Death -	Event rate
non-pCR	13	92	12.4%
pCR	0	29	0%

E

p<0.0001	Recurrence +	Recurrence -	Event rate
NAT ctDNA-non-responder	9	7	56.3%
NAT ctDNA-responder	11	95	10.4%

F

p=0.0002	Death +	Death -	Event rate
NAT ctDNA-non-responder	7	9	43.8%
NAT ctDNA-responder	6	100	5.7%

G

p=0.081	Recurrence +	Recurrence -	Event rate
Landmark ctDNA+	4	8	33.3%
Landmark ctDNA−	16	106	13.1%

H

p=0.017	Death +	Death -	Event rate
Landmark ctDNA+	4	8	33.3%
Landmark ctDNA−	9	113	7.4%

Figure EV3. Contingency tables for pCR and ctDNA variables versus survival outcomes.

End-NAT ctDNA status versus recurrence (**A**) and death (**B**). pCR status versus recurrence (**C**) and death (**D**). NAT ctDNA responder status versus recurrence (**E**) and death (**F**). Landmark ctDNA status versus recurrence (**G**) and death (**H**). *P*-values calculated using the Fisher's exact test.

© The Author(s)

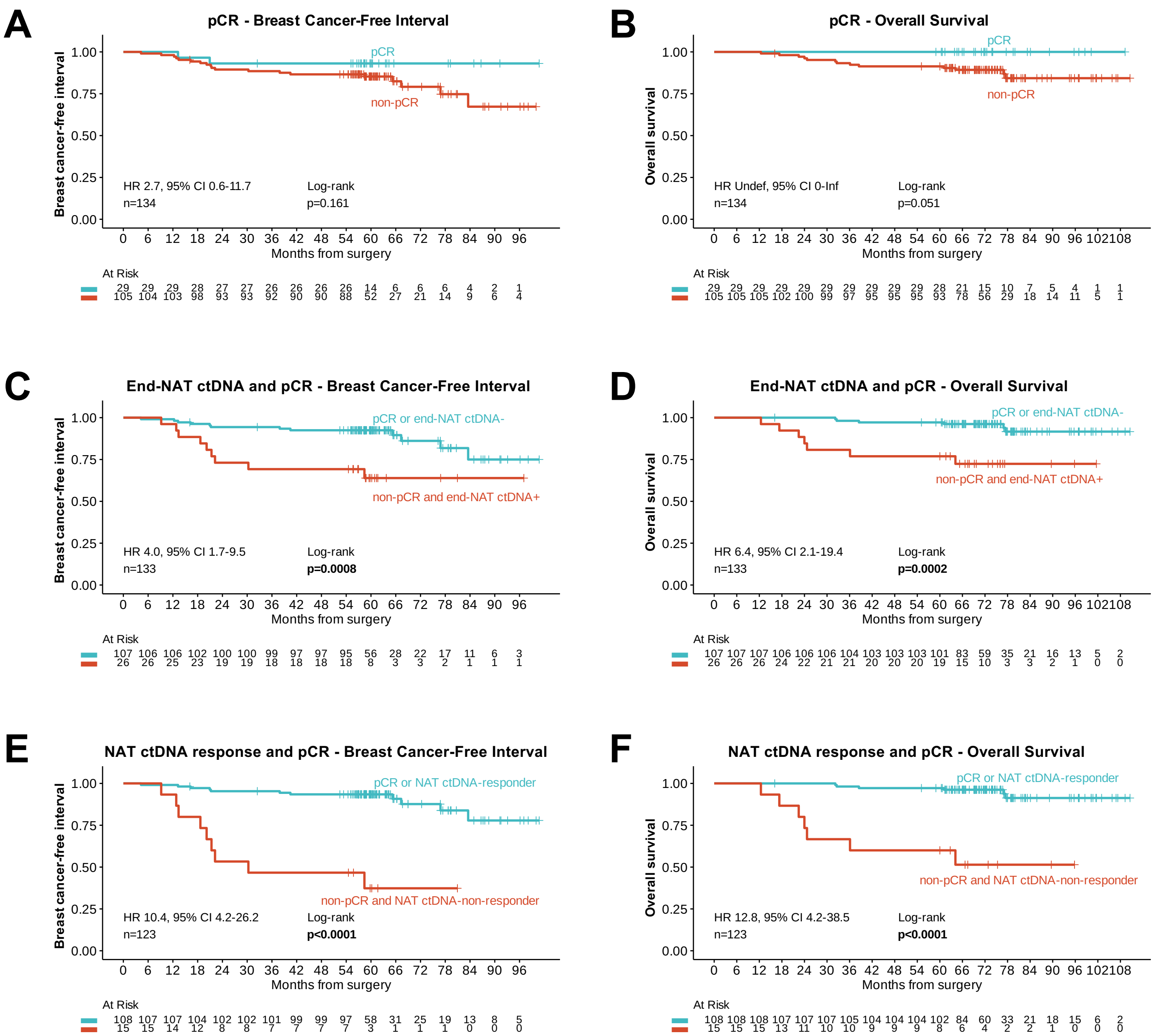

Figure EV4. Kaplan–Meier survival estimates for all patients.

For breast cancer-free interval (**A**, **C**, **E**) and overall survival (**B**, **D**, **F**), survival curves are plotted for: (**A**, **B**) pCR, (**C**, **D**) End-NAT ctDNA and pCR combined, and (**E**, **F**) NAT ctDNA response and pCR combined. *P*-values calculated using the log-rank test.

 © The Author(s)
