# Supplementary material for: NeoCircle: pre- and post-operative circulating tumor DNA dynamics predicts survival in neoadjuvant-treated early breast cancer
Source: EMBO Mol Med. 2026 May 26;18(7):2617–34. doi: 10.1038/s44321-026-00447-z (PMC13365804; doi:10.1038/s44321-026-00447-z)
Supplement: Supplementary file 2 — Peer Review File [file 44321_2026_447_MOESM2_ESM.pdf]

EMBO Molecular Medicine

NeoCircle: pre- and post-operative ctDNA predicts survival in neoadjuvant-treated breast cancer

Anthony George, Yilun Chen, Sergii Gladchuk, Miguel Alcaide, Hina Dalal, Pei Meng, Christian Brueffer, Hani Saghir, Siker Kimbung, Kristina Aaltonen, Lucia Oton, Christopher Rushton, Sofia Birkeälv, Mats Jönsson, Sophia Zackrisson, Ida Skarping, Daniel Förnvik, Lina Zander, Gabriella Honeth, Samuel Woodhouse, Karen Howarth, Åke Borg, Anna Ehinger, Martin Malmberg, Lisa Rydén, Niklas Loman, and Lao Saal

Corresponding author(s): Lao Saal (lao.saal@med.lu.se)

Review Timeline:

Submission Date:	10th Nov 25
Editorial Decision:	24th Dec 25
Revision Received:	27th Mar 26
Editorial Decision:	20th Apr 26
Revision Received:	24th Apr 26
Accepted:	27th Apr 26

Editor: Zeljko Durdevic

Transaction Report:

(Note: With the exception of the correction of typographical or spelling errors that could be a source of ambiguity, letters and reports are not edited. Depending on transfer agreements, referee reports obtained elsewhere may or may not be included in this compilation. Referee reports are anonymous unless the Referee chooses to sign their reports.)

24th Dec 2025

Dear Dr. Saal,

Thank you for the submission of your manuscript to EMBO Molecular Medicine, and please accept my apologies for the unusual delay in getting back to you. We have now received feedback from two of the three reviewers who agreed to evaluate your manuscript. As the referee #3 will unfortunately not be able to return his/her report in a timely manner, we prefer to make a decision now in order to avoid further delay in the process. Should referee #3 provide a report, we will send it to you, with the understanding that we will not ask for an additional revision.

As you will see from their reports pasted below, while the referee #1 supports publication raising minor concerns, referee #2 recognizes interest of the study but also raises serious concerns that should be addressed in a major revision. If you would like to discuss further the points raised by the referees, I am available to do so via email or video. Let me know if you are interested in this option.

Further consideration of a revision that addresses reviewers' concerns in full will entail a second round of review. EMBO Molecular Medicine encourages a single round of revision only and therefore, acceptance or rejection of the manuscript will depend on the completeness of your responses included in the next, final version of the manuscript. For this reason, and to save you from any frustrations in the end, I would strongly advise against returning an incomplete revision.

We would welcome the submission of a revised version within three months for further consideration. Please let us know if you require longer to complete the revision.

I look forward to receiving your revised manuscript.

Yours sincerely,

Zeljko Durdevic

Zeljko Durdevic
Senior Editor
EMBO Molecular Medicine

*****
When preparing your revised manuscript, please refer to our guidelines: https://link.springer.com/journal/44321/submission-guidelines#cms-Revised-submissions. We perform an initial quality control of all revised manuscripts before re-review; failure to include requested items will delay the evaluation of your revision.

We require:

1) A .docx formatted version of the manuscript text (including legends for main figures, EV figures and tables). Please make sure that the changes are highlighted to be clearly visible.

2) Individual production quality figure files as .eps, .tif, .jpg (one file per figure). For guidance, download the 'Figure Guide PDF': https://media.springernature.com/original/springer-cms/rest/v1/content/27825798/data/v1.

3) A .docx formatted letter INCLUDING the reviewers' reports and your detailed point-by-point responses to their comments. As part of the EMBO Press transparent editorial process, the point-by-point response is part of the Review Process File (RPF), which will be published alongside your paper.

4) A complete author checklist, which you can download from our author guidelines. Please insert information in the checklist that is also reflected in the manuscript. The completed author checklist will also be part of the RPF.

5) Please note that all corresponding authors are required to supply an ORCID ID for their name upon submission of a revised manuscript.

6) It is mandatory to include a 'Data Availability' section after the Materials and Methods. Before submitting your revision, primary datasets produced in this study need to be deposited in an appropriate public database, and the accession numbers and database listed under 'Data Availability'. Please remember to provide a reviewer password if the datasets are not yet public.

In case you have no data that requires deposition in a public database, please state so in this section. Note that the Data Availability Section is restricted to new primary data that are part of this study.

7) For data quantification: please specify the name of the statistical test used to generate error bars and P values, the number (n) of independent experiments (specify technical or biological replicates) underlying each data point and the test used to calculate p-values in each figure legend. The figure legends should contain a basic description of n, P and the test applied. Graphs must include a description of the bars and the error bars (s.d., s.e.m.).

8) At EMBO Press we ask authors to provide source data for the main manuscript figures. You will receive a separate email with instructions for providing source data with your revised manuscript, including how to upload and organize the files.

9) Our journal encourages inclusion of *data citations in the reference list* to directly cite datasets that were re-used and obtained from public databases. Data citations in the article text are distinct from normal bibliographical citations and should directly link to the database records from which the data can be accessed. In the main text, data citations are formatted as follows: "Data ref: Smith et al, 2001" or "Data ref: NCBI Sequence Read Archive PRJNA342805, 2017". In the Reference list, data citations must be labeled with "[DATASET]". A data reference must provide the database name, accession number/identifiers and a resolvable link to the landing page from which the data can be accessed at the end of the reference.

10) We replaced Supplementary Information with Expanded View (EV) Figures and Tables that are collapsible/expandable online. A maximum of 5 EV Figures can be typeset. EV Figures should be cited as 'Figure EV1, Figure EV2" etc... in the text and their respective legends should be included in the main text after the legends of regular figures.

- For the figures that you do NOT wish to display as Expanded View figures, they should be bundled together with their legends in a single PDF file called *Appendix*, which should start with a short Table of Content. Appendix figures should be referred to in the main text as: "Appendix Figure S1, Appendix Figure S2" etc.

- Additional Tables/Datasets should be labeled and referred to as Table EV1, Dataset EV1, etc. Legends have to be provided in a separate tab in case of .xls files. Alternatively, the legend can be supplied as a separate text file (README) and zipped together with the Table/Dataset file.

11) The paper explained: EMBO Molecular Medicine articles are accompanied by a summary of the articles to emphasize the major findings in the paper and their medical implications for the non-specialist reader. Please provide a draft summary of your article highlighting

- the medical issue you are addressing,

- the results obtained and

- their clinical impact.

This may be edited to ensure that readers understand the significance and context of the research. Please refer to any of our published articles for an example.

12) Author contributions: You will be asked to provide CRediT (Contributor Role Taxonomy) terms in the submission system. These replace a narrative author contribution section in the manuscript.

13) A Conflict of Interest statement should be provided in the main text.

14) Every published paper includes a 'Synopsis' to further enhance discoverability. Synopses are displayed on the journal webpage and are freely accessible to all readers. They include a short stand first (maximum of 300 characters, including space) as well as 2-5 one-sentences bullet points that summarizes the paper. Please write the bullet points to summarize the key NEW findings. They should be designed to be complementary to the abstract - i.e. not repeat the same text. We encourage inclusion of key acronyms and quantitative information (maximum of 30 words / bullet point). Please use the passive voice. Please attach these in a separate file or send them by email, we will incorporate them accordingly.

Please also suggest a striking image or visual abstract to illustrate your article as a PNG file 550 px wide x 300-600 px high.

15) Include a Reagents and Tools Table as part of the Methods section, which can be downloaded from our author guidelines.

EMBO Molecular Medicine has a "scooping protection" policy, whereby similar findings that are published by others during review or revision are not a criterion for rejection. Should you decide to submit a revised version, I do ask that you get in touch after three months if you have not completed it, to update us on the status.

***** Reviewer's comments *****

Referee #1 (Remarks for Author):

This study presents an original approach with a new technique for tumor-informed ctDNA detection of minimal residual disease (MRD). The authors demonstrated that they could achieve a high level of sensitivity and specificity in MRD detection using a novel digital multiplex PCR SV technique. This series is characterized by long follow-up, which provides the opportunity to detect more events in particular in the luminal breast cancer subtype. The clinical series is well documented in terms of patient characteristics and detailed outcomes (in supplementary data).
This work will make an important contribution to the rapidly evolving literature in this field.
Minor comment:
On page 4, in Figure 1, and on page 5, in the Results section, could the authors provide the number of cases excluded due to a lack of tumor or blood sample availability? It would be interesting to provide the total percentage of patients excluded for technical reasons (lack of tumor material, insufficient DNA quantity, or insufficient SVs in the fingerprint) out of the total number of patients enrolled. Is it 20% (34/170)? This information is important for evaluating tumor-informed ctDNA detection.
In Table 1, why did 35 patients receive anti-HER2 adjuvant treatment when only 32 patients had HER2-positive tumors?
In the discussion, the authors should reference the recent paper by I. Garcia-Murillas et al.
Ann. Oncol. 2025 Jun;36(6):673-681, "Whole genome sequencing-powered ctDNA sequencing for breast cancer detection."

Referee #2 (Comments on Novelty/Model System for Author):

The study is undoubtedly a valuable addition to the existing literature. Prospectively-collected data from a clinical trial conducted in a real-world setting ideally portrays the therapeutic journey of patients with early-stage breast cancer and strongly contribute to the growing body of evidence supporting clinical validity of MRD in this setting. Nonetheless, some further revisions and additions to the manuscript are needed to broaden discussion, put results into perspective, improve overall clarity and interpretability for readers.

Referee #2 (Remarks for Author):

Summary: This manuscript presents data from a prospective study conducted on patients with early-stage breast cancer. It focuses on circulating tumor DNA (ctDNA) detection, using a tumor-informed approach focused on structural variants (SVs) rather than single nucleotide variants only, and analyses its dynamic changes across pre- and post-operative treatment and follow-up, as well as potential associations with pathological response and clinical outcomes. Results nicely add to a growing body of evidence, pointing toward increased risk of disease recurrence or death in case of persistent ctDNA detection after neoadjuvant therapy and further suggest the clinical validity of molecular residual disease (MRD) monitoring.
Major Strengths:
• Prospective enrolment and follow-up of patients in a real-world context
• Adequate analytical workflows and study procedures
• Use of a novel, ultrasensitive, tumor-informed approach to ctDNA detection
• Focus on SVs, nice and detailed presentation of their advantages in the "Introduction"
Major Concerns:
1. Data contextualization: the manuscript currently lacks a clear description of how the data generated will fit in the expanding frame of MRD testing. A brief comment on the following topics would highly aid the readership to put data into perspective:
• how PathlightTM differs from other tumor-informed platforms (e.g., SignateraTM)
• advantages and disadvantages of dPCR approaches, also comparing with novel NGS-based tumor-agnostic assays
• can the detection of other analytes (e.g., CTCs) provide a more refined evaluation of minimal residual disease?
2. Treatment choice: authors state that enrolment occurred from December 2014 to March 2019; in the meantime, newer treatment options are being introduced for all disease subtypes, both in the neoadjuvant and in the adjuvant setting. This should be stated as a limitation; additionally, authors may discuss how newer options might sometimes overlap with established

standard-of-care treatments and whether ctDNA might have a role in refining more personalized escalation or de-escalation strategies.

3. Statistical analyses and data presentation: the last paragraph of the "Introduction" (pages 4-5) seems too long and a bit redundant and does not clearly state the aim(s) of the analysis. Although this seems to be the case, clearly pointing out in the manuscript whether the analyses are hypothesis-generating only would improve scientific soundness. Moreover, data reporting seems somewhat heterogeneous across the manuscript and would benefit from a thorough revision; inconsistencies include some 95% CIs missing (e.g., page 9), median follow-up being expressed in years while the range is provided in months (pages 5-6), incorrectly mentioning a nonsignificant trend of pCR toward less recurrence with a $p > 0.1$ (page 8) and some discordance between the number of patients declared in the "FFPE and FF workflows" (pages 17-18) and Figure 1A.

Minor Suggestions:

• Text would benefit from further revision and harmonization of some abbreviations, especially providing a clearer distinction between end-neoadjuvant therapy ctDNA status (positive vs negative) and definition of ctDNA responders vs non-responders

• Some parts of the "Results" section could be rephrased in a more direct way, in order to present results in a descriptive-only fashion and leave room for interpretation in the "Discussion". Moreover, authors might consider moving the last part of the "Tumor-informed SV breakpoint assay design" paragraph (page 6) in the "Discussion"

• In the "ctDNA and clinical course" paragraph, authors might need to clarify the "short course of trastuzumab" that patient P06091 received, as this approach is not unanimously endorsed by guidelines. It is also worth discussing the rationale for the 3-year zoledronic acid given to patient P09322 and it should be mentioned that, according to Supplementary Figure S3, the patient eventually died about 10 months after recurrence

• Authors might provide a few more details about the time windows allowed for blood samples' collection (besides, was the 24-month timepoint not collected or was it a typo?) and about patients who received modified regimens and the reason why it was chosen to do so (maybe it might be integrated in the Supplementary Table)

• In the "Patients" paragraph (pages 15-17), authors only mention performing mammogram and/or breast US at diagnosis and during follow-up; was breast MRI employed or considered for some patients?

• The legend of the Supplementary Figures should be updated, as it seems like some images have been moved to the main text

• Recommend some language revision, especially in terms of medical jargon (e.g., use "patients with early breast cancer" instead of "early breast cancer patients", in order to remark that we are treating patients, not tumors) and sentence length (some of them seem too long and could be broken into two; e.g., first paragraph in the "Results" section, page 5)

We thank the Editors and Reviewers for considering our manuscript and for the careful interpretation of our study and results. We appreciate the generally positive remarks on our paper and the novelty of our results. The reviewers' comments have been helpful in identifying areas where we can improve the presentation and contextualization of the results. Below we address each reviewers' comments and we have made the relevant changes to the manuscript (changes are shown in the markup version). We have also addressed all items related to the journal requirements, including reassigning supplementary information, adapting the figure panel naming and legends to journal style, and adding statistical test information to relevant legends. We note that we inadvertently had omitted one middle-author, who has now been included in the revised manuscript.

The following supplementary information in the original submission have been reassigned in our revised manuscript:

- Supplementary Figure S1 is now Figure EV1
- Supplementary Figure S2 is now Figure EV2
- Supplementary Figure S3 is now Appendix Figure S1
- Supplementary Figure S4 is now Figure EV3
- Supplementary Figure S5 is now Figure EV4
- Supplementary Figure S6 is now Appendix Figure S2
- Supplementary Figure S7 is now Appendix Figure S3
- Supplementary Figure S8 is now Appendix Figure S4
- Supplementary Table S1 is now Appendix Table S1

We believe we have addressed each of the reviewers' concerns, and in so doing, our manuscript has been significantly improved, and we hope it can now be judged to be acceptable for publication in *EMBO Molecular Medicine*.

Our point-by-point responses follow below:

Referee #1 (Remarks for Author):

This study presents an original approach with a new technique for tumor-informed ctDNA detection of minimal residual disease (MRD). The authors demonstrated that they could achieve a high level of sensitivity and specificity in MRD detection using a novel digital multiplex PCR SV technique. This series is characterized by long follow-up, which provides the opportunity to detect more events in particular in the luminal breast cancer subtype. The clinical series is well documented in terms of patient characteristics and detailed outcomes (in supplementary data).
This work will make an important contribution to the rapidly evolving literature in this field.

Response: We thank the reviewer for the careful reading of our manuscript, your very positive remarks with regards to our study and our tumor-informed multiplex dPCR assay

technology for MRD monitoring with high sensitivity and specificity, and for the helpful comments and questions.

Minor comment:

On page 4, in Figure 1, and on page 5, in the Results section, could the authors provide the number of cases excluded due to a lack of tumor or blood sample availability? It would be interesting to provide the total percentage of patients excluded for technical reasons (lack of tumor material, insufficient DNA quantity, or insufficient SVs in the fingerprint) out of the total number of patients enrolled. Is it 20% (34/170)? This information is important for evaluating tumor-informed ctDNA detection.

Response: Thank you for highlighting an aspect that was not clearly communicated in our original submission, that the number and proportion of cases excluded for technical reasons were not clearly reported. To address this, we have now revised Figure 1 and updated the Results and Methods sections to explicitly report the number of excluded cases and the reasons for exclusion (also in response to one of reviewer #2's remarks) (see markup version), thereby improving the clarity of the study flow and assay success rate.

In total, we enrolled 170 patients in NeoCircle, of whom 21 were excluded due to non-technical reasons (each reason enumerated in the revised Figure 1), leaving 149 patients eligible for ctDNA analysis. Of these, 13 (13/149=8.7%) could not be analyzed for ctDNA due to technical reasons: 5 with inadequate tumor content or insufficient quantity of tumor DNA, and 8 due to insufficient SVs in the fingerprint. Thus, in summary the technical exclusion rate was 13/170 (7.6%) of enrolled patients and 13/149 (8.7%) of patients available for analysis.

In Table 1, why did 35 patients receive anti-HER2 adjuvant treatment when only 32 patients had HER2-positive tumors?

Response: We thank the reviewer's careful attention here and can provide clarification for the discrepancy between number of patients with HER2-positive disease and the number that received anti-HER2 adjuvant treatment. The difference is explained by 3 individual cases:

1. Data annotation error (corrected): First, we identified an error in the study data where one patient (P00392) was incorrectly marked as having an ER+/HER2-negative tumor, when in fact the tumor was ER+/HER2-positive (consistent with her receiving anti-HER2 therapy both neoadjuvantly and adjuvantly). We have corrected this classification and updated all affected tables and figures (Table 1, Figure 3, Supplementary Figures S1, S3, S6, and S7, Supplementary Table S1, the latter of which are now reassigned as Figure EV1, Appendix Figures S1, S2, and S3, respectively), as well as recalculated the relevant hazard ratios, p-values, and other statistics, and made all necessary corrections to the manuscript text (see markup version). Importantly, this correction made no material difference to our results, with

all plots and statistics nearly identical in appearance, magnitude, and significance, and thus our scientific conclusions remain the same.

2. HER2 conversion after NAT: One patient (P05962) had a HER2-negative tumor at diagnostic biopsy (thus clinically HER2-negative and reported as such in Table 1 and elsewhere). However, the residual tumor at surgery was found to be HER2-positive, and adjuvant anti-HER2 treatment was consequently administered.

3. Focal HER2 amplification: The third patient (P04082) had a focal HER2 positivity (<10% of tumor cells). Although this patient was classified as clinically HER2-negative according to guideline criteria and therefore counted as HER2-negative in Table 1, the treating physician elected to administer adjuvant anti-HER2 therapy.

To clarify these points to the reader, we have added an explanatory note to Table 1.

In the discussion, the authors should reference the recent paper by I. Garcia-Murillas et al. Ann. Oncol. 2025 Jun;36(6):673-681, "Whole genome sequencing-powered ctDNA sequencing for breast cancer detection."

Response: Yes, we agree this important publication should be cited. We have now included this reference in the Discussion section, as well as three other relevant papers showing the potential clinical utility of ctDNA monitoring in the neoadjuvant and adjuvant settings in EBC (see markup version).

Referee #2 (Comments on Novelty/Model System for Author):

The study is undoubtedly a valuable addition to the existing literature. Prospectively-collected data from a clinical trial conducted in a real-world setting ideally portrays the therapeutic journey of patients with early-stage breast cancer and strongly contribute to the growing body of evidence supporting clinical validity of MRD in this setting. Nonetheless, some further revisions and additions to the manuscript are needed to broaden discussion, put results into perspective, improve overall clarity and interpretability for readers.

Response: We thank the reviewer for the thorough reading of our manuscript and constructive suggestions and questions, and for the praise that our study is undoubtedly a valuable addition to the existing literature.

Referee #2 (Remarks for Author):

Summary: This manuscript presents data from a prospective study conducted on patients with early-stage breast cancer. It focuses on circulating tumor DNA (ctDNA) detection, using a

tumor-informed approach focused on structural variants (SVs) rather than single nucleotide variants only, and analyses its dynamic changes across pre- and post-operative treatment and follow-up, as well as potential associations with pathological response and clinical outcomes. Results nicely add to a growing body of evidence, pointing toward increased risk of disease recurrence or death in case of persistent ctDNA detection after neoadjuvant therapy and further suggest the clinical validity of molecular residual disease (MRD) monitoring.
Major Strengths:
• Prospective enrolment and follow-up of patients in a real-world context
• Adequate analytical workflows and study procedures
• Use of a novel, ultrasensitive, tumor-informed approach to ctDNA detection
• Focus on SVs, nice and detailed presentation of their advantages in the "Introduction"

Response: We appreciate this excellent overview of our study, our results, and itemized highlights of its major strengths.

Major Concerns:
1. Data contextualization: the manuscript currently lacks a clear description of how the data generated will fit in the expanding frame of MRD testing. A brief comment on the following topics would highly aid the readership to put data into perspective:
• how PathlightTM differs from other tumor-informed platforms (e.g., SignateraTM)
• advantages and disadvantages of dPCR approaches, also comparing with novel NGS-based tumor-agnostic assays
• can the detection of other analytes (e.g., CTCs) provide a more refined evaluation of minimal residual disease?

Response: The reviewer makes a good point that we could improve the contextualization of our study and how our methods compare to other approaches. To address this comment, we have made a number of significant additions and textual changes within the Introduction section (see markup version).

Specifically, we now discuss (with additional references):

- the distinction between tumor-informed vs. tumor-naïve approaches and between SNV vs. SV approaches; and
- the relative advantages of NGS vs. dPCR for ctDNA detection.

These additions help position our SV-based dPCR approach within the broader MRD testing landscape.

With respect to the reviewer's suggestion regarding other analytes such as CTCs, we agree that multimodal approaches have a place in MRD assessment. However, because the present study focuses specifically on ctDNA-based detection, and given space limitations, we felt that an expanded discussion of alternative analytes would fall outside the scope of the manuscript. We have therefore limited the discussion to ctDNA-based MRD methodologies.

2. Treatment choice: authors state that enrolment occurred from December 2014 to March 2019; in the meantime, newer treatment options are being introduced for all disease subtypes, both in the neoadjuvant and in the adjuvant setting. This should be stated as a limitation; additionally, authors may discuss how newer options might sometimes overlap with established standard-of-care treatments and whether ctDNA might have a role in refining more personalized escalation or de-escalation strategies.

Response: Thank you for this comment. We agree that systemic treatment strategies for EBC have evolved since the enrollment period of the NeoCircle study (2014-2019), including the introduction of newer/alternative neoadjuvant and adjuvant treatment options. We have therefore revised the Discussion section to further contextualize the changing treatment landscape and incorporate the reviewer's suggestions.

In addition, we have expanded the Discussion to briefly address how molecular monitoring by liquid biopsies could complement modern treatment paradigms, particularly in guiding personalized treatment choice as well as its escalation or de-escalation. These changes we believe improve the manuscript.

3. Statistical analyses and data presentation: the last paragraph of the "Introduction" (pages 4-5) seems too long and a bit redundant and does not clearly state the aim(s) of the analysis. Although this seems to be the case, clearly pointing out in the manuscript whether the analyses are hypothesis-generating only would improve scientific soundness.

Response: We thank the reviewer for this helpful comment. We have amended the Introduction section considerably to address this and this reviewer's comment #1, and feel the section now reads better and provides improved contextualization of the methods and the planned as well as exploratory analyses performed, and in this context the last paragraph consisting of only two sentences fits well.

Specifically, we make it clearer that the NeoCircle study aims to:

- evaluate the clinical validity of SV-based ctDNA detection in EBC;
- evaluate the usefulness of this approach for assessment of response to NAT and its prognostic value; and
- validate the ability of this approach to detect MRD during post-operative follow-up.

As stated in the Results section 'ctDNA dynamics during neoadjuvant therapy', the majority of analyses were pre-specified (planned), and the exploratory analyses are indicated in the relevant parts of the Results.

Moreover, data reporting seems somewhat heterogeneous across the manuscript and would benefit from a thorough revision; inconsistencies include some 95% CIs missing (e.g., page 9), median follow-up being expressed in years while the range is provided in months (pages 5-6), incorrectly mentioning a nonsignificant trend of pCR toward less recurrence with a p > 0.1 (page 8) and some discordance between the number of patients declared in the "FFPE and FF workflows" (pages 17-18) and Figure 1A.

Response: We agree that the data reporting could be harmonized and improved. Although some of the original presentation is based on stylistic choices that we thought made the paper more readable, to address this reviewer comment we have elected to provide complete textual summaries and CIs in all relevant parts of the Results (see markup version).

Moreover, we have corrected the statement referring to a "non-significant trend" in pCR.

Finally, we clarified the patient numbers in the "FFPE and FF workflows" section and Figure 1A. These numbers reflected counts before and after specific exclusions, which may have caused confusion in the original presentation. The text and figure have now been revised to clearly indicate the relevant numbers (see markup version; see also response to Reviewer #1).

Minor Suggestions:

• Text would benefit from further revision and harmonization of some abbreviations, especially providing a clearer distinction between end-neoadjuvant therapy ctDNA status (positive vs negative) and definition of ctDNA responders vs non-responders

Response: Thank you for identifying an instance where the definitions could be made clearer. We have made minor amendments to the text where these variables are defined to address this comment and believe it is now easier to follow. We have also reviewed the text and figures carefully to ensure the terminology and abbreviations are harmonized throughout.

• Some parts of the "Results" section could be rephrased in a more direct way, in order to present results in a descriptive-only fashion and leave room for interpretation in the "Discussion". Moreover, authors might consider moving the last part of the "Tumor-informed SV breakpoint assay design" paragraph (page 6) in the "Discussion"

Response: While we appreciate the reviewer's comment, we find this to be primarily a stylistic choice and find this paper is easier to read and follow when some relevant context is, on occasion where we think it helpful, provided in the Results section.

With respect to the "Tumor-informed SV breakpoint assay design" section, we considered relocating this text to the Discussion as suggested. However, we believe that providing a brief description of the assay and its performance at this point in the Results section is helpful to the reader in contextualizing and interpreting the subsequent analyses. For this reason, we have retained this material in Results section.

• In the "ctDNA and clinical course" paragraph, authors might need to clarify the "short course of trastuzumab" that patient P06091 received, as this approach is not unanimously endorsed by guidelines. It is also worth discussing the rationale for the 3-year zoledronic acid given to patient P09322 and it should be mentioned that, according to Supplementary Figure S3, the patient eventually died about 10 months after recurrence

Response: Thank you for drawing attention to these points and the need to clarify the relevant details in the manuscript.

The short course of trastuzumab for patient P06091 was due to treatment discontinuation following cardiotoxicity; this additional information has been added to the text.

For patient P09322, we identified an error in the end date of zoledronic acid. This has now been corrected in Appendix Figure S1 (named Supplementary Figure S3 in the original submission). ZA was last administered at about the time of clinical relapse, and for visualization purposes we depict a duration of 6 months for each dose.

Finally, we have added to the text that this patient died about 10 months after recurrence (see markup version).

• Authors might provide a few more details about the time windows allowed for blood samples' collection (besides, was the 24-month timepoint not collected or was it a typo?) and about patients who received modified regimens and the reason why it was chosen to do so (maybe it might be integrated in the Supplementary Table)

Response: Thank you for the close reading. The listing of the standard blood sampling timepoints collected in the original manuscript contained an error, which has now been corrected (see markup version). We have also clarified in the Methods section that blood samples were generally collected within +/- 1 month of scheduled timepoints, although some deviations or missed collections occurred (e.g. during the COVID-19 pandemic).

Regarding the modified treatment regimes, patients in the NeoCircle study were treated according to standard-of-care Swedish national guidelines, where treatment adjustments may occur in the routine clinical practice for a variety of patient-specific reasons in the real-world setting (e.g., toxicities, comorbidities, and patient preference). Because the present study focuses on the performance of ctDNA monitoring rather than treatment optimization, we find that a detailed explanation of each treatment regime or modification to be beyond the scope of the study and does not affect our results. For the interested reader, the complete overview of treatments administered for each patient are provided in Appendix Figure S1 and Appendix Table S1 (respectively named Supplementary Figure S3 and Supplementary Table S1 in the original submission).

• In the "Patients" paragraph (pages 15-17), authors only mention performing mammogram and/or breast US at diagnosis and during follow-up; was breast MRI employed or considered for some patients?

Response: MRI is not part of the standard routine follow-up for women treated with EBC in Sweden. In hereditary high-risk breast cancer, MRI may be utilized in high-risk surveillance programs (often annual MRI alternating with mammography). In younger women with extremely dense breasts, MRI may be considered if mammographic sensitivity is limited and clinical suspicion exists. MRI may also be performed when there is equivocal mammography/ultrasound, there is clinical suspicion of recurrence but negative conventional imaging, or there are post-surgical or radiation changes that obscure evaluation. We have added these details to the revised manuscript within the Methods section on 'Patients'.

• The legend of the Supplementary Figures should be updated, as it seems like some images have been moved to the main text

Response: Thank you for identifying a discrepancy and helping us to improve our paper. Some supplementary panels had been moved to the main text shortly before the original submission, but the corresponding Supplementary Figure legends were not updated accordingly (now named EV figures and Appendix Figures). This has now been corrected in the revised manuscript.

• Recommend some language revision, especially in terms of medical jargon (e.g., use "patients with early breast cancer" instead of "early breast cancer patients", in order to remark that we are treating patients, not tumors) and sentence length (some of them seem too long and could be broken into two; e.g., first paragraph in the "Results" section, page 5)

Response: Thank you for this comment. We have carefully reviewed the entire manuscript and revised the language where appropriate to ensure patient-centered phrasing. We have also edited several sentences throughout the manuscript, to shorten or divide overly long sentences to improve readability.

20th Apr 2026

Dear Dr. Saal,

Thank you for submitting your revised manuscript to EMBO Molecular Medicine. Your manuscript will be ready for acceptance pending the following final amendments:

1) Please address referee's minor comment.
2) In the main manuscript file, please do the following:
- Please address all comments suggested by our data editors listed below:
o Figure legends:
1. Please note that the exact p values are not provided in the legends of figures 4B, D, E; 6A, B.
2. Please note that the box plots need to be defined in terms of minima, maxima, centre, bounds of box and whiskers, and percentile in the legends of figures 2A, 3E, EV1, EV2 A-F.
- Add panel callouts for Fig 2A,B, Fig 5A,B, Fig EV2, EV3 and EV4.
- Indicate in legends number and nature of replicates and exact p= values, not a range, along with the statistical test used. To keep the figures "clear" some authors found providing an Appendix table Sx with all exact p-values preferable. You are welcome to do this if you want to.
- Please remove the Reagent Table from the manuscript and uploaded it as a separate .doc file.
- Author contributions: Please remove it from the manuscript and specify author contributions in our submission system. CRediT has replaced the traditional author contributions section because it offers a systematic machine-readable author contributions format that allows for more effective research assessment. You are encouraged to use the free text boxes beneath each contributing author's name to add specific details on the author's contribution. More information is available in our guide to authors:
https://www.embopress.org/page/journal/17574684/authorguide#authorshipguidelines
3) Funding: Please add project numbers where available. This list will be linked to PuBMed in published papers, it is therefore important that the list is complete and accurate.
4) Synopsis:
- Synopsis image: Please simplify the image and upload it as a high-resolution jpeg file, exactly 550 px-wide x 300 pixels high.
- Please check your synopsis text and image before submission with your revised manuscript. Please be aware that in the proof stage minor corrections only are allowed (e.g., typos).
5) As part of the EMBO Publications transparent editorial process (see our Editorial at http://embomolmed.embopress.org/content/2/9/329), EMBO Molecular Medicine will publish online a Review Process File (RPF) to accompany accepted manuscripts. This file will be published in conjunction with your paper and will include the anonymous referee reports, your point-by-point response and all pertinent correspondence relating to the manuscript. Let us know if you want to remove or not any figures from it prior to publication. Please note that the Authors checklist will be published at the end of the RPF.
6) Please provide a point-by-point letter INCLUDING my comments as well as the reviewer's reports and your detailed responses (as Word file).

I look forward to reading a new revised version of your manuscript as soon as possible.

Yours sincerely,

Zeljko Durdevic

Zeljko Durdevic
Senior Editor
EMBO Molecular Medicine

*** Instructions to submit your revised manuscript ***

*** PLEASE NOTE *** As part of the EMBO Publications transparent editorial process initiative (see our Editorial at https://www.embopress.org/doi/pdf/10.1002/emmm.201000094), EMBO Molecular Medicine will publish online a Review Process File to accompany accepted manuscripts.

In the event of acceptance, this file will be published in conjunction with your paper and will include the anonymous referee

reports, your point-by-point response and all pertinent correspondence relating to the manuscript. If you do NOT want this file to be published, please inform the editorial office at contact@embomolmed.org.

*****

When preparing your revised manuscript, please refer to our guidelines: https://link.springer.com/journal/44321/submission-guidelines#cms-Revised-submissions. We perform an initial quality control of all revised manuscripts before re-review; failure to include requested items will delay the evaluation of your revision.

We require:

1) A .docx formatted version of the manuscript text (including legends for main figures, EV figures and tables). Please make sure that the changes are highlighted to be clearly visible.

2) Individual production quality figure files as .eps, .tif, .jpg (one file per figure). For guidance, download the 'Figure Guide PDF': https://media.springernature.com/original/springer-cms/rest/v1/content/27825798/data/v1.

3) A .docx formatted letter INCLUDING the reviewers' reports and your detailed point-by-point responses to their comments. As part of the EMBO Press transparent editorial process, the point-by-point response is part of the Review Process File (RPF), which will be published alongside your paper.

4) A complete author checklist, which you can download from our author guidelines. Please insert information in the checklist that is also reflected in the manuscript. The completed author checklist will also be part of the RPF.

5) Please note that all corresponding authors are required to supply an ORCID ID for their name upon submission of a revised manuscript.

6) It is mandatory to include a 'Data Availability' section after the Materials and Methods. Before submitting your revision, primary datasets produced in this study need to be deposited in an appropriate public database, and the accession numbers and database listed under 'Data Availability'. Please remember to provide a reviewer password if the datasets are not yet public.

In case you have no data that requires deposition in a public database, please state so in this section. Note that the Data Availability Section is restricted to new primary data that are part of this study.

7) For data quantification: please specify the name of the statistical test used to generate error bars and P values, the number (n) of independent experiments (specify technical or biological replicates) underlying each data point and the test used to calculate p-values in each figure legend. The figure legends should contain a basic description of n, P and the test applied. Graphs must include a description of the bars and the error bars (s.d., s.e.m.).

8) At EMBO Press we ask authors to provide source data for the main manuscript figures. You will receive a separate email with instructions for providing source data with your revised manuscript, including how to upload and organize the files.

9) Our journal encourages inclusion of *data citations in the reference list* to directly cite datasets that were re-used and obtained from public databases. Data citations in the article text are distinct from normal bibliographical citations and should directly link to the database records from which the data can be accessed. In the main text, data citations are formatted as follows: "Data ref: Smith et al, 2001" or "Data ref: NCBI Sequence Read Archive PRJNA342805, 2017". In the Reference list, data citations must be labeled with "[DATASET]". A data reference must provide the database name, accession number/identifiers and a resolvable link to the landing page from which the data can be accessed at the end of the reference.

10) We replaced Supplementary Information with Expanded View (EV) Figures and Tables that are collapsible/expandable online. A maximum of 5 EV Figures can be typeset. EV Figures should be cited as 'Figure EV1, Figure EV2" etc... in the text and their respective legends should be included in the main text after the legends of regular figures.

- For the figures that you do NOT wish to display as Expanded View figures, they should be bundled together with their legends in a single PDF file called *Appendix*, which should start with a short Table of Content. Appendix figures should be referred to in the main text as: "Appendix Figure S1, Appendix Figure S2" etc.

- Additional Tables/Datasets should be labeled and referred to as Table EV1, Dataset EV1, etc. Legends have to be provided in a separate tab in case of .xls files. Alternatively, the legend can be supplied as a separate text file (README) and zipped

together with the Table/Dataset file.

11) The paper explained: EMBO Molecular Medicine articles are accompanied by a summary of the articles to emphasize the major findings in the paper and their medical implications for the non-specialist reader. Please provide a draft summary of your article highlighting

- the medical issue you are addressing,

- the results obtained and

- their clinical impact.

This may be edited to ensure that readers understand the significance and context of the research. Please refer to any of our published articles for an example.

12) Author contributions: You will be asked to provide CRediT (Contributor Role Taxonomy) terms in the submission system. These replace a narrative author contribution section in the manuscript.

13) A Conflict of Interest statement should be provided in the main text.

14) Every published paper includes a 'Synopsis' to further enhance discoverability. Synopses are displayed on the journal webpage and are freely accessible to all readers. They include a short stand first (maximum of 300 characters, including space) as well as 2-5 one-sentences bullet points that summarizes the paper. Please write the bullet points to summarize the key NEW findings. They should be designed to be complementary to the abstract - i.e. not repeat the same text. We encourage inclusion of key acronyms and quantitative information (maximum of 30 words / bullet point). Please use the passive voice. Please attach these in a separate file or send them by email, we will incorporate them accordingly.

Please also suggest a striking image or visual abstract to illustrate your article as a PNG file 550 px wide x 300 px high.

15) Include a Reagents and Tools Table as part of the Methods section, which can be downloaded from our author guidelines.

EMBO Molecular Medicine has a "scooping protection" policy, whereby similar findings that are published by others during review or revision are not a criterion for rejection. Should you decide to submit a revised version, I do ask that you get in touch after three months if you have not completed it, to update us on the status.

*Additional important information regarding Figures

Each figure should be given in a separate file and should have the following resolution:
Graphs 800-1,200 DPI
Photos 400-800 DPI
Colour (only CMYK) 300-400 DPI"

Figures are not edited by the production team. All lettering should be the same size and style; figure panels should be indicated by capital letters (A, B, C etc). Gridlines are not allowed except for log plots. Figures should be numbered in the order of their appearance in the text with Arabic numerals. Each Figure must have a separate legend and a caption is needed for each panel.

*Additional important information regarding figures and illustrations can be found at
https://media.springernature.com/original/springer-cms/rest/v1/content/27825798/data/v1

***** Reviewer's comments *****

Referee #1 (Remarks for Author):

The authors should be thanked for revising the manuscript, which has improved its overall quality.

Minor comment:
In Figure 1A, the number of unavailable tumor samples should be reported separately rather than combined with blood samples. This distinction is necessary to accurately determine the total number of cases lacking tumor information, which represents a key limitation of tumor-informed ctDNA detection.

We thank the reviewers and editors for carefully reading out revised manuscript and for your comments and instructions, and are pleased that our paper is acceptable for publication pending final amendments.

Below, we provide a point-by-point response to the reviewer comment and editorial instructions:

1) Please address referee's minor comment.

Minor comment:
In Figure 1A, the number of unavailable tumor samples should be reported separately rather than combined with blood samples. This distinction is necessary to accurately determine the total number of cases lacking tumor information, which represents a key limitation of tumor-informed ctDNA detection.

Response: We have revised Figure 1A to indicate the number of cases excluded due to lack of available tumor (7) or blood samples (9).

2) In the main manuscript file, please do the following:
- Please address all comments suggested by our data editors listed below:
o Figure legends:
1. Please note that the exact p values are not provided in the legends of figures 4B, D, E; 6A, B.

Response: As suggested by the editors as an option (see below), we now have included a new Appendix Table S2 that contains all the missing exact p-values.

2. Please note that the box plots need to be defined in terms of minima, maxima, centre, bounds of box and whiskers, and percentile in the legends of figures 2A, 3E, EV1, EV2 A-F.

Response: For the indicated figures with boxplots, we have amended the figure legends and now provide the definitions for the boxplots. The following text has been added: "All values are plotted in the boxplot, with the median (center line), interquartile 75th to 25th percentile range (box), and 1.5 times the interquartile range (whiskers) indicated.").

- Add panel callouts for Fig 2A,B, Fig 5A,B, Fig EV2, EV3 and EV4.

Response: We have gone through the manuscript text and added specific callouts for each of the figure panels which were missing callouts.

- Indicate in legends number and nature of replicates and exact p= values, not a range, along with the statistical test used. To keep the figures "clear" some authors found providing an

Appendix table Sx with all exact p-values preferable. You are welcome to do this if you want to.

Response: Thank you for this suggestion. We have chosen to provide all missing exact p-values in the new Appendix Table S2, which indicates the figure, panel, exact p-value, and statistical test used. Note, information on replicates is not relevant for our type of data.

- Please remove the Reagent Table from the manuscript and uploaded it as a separate .doc file.

Response: We have removed the Reagent Table from the manuscript and placed it in a separate Word document.

- Author contributions: Please remove it from the manuscript and specify author contributions in our submission system. CRediT has replaced the traditional author contributions section because it offers a systematic machine-readable author contributions format that allows for more effective research assessment. You are encouraged to use the free text boxes beneath each contributing author's name to add specific details on the author's contribution. More information is available in our guide to authors: https://www.embopress.org/page/journal/17574684/authorguide#authorshipguidelines

Response: We have removed the Author contributions section from the manuscript and double checked the specified author contributions that were input in the submission system.

3) Funding: Please add project numbers where available. This list will be linked to PuBMed in published papers, it is therefore important that the list is complete and accurate.

Response: The funding list in the submission system and manuscript text has been reviewed and is complete.

4) Synopsis:
- Synopsis image: Please simplify the image and upload it as a high-resolution jpeg file, exactly 550 px-wide x 300 pixels high.
- Please check your synopsis text and image before submission with your revised manuscript. Please be aware that in the proof stage minor corrections only are allowed (e.g., typos).

Response: We have now revised the synopsis image, simplifying it and provide it as a 550x300 pixel jpeg image, and we have reviewed the synopsis text.

5) As part of the EMBO Publications transparent editorial process (see our Editorial at http://embomolmed.embopress.org/content/2/9/329), EMBO Molecular Medicine will

publish online a Review Process File (RPF) to accompany accepted manuscripts. This file will be published in conjunction with your paper and will include the anonymous referee reports, your point-by-point response and all pertinent correspondence relating to the manuscript. Let us know if you want to remove or not any figures from it prior to publication. Please note that the Authors checklist will be published at the end of the RPF.

Response: Thank you for this notification. We do not request any removals of figures or information from the Review Profess File.

6) Please provide a point-by-point letter INCLUDING my comments as well as the reviewer's reports and your detailed responses (as Word file).

Response: Done.

We again thank the editorial team and reviewers for a smooth and professional submission and revision process.

27th Apr 2026

Dear Dr. Saal,

We are pleased to inform you that your manuscript is accepted for publication and is now being sent to our publisher to be included in the next available issue of EMBO Molecular Medicine.

Your manuscript will be processed for publication by EMBO Press. It will be copy edited and you will receive page proofs prior to publication. Please note that you will be contacted by Springer Nature Author Services to complete licensing and payment information.

You may qualify for financial assistance for your publication charges - either via a Springer Nature fully open access agreement or an EMBO initiative. Check your eligibility: https://link.springer.com/journal/44321/how-to-publish-with-us

Should you be planning a Press Release on your article, please get in contact with embo_production@springernature.com as early as possible in order to coordinate publication and release dates.

If you have any questions, please do not hesitate to contact the Editorial Office. Thank you for your contribution to EMBO Molecular Medicine.

Yours sincerely,
Zeljko Durdevic

Zeljko Durdevic
Senior Editor
EMBO Molecular Medicine

>>> Please note that it is EMBO Molecular Medicine policy for the transcript of the editorial process (containing referee reports and your response letter) to be published as an online supplement to each paper. If you do NOT want this, you will need to inform the Editorial Office via email immediately. More information is available here: https://link.springer.com/partners/embo-press/editorial-policies#Peer%20review

EMBO Press Author Checklist

Corresponding Author Name: Lao Saal	
Journal Submitted to: EMBO Molecular Medicine	
Manuscript Number: EMM-2025-22956	

USEFUL LINKS FOR COMPLETING THIS FORM
The EMBO Journal - Author Guidelines
EMBO Reports - Author Guidelines
Molecular Systems Biology - Author Guidelines
EMBO Molecular Medicine - Author Guidelines

Reporting Checklist for Life Science Articles (updated January
This checklist is adapted from Materials Design Analysis Reporting (MDAR) Checklist for Authors. MDAR establishes a minimum set of requirements in transparent reporting in the life sciences (see Statement of Task: 10.31222/osf.io/9sm4x). Please follow the journal's guidelines in preparing your
Please note that a copy of this checklist will be published alongside your article.

Abridged guidelines for figures
1. Data
The data shown in figures should satisfy the following conditions:
- the data were obtained and processed according to the field's best practice and are presented to reflect the results of the experiments in an accurate and unbiased manner.
- ideally, figure panels should include only measurements that are directly comparable to each other and obtained with the same assay.
- plots include clearly labeled error bars for independent experiments and sample sizes. Unless justified, error bars should not be shown for technical
- if n<5, the individual data points from each experiment should be plotted. Any statistical test employed should be justified.
- Source Data should be included to report the data underlying figures according to the guidelines set out in the authorship guidelines on Data

2. Captions
Each figure caption should contain the following information, for each panel where they are relevant:
- a specification of the experimental system investigated (eg cell line, species name).
- the assay(s) and method(s) used to carry out the reported observations and measurements.
- an explicit mention of the biological and chemical entity(ies) that are being measured.
- an explicit mention of the biological and chemical entity(ies) that are altered/varied/perturbed in a controlled manner.
- the exact sample size (n) for each experimental group/condition, given as a number, not a range;
- a description of the sample collection allowing the reader to understand whether the samples represent technical or biological replicates (including how many animals, litters, cultures, etc.).
- a statement of how many times the experiment shown was independently replicated in the laboratory.
- definitions of statistical methods and measures:
  - common tests, such as t-test (please specify whether paired vs. unpaired), simple χ2 tests, Wilcoxon and Mann-Whitney tests, can be unambiguously identified by name only, but more complex techniques should be described in the methods section;
  - are tests one-sided or two-sided?
  - are there adjustments for multiple comparisons?
  - exact statistical test results, e.g., P values = x but not P values < x;
  - definition of 'center values' as median or average;
  - definition of error bars as s.d. or s.e.m.

Please complete ALL of the questions below.
Select "Not Applicable" only when the requested information is not relevant for your study.

Materials

Newly Created Materials	Information included in the manuscript?	In which section is the information available? (Reagents and Tools Table, Materials and Methods, Figures, Data Availability Section)
New materials and reagents need to be available; do any restrictions apply?	Not Applicable	

Antibodies	Information included in the manuscript?	In which section is the information available? (Reagents and Tools Table, Materials and Methods, Figures, Data Availability Section)
For **antibodies** provide the following information:<br>- Commercial antibodies: RRID (if possible) or supplier name, catalogue number and or/clone number<br>- Non-commercial: RRID or citation	Not Applicable	

DNA and RNA sequences	Information included in the manuscript?	In which section is the information available? (Reagents and Tools Table, Materials and Methods, Figures, Data Availability Section)
**Short novel DNA or RNA including primers, probes**: provide the sequences.	Not Applicable	

Cell materials	Information included in the manuscript?	In which section is the information available? (Reagents and Tools Table, Materials and Methods, Figures, Data Availability Section)
**Cell lines:** Provide species information, strain. Provide accession number in repository **OR** supplier name, catalog number, clone number, and/**OR** RRID.	Not Applicable	
**Primary cultures:** Provide species, strain, sex of origin, genetic modification status.	Not Applicable	
Report if the cell lines were recently **authenticated** (e.g., by STR profiling) and tested for mycoplasma contamination.	Not Applicable	

Experimental animals	Information included in the manuscript?	In which section is the information available? (Reagents and Tools Table, Materials and Methods, Figures, Data Availability Section)
**Laboratory animals or Model organisms:** Provide species, strain, sex, age, genetic modification status. Provide accession number in repository **OR** supplier name, catalog number, clone number, **OR** RRID.	Not Applicable	
**Animal observed in or captured from the field:** Provide species, sex, and age where possible.	Not Applicable	
Please detail **housing and husbandry conditions**.	Not Applicable	

Plants and microbes	Information included in the manuscript?	In which section is the information available? (Reagents and Tools Table, Materials and Methods, Figures, Data Availability Section)
**Plants:** provide species and strain, ecotype and cultivar where relevant, unique accession number if available, and source (including location for collected wild specimens).	Not Applicable	
**Microbes:** provide species and strain, unique accession number if available, and source.	Not Applicable	

Human research participants	Information included in the manuscript?	In which section is the information available? (Reagents and Tools Table, Materials and Methods, Figures, Data Availability Section)
If collected and within the bounds of privacy constraints report on age, sex and gender or ethnicity for all study participants.	Yes	Methods section, Table 1, Appendix Table S1

Core facilities	Information included in the manuscript?	In which section is the information available? (Reagents and Tools Table, Materials and Methods, Figures, Data Availability Section)
If your work benefited from core facilities, was their service mentioned in the acknowledgments section?	Yes	Acknowledgements section

Design

Study protocol	Information included in the manuscript?	In which section is the information available? (Reagents and Tools Table, Materials and Methods, Figures, Data Availability Section)
If study protocol has been **pre-registered, provide DOI in the manuscript**. For clinical trials, provide the trial registration number **OR** cite DOI.	Not Applicable	
Report the **clinical trial registration number** (at ClinicalTrials.gov or equivalent), where applicable.	Yes	Abstract, Introduction, and Methods sections

Laboratory protocol	Information included in the manuscript?	In which section is the information available? (Reagents and Tools Table, Materials and Methods, Figures, Data Availability Section)
Provide DOI OR other citation details if **external detailed step-by-step protocols** are available.	Not Applicable	

Experimental study design and statistics	Information included in the manuscript?	In which section is the information available? (Reagents and Tools Table, Materials and Methods, Figures, Data Availability Section)
Include a statement about **sample size** estimate even if no statistical methods were used.	Not Applicable	
Were any steps taken to minimize the effects of subjective bias when allocating animals/samples to treatment (e.g. **randomization procedure**)? If yes, have they been described?	Not Applicable	
Include a statement about **blinding** even if no blinding was done.	Yes	Methods section
Describe **inclusion/exclusion criteria** if samples or animals were excluded from the analysis. Were the criteria pre-established?<br><br>If sample or data points were omitted from analysis, report if this was due to attrition or intentional exclusion and provide justification.	Yes	Methods section, Figure 1
For every figure, are **statistical tests** justified as appropriate? Do the data meet the assumptions of the tests (e.g., normal distribution)? Describe any methods used to assess it. Is there an estimate of variation within each group of data? Is the variance similar between the groups that are being statistically compared?	Yes	Statistical test(s) used are described in each figure legend, and details of statistical analyses provided in Methods section

Sample definition and in-laboratory replication	Information included in the manuscript?	In which section is the information available? (Reagents and Tools Table, Materials and Methods, Figures, Data Availability Section)
In the figure legends: state number of times the experiment was **replicated** in laboratory.	Not Applicable	
In the figure legends: define whether data describe **technical or biological replicates**.	Not Applicable	

Ethics

Ethics	Information included in the manuscript?	In which section is the information available? (Reagents and Tools Table, Materials and Methods, Figures, Data Availability Section)
Studies involving **human participants**: State details of **authority granting ethics approval** (IRB or equivalent committee(s), provide reference number for approval.	Yes	Methods section
Studies involving **human participants**: Include a statement confirming that **informed consent** was obtained from all subjects and that the experiments conformed to the principles set out in the WMA Declaration of Helsinki and the Department of Health and Human Services Belmont Report.	Yes	Methods section
Studies involving **human participants:** For publication of **patient photos**, include a statement confirming that consent to publish was obtained.	Not Applicable	
Studies involving experimental **animals**: State details of **authority granting ethics approval** (IRB or equivalent committee(s), provide reference number for approval. Include a statement of compliance with ethical regulations.	Not Applicable	
Studies involving **specimen and field samples:** State if relevant **permits** obtained, provide details of authority approving study; if none were required, explain why.	Not Applicable	

Dual Use Research of Concern (DURC)	Information included in the manuscript?	In which section is the information available? (Reagents and Tools Table, Materials and Methods, Figures, Data Availability Section)
Could your study fall under dual use research restrictions? Please check biosecurity documents and list of **select agents and toxins** (CDC): https://www.selectagents.gov/sat/list.htm	Not Applicable	
If you used a select agent, is the security level of the lab appropriate and reported in the manuscript?	Not Applicable	
If a study is subject to dual use research of concern regulations, is the name of the **authority granting approval and reference number** for the regulatory approval provided in the manuscript?	Not Applicable	

Reporting

The MDAR framework recommends adoption of discipline-specific guidelines, established and endorsed through community initiatives. Journals have their own policy about requiring specific guidelines and recommendations to complement MDAR.

Adherence to community standards	Information included in the manuscript?	In which section is the information available? (Reagents and Tools Table, Materials and Methods, Figures, Data Availability Section)
State if relevant guidelines or checklists (e.g., **ICMJE, MIBBI, ARRIVE, PRISMA**) have been followed or provided.	Yes	REMARK
For **tumor marker prognostic studies,** we recommend that you follow the **REMARK** reporting guidelines (see link list at top right). See author guidelines, under 'Reporting Guidelines'. Please confirm you have followed these guidelines.	Yes	
For **phase II and III randomized controlled trials**, please refer to the **CONSORT** flow diagram (see link list at top right) and submit the CONSORT checklist (see link list at top right) with your submission. See author guidelines, under 'Reporting Guidelines'. Please confirm you have submitted this list.	Not Applicable	

Data Availability

Data availability	Information included in the manuscript?	In which section is the information available? (Reagents and Tools Table, Materials and Methods, Figures, Data Availability Section)
Have **primary datasets** been deposited according to the journal's guidelines (see 'Data Deposition' section) and the respective accession numbers provided in the Data Availability Section?	Not Applicable	
Were **human clinical and genomic datasets** deposited in a public access-controlled repository in accordance to ethical obligations to the patients and to the applicable consent agreement?	Not Applicable	
Are **computational models** that are central and integral to a study available without restrictions in a machine-readable form? Were the relevant accession numbers or links provided?	Not Applicable	
If publicly available data were reused, provide the respective **data citations in the reference list**.	Not Applicable	
